# RNA-seq reveals distinctive RNA profiles of small extracellular vesicles from different human liver cancer cell lines

**DOI:** 10.18632/oncotarget.20503

**Published:** 2017-08-24

**Authors:** Martina Berardocco, Annalisa Radeghieri, Sara Busatto, Marialucia Gallorini, Chiara Raggi, Clarissa Gissi, Igea D’Agnano, Paolo Bergese, Armando Felsani, Anna C. Berardi

**Affiliations:** ^1^ U.O.C. of Immunohaematology and Transfusion Medicine, Laboratory of Stem Cells, Spirito Santo Hospital, Pescara, Italy; ^2^ Department of Molecular and Translational Medicine, University of Brescia, Brescia, Italy; ^3^ Department of Pharmacy, University G. d’Annunzio, Chieti, Italy; ^4^ Center for Autoimmune Liver Diseases, Humanitas Clinical and Research Center, Rozzano, Italy; ^5^ Institute of Cell Biology and Neurobiology, CNR, Monterotondo, Italy; ^6^ Genomnia Srl, Bresso, Italy

**Keywords:** extracellular vesicles, liver cancer, microRNA, small nucleolar RNA, RNA sequencing

## Abstract

Liver cancer (LC) is one of the most common cancers and represents the third highest cause of cancer-related deaths worldwide. Extracellular vesicle (EVs) cargoes, which are selectively enriched in RNA, offer great promise for the diagnosis, prognosis and treatment of LC. Our study analyzed the RNA cargoes of EVs derived from 4 liver-cancer cell lines: HuH7, Hep3B, HepG2 (hepato-cellular carcinoma) and HuH6 (hepatoblastoma), generating two different sets of sequencing libraries for each. One library was size-selected for small RNAs and the other targeted the whole transcriptome. Here are reported genome wide data of the expression level of coding and non-coding transcripts, microRNAs, isomiRs and snoRNAs providing the first comprehensive overview of the extracellular-vesicle RNA cargo released from LC cell lines. The EV-RNA expression profiles of the four liver cancer cell lines share a similar background, but cell-specific features clearly emerge showing the marked heterogeneity of the EV-cargo among the individual cell lines, evident both for the coding and non-coding RNA species.

## INTRODUCTION

Human liver cancer (LC) is among the most common forms of cancer and has a dismal clinical outcome, accounting for the third highest cause of cancer-related deaths worldwide [1]. The severity of LCs and the lack of good diagnostic markers and treatment strategies have rendered the disease a major challenge [2, 3]. It should be underlined that detection at an early stage of development of the disease does significantly increase the 5-year survival rate. Therefore, it is of great interest to develop molecular and cellular diagnostic assays with the potential to aid early diagnosis, clinical decision-making, and patient management [4]. From a clinical viewpoint, the ideal human liver cancer biomarker is one that enables clinicians to diagnose asymptomatic LC patients and which can be widely used in screening processes. Advances in translating cancer genomics into clinical oncology strongly indicate that it is essential to move to predictive models that are personalized and based on molecular classification and targeted therapy. The personalized approach to clinical care promises to increase the efficacy of treatment while reducing its toxicity and cost.

Non-coding (nc)RNA is a functional RNA molecule that is not translated into a protein. Accumulating findings have demonstrated that many ncRNAs such as microRNAs (miRNAs) and small nucleolar (sno)RNAs play diverse biological regulatory functions in many life events and are implicated in cancer progression [5, 6]. It is known that miRNA participate in the development of LC and that they could serve as potential diagnostic and therapeutic marker for LC. In liver carcinogenesis, miRNAs have been found to have both tumor suppressive (miR-122, miR-21, miR-34a) and oncogenic (miR-17-92 family) activity [5, 6]. Multiple, distinct, mature miRNA types, termed isomiRs, can arise from the same hairpin arm, as revealed by recent advances in miRNA transcriptome profiling [7]. These sequence variants differ from the mature miRNA sequence at either 5’ or 3’ ends, thereby increasing the diversity and complexity of the miRNAome. [8]. While the biological relevance of isomiRs is not fully understood, increasing evidence suggests that a proportion of isomiRs are related to the disease state, possibly due to differences in stability and turnover [9-13]. snoRNAs are small RNA molecules, approximately 60 to 300 nucleotides long, which generally serve as guides for the catalytic modification of ribosomal RNAs [14, 15]. Many snoRNAs have been described as retrogenes [16] and some are processed to a small RNA which can perform miRNA function [15]. Although few data have been experimentally verified, growing evidence indicates an association between snoRNAs and various diseases, and involvement in several types of cancer including liver cancer [14]. In addition, recently, it has been reported that liver cancer development and progression is also associated with several extracellular miRNAs encapsulated in vesicles, that may serve as candidate for biomarker [17].

Recently, small (nanosized) extracellular vesicles (EVs) have emerged as novel entities, which play a fascinating role in cancer progression and therapy, including liver cancer [17-19]. EVs are lipid bilayer membrane-enclosed vesicles released by cells as mediators for intercellular communication. They are very heterogeneous in size (ranging from ∼50 nm to > 1μm, with the vast majority <200 nm) and in molecular composition, carrying functional proteins, DNA, mRNA, ncRNA and lipids. Tumor-derived EVs have been intensively studied recently as novel microenvironment modulators because they may promote tumor-cell migration, invasion, formation of distant metastatic niches. Identification and characterization of liver-derived EVs may permit the development of new diagnostic approaches to screen for liver cancers, and may even have significant wider applications across a broad range of cancer treatments.

In this study, we used RNA-seq to provide the first comprehensive overview of the expression profiles of coding and non-coding transcripts, microRNAs, isomiRs and snoRNAs carried by the EVs derived from 4 different human liver-cancer cell (LCC)-lines. Form our data clearly emerges, together with certain shared characteristics, the heterogeneity across the 4 cell-lines of the small EVs RNA cargoes, evident from the disparity of the miRNAs, isomiRs, snoRNAs and gene expression profiles.

## RESULTS

### Identification and characterization of small EVs secreted by HuH7, Hep3B, HepG2 and HuH6 cells

**Figure 1:**
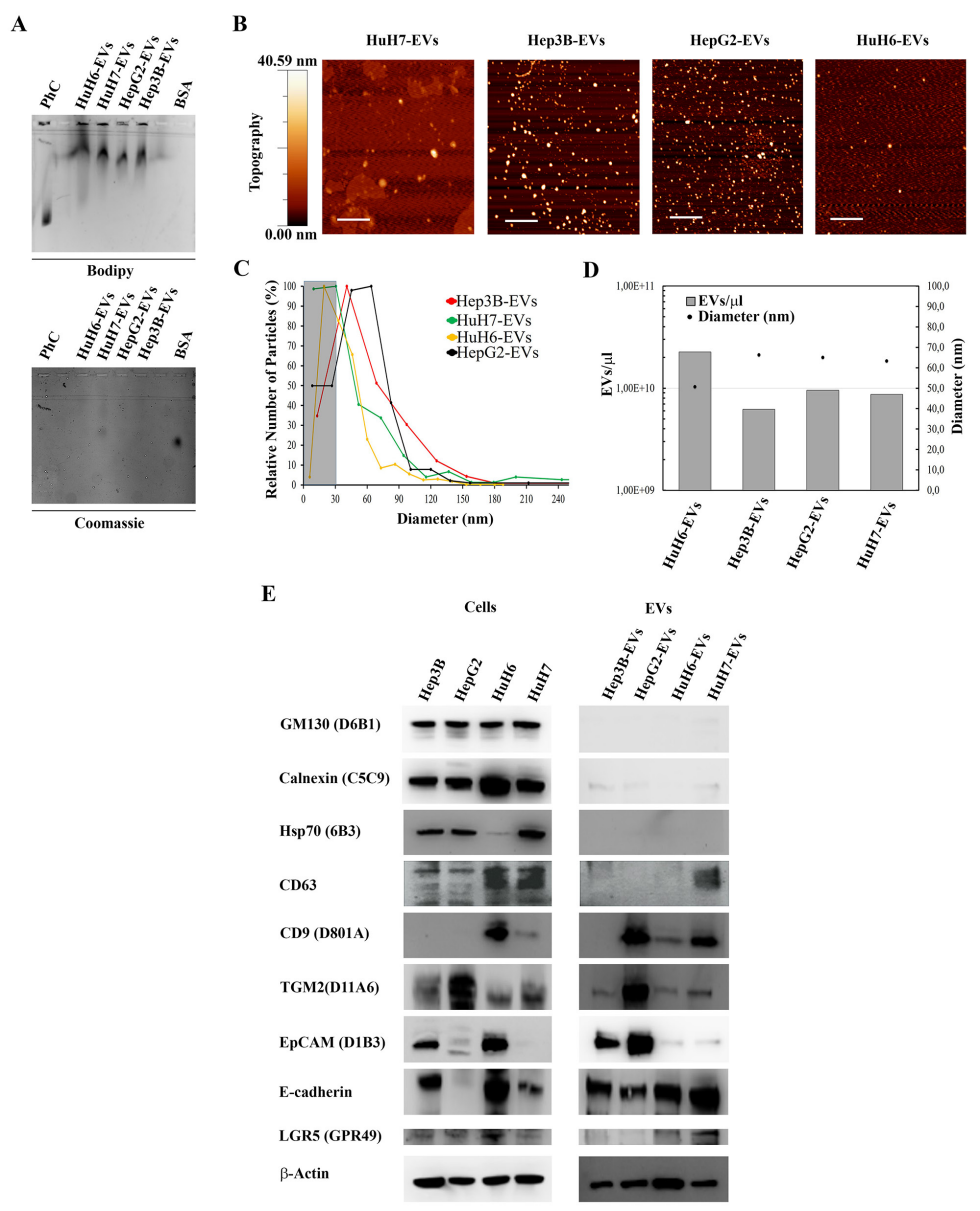
Characterization of human LCC derived extracellular vesicles (HuH7-EVs, Hep3B-EVs HepG2-EVs and HuH6-EVs) **(A)** EV preparations electrophoretic mobility: 1-palmitoyl-2-oleoyl-sn-glycero-3-phosphocholine (POPC) liposomes (270 nM) and vesicle samples were stained with green fluorescent dye (BODIPY FL C5-HPC) and run on a 0.6% agarose gel together with BSA solution (1μg/μL). Gel was also stained with Coomassie Brilliant Blue dye to evidence samples protein content and possible exogenous contaminants. **(B)** AFM topography image of the four EV preparations. Scale bars are 1 μm. **(C)** Size distribution of EV samples. Around 150-250 objects with a diameter between 0 and 250 nm were analyzed for each preparation using the WSxM 5.0 software. The EV diameter (nm) of each EV population was plotted against the relative number of particles (%). Objects with a diameter lower than 30 nm were not included in the analysis (darken plot area). **(D)** For each sample, weighted mean diameter values (nm) have been plotted together with sample concentration values (EV/μL) obtained by nanoplasmonic colorimetric assay [45]. **(E)** Representative western blots showing the expression of: GM130, calnexin, Hsp70, CD63, CD9, TGM2, EpCAM, E-cadherin, LGR5 and β-actin in LCC and LCC-derived EVs. Experiments were performed with similar results.

In order to obtain a comprehensive picture of the RNA types transported by liver cancer cell-derived EVs, we isolated small EVs from the conditioned media of four liver cancer cell-lines using a previously published and validated sequential centrifugation protocol [15]. These specific cell-lines were selected according to the cancer types they were derived from, i.e. hepatocellular carcinoma (HCC: HuH7, Hep3B and HepG2), and hepatoblastoma (HB: HuH6), in order to reflect the most common subtypes and heterogeneity of liver cancers. Studies have shown that these cell-lines tend to mirror both the genomic heterogeneity and the recurrent genome copy number abnormalities found in the primary liver tumors [20, 21]. To first assess the presence of EVs in the formulations and their purity we analyzed them by gel electrophoresis. EV lipid membranes were labeled by fluorescent dye Bodipy Fl, while Coomassie Brillant Blue was used for staining possible exogenous protein contaminants (Figure 1A). All the EV samples showed comparable electrophoretic mobility (Figure 1A upper agarose gel), distinct from monodispersed synthetic liposomes and with smeares ascribable to size polidispersity. Coomassie signal was lightly positive only in HuH7-EV (Figure 1A, bottom agarose gel), indicating traces of contaminants only in this sample.

EV morphological properties and size distribution were also investigated by atomic force microscopy (AFM). Figure 1B shows representative topography images of each sample, indicating that they were composed of EVs with sizes ranging from tens to a few hundreds of nm. The Hep3B, HepG2 and HuH6-EV samples showed a dark background without any relevant features other than EVs, confirming the purity of the preparations, while the HuH7-EV sample showed some background islands, indicating the presence of small amounts of exogenous protein, consistent with the gel results. The size-distribution of the vesicles was similar in all 4 samples, ranging from 30 nm to 240 nm, and peaking at the size of 60-70 nm (Figure 1C), which, in accordance with the latest convention on nomenclature, can be referred to as “small extracellular vesicles” [22]. The HuH6-EV sample contained the smallest vesicles, with a weighted mean size of 50 nm, whereas the other samples contained 10% larger EVs (65 nm).

A quantitative estimation of the molar concentration of EVs (Figure 1D) was obtained by applying a colorimetric nanoplasmonic assay we recently developed [23]. Results showed that HuH6-EVs sample was the most concentrated, with about 2,3×10^10^ EVs/μl, whereas the Hep3B-EVs was the lower, with about 30% less EVs/μl (about 6,2×10^9^ EVs/μl)

Finally, the four EV samples were analyzed for the presence of specific EV biomarkers by Western blot. Figure 1E shows that the EV preparations were devoid of intracellular debris contamination, since Calnexin and GM-130 (which are markers for the endoplasmic reticulum and the cis-Golgi, respectively), were absent or negligible with respect to the cell line lysates. All the four EV-samples expressed typical EV protein biomarkers as well as biomarkers previously associated with liver and related cancer types, although at different levels as shown in Figure 1E. TGM2, a candidate marker for hepatocellular carcinoma [24-26] and EpCAM (epithelial cell adhesion molecule) were highly expressed in Hep3B-EVs and HepG2-EVs, but only faintly in HuH7-EVs and HuH6-EVs. E-cadherin was clearly expressed in all samples, while the leucine-rich, repeat-containing, G protein-coupled receptor 5 (LGR5) was expressed only in HuH7- and HuH6-EVs.

### Identification of the most enriched gene transcripts carried by the extracellular vesicles derived from the four liver-cancer cell-lines

To identify the main species of RNA contained in the EVs derived from liver cancer cells, total RNA was extracted from each EV sample. These RNAs were used to generate two different sets of sequencing libraries: one library was size selected to be enriched for small RNAs (small RNA library) and the other was prepared for the whole transcriptome (WTA library). For the WTA libraries, reads mapped to the human genome (GRCh38/hg38) include ribosomal RNA sequences, LINE and SINE repeated sequences and specific gene transcripts, with the exclusion of miRNAs (Supplementary Figure 1). We selected transcripts that account for at least 0.05% of the counts in at least one cell line to exclude poorly expressed RNAs (Supplementary Table 1). Using this threshold level, we identified a total of 350 RNAs expressed in the EVs across all liver-cancer cell-lines. Of those 238 were protein coding, 61 belonged to snoRNA and 35 were classified as lincRNA. Of the protein coding RNAs, 35 were mRNA for ribosomal proteins, 16 coding for small subunit and 19 for large subunit proteins. Six components of the nonsense-mediated mRNA decay mechanism were also present.

**Figure 2:**
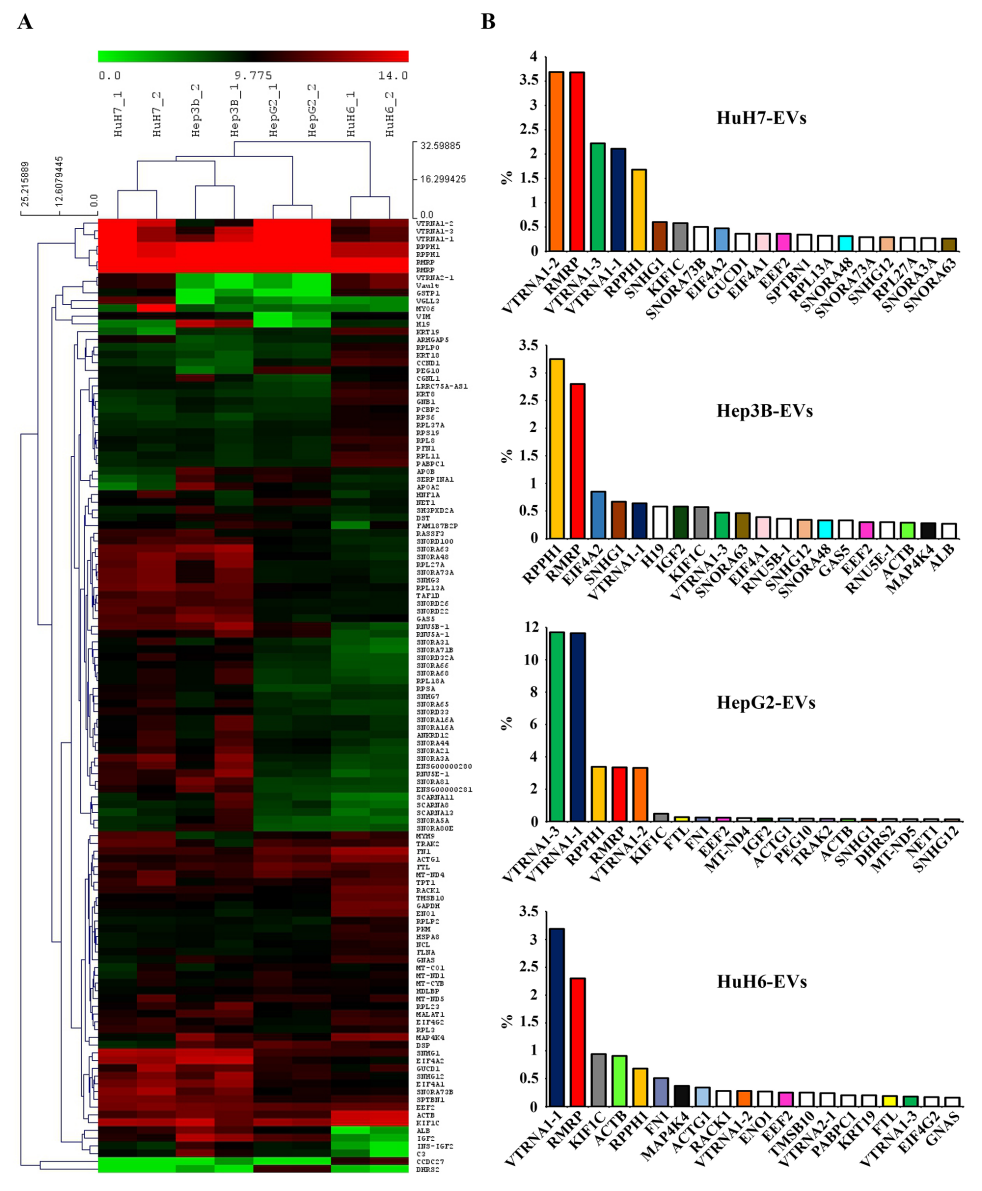
**(A)** Hierarchical cluster of the gene expression profiles of the RNAs carried by EVs of the 4 LC cell-lines. The graphic shows only the long coding and non-coding transcripts reaching the 0.1% expression level at least in one cell line. Red and green indicate the log2 expression levels according to the scale shown above the picture. **(B)** The 20 most abundant RNAs in the EVs from each cell-line, expressed as a percentage of total transcripts. The colored columns identify transcripts found in more than one cell-line; white columns show transcripts found only in one specific cell-line.

Figure 2A show these RNAs hierarchically clustered, limiting the analysis only to the transcripts attaining the 0.1% level at least in one cell line for sake of clarity. The biological replicates of EV-carried transcripts of the four cell-lines clustered together with each other and separately for each cell type, indicating that their RNA populations, although partially overlapping possess clearly distinct profiles of expression. It can be easily seen that HuH7 and Hep3B have very similar EV RNA expression profiles. HepG2 and HuH6 EV profiles are distinct from each other and from the first two cell lines. Figure 2B shows the 20 most abundant RNA in each cell line, expressed as percentage of the total transcripts, showing molecules found in the EV derived from more than one cell-line are shown as colored columns of the histogram, while non-colored columns represent those transcripts that were unique for a single cell line. Five non-coding genes, RMRP, RPPH1, VTRNA1-1, VTRNA1-2, and VTRNA1-3 are highly expressed in all EVs analyzed (accounting for almost one third of the total HepG2 EV-RNA), as was also shown in the hierarchical cluster of panel A. It is noteworthy that all 5 transcripts represent non-coding small-RNAs that are components of nucleoproteic aggregates, with the first two being ribozymes. Other highly represented transcripts are non-coding nucleolar and nuclear RNAs present in most of the lines (SNHG1, SNHG12) or only in some lines (SNORA63, SNORA73b, SNORA48, RNU5E-1). Among the coding RNAs particularly abundant and present in more than one cell line are KIF1C, ACTB, ACTG1, EEF2 and MAP4K4. From the size of the RNA molecules contained in EVs (see Supplementary Figure 2) it is possible to hypothesize that some of these mRNAs are potentially translatable.

It is also noteworthy that many of the highly represented transcripts, both coding and non-coding, are snoRNA host-genes (SNHG1, SNHG12, EIF4A2, GAS5, RPL13A, etc.), thus arising the question whether the vesicles contain the complete transcripts or only their relevant snoRNAs. To answer this question and to validate some of these findings a different RNA measuring technique, the RT-qPCR, was used on total RNA derived from EV collected from cell medium and cells bodies of parallel cell cultures.

**Figure 3:**
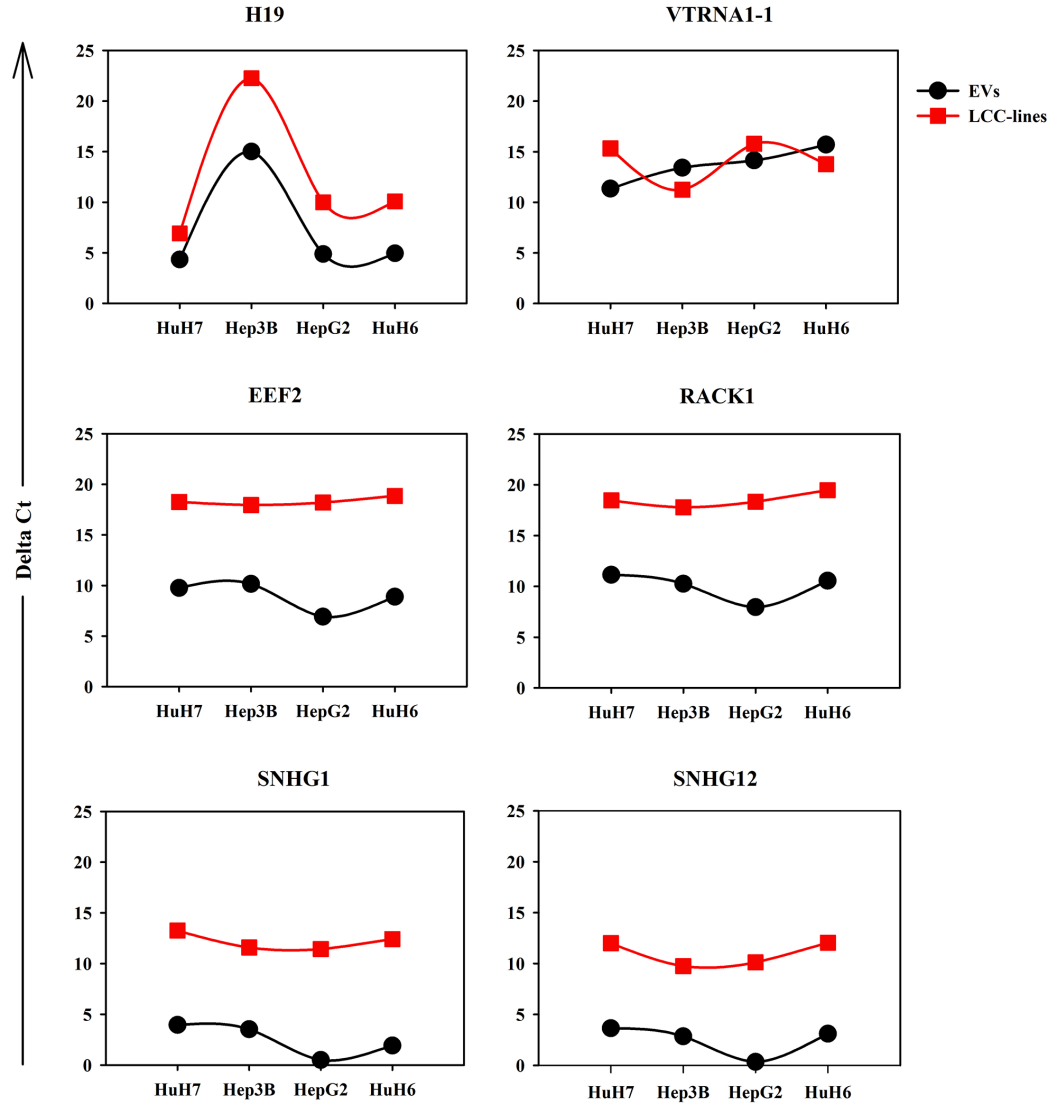
RT-qPCR analysis of the expression levels in LCC-EVs and LCC-lines of the following 6 transcript: H19, VTRNA1-1, EEF2, RACK1, SNHG1, SNHG12 Each panel shows two dot plots reporting the expression data in LCC-EVs and LCC-lines relative to the indicated RNA transcript. Black series: LCC-EVs; red series; LCC-lines. Data are from biological duplicates and each measurement was performed in triplicate. Standard error bars are comprised within the area of the square and round symbols.

Figure 3 shows the results obtained for 6 transcripts representative of the various classes of RNA most expressed in the LCC-EVs. The expression profile of some non-coding transcripts is in good agreement with what has been found in the EV by RNA-seq. For example, H19 is found most expressed in Hep3B-EV by both methods and its accumulation in cell bodies follows the EV levels. VTRNA1-1 levels also are like those found by RNA-seq, it is most present in HuH6-EVs, and its expression level in the cells is comparable. On the contrary, the accumulation in the EVs of the coding transcripts examined (EEF2 and RACK1) is less reproducible and for both genes the expression level is much higher in the cells, suggesting that the coding RNA presence in EVs depends by a poor regulated mechanism. Finally, the behavior of the two snoRNA host genes examined is peculiar: both are almost not expressed in the EVs and highly expressed in the cells, indicating that their detection by Lifescope software in the LCC-EVs by RNA-seq was due essentially to their snoRNA moiety.

### High diversity and specificity of miRNA content of EVs derived from the four liver-cancer cell-lines

For the study of the miRNA populations contained in the EVs secreted by each liver cancer cell line, we generated small-RNA sequencing libraries starting from total RNA extracted from the EVs released in the growth medium. The libraries were size selected to be enriched for the miRNA fraction and then sequenced on the SOLiD 5500xl platform. After excluding low-quality reads and trimming adaptor sequences, the remaining reads were first mapped to the human genome (GRCh38/hg38) and then to miRbase (v. 20), to annotate known miRNAs in each library. The metrics and the results of this procedure for all the samples are reported in Supplementary Figure 3.

An average expression level of 0.05% in at least one cell line was taken as a threshold to exclude poorly-expressed miRNAs. Using this threshold level, we identified a total of 167 miRNAs in the EVs across all the LCC-lines analyzed (Supplementary Table 2).

**Figure 4:**
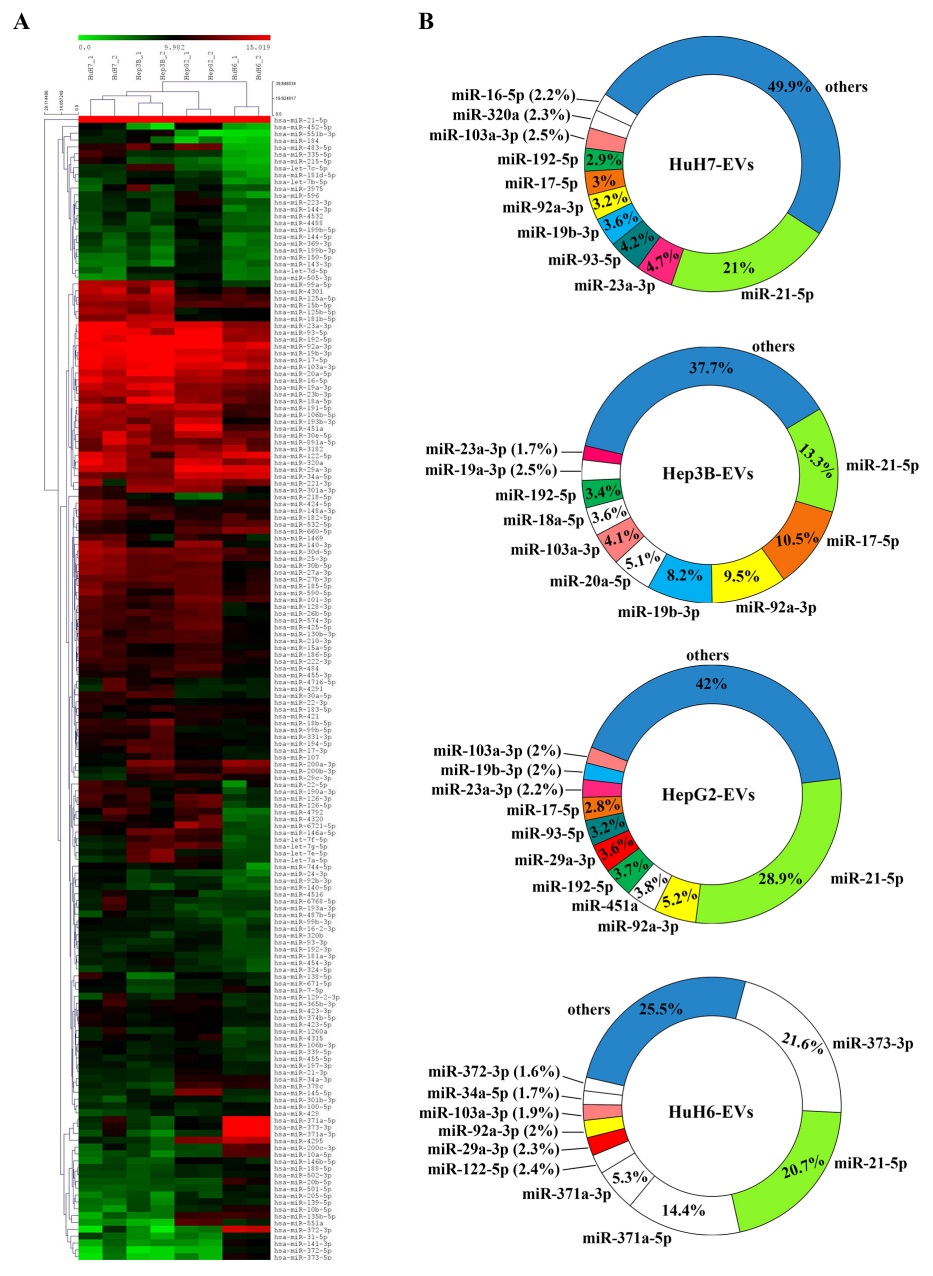
**(A)** Hierarchical cluster of the expression profiles of the miRNAs carried by the EVs of the 4 cell-lines. The graphic shows only the miRNAs reaching the 0.05% expression level at least in one cell line. Red and green indicate the log2 expression levels according to the scale shown above the picture. **(B)** The 10 most abundant miRNAs contained in the EVs derived from the 4 cell-lines. The colored sectors of the donut charts identify miRNAs found in more than one cell-line; white sectors show miRNAs found only in one specific cell-line. Hepato-cellular carcinoma (HuH7, Hep3B and HepG2) and hepatoblastoma (HuH6) cell lines.

To compare and visualize the miRNA transcriptomes of EVs produced from liver-cancer cell-lines, we performed a hierarchical cluster analysis. As shown in Figure 4A, the biological replicates of EV-derived miRNA profiles of the four cell-lines clustered together and independently for each cell line, the HuH7 close to the Hep3B, then the HepG2 and finally the HuH6, the most divergent from the other cell lines. This indicates that the EVs derived from different LCC-lines possess cell-line specific assortments of miRNAs. Nevertheless, a group of at least 14 miRNAs are present at high concentration in all the LCC-EVs. This group include hsa-miR-103a-3p, hsa-miR-106b-5p, hsa-miR-122-5p, hsa-miR-16-5p, hsa-miR-18a-5p, hsa-miR-193b-3p, hsa-miR-19a-3p, hsa-miR-20a-5p, hsa-miR-23b-3p, hsa-miR-29a-3p, hsa-miR-30e-5p, hsa-miR-320a, hsa-miR-34a-5p, hsa-miR-451a. Analyzing their targets using Diana Tools mirPath v3 [27], all these miRNAs were incorporated in the Kegg pathway ‘Pathways in cancer’ (hsa05200), that controls cellular processes like “Sustained angiogenesis”, “Evading apoptosis”, “Proliferation”, “Tissue invasion and metastasis”, “Block of differentiation”, “Resistance to chemotherapy”.

To clearly describe what makes the different EV-types unique in their miRNA content, in Figure 4B we showed the 10 most expressed miRNAs, ordered according to their abundance. Interestingly, for all the four liver-cancer cell lines these 10 most abundant miRNAs represented from 50% (HuH7) up to about 75% (HuH6) of the EV-cargo of miRNAs. MiR-21-5p was the most abundant miRNA across all four cell-lines, holding the first position in HuH7, Hep3B and HepG2 and the second one in HuH6. Other miRNAs were also present consistently across all four cell lines, such as miR-92a-3p and miR-103a-3p.

High amounts of these four following miRNAs were present in all EVs except those derived from the HuH6 cell-line: miR-19b-3p; miR-17-5p; miR-192-5p; and miR 23a-3p. As noted above, the HuH6 EVs carried the most specific miRNA load, missing or carrying at lower concentration some miRNA particularly abundant in the EVs of other cells, but showing several uniquely enriched miRNAs as follows: miR-372-3p; miR-371a-3p; miR-371a-5p; miR-373-3p; miR-34a-3p and miR-122-5p

The EV-cargos did show some specificity with the detection of miRNAs expressed at high levels in only one line: miR-16-5p in HuH7-EVs only; miR-18a-5p and miR-20a-5p in Hep3B-EVs only and miR-451a in HepG2-EVs only.

**Figure 5:**
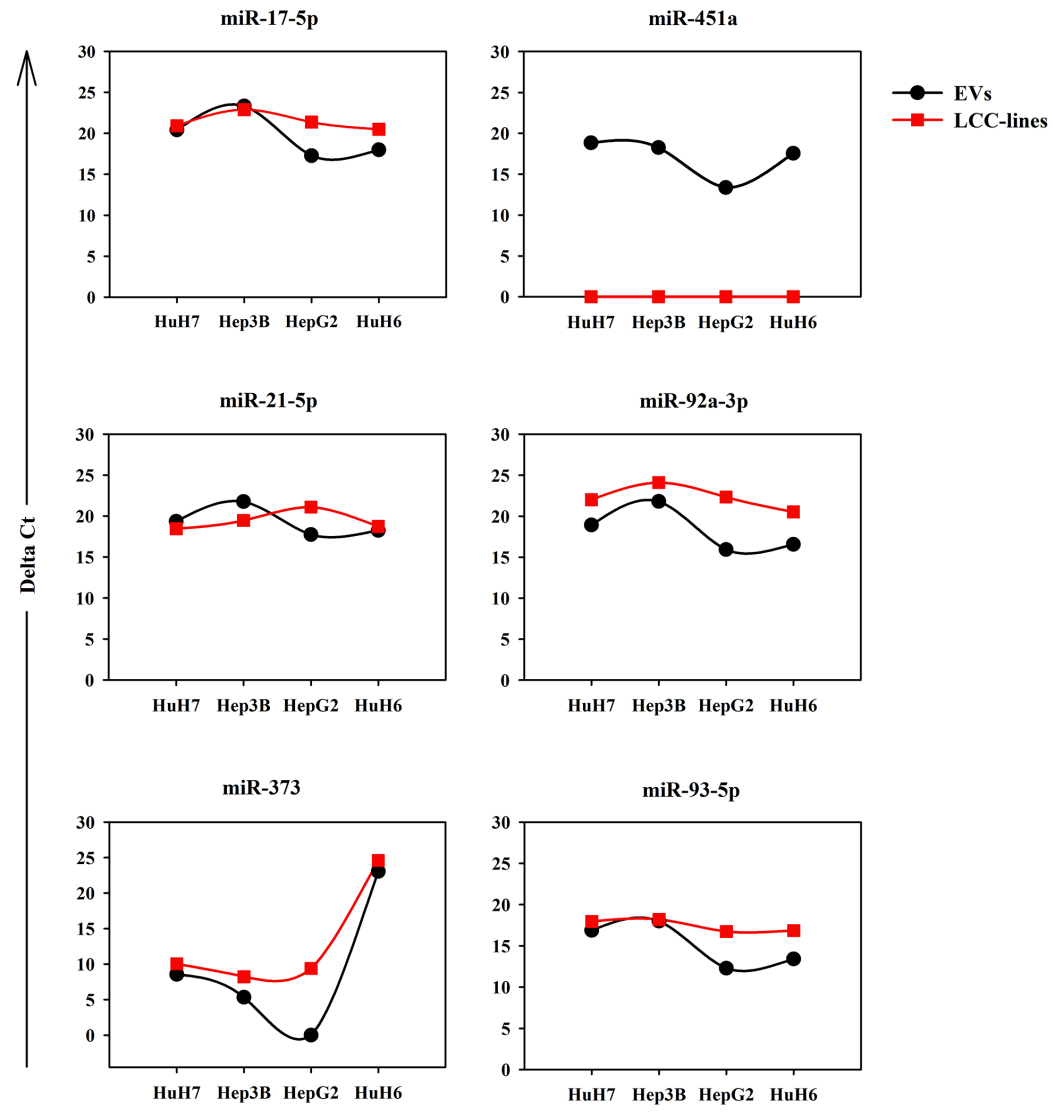
RT-qPCR analysis of the expression levels in LCC-EVs and LCC-lines of the following 6 miRNAs: miR-17-5p, miR-451a, miR-21-5p, miR-92a-3p, miR-373, miR-93-5p Each panel shows two dot plots reporting the expression data in LCC-EVs and LCC-lines relative to the indicated miRNAs. Black series: LCC-EVs; red series; LCC-lines. Data are from biological duplicates and each measurement was performed in triplicate. Standard error bars are comprised within the area of the square and round symbols.

The expression data obtained by RNA-seq of some of the miRNAs were validated by using RT-qPCR on parallel batches of total RNA derived from LCC EVs and whole cells. The analysis of the cellular RNA was performed to see how similar were the EV-miRNA and the cellular-miRNA expression profiles. Figure 5 shows both series of data for 6 miRNAs chosen among those highly abundant in the EVs derived from at least one LCC. The validation of the RNA-seq data was achieved partially with 3 miRNAs only, miR-17-5p, miR-92a and miR-373. Two are the possible explanations of this fact: (i) the heterogeneity of the miRNA expression profiles of the biological replicates, detectable also in the RNA-seq data, as shown in the cluster reported in Figure 4A; (ii) the large number of isomirs found for each canonical miRNA (see next paragraph) could hindrance the performance of the specific Taqman miRNA PCR assays designed to detect mainly the canonical miRNA.

More interesting are the results concerning the accumulation of the same six miRNAs in the cells producing the EVs (Figure 5). For most of the LCC, maybe except for the HepG2, the deltaCT of the specific miRNA in the EVs is similar to the deltaCT of the same miRNA in the corresponding cells. The quantitative data are comparable since we used for all the assays the same amount of total RNA. There is the notable exception of miR-451a well detectable in all the EVs but completely absent in the cell bodies. This result suggests that miR-451a does not accumulate in the cell where it is transcribed but is immediately exported into the EVs.

### The EVs released by the four liver-cancer cell-lines transport a rich and highly specific isomiR cargo

Most miRNAs comprise multiple sequence isoforms (termed isomiRs) [8, 28, 29]. These sequence variants differ from the canonical mature miRNA sequence deposited in the miRBase by the addition or trimming of nucleotides at either end and may also carry internal nucleotide substitutions.

By applying the procedure described in detail in Material and Methods, a total of 6,953 different isomiRs were identified in the EVs of the four cell lines. Of those, 421 were expressed at a level greater than 0.05% at least in one LCC line and are reported in the Supplementary Table 3A, together with the isomiR naming rules and their sequences. Considering the total miRNA content of the EVs, the isomiRs represent about 26-30% of all these reads across all the LCC-lines (see Supplementary Table 4).

We focused our attention on the isomiRs of the 10 most abundant canonical miRNAs for each cell line, as listed in the previous paragraph and reported in Supplementary Table 3B. As shown in Figure 4, left panel, the isomiRs of miR-21-5p were collectively the most abundant in all the LCC-EVs analyzed. But individual cell lines show specific isomiR profiles: in HuH6 miR-371a-5p and miR-371a-3p isomiRs are the most abundant, and are quite exclusively carried by the EVs generated by these cells. In HuH7-EVs miR-23a-3p isomiRs are the most abundant. miR-17-5p isomiRs are particularly enriched in Hep3B-EVs and miR-451a isomiRs in HepG2-EVs.

**Figure 6:**
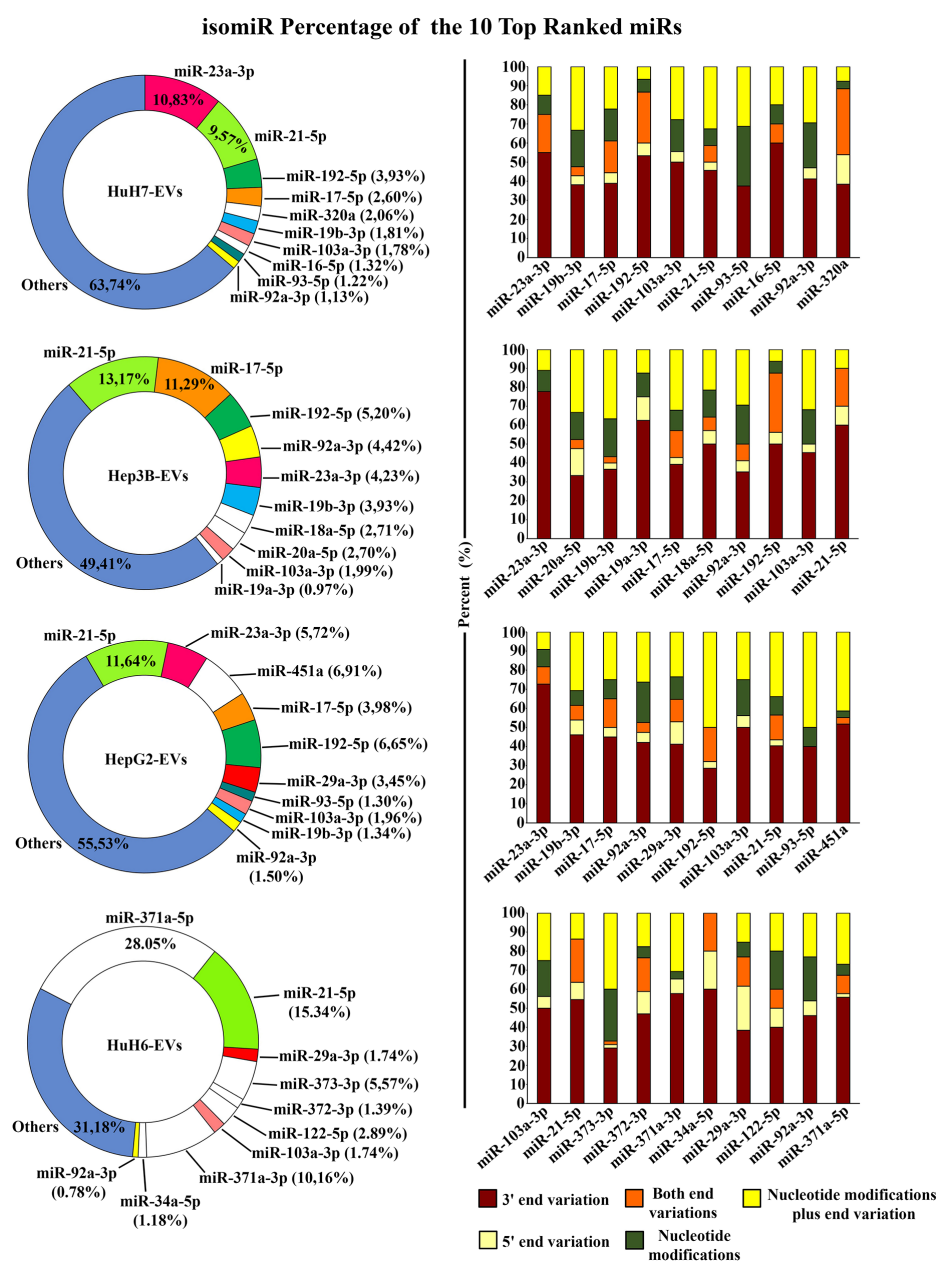
isomiRs of the 10 most abundant miRNAs The donut charts on the left show the expression level of isomiRs of the 10 most abundant miRNAs found in the EVs derived from the 4 LCC lines; white sectors show miRNAs found only in one specific cell-line. The histograms on the right represent for each individual miRNA the percentage of the variations types. The most abundant isomiR differing in sequence from the canonical miRNA at 5’ end only (show in beige), at 3’ end only (in dark brown), or at both ends (in orange), internal nucleotide variations (in green) and nucleotide variations plus end variation (in yellow).

Figure 6, right panel, illustrates the frequency of the modification types shown by each individual miRNA in the different cell lines. A marked preponderance of 3′ end variations is common to all cell lines, while the more critical 5’ end variations are less frequent. The nucleotide modifications, either alone or in combination with end variations, are quite frequent, accounting for almost one third of the total isomiRs. Overall, examining the specific miRNAs, it appears that the frequency of the different modification types is more linked to the cell line than to the individual miRNA species.

### Specific sets of snoRNAs are carried by the EVs derived from the four liver-cancer cell-lines

Since the de-regulation of snoRNA expression is increasingly linked to cancer [30], we determined the contribution of snoRNAs to the cargo of nuclei acids carried by the EVs produced by liver cancer cells. Since snoRNAs vary in length ranging from 60 to 300 nt, we searched for them in both WTA and small RNA libraries. After normalization, the snoRNA expression levels were reported for the WTA library in Supplementary Table 5 and for the small RNA library in Supplementary Table 6. Considering the library construction protocol (se Material and Methods), it is likely that the snoRNAs found in the small RNA libraries were present in the EVs as fragments.

**Figure 7:**
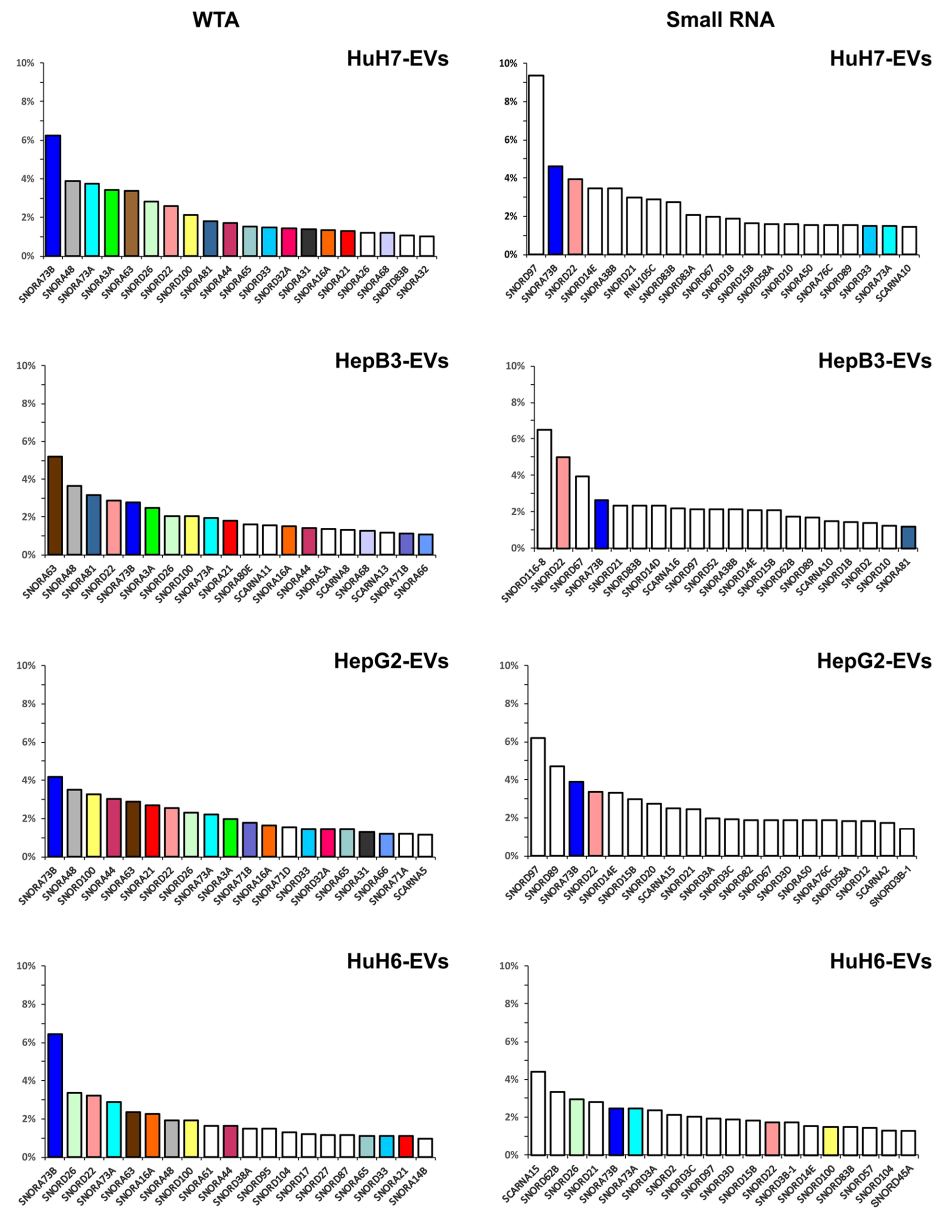
The 20 most abundant snoRNAs contained in EVs derived from the 4 LCC-lines The colored columns identify the snoRNAs found in more than one cell line, white columns show snoRNAs found only in one specific cell line. Hepato-cellular carcinoma (HuH7, Hep3B and HepG2) and hepatoblastoma (HuH6) cell lines.

In Figure 7 shows the 20 most expressed snoRNAs, ranked based on their abundance, from both the WTA and the small RNA libraries. The snoRNA abundance distribution is quite shallow, very different from that of the miRNAs. In fact, the most abundant 10 miRNAs accounted for more than 50% of the total miRNA amount, while the 20 most abundant snoRNAs do not attain 50% of the total.

The two types of libraries produced very different results. The 20 most abundant snoRNAs for each cell line, detected by the WTA libraries, account for a total of 38 species, 22 belonging to the H/ACA box type, 12 to the C/D box type, and only 4 to the Small Cajal body-specific RNAs (scaRNA). On the other side, their counterparts from the small RNA libraries account for a total of 41 species, 30 belonging to the C/D box type, only 6 to the H/ACA box type and 5 of the scaRNA type.

SNORA73B, SNORA73A, SNORA63 were well represented in all the EVs according the data from the WTA libraries, and are known to be among the most abundant snoRNAs in mammalian cells. Widely present were also SNORD26, SNORD22, SNORA48 and SNORD100. Again, the HuH6 EVs carried a cargo of snoRNA most different from the other cell lines, with many snoRNAs, as SNORA61, SNORD38A and SNORD95, expressed at higher level only in the EVs from this cell line. The snoRNAs detected in the small RNA libraries were only partially overlapping with those identified in the WTA libraries. Most of snoRNAs detected in small RNA libraries, as SNORD97, were expressed at high level only in these libraries.

## DISCUSSION

Previous studies have demonstrated that the hepatoblastoma-like (HuH6) and the hepatocellular-carcinoma-like (HuH7, Hep3B, HepG2) cancer cell-lines maintain the hepatocarcinogenic phenotype at gene, miRNA, and protein expression levels, and are useful to gain new insights into the pathogenesis of hepatoblastoma and hepatocellular carcinoma, providing novel biomarkers [20, 21].

The present study represents a detailed analysis of all the coding and non-coding transcripts carried by EVs derived from 4 LCC-lines, profiling gene expression through RNA-seq, and identifying large RNAs, microRNAs, isomiRs, and snoRNAs. To our knowledge, only one previous study characterized the EVs large RNA content derived from three different HCC cell lines [31] using NGS technology.

Biophysical analysis indicates that the EV populations have comparable physical properties, including morphology and size distribution. Our study also demonstrated that membrane composition and EV-cargo content differ across the 4 LCC-line-derived EV-populations, confirming several previous studies which have demonstrated that EV-membrane composition and cargo content differ greatly, depend on cell-type, and are usually regulated by it [32, 33].

We demonstrate the purity of the isolated EVs by western blot, excluding contamination by other membrane fragments. The western blots revealed the presence of EpCAM in all EVs and, to a greater extent, in Hep-3B and HepG2-EVs. EpCAM is a “stem-associated” marker and has been linked to poor outcome in HCC [34], suggesting that it could be used as a prognostic predictor to test for HCC recurrence in patients [35, 36]. We found TGM2 in the EVs released by all cell lines, in agreement with other studies demonstrating that TGM2 is a hepatoma-related protein and may be a candidate for use as an HCC marker [26]. E-cadherin, which plays fundamental roles in epithelial cell function and structure, was detected in the EVs from all LCC, thus confirming other reports [37]. E-cadherin is involved in cell-cell recognition, cell adhesion, and in the entry of Hepatitis C virus [38] and could play a role in the recognition and entry of LCC-EVs into their target cells. The leucine-rich, repeat-containing, G protein-coupled receptor 5 (LGR5), another marker of cell plasticity and stem cell potential in LC, is expressed only in HuH7- and HuH6-EVs. It has been suggested that LGR5-expressing HCC stem-like cells may play an important role for the pathogenesis and drug resistance of HCC [39]. It is possible that EpCAM-, E-cadherin- and LGR5-carrying EVs participate to the preparation of the microenvironment to accommodate the subpopulation of tumor stem like cells originating from the LCC.

The size-distribution of the RNA cargo of all the LCC-EVs, spanning from 25 up to 4000 nt, is well in accord with that reported in previous studies [31]. The transcripts we have identified in LCC-EVs have already been described as expressed in liver tissues and all play some role in protein metabolic processes, in cell differentiation, in certain immune system processes, and in cell death, cell invasion and cell proliferation.

The abundance in all EVs of vault RNAs is puzzling but has already been reported [40, 41]. The ribozyme RNA components RMRP and RPPH1 are also very abundant. It is very likely that all these RNAs are carried by the EVs as ribonucleoproteic particles, the normal state in which they exist in the cells. The presence of these non-coding structural RNAs and of two endoribonuclease complexes could be related to RNA-selection and EV-loading mechanisms.

Among the lncRNAs, H19 is the most documented in oncogenesis and aberrantly expressed in multiple malignancies and its expression levels correlate with recurrence, metastasis, and patient survival. Its presence in HCC-EVs has already been described [42, 43]. H19 is particularly abundant in Hep3B-EVs, but it is expressed at lower lever in all the LCC-EVs.

miRNA-analysis of the LCC-derived EV cargoes showed that some miRNAs were abundantly present in the EVs secreted from all cell lines, while certain other miRNAs were only abundant in the EV derived from one cell line. The most abundant commonly-shared miRNAs were known oncomiRs such as miR-21 [44], members of the miR17-92 cluster [45-47] and members of the miR-23a and miR-23b clusters [48]. It is worth noting that the expression of EV-contained miR-21 had already been found to be significantly higher in HB patients [49]. In HCC serum/plasma, the miR-21 had, again, also already been found to be over-expressed and it has been suggested that this miRNA could contribute to hepatocyte proliferation [50, 51]. Bearing this in mind, our results reinforce the hypothesis that miR-21 expression is a good candidate for a circulating, non-invasive, diagnostic and prognostic biomarker for LC. We also found miR-192-5p and miR-122-5p to be abundantly present in EV cargoes derived from LCC-lines. These had both already been suggested as being abundantly expressed in the liver and are believed to be serum biomarkers of hepatic injury [52]. Furthermore, several studies have identified miR-192 as an HCC diagnostic marker which is upregulated in EV-cargoes in HCC patients [53, 54]. Accordingly, our findings reinforce the suggestion that miR-192-5p and miR-122-5p could be the LC “miR-signature” in EV cargoes.

Only the HuH6-derived EV cargoes contained abundant levels of miR-372-3p, miR-371a-3p, miR-371a-5p and miR-373-3p. Since the miR-371/372/373 cluster has been identified as being specific to “human embryonic stem cells” (hESCs), these miRNAs are directly associated with embryonic carcinomas [55-57]. HB is considered to be an embryonal tumor probably originating from hepatoblasts and it has already been reported that miR-373 and miR371 are overexpressed in HB. In addition, it has been suggested that the miR-371/372/373 cluster only identifies aggressive HBs [58]. The high abundance of these miRNAs in HuH6-released EVs agrees with the fact that these cells derive from HB tumors, probably originating from embryonic liver stem cells [59, 60]. Accordingly miR-373, which had already been proposed as a blood-based biomarker for more aggressive tumors, may also be a candidate as an HB EV-cargo marker [61].

The expression data, produced by RNA-seq, of a specific set of EV-miRNAs has been validated by RT-qPCR, obtaining only a partial consensus between the measurements as reported in the Results section. More interesting, for the same set of miRNAs, the expression level in the EVs has been compared with that in the cells from which the vesicles originated. In general, apart from some slight variation, miRNA expression amount in EV-cargoes mirrored miRNA expression level in the source cell-line, except for miR-451a. This miRNA was present in all the LCC-EVs, but was undetectable in the LCC-lines, thus indicating the existence, at least for some miRNA, of a very efficient and selective EV-loading mechanism. miR-451a is encoded by a gene located on chromosome 17. It is already known that miR-451a acts as a tumor suppressor, down-regulated in many tumors, including LC [62, 63]. It has been experimentally found that miR-451a preferentially enter extracellular vesicles secreted from HEK293T cells [64]. Recently it has been suggested that tumor cells dispose onco-suppressor miRNA into EVs and outside them into the extracellular environment, to preserve their invasiveness and tumorigenic phenotype [65]. A similar mechanism could explain our finding about the compartmentalization of miR-451a expression into LCC-EVs.

The miRNA sequence diversity indicated that a range of variants were expressed in the EVs derived from the LCC cell lines, showing different and often composite sequence alterations. As far as isomiR ends are concerned, in conformity with previous studies [12, 66, 67], we observed positional variations at both termini. Both the number of individual variants and their contribution to miRNA expression indicated that 3′-modified isomiRs are the predominant category, consistent with the model that heterogeneity at the 5′ is expected to have a major impact on miRNA targeting [11]. Even though the exact function of 3′-end modifications is still under investigation, increasing evidence suggests that a proportion of isomiRs are related to disease state, possibly because of differences in stability and turnover [9, 11]. Beside 5’- and 3’-end modifications, internal editing of the canonical sequences was also found. The total ratio of the isomiR species versus their canonical miRNAs was relatively similar in all the LCC-lines, although the proportion of individual isomiRs sometime differed between the four LCC-derived EVs. In general, the isomiRs derived from the different cell lines were highly correlated with the canonical miRNAs present in the same EVs, supporting the [59, 60, 68] hypothesis that they would be likely to drive similar biology, similarly to what has already been suggested for cellular isomiRs [67].

Along with miRNAs, snoRNAs were the most highly-represented class of non-coding RNA in all the LCC-EVs. snoRNAs play important roles in the maturation of rRNA, tRNA, snRNA as well as in mRNA biogenesis [30]. snoRNAs may also be involved in human cancers as demonstrated by recent studies in lymphomas, leukemia and in human liver cancer [14, 69, 70]. snoRNAs have tissue-specific expression [30, 71, 72] and show altered expression in cancer cells with possible consequences on translation in these cells [30].

Many studies have proposed that fragments of full-length ncRNAs could acquire new functions [73, 74], and this has also been described for snoRNA fragments [75]. Our data indicate that the EV snoRNA cargo was composed both by full-length RNA, with a low persistence of yet unprocessed host genes, as detected by the WTA libraries, and by smaller fragments, as detected by the small RNA libraries. These two subpopulations only partially overlapped and their composition was very different in terms of H/ACA versus C/D box types. This finding dismisses the possibility that the fragments could be derived by the processing of larger molecules inside the EVs, suggesting two independent cellular origins for most of them. It is worth noting that of the 38 most abundant snoRNAs in LCC-EVs, only 9 belong to the most expressed snoRNA according to the ENCODE RNA-seq data. Among these SNORA73A and SNORA73B, abundantly found in both types of libraries, that have a non-canonical role in 18S rRNA maturation [76]. There is also evidence that SNORA73A could function as a regulator of chromatin function [77].

The study of the biological relevance of EVs in liver-cancer development and progression represents a research field in rapid growth [17, 19] and the data we collected on the complex and heterogeneous RNA cargo of the LCC EVs will be relevant for the understanding of the role of EV-transported nucleic acids in liver physiology and pathology.

## MATERIALS AND METHODS

### Cell culture

The following liver cancer-derived cell-lines were used in this study: HepG2, Hep3B, (ATCC, Manassas, VA, USA), HuH-7, HuH6 clone 5 (JCRB Cell Bank, Osaka, Japan). HuH7, Hep3B and HuH6 cells were grown in Dulbecco’s Modified Eagle Medium (DMEM) (Life Technologies, Carlsbad, CA, USA) while HepG2 cells were cultured in Eagle’s Minimum Essential Medium (EMEM) (Life Technologies). All media were supplemented with 10% fetal bovine serum (FBS) (Life Technologies), 1% l-glutamine, 1% penicillin/streptomycin (Invitrogen, Life Technologies, Carlsbad, CA, USA), in a 5% CO2-humidified chamber at 37°C.

### EV isolation

FBS and human serum albumin (hBSA) solution (Life Technologies) used for EV production were depleted from endogenous EVs prior to use by ultracentrifugation at 120,000 g for 5 hours, using an Ultracentrifuge Optima L-100K Ultracentrifuge (Beckmann Coulter, Pasadena, CA). Afetr centrifugation, the FBS and hBSA supernatants were filtered with a 0.22 μm filter (ThermoFisher Scientific, Waltham, MA, USA) and stored in aliquots at -80°C.

HuH7, Hep3B, HepG2 and HuH6 80%-confluent cell plates (1-2 ×10^6^ cells/mL) were washed 2 times with PBS (Life Technologies), then incubated for 24 hours in DMEM/F12 (1:1) (Life Technologies) supplemented with 1% EV-free FBS and 0.25% EV-free hBSA at 37°C and 5% CO2. Cell viability was assessed using trypan blue exclusion methods. Presence of apoptotic cells were checked by flow-cytometric assay for Annexin-V expression. EVs were prepared from the cell-culture media using differential centrifugation steps as previously described with some modification [15]. All preparation and centrifugation steps were performed at 4°C. Briefly, collected cell-media were subjected to a first centrifugation at 300×g for 10 min to remove non-attached cells, followed by a second centrifugation at 2,000×g for 30 min to remove apoptotic bodies (ABs), and finally a third centrifugation at 16,000×g for 20 min to remove residual ABs and cell organelles. EVs were then pelleted from the purified supernatant by centrifugation at 120,000×g for 70 min in 38 ml polycarbonate tubes (Beckman #355631). This EV-enriched pellet was resuspended in PBS and the centrifugation was repeated again as above. The final EV pellets was thoroughly drained, rapidly frozen in liquid nitrogen and stored at -80°C until the use.

### Phosphatidylcholine liposomes

Synthetic phosphatidylcholine (PCh) liposomes were prepared as previously described [23]. Briefly, for PCh liposomes preparation the proper amount of (1-palmitoyl–2-oleoyl-sn-glycero-3-phosphocholine, POPC, purchased from Avanti Lipids) was dissolved in chloroform/methanol 6:1 (v/v). A lipid film was obtained by evaporating the solvent under a stream of nitrogen and overnight vacuum drying. The film was then detached and broke apart in warm (50 °C) 0.9% NaCl solution by vigorous vortex mixing. To prepare vesicles with narrow distribution, the dispersion was tip-sonicated for 30 minutes and the number of vesicles was evaluated as described in Maiolo et al. [23].

### Nanoplasmonic colorimetric assay

EV preparations were checked for purity using a colorimetric nanoplasmonic assay developed by Maiolo et al. [23, 78]. The assay exploits the properties of a colloidal solution of gold nanoparticles (AuNPs) and EVs. The EV preparations were treated as follows. Pellets were re-suspended in 100 μL sterile H_2_O with protease inhibitors (1:1000), diluted 1:12,5 with deionized H_2_O and mixed with a final concentration of 3 nM AuNPs (15 nm). The blue shift was quantified by collecting the UV-Vis spectra of the different AuNP-EVs solutions. UV-Vis spectra were measured with an EnSight multimode plate reader (PerkinElmer) spectrophotometer and acquired with 1 nm step size in a wavelength window ranging from 400 nm to 900 nm. The AuNPs aggregation index (AI) was defined as the ratio of the LSPR absorption at 519 and 650 nm (AI = A519/A650).

### Fluorescent labeling

Liposomes and EVs were fluorescently labeled with 2-(4,4-Difluoro-5,7-Dymethyl-4-Bora-3a,4a-Diaza-s-Indacene-3-Pentanoyl)-1-Hexadecanoyl-sn-Glycero-3-Phosphocholine (BODIPY FL C5-HPC), a highly fluorescent lipid probe previously dissolved in ethanol up to a final concentration of 0,01 mg/mL. Briefly, 5 μL of BODIPY FL C5-HPC has been dried in a 1,5 mL Eppendorf. Then 90 μl of resuspended EVs preparations were added and incubated for 2 hours at room temperature under constant rotation. Finally, exosome labeled preparations were centrifuged at 100,000 × g for 2 h.

### Vesicle and protein detection on agarose gel

Liposomes and EVs were labeled with BODIPY FL C5-HPC membrane dye as described above. EVs enriched pellet were resuspended in 10 μL of TAE (TRIS 39 mM, acetic acid glacial 19,9 mM, EDTA 1,27 mM) plus 0.02% SDS and then loaded on a 0.6% agarose gel and electrophoresed 30 min at 100 V. Fluorescent signal was acquired using a G:Box Chemi XT Imaging system (Syngene). Then the gel was stained with Coomassie Brilliant Blue for 30 minutes at room temperature and destained in 10% acetic acid, 20% methanol overnight. Images were acquired using a G:Box Chemi XT Imaging system (Syngene).

### Atomic force microscopy (AFM) imaging

Each EV sample was diluted 1:100 with deionized water. Five to 10 μL of samples were then spotted onto freshly cleaved round shaped mica sheets (thickness 0.10 mm, diameter 9.9 mm). Mica substrates were dried at room temperature and analyzed using a NANOSURF NAIO AFM, equipped with Budget Sensors AFM tips (Multi75GD-G). Images were snapped in light tapping mode; scan size ranged from 1 to 25 μm and scan speed ranged from 0.8 to 1.2 ms x clock [67].

### Extracellular vesicles size distribution analysis

EVs size distribution analysis was performed with WSxM 5.0 software as previously published [79]. Briefly, for each sample, an AFM image (field 5 × 5 μm) containing at least 230 objects with a diameter between 30 and 1000 nm, was analyzed. Off-scale objects were not included in the analysis. EV perimeter was calculated using a specific algorithm; to simplify diameter evaluation, vesicles were assumed to be perfect spheres. After diameters analysis, to better represent big amounts of data, all values were grouped between intervals and plotted in a scatter graph against the number of vesicles counted for that interval (Figure 1C). For each EV sample, weighted mean diameter was calculated and plotted in diagram shown in Figure 1D together with EV samples content (EVs/μL). EVs/μL values were determined comparing AI values of the four samples with the AI of calibration curve made with liposomes obtained by nanoplasmonic assay [80]. Quantification values were obtainable due to the high purity of all EV preparations lacking protein-based contaminants as previously shown in Figure 1A.

### Protein preparation from cells and EVs

Proteins were isolated from HuH7, Hep3B, HepG2 and HuH6 cells and from EVs derived from the same liver-cancer cell-lines using M-PER Mammalian protein extraction reagent (ThermoFisher Scientific, Waltham, MA, USA) supplemented with 1% of Halt Protease and the EDTA-free, phosphatase-inhibitor cocktail (Thermo Fisher Scientific, Waltham, MA, USA) and 1% of EDTA solution (Thermo Fisher Scientific, Waltham, MA, USA), according to the manufacturer’s indication. The isolated proteins were stored at -20°C. The concentration of total protein preparations was quantified by the BCA assay (QuantiPro™ BCA assay kit, Sigma-Aldrich, Milano, Italy).

### Analysis of protein expression by western blotting

Cells and EV lysates (10 μg) were electrophoresed and transferred to nitrocellulose membranes. Membranes were then blocked in 5% non-fat milk or 5% BSA, 10 mM Tris-HCl pH 7.5, 100 mM NaCl, 0.1% Tween-20, and probed with the following primary antibodies (work dilution 1:1,000): anti-Hsp70, anti-Calnexin, anti-GM130, anti-CD9, anti-TGM2 (D11A6), anti-EpCAM (D1B3) and anti-E-cadherin all purchased from Cell Signaling Technology (Danvers, MA, USA); anti-CD63 and anti-β-actin purchased from Sigma-Aldrich (St. Louis, MO, USA); anti-LGR5 (-GPCR GPR49) purchased from ABCAM (Cambridge Science Park Cambridge,UK); and incubated in the presence of specific horseradish-peroxidase conjugated IgG. Immunoreactive bands were identified using the ECL detection system (Amersham International, Buckinghamshire, UK).

### Isolation of EVs and cellular RNA

Total RNA was extracted from EVs prepared as described above using Fatty Tissue RNA Purification Kit (Norgen, Thorold, Canada) following the manufacturer’s protocol. For each cell line, two independent biological replicates were used for RNA preparation. Quality, yield and size of the EV RNA were analyzed using capillary electrophoresis, with both Agilent RNA 6000 Pico Kit and Agilent Small RNA Kit on an Agilent 2100 Bioanalyzer (Agilent Technologies, Santa Clara, CA, USA). Supplementary Figure 3 shows that total RNA contained in EVs derived from all the 4 cell lines spans a size range from about 25 up to 4000 nt, with a peak between 25 and 250 nt. When the small RNAs range was analyzed in more detail (Supplementary Figure 4), the amount of RNA in the miRNA region was estimated about 20-30% of the sample.

Total RNA was extracted from cultured LCC cells lines using the Fatty Tissue RNA Purification Kit (Norgen, Thorold, Canada) following the manufacturer’s protocol. RNA quality was checked by analysis on Agilent Bioanalyzer using the Agilent RNA 6000 Nano Kit.

### SOLiD library construction and sequencing

Library preparation, sequencing and bioinformatic analysis were done by the service provider GENOMNIA srl (Bresso, Milano). For each RNA sample, two different libraries were generated, one to analyze small RNA and the other larger RNA. Small RNA libraries were prepared directly from total RNA samples without any RNA selection using the small RNA protocol of the SOLiD® Total RNA-Seq Kit, consisting in directional SOLiD adapter ligation, retrotranscription and barcoding through PCR amplification. The libraries were quantified with the Bioanalyzer (see Supplementary Figure 5) and then pooled and size selected by gel electrophoresis, to eliminate the adapter band. Templated bead preparation and sequencing was performed according the standard SOLiD 5500 XL workflow. At least 25 M of tags for each library, 35 bp long, were obtained.

WTA libraries were prepared starting from the same total RNA samples using the WTA protocol of the SOLiD® Total RNA-Seq Kit. The RNA was first fragmented by mild alkaline hydrolysis, then after ligation of the directional SOLiD adapter, was retrotranscribed and barcoded through PCR amplification. The libraries were quantified with the Bioanalyzer (see Supplementary Figure 6), then pooled and used for the preparation of the templated beads. Sequencing was performed with the 5500XL platform and about 25 M tags, 50 bp long, were obtained for each library.

### Transcriptome analyses

To obtain absolute quantification of gene expression, the colorspace sequencing files produced by sequencing the WTA libraries were mapped against human genome GRCh38/hg38 using Lifescope software (ver. 2.5.1) in single-ended mode. Each Ensembl Gene ID (release 81) was then assigned an expression value representing the number of mapped sequences. These counts were then normalized and used to perform differential analysis using the R EdgeR package (version 3.2.1) and Genomnia analytical parameters. Then to each Ensembl Gene ID a Gene Name and Gene type have been associated by using Ensembl BiomaRt software. The Gene Type was used to identify the different categories of genes quantified in this study. To determine the miRNA expression profiles, the data files obtained by sequencing the small RNA libraries were analyzed by the Lifescope software using the “small RNA” pipeline and mapped against the human genome GRCh38/hg38 and the miRBase (version 21) dataset.

### Real-time RT-PCR analysis

For the large transcript analysis total RNA was reverse-transcribed with the High-Capacity cDNA Reverse Transcription Kit (Applied Biosystems) according to the manufacturer’s instructions, using a maximum of 100 ng of total RNA for each 10 μL of reaction final volume. cDNA aliquots equivalent to 1 ng of RNA were then subjected to real time PCR analysis with an Applied Biosystems 7900HT thermal cycler using the TaqMan® Gene Expression Master Mix (Applied Biosystems) and the TaqMan® Gene Expression Assays (Applied Biosystems) for the following genes: VTRNA1-1, H19, EEF2, RACK1 (GNB2L1), SNHG12, SNHG1. The experiments were performed using biological duplicates and each measurement was performed in triplicate.

For the analysis of miRNA expression, 10 ng of total RNA was reverse-transcribed with the TaqMan Advanced miRNA cDNA Synthesis Kit (Applied Biosystems) according to the manufacturer’s instructions. Equal amounts of cDNA (corresponding to 10 pg of RNA) were then subjected to real time PCR analysis with an Applied Biosystems 7900HT thermal cycler using the Fast Advanced Master Mix (Applied Biosystems) and the TaqMan® Advanced miRNA Assays for the following microRNAs: hsa-miR-92a-3p, hsa-miR-93-5p, hsa-miR-17-5p, hsa-miR-451a, hsa-miR-21-5p, hsa-miR-373. The experiments were performed using biological duplicates and each measurement was performed in triplicate.

The data were analyzed by the Applied Biosystems SDS software v4.0 and the deltaCt were plotted and used to compare specific RNA and miRNA abundance.

## SUPPLEMENTARY MATERIALS FIGURES AND TABLES














