# Supplementary material for: RNA-seq reveals distinctive RNA profiles of small extracellular vesicles from different human liver cancer cell lines
Source: Oncotarget. 2017 Aug 24;8(47):82920–39. doi: 10.18632/oncotarget.20503 (PMC5669939; doi:10.18632/oncotarget.20503)
Supplement: Supplementary file 1 [file oncotarget-08-82920-s001.pdf]

RNA-seq reveals distinctive RNA profiles of small extracellular vesicles from different human liver cancer cell lines

SUPPLEMENTARY MATERIALS

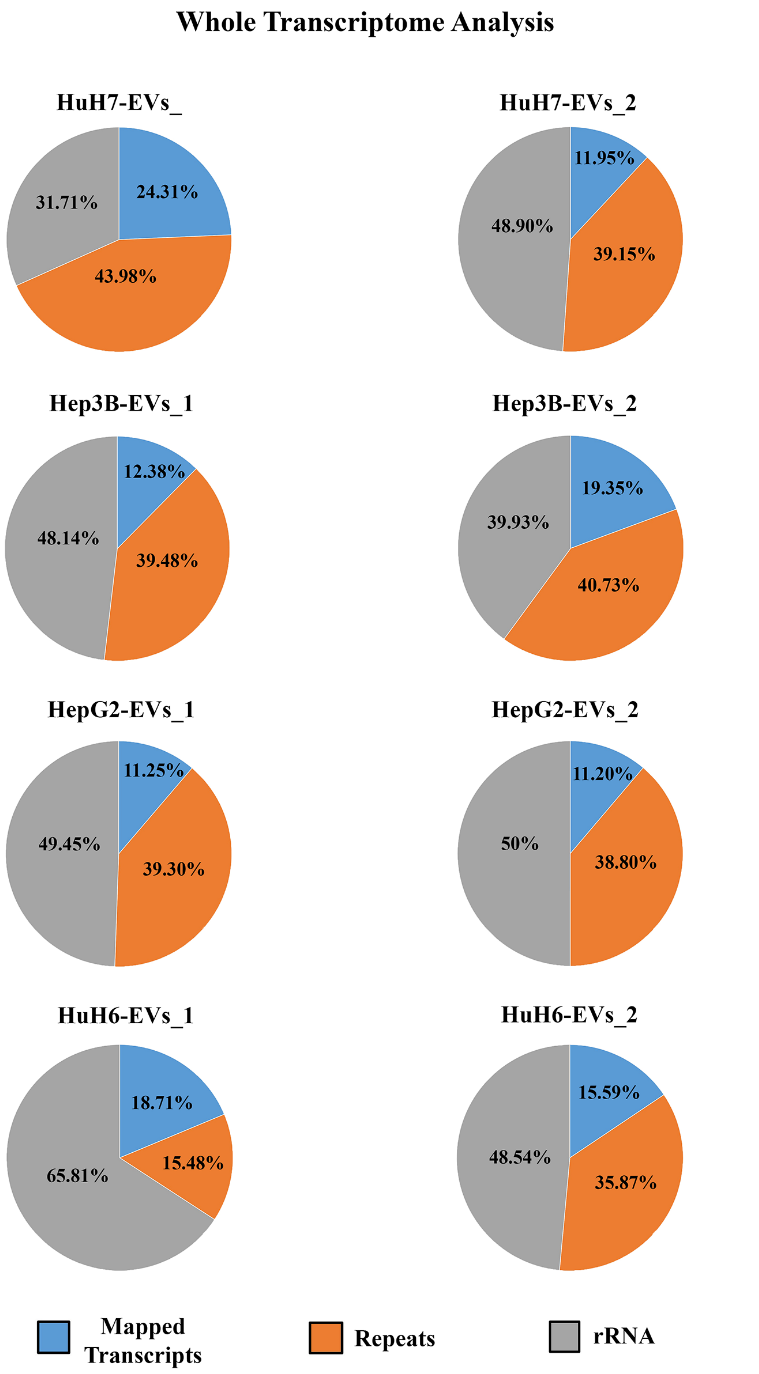

Supplementary Figure 1: WTA library mapping statistics on the reference genome (hg38). The WTA libraries, two duplicates for each LCC analyzed, have been used to identify coding and non-coding RNAs and snoRNAs**.**

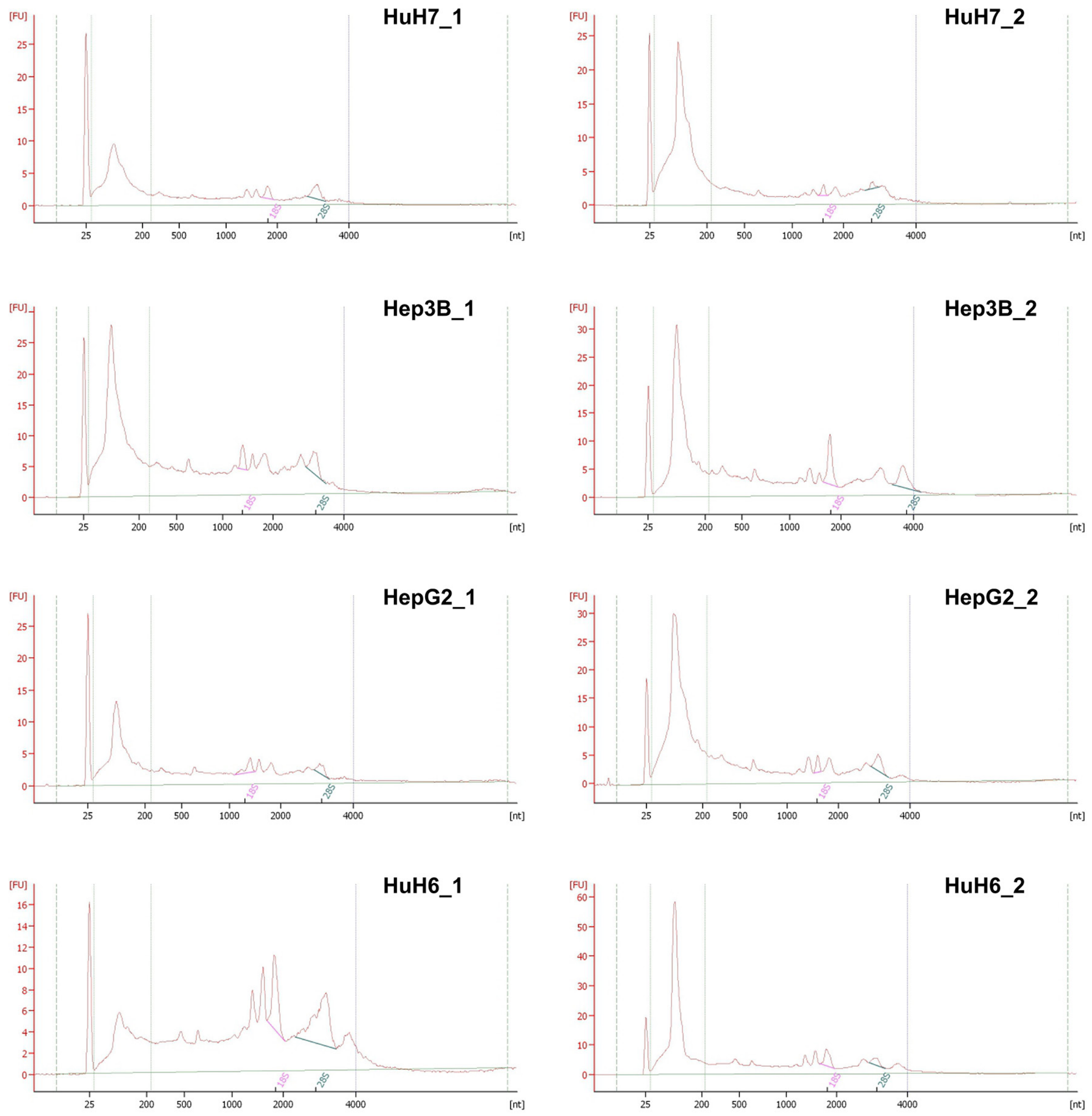

Supplementary Figure 2: Size distribution of total RNA molecules contained in extracellular vesicles secreted from the 4 LCC. Two duplicates for each LCC are shown.

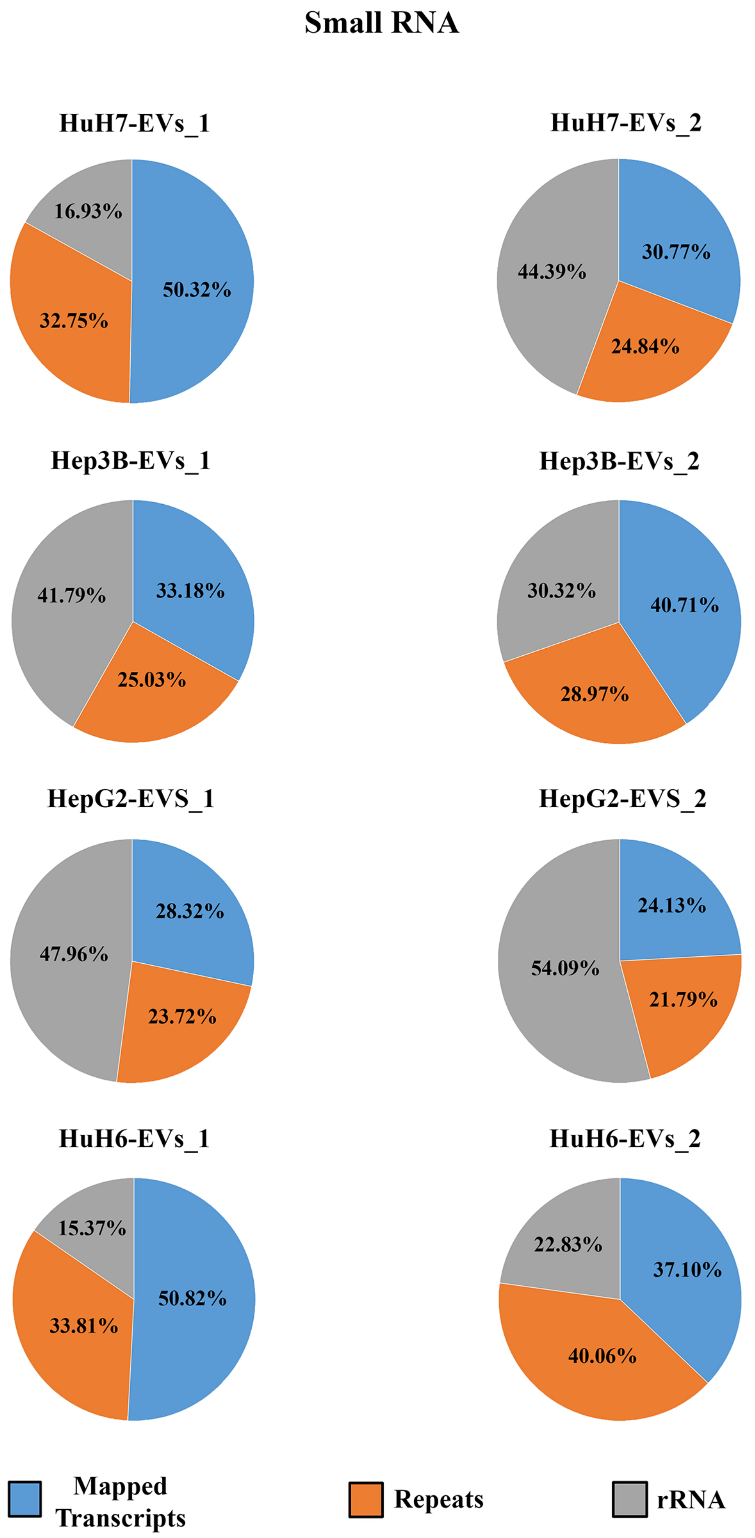

Supplementary Figure 3: Small RNA library mapping statistics on the reference genome (hg38). Small RNA libraries, two duplicates for each LCC analyzed, have been used to identify microRNAs and snoRNAs.

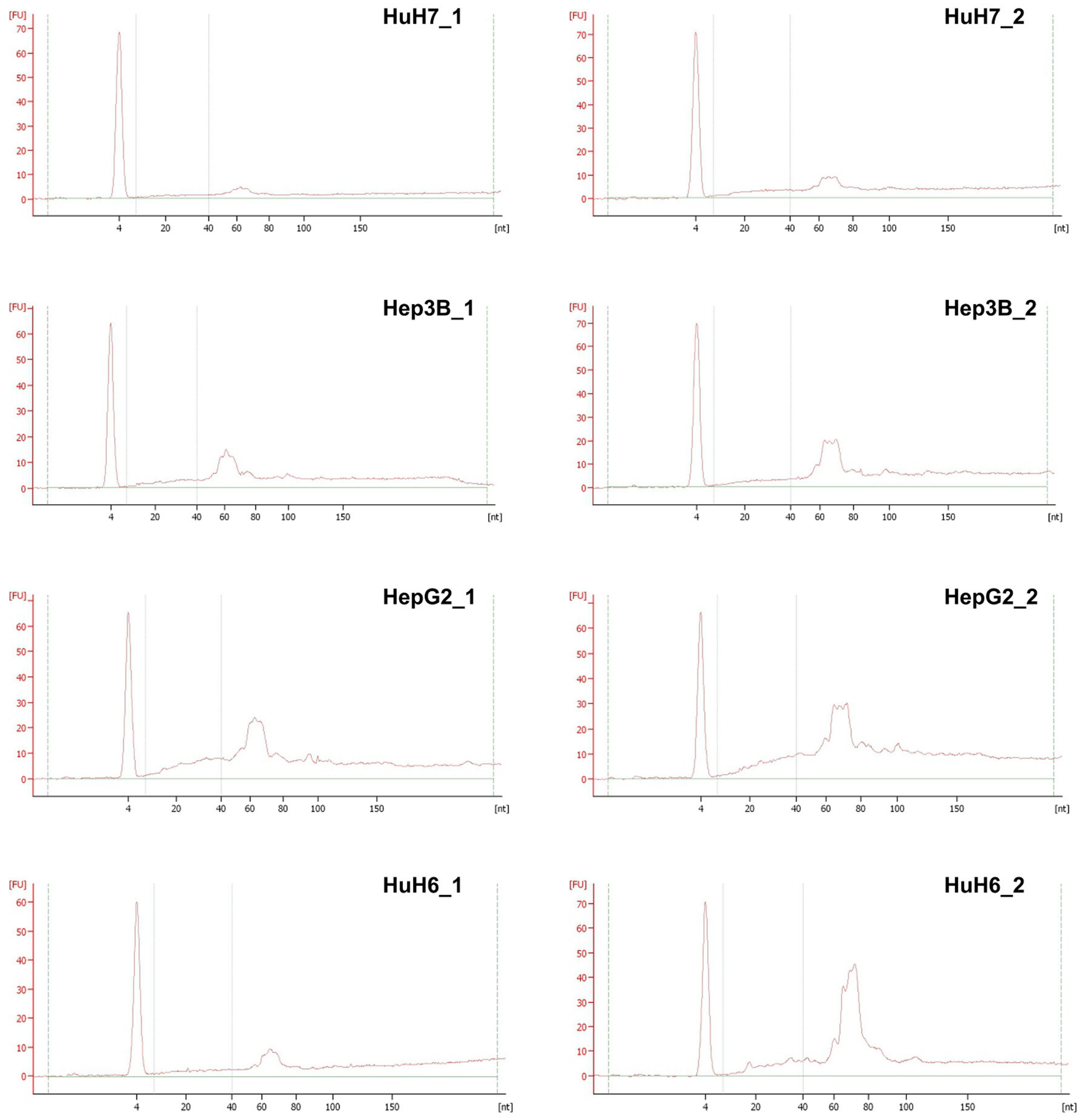

Supplementary Figure 4: Size distribution of small RNA molecules contained in extracellular vesicles secreted from the 4 LCC. Two duplicates for each LCC are shown, as indicated.

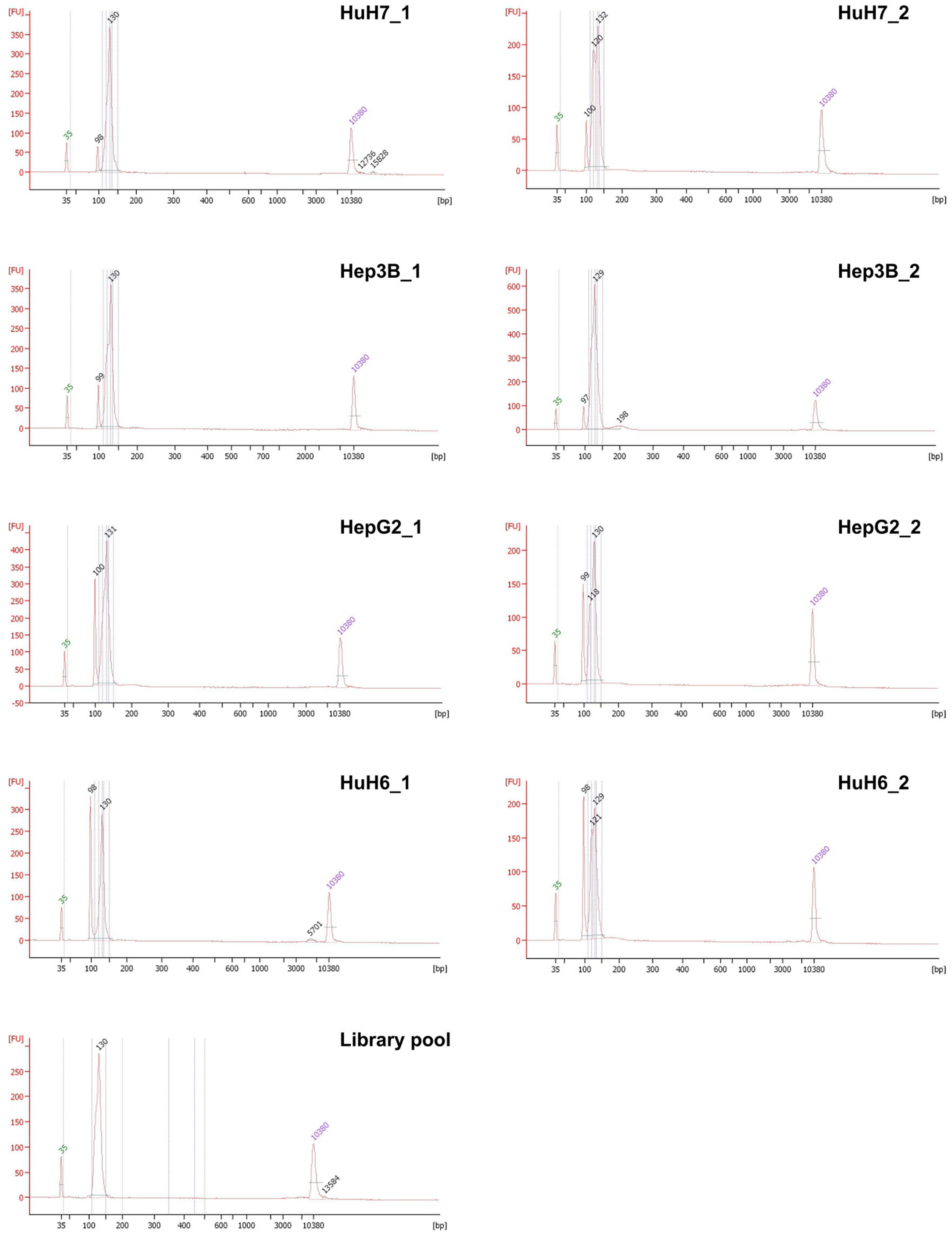

Supplementary Figure 5: Size distribution of the small RNA libraries prepared from the total RNA contained in extracellular vesicles secreted from the 4 LCC. Two duplicates for each LCC are shown, as indicated. 99-100 bp peak: "devil band", formed by the excess adapter dimers; 118-130 bp peaks: bands containing the library inserts plus the adapters. Library pool: the 8 small RNA barcoded libraries were pooled and purified by gel electrophoresis to eliminate the devil band. The peaks at 35 bp and 10380 bp are size markers.

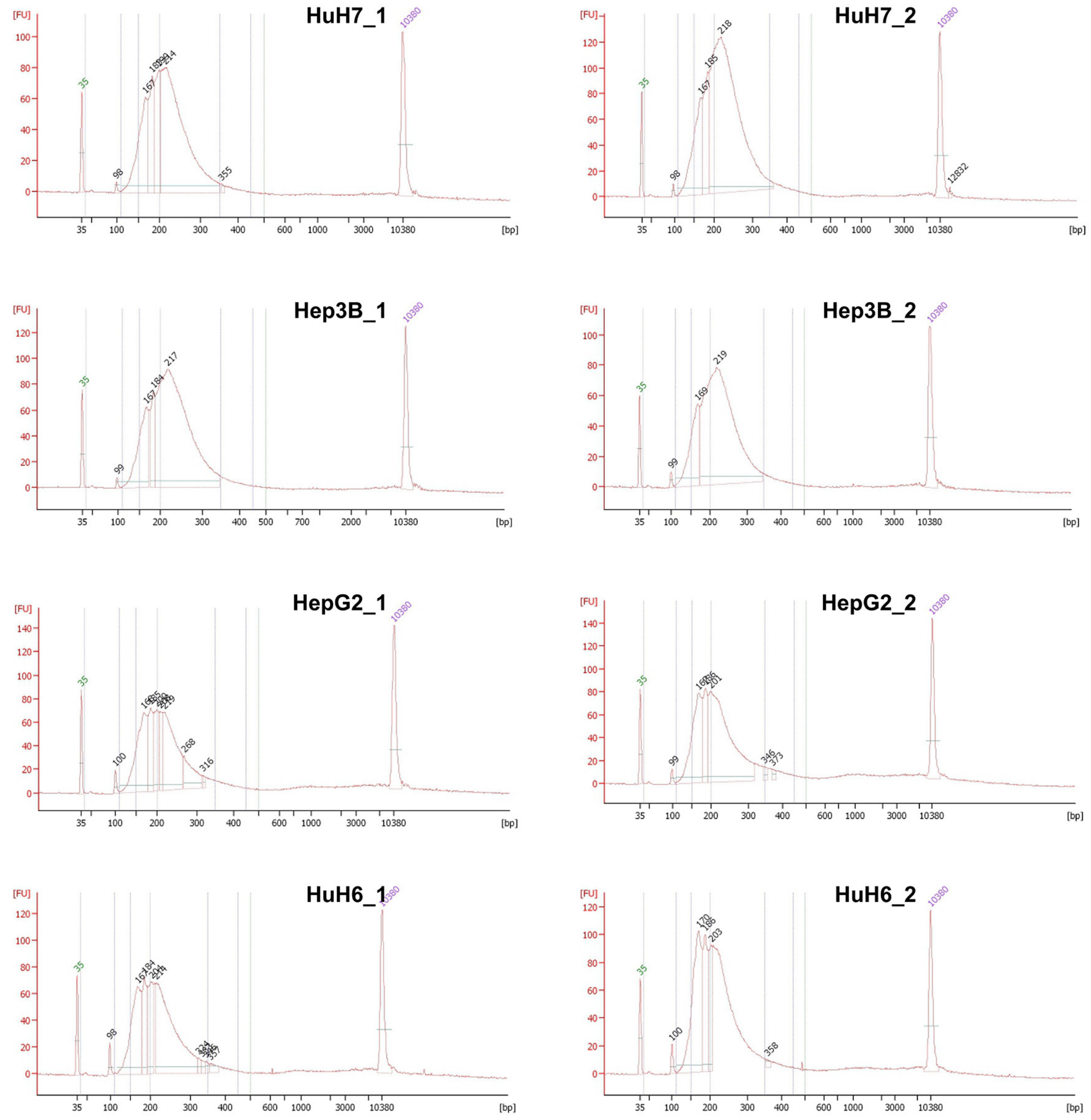

Supplementary Figure 6: Size distribution of the WTA libraries prepared from the total RNA contained in extracellular vesicles secreted from the 4 LCC. Two duplicates for each LCC are shown, as indicated. 99-100 bp peak: “devil band”, formed by the excess adapter dimers; 150-300 bp range: region containing the library inserts plus the adapters. The peaks at 35 bp and 10380 bp are size markers.

Supplementary Table 1: Gene transcripts

See Supplementary File 1

Supplementary Table 2: miRNAs CPM

See Supplementary File 2

Supplementary Table 3A: isomiR CPM

See Supplementary File 3A

Supplementary Table 3B: 10 most expressed isomiRs

See Supplementary File 3B

Supplementary Table 4: miRNA-isomiR fraction

Small RNA library	isomiR reads	Canonical miRNA reads	Total reads	miRNA fraction	isomiR fraction
**HuH7-EVs_1**	349646	151404	501050	0,70	0,30
**HuH7-EVs_2**	73628	25826	99454	0,74	0,26
**Hep3B-EVs_1**	252180	110761	362941	0,69	0,31
**Hep3B-EVs_2**	644988	229879	874867	0,74	0,26
**HepG2-EVs_1**	273205	101597	374802	0,73	0,27
**HepG2-EVs_2**	428603	144845	573448	0,75	0,25
**HuH6-EVs_1**	796749	313573	1110322	0,72	0,28
**HuH6-EVs_2**	1761404	697756	2459160	0,72	0,28

Supplementary Table 5: snoRNAs

See Supplementary File 4

Supplementary Table 6: snoRNA

See Supplementary File 5
