# Supplementary material for: RNA-seq reveals distinctive RNA profiles of small extracellular vesicles from different human liver cancer cell lines
Source: Oncotarget. 2017 Aug 24;8(47):82920–39. doi: 10.18632/oncotarget.20503 (PMC5669939; doi:10.18632/oncotarget.20503)
Supplement: Supplementary file 2 [file oncotarget-08-82920-s002.docx]

**Table S1. Gene Transcripts**

| **Ensembl Gene ID** | **Gene Symbol** | **Transcript type** | **HuH7-EVs_1** | **HuH7-EVs_2** | **Hep3B-EVs_1** | **Hep3b-EVs_2** | **HepG-EVs_1** | **HepG2-EVs_2** | **HuH6-EVs_1** | **HuH6-EVs_2** |
| --- | --- | --- | --- | --- | --- | --- | --- | --- | --- | --- |
| **ENSG00000269900** | RMRP | lincRNA | 42518 | 31217 | 33459 | 22677 | 32156 | 34952 | 19186 | 26950 |
| **ENSG00000277027** | RMRP | ribozyme | 42518 | 31217 | 33459 | 22677 | 32156 | 34952 | 19186 | 26950 |
| **ENSG00000129250** | KIF1C | protein_coding | 5979 | 5639 | 2557 | 8847 | 5242 | 4373 | 8903 | 10033 |
| **ENSG00000075624** | ACTB | processed_transcript | 1780 | 2354 | 2557 | 3323 | 1653 | 1657 | 8868 | 9442 |
| **ENSG00000259001** | RPPH1 | antisense | 22028 | 11594 | 27386 | 37759 | 33398 | 34262 | 6885 | 6740 |
| **ENSG00000277209** | RPPH1 | ribozyme | 22028 | 11594 | 27386 | 37759 | 33398 | 34262 | 6885 | 6740 |
| **ENSG00000115414** | FN1 | protein_coding | 1762 | 1686 | 1144 | 1751 | 2610 | 2539 | 5046 | 5245 |
| **ENSG00000071054** | MAP4K4 | protein_coding | 701 | 632 | 1766 | 3983 | 1242 | 932 | 3541 | 3927 |
| **ENSG00000184009** | ACTG1 | protein_coding | 1142 | 1107 | 1312 | 1351 | 2147 | 1685 | 3529 | 3387 |
| **ENSG00000111640** | GAPDH | protein_coding | 998 | 1019 | 521 | 891 | 905 | 782 | 3022 | 3133 |
| **ENSG00000204628** | RACK1 | nonsense_mediated_decay | 1457 | 1423 | 1346 | 1321 | 863 | 939 | 2878 | 2855 |
| **ENSG00000202111** | VTRNA1-2 | vaultRNA | 64006 | 9820 | 1194 | 450 | 30009 | 36267 | 2126 | 3497 |
| **ENSG00000074800** | ENO1 | protein_coding | 665 | 703 | 404 | 731 | 579 | 704 | 2944 | 2576 |
| **ENSG00000167658** | EEF2 | protein_coding | 3354 | 3917 | 2944 | 3242 | 2463 | 2318 | 2556 | 2610 |
| **ENSG00000034510** | TMSB10 | protein_coding | 935 | 913 | 824 | 1101 | 874 | 761 | 2544 | 2475 |
| **ENSG00000270123** | VTRNA2-1 | vaultRNA | 1286 | 1581 | 0 | 10 | 11 | 0 | 1768 | 3167 |
| **ENSG00000278815** | Vault | vaultRNA | 1286 | 1581 | 0 | 10 | 11 | 0 | 1768 | 3167 |
| **ENSG00000133112** | TPT1 | protein_coding | 1537 | 2758 | 1127 | 721 | 1210 | 981 | 2078 | 2187 |
| **ENSG00000070756** | PABPC1 | protein_coding | 342 | 404 | 252 | 390 | 358 | 391 | 2036 | 2120 |
| **ENSG00000171345** | KRT19 | protein_coding | 81 | 18 | 219 | 310 | 411 | 327 | 2036 | 2019 |
| **ENSG00000110092** | CCND1 | protein_coding | 279 | 228 | 84 | 90 | 568 | 434 | 2126 | 1841 |
| **ENSG00000199990** | VTRNA1-1 | vaultRNA | 37564 | 4743 | 10194 | 2712 | 105267 | 127540 | 1666 | 2272 |
| **ENSG00000087086** | FTL | protein_coding | 1663 | 1282 | 1548 | 1721 | 2863 | 2546 | 1869 | 1934 |
| **ENSG00000202515** | VTRNA1-3 | vaultRNA | 39830 | 4655 | 8276 | 1231 | 110804 | 124148 | 1320 | 2373 |
| **ENSG00000142676** | RPL11 | protein_coding | 315 | 527 | 269 | 440 | 474 | 327 | 1875 | 1706 |
| **ENSG00000198886** | MT-ND4 | protein_coding | 1232 | 2600 | 1514 | 1141 | 2547 | 1806 | 1499 | 1985 |
| **ENSG00000110321** | EIF4G2 | protein_coding | 1286 | 1722 | 1464 | 991 | 674 | 668 | 1797 | 1630 |
| **ENSG00000100345** | MYH9 | protein_coding | 2095 | 2319 | 404 | 210 | 1126 | 996 | 1971 | 1444 |
| **ENSG00000087460** | GNAS | protein_coding | 477 | 632 | 1077 | 1421 | 789 | 875 | 1738 | 1638 |
| **ENSG00000255717** | SNHG1 | lincRNA | 6384 | 5674 | 6392 | 7095 | 1684 | 1621 | 1576 | 1706 |
| **ENSG00000115306** | SPTBN1 | protein_coding | 3542 | 3390 | 2557 | 2512 | 1179 | 1124 | 1553 | 1664 |
| **ENSG00000170421** | KRT8 | protein_coding | 261 | 299 | 236 | 390 | 211 | 178 | 1696 | 1520 |
| **ENSG00000161016** | RPL8 | retained_intron | 620 | 509 | 336 | 530 | 484 | 334 | 1642 | 1554 |
| **ENSG00000115993** | TRAK2 | protein_coding | 2463 | 2442 | 303 | 1121 | 1863 | 1557 | 1445 | 1714 |
| **ENSG00000162592** | CCDC27 | retained_intron | 0 | 0 | 17 | 0 | 0 | 0 | 1284 | 1858 |
| **ENSG00000111057** | KRT18 | protein_coding | 450 | 264 | 84 | 180 | 253 | 213 | 1678 | 1436 |
| **ENSG00000078369** | GNB1 | protein_coding | 216 | 299 | 421 | 570 | 274 | 277 | 1469 | 1470 |
| **ENSG00000251562** | MALAT1 | lincRNA | 1178 | 949 | 2035 | 2002 | 821 | 668 | 1576 | 1284 |
| **ENSG00000109971** | HSPA8 | protein_coding | 512 | 668 | 589 | 741 | 716 | 604 | 1415 | 1444 |
| **ENSG00000067225** | PKM | retained_intron | 683 | 580 | 572 | 691 | 463 | 633 | 1606 | 1216 |
| **ENSG00000084207** | GSTP1 | protein_coding | 557 | 913 | 17 | 0 | 0 | 0 | 1308 | 1402 |
| **ENSG00000096696** | DSP | protein_coding | 297 | 422 | 1514 | 2132 | 2474 | 2055 | 1493 | 1191 |
| **ENSG00000108518** | PFN1 | protein_coding | 566 | 334 | 286 | 590 | 453 | 320 | 1200 | 1461 |
| **ENSG00000198786** | MT-ND5 | protein_coding | 791 | 2284 | 1127 | 761 | 1463 | 1444 | 967 | 1579 |
| **ENSG00000175061** | LRRC75A-AS1 | antisense | 548 | 615 | 320 | 560 | 211 | 171 | 1373 | 1140 |
| **ENSG00000115053** | NCL | protein_coding | 512 | 615 | 454 | 731 | 821 | 775 | 1194 | 1309 |
| **ENSG00000105372** | RPS19 | protein_coding | 396 | 457 | 320 | 560 | 526 | 505 | 1314 | 1157 |
| **ENSG00000196924** | FLNA | nonsense_mediated_decay | 683 | 1054 | 538 | 540 | 632 | 782 | 1242 | 1174 |
| **ENSG00000089157** | RPLP0 | protein_coding | 225 | 176 | 135 | 120 | 232 | 199 | 1164 | 1242 |
| **ENSG00000177600** | RPLP2 | protein_coding | 512 | 527 | 707 | 610 | 547 | 384 | 1081 | 1292 |
| **ENSG00000100316** | RPL3 | protein_coding | 1501 | 1581 | 858 | 1241 | 568 | 548 | 1093 | 1258 |
| **ENSG00000138867** | GUCD1 | protein_coding | 1304 | 6078 | 2490 | 2252 | 1368 | 1422 | 639 | 1664 |
| **ENSG00000197756** | RPL37A | protein_coding | 360 | 316 | 252 | 290 | 505 | 455 | 1087 | 1132 |
| **ENSG00000137154** | RPS6 | protein_coding | 297 | 211 | 151 | 300 | 337 | 320 | 1093 | 1056 |
| **ENSG00000200087** | SNORA73B | snoRNA | 4019 | 6043 | 3129 | 1941 | 821 | 1152 | 1009 | 1064 |
| **ENSG00000198727** | MT-CYB | protein_coding | 629 | 1247 | 1110 | 640 | 1347 | 1109 | 1069 | 997 |
| **ENSG00000197111** | PCBP2 | protein_coding | 189 | 211 | 303 | 510 | 263 | 277 | 1087 | 929 |
| **ENSG00000172757** | CFL1 | processed_transcript | 351 | 404 | 336 | 480 | 358 | 313 | 1117 | 878 |
| **ENSG00000128591** | FLNC | protein_coding | 90 | 141 | 151 | 20 | 11 | 0 | 1105 | 853 |
| **ENSG00000115677** | HDLBP | protein_coding | 692 | 755 | 858 | 981 | 1137 | 974 | 1039 | 912 |
| **ENSG00000197747** | S100A10 | protein_coding | 126 | 281 | 0 | 10 | 42 | 85 | 967 | 971 |
| **ENSG00000198888** | MT-ND1 | protein_coding | 351 | 1318 | 673 | 771 | 1421 | 946 | 794 | 1140 |
| **ENSG00000131469** | RPL27 | protein_coding | 198 | 141 | 185 | 240 | 368 | 334 | 908 | 1013 |
| **ENSG00000142534** | RPS11 | retained_intron | 890 | 878 | 488 | 721 | 600 | 434 | 902 | 1013 |
| **ENSG00000137818** | RPLP1 | protein_coding | 144 | 211 | 168 | 260 | 263 | 220 | 938 | 929 |
| **ENSG00000083857** | FAT1 | protein_coding | 153 | 141 | 269 | 681 | 526 | 597 | 717 | 1115 |
| **ENSG00000105193** | RPS16 | protein_coding | 432 | 281 | 168 | 350 | 379 | 377 | 991 | 836 |
| **ENSG00000143761** | ARF1 | protein_coding | 468 | 492 | 219 | 350 | 253 | 235 | 872 | 954 |
| **ENSG00000197989** | SNHG12 | retained_intron | 2266 | 3566 | 5215 | 1741 | 1210 | 1486 | 1087 | 726 |
| **ENSG00000156482** | RPL30 | processed_transcript | 252 | 105 | 303 | 340 | 242 | 270 | 908 | 870 |
| **ENSG00000009307** | CSDE1 | protein_coding | 548 | 492 | 404 | 450 | 474 | 441 | 794 | 980 |
| **ENSG00000149925** | ALDOA | protein_coding | 575 | 843 | 690 | 600 | 726 | 683 | 848 | 912 |
| **ENSG00000067560** | RHOA | protein_coding | 234 | 299 | 236 | 300 | 158 | 206 | 967 | 785 |
| **ENSG00000149273** | RPS3 | protein_coding | 198 | 176 | 135 | 240 | 453 | 277 | 878 | 861 |
| **ENSG00000159335** | PTMS | processed_transcript | 234 | 193 | 151 | 300 | 179 | 107 | 782 | 937 |
| **ENSG00000125691** | RPL23 | protein_coding | 980 | 1528 | 2809 | 1131 | 895 | 1045 | 842 | 870 |
| **ENSG00000142669** | SH3BGRL3 | protein_coding | 198 | 105 | 84 | 150 | 116 | 50 | 806 | 895 |
| **ENSG00000026025** | VIM | protein_coding | 971 | 1089 | 538 | 821 | 0 | 14 | 824 | 861 |
| **ENSG00000161960** | EIF4A1 | nonsense_mediated_decay | 2994 | 4357 | 4660 | 3272 | 1095 | 1052 | 866 | 819 |
| **ENSG00000142937** | RPS8 | processed_transcript | 701 | 509 | 437 | 440 | 368 | 341 | 902 | 777 |
| **ENSG00000161011** | SQSTM1 | processed_transcript | 512 | 492 | 437 | 300 | 400 | 327 | 693 | 954 |
| **ENSG00000075618** | FSCN1 | protein_coding | 81 | 0 | 303 | 200 | 147 | 178 | 836 | 811 |
| **ENSG00000182718** | ANXA2 | protein_coding | 189 | 70 | 151 | 70 | 32 | 28 | 800 | 836 |
| **ENSG00000163191** | S100A11 | protein_coding | 72 | 123 | 34 | 30 | 42 | 85 | 830 | 777 |
| **ENSG00000138326** | RPS24 | processed_transcript | 207 | 246 | 84 | 170 | 221 | 206 | 812 | 794 |
| **ENSG00000171858** | RPS21 | protein_coding | 135 | 123 | 236 | 160 | 253 | 242 | 794 | 802 |
| **ENSG00000128849** | CGNL1 | processed_transcript | 539 | 545 | 572 | 2012 | 158 | 121 | 645 | 946 |
| **ENSG00000234741** | GAS5 | lincRNA | 1987 | 2213 | 2977 | 3693 | 632 | 348 | 729 | 861 |
| **ENSG00000242265** | PEG10 | protein_coding | 566 | 492 | 101 | 40 | 1768 | 1906 | 752 | 836 |
| **ENSG00000198804** | MT-CO1 | protein_coding | 279 | 1089 | 841 | 390 | 1084 | 1060 | 651 | 937 |
| **ENSG00000158710** | TAGLN2 | protein_coding | 135 | 281 | 151 | 250 | 74 | 14 | 764 | 819 |
| **ENSG00000189334** | S100A14 | protein_coding | 0 | 0 | 118 | 260 | 0 | 0 | 740 | 828 |
| **ENSG00000112306** | RPS12 | protein_coding | 324 | 404 | 336 | 230 | 305 | 299 | 824 | 735 |
| **ENSG00000211679** | IGLC3 | IG_C_gene | 503 | 1159 | 656 | 320 | 589 | 910 | 382 | 1174 |
| **ENSG00000060138** | YBX3 | protein_coding | 126 | 88 | 50 | 40 | 105 | 57 | 908 | 642 |
| **ENSG00000145592** | RPL37 | protein_coding | 593 | 562 | 286 | 430 | 326 | 313 | 776 | 760 |
| **ENSG00000110955** | ATP5B | processed_transcript | 252 | 474 | 488 | 630 | 347 | 235 | 920 | 617 |
| **ENSG00000164587** | RPS14 | protein_coding | 144 | 193 | 17 | 50 | 168 | 220 | 717 | 819 |
| **ENSG00000152767** | FARP1 | protein_coding | 234 | 176 | 236 | 320 | 242 | 220 | 663 | 845 |
| **ENSG00000156976** | EIF4A2 | protein_coding | 4945 | 4532 | 8697 | 8346 | 1421 | 1067 | 854 | 650 |
| **ENSG00000257379** | RP11-793H13.8 | retained_intron | 144 | 211 | 185 | 420 | 200 | 192 | 752 | 726 |
| **ENSG00000198034** | RPS4X | processed_transcript | 333 | 281 | 135 | 150 | 263 | 228 | 729 | 743 |
| **ENSG00000169100** | SLC25A6 | protein_coding | 261 | 351 | 135 | 200 | 389 | 384 | 752 | 718 |
| **ENSG00000075415** | SLC25A3 | protein_coding | 450 | 509 | 454 | 420 | 411 | 213 | 764 | 693 |
| **ENSG00000196230** | TUBB | protein_coding | 405 | 193 | 791 | 1001 | 695 | 434 | 752 | 701 |
| **ENSG00000083845** | RPS5 | protein_coding | 405 | 211 | 353 | 400 | 368 | 235 | 752 | 693 |
| **ENSG00000116649** | SRM | retained_intron | 225 | 193 | 118 | 50 | 53 | 156 | 836 | 600 |
| **ENSG00000101361** | NOP56 | protein_coding | 764 | 720 | 471 | 941 | 589 | 405 | 860 | 574 |
| **ENSG00000011052** | NME1-NME2 | protein_coding | 351 | 386 | 219 | 310 | 242 | 256 | 645 | 785 |
| **ENSG00000143321** | HDGF | protein_coding | 279 | 228 | 404 | 480 | 200 | 171 | 794 | 633 |
| **ENSG00000171552** | BCL2L1 | protein_coding | 216 | 141 | 168 | 150 | 242 | 334 | 776 | 633 |
| **ENSG00000096384** | HSP90AB1 | protein_coding | 369 | 316 | 320 | 540 | 800 | 697 | 764 | 591 |
| **ENSG00000196141** | SPATS2L | protein_coding | 171 | 264 | 387 | 240 | 284 | 192 | 770 | 583 |
| **ENSG00000119314** | PTBP3 | protein_coding | 135 | 70 | 168 | 140 | 95 | 128 | 573 | 777 |
| **ENSG00000136938** | ANP32B | protein_coding | 360 | 228 | 168 | 540 | 463 | 491 | 555 | 785 |
| **ENSG00000114867** | EIF4G1 | protein_coding | 423 | 422 | 505 | 711 | 463 | 476 | 609 | 726 |
| **ENSG00000213626** | LBH | protein_coding | 0 | 0 | 0 | 10 | 0 | 0 | 699 | 625 |
| **ENSG00000221983** | UBA52 | protein_coding | 387 | 369 | 336 | 470 | 242 | 228 | 723 | 600 |
| **ENSG00000162909** | CAPN2 | protein_coding | 27 | 88 | 84 | 180 | 53 | 100 | 669 | 650 |
| **ENSG00000142599** | RERE | protein_coding | 144 | 88 | 118 | 290 | 158 | 213 | 621 | 684 |
| **ENSG00000171219** | CDC42BPG | protein_coding | 54 | 35 | 185 | 370 | 168 | 156 | 627 | 676 |
| **ENSG00000106263** | EIF3B | protein_coding | 171 | 193 | 622 | 520 | 263 | 156 | 663 | 625 |
| **ENSG00000148730** | EIF4EBP2 | protein_coding | 162 | 70 | 118 | 200 | 53 | 92 | 561 | 726 |
| **ENSG00000173812** | EIF1 | retained_intron | 180 | 141 | 236 | 250 | 253 | 220 | 639 | 642 |
| **ENSG00000142541** | RPL13A | protein_coding | 2832 | 3601 | 2355 | 2382 | 968 | 896 | 603 | 676 |
| **ENSG00000175166** | PSMD2 | protein_coding | 135 | 246 | 269 | 170 | 158 | 149 | 597 | 676 |
| **ENSG00000123562** | MORF4L2 | protein_coding | 153 | 88 | 135 | 90 | 74 | 100 | 699 | 566 |
| **ENSG00000163468** | CCT3 | protein_coding | 252 | 404 | 807 | 520 | 379 | 270 | 705 | 557 |
| **ENSG00000133816** | MICAL2 | protein_coding | 656 | 580 | 17 | 20 | 42 | 28 | 591 | 659 |
| **ENSG00000122566** | HNRNPA2B1 | processed_transcript | 468 | 404 | 807 | 881 | 389 | 284 | 615 | 633 |
| **ENSG00000067057** | PFKP | protein_coding | 0 | 0 | 0 | 20 | 11 | 21 | 723 | 524 |
| **ENSG00000188643** | S100A16 | protein_coding | 45 | 53 | 168 | 110 | 63 | 78 | 681 | 557 |
| **ENSG00000086758** | HUWE1 | protein_coding | 1133 | 861 | 723 | 1051 | 484 | 476 | 663 | 574 |
| **ENSG00000063177** | RPL18 | retained_intron | 306 | 369 | 185 | 230 | 253 | 320 | 657 | 566 |
| **ENSG00000117394** | SLC2A1 | protein_coding | 27 | 70 | 0 | 10 | 284 | 284 | 657 | 566 |
| **ENSG00000111206** | FOXM1 | retained_intron | 180 | 193 | 168 | 250 | 137 | 171 | 579 | 642 |
| **ENSG00000008988** | RPS20 | protein_coding | 135 | 193 | 168 | 150 | 126 | 114 | 681 | 515 |
| **ENSG00000092847** | AGO1 | protein_coding | 378 | 404 | 236 | 330 | 105 | 114 | 520 | 676 |
| **ENSG00000163466** | ARPC2 | protein_coding | 135 | 141 | 303 | 200 | 242 | 128 | 627 | 566 |
| **ENSG00000010404** | IDS | protein_coding | 18 | 88 | 84 | 0 | 21 | 57 | 555 | 633 |
| **ENSG00000155506** | LARP1 | protein_coding | 225 | 492 | 202 | 340 | 432 | 377 | 621 | 566 |
| **ENSG00000184216** | IRAK1 | protein_coding | 126 | 53 | 50 | 130 | 137 | 142 | 520 | 667 |
| **ENSG00000161203** | AP2M1 | protein_coding | 153 | 176 | 185 | 180 | 63 | 149 | 603 | 583 |
| **ENSG00000092841** | MYL6 | protein_coding | 234 | 246 | 454 | 370 | 232 | 149 | 627 | 557 |
| **ENSG00000118971** | CCND2 | protein_coding | 0 | 18 | 0 | 10 | 63 | 107 | 520 | 659 |
| **ENSG00000080824** | HSP90AA1 | protein_coding | 243 | 281 | 185 | 520 | 379 | 341 | 788 | 389 |
| **ENSG00000124208** | TMEM189-UBE2V1 | protein_coding | 54 | 88 | 84 | 90 | 137 | 57 | 609 | 566 |
| **ENSG00000186468** | RPS23 | protein_coding | 180 | 246 | 151 | 130 | 116 | 149 | 669 | 498 |
| **ENSG00000100097** | LGALS1 | protein_coding | 0 | 18 | 17 | 70 | 168 | 164 | 466 | 701 |
| **ENSG00000142798** | HSPG2 | retained_intron | 450 | 580 | 589 | 570 | 147 | 164 | 603 | 557 |
| **ENSG00000124942** | AHNAK | protein_coding | 333 | 299 | 303 | 240 | 116 | 114 | 585 | 574 |
| **ENSG00000115484** | CCT4 | protein_coding | 279 | 246 | 303 | 260 | 179 | 213 | 627 | 532 |
| **ENSG00000122406** | RPL5 | protein_coding | 324 | 299 | 84 | 150 | 295 | 78 | 573 | 583 |
| **ENSG00000100852** | ARHGAP5 | protein_coding | 1043 | 1212 | 135 | 100 | 347 | 348 | 567 | 583 |
| **ENSG00000173848** | NET1 | protein_coding | 926 | 913 | 269 | 991 | 1389 | 1415 | 531 | 617 |
| **ENSG00000101444** | AHCY | protein_coding | 207 | 176 | 219 | 240 | 368 | 256 | 555 | 591 |
| **ENSG00000136942** | RPL35 | protein_coding | 162 | 88 | 84 | 100 | 137 | 92 | 675 | 465 |
| **ENSG00000110700** | RPS13 | retained_intron | 171 | 264 | 135 | 110 | 137 | 156 | 555 | 583 |
| **ENSG00000153187** | HNRNPU | processed_transcript | 521 | 369 | 538 | 831 | 305 | 256 | 717 | 414 |
| **ENSG00000148677** | ANKRD1 | protein_coding | 279 | 246 | 219 | 10 | 84 | 92 | 699 | 431 |
| **ENSG00000172809** | RPL38 | protein_coding | 162 | 158 | 185 | 240 | 337 | 242 | 681 | 448 |
| **ENSG00000108107** | RPL28 | protein_coding | 306 | 386 | 135 | 250 | 253 | 306 | 585 | 541 |
| **ENSG00000197157** | SND1 | protein_coding | 279 | 369 | 185 | 270 | 284 | 178 | 531 | 583 |
| **ENSG00000174444** | RPL4 | protein_coding | 620 | 597 | 454 | 490 | 263 | 270 | 573 | 541 |
| **ENSG00000169564** | PCBP1 | protein_coding | 306 | 228 | 219 | 350 | 326 | 235 | 555 | 557 |
| **ENSG00000182899** | RPL35A | protein_coding | 135 | 264 | 118 | 320 | 179 | 128 | 555 | 549 |
| **ENSG00000276788** | SNORD26 | snoRNA | 2194 | 2266 | 1867 | 1781 | 579 | 519 | 567 | 515 |
| **ENSG00000242125** | SNHG3 | retained_intron | 2634 | 3355 | 2607 | 1131 | 505 | 548 | 699 | 380 |
| **ENSG00000078804** | TP53INP2 | protein_coding | 216 | 193 | 135 | 961 | 747 | 661 | 460 | 617 |
| **ENSG00000108298** | RPL19 | protein_coding | 117 | 158 | 219 | 190 | 316 | 185 | 609 | 465 |
| **ENSG00000182774** | RPS17 | protein_coding | 180 | 264 | 168 | 200 | 211 | 199 | 573 | 498 |
| **ENSG00000108953** | YWHAE | protein_coding | 288 | 228 | 101 | 240 | 200 | 156 | 597 | 465 |
| **ENSG00000166012** | TAF1D | nonsense_mediated_decay | 1726 | 2213 | 2338 | 1771 | 800 | 732 | 496 | 557 |
| **ENSG00000240972** | MIF | protein_coding | 63 | 141 | 168 | 430 | 242 | 171 | 514 | 532 |
| **ENSG00000155366** | RHOC | protein_coding | 99 | 158 | 84 | 80 | 200 | 128 | 520 | 524 |
| **ENSG00000163399** | ATP1A1 | protein_coding | 342 | 369 | 303 | 240 | 347 | 199 | 543 | 498 |
| **ENSG00000213639** | PPP1CB | protein_coding | 72 | 158 | 84 | 60 | 147 | 149 | 543 | 498 |
| **ENSG00000159217** | IGF2BP1 | protein_coding | 252 | 264 | 303 | 340 | 421 | 391 | 585 | 456 |
| **ENSG00000108829** | LRRC59 | protein_coding | 216 | 316 | 151 | 140 | 263 | 171 | 585 | 456 |
| **ENSG00000198431** | TXNRD1 | protein_coding | 171 | 88 | 151 | 110 | 158 | 320 | 490 | 549 |
| **ENSG00000071127** | WDR1 | protein_coding | 279 | 351 | 151 | 350 | 211 | 284 | 430 | 608 |
| **ENSG00000127022** | CANX | protein_coding | 252 | 299 | 269 | 450 | 158 | 199 | 615 | 422 |
| **ENSG00000244687** | UBE2V1 | protein_coding | 27 | 53 | 50 | 50 | 126 | 57 | 525 | 507 |
| **ENSG00000197694** | SPTAN1 | protein_coding | 890 | 913 | 639 | 881 | 242 | 171 | 549 | 481 |
| **ENSG00000277194** | SNORD22 | snoRNA | 2356 | 1599 | 2237 | 2732 | 621 | 583 | 490 | 541 |
| **ENSG00000101182** | PSMA7 | protein_coding | 99 | 141 | 67 | 80 | 189 | 164 | 525 | 498 |
| **ENSG00000168036** | CTNNB1 | protein_coding | 351 | 492 | 370 | 430 | 347 | 284 | 454 | 566 |
| **ENSG00000262497** | FAM187B2P | unprocessed_pseudogene | 854 | 949 | 1548 | 490 | 1232 | 882 | 36 | 980 |
| **ENSG00000119318** | RAD23B | protein_coding | 297 | 439 | 269 | 210 | 179 | 192 | 466 | 549 |
| **ENSG00000105379** | ETFB | protein_coding | 144 | 88 | 101 | 150 | 295 | 135 | 508 | 507 |
| **ENSG00000169714** | CNBP | protein_coding | 144 | 123 | 135 | 210 | 74 | 149 | 597 | 414 |
| **ENSG00000198286** | CARD11 | protein_coding | 0 | 0 | 0 | 10 | 0 | 0 | 520 | 490 |
| **ENSG00000136068** | FLNB | protein_coding | 378 | 527 | 471 | 520 | 642 | 384 | 484 | 524 |
| **ENSG00000185963** | BICD2 | protein_coding | 117 | 18 | 84 | 180 | 168 | 206 | 430 | 574 |
| **ENSG00000166913** | YWHAB | protein_coding | 81 | 105 | 168 | 200 | 232 | 213 | 412 | 591 |
| **ENSG00000169710** | FASN | protein_coding | 773 | 685 | 673 | 971 | 789 | 882 | 520 | 481 |
| **ENSG00000264772** | RP11-186B7.4 | processed_transcript | 351 | 474 | 421 | 580 | 232 | 171 | 460 | 532 |
| **ENSG00000107581** | EIF3A | protein_coding | 432 | 369 | 505 | 671 | 326 | 277 | 466 | 515 |
| **ENSG00000184640** | SEPT9 | protein_coding | 234 | 422 | 572 | 540 | 747 | 732 | 525 | 439 |
| **ENSG00000198938** | MT-CO3 | protein_coding | 288 | 896 | 471 | 580 | 716 | 647 | 370 | 583 |
| **ENSG00000084674** | APOB | protein_coding | 216 | 246 | 1144 | 2332 | 1295 | 1173 | 508 | 439 |
| **ENSG00000130402** | ACTN4 | retained_intron | 773 | 861 | 320 | 620 | 347 | 313 | 555 | 389 |
| **ENSG00000069275** | NUCKS1 | protein_coding | 153 | 105 | 707 | 480 | 95 | 149 | 484 | 456 |
| **ENSG00000178209** | PLEC | protein_coding | 441 | 492 | 572 | 440 | 305 | 149 | 525 | 414 |
| **ENSG00000127603** | MACF1 | protein_coding | 521 | 597 | 707 | 811 | 421 | 228 | 448 | 490 |
| **ENSG00000274266** | SNORA73A | snoRNA | 2607 | 3355 | 2607 | 1121 | 495 | 548 | 579 | 355 |
| **ENSG00000122786** | CALD1 | protein_coding | 611 | 668 | 202 | 130 | 242 | 228 | 525 | 405 |
| **ENSG00000117523** | PRRC2C | processed_transcript | 198 | 246 | 740 | 1051 | 316 | 277 | 424 | 456 |
| **ENSG00000166441** | RPL27A | protein_coding | 2239 | 3408 | 3970 | 1111 | 537 | 540 | 490 | 389 |
| **ENSG00000165280** | VCP | protein_coding | 441 | 562 | 286 | 230 | 305 | 171 | 472 | 405 |
| **ENSG00000185624** | P4HB | protein_coding | 548 | 668 | 622 | 841 | 389 | 398 | 436 | 405 |
| **ENSG00000107957** | SH3PXD2A | protein_coding | 369 | 351 | 555 | 1911 | 695 | 505 | 316 | 515 |
| **ENSG00000116133** | DHCR24 | protein_coding | 288 | 457 | 320 | 801 | 179 | 100 | 316 | 507 |
| **ENSG00000047849** | MAP4 | protein_coding | 486 | 334 | 320 | 691 | 274 | 277 | 430 | 372 |
| **ENSG00000158874** | APOA2 | retained_intron | 36 | 141 | 1363 | 3393 | 821 | 562 | 400 | 397 |
| **ENSG00000153179** | RASSF3 | protein_coding | 1528 | 1511 | 656 | 2142 | 695 | 576 | 346 | 448 |
| **ENSG00000111642** | CHD4 | protein_coding | 324 | 457 | 387 | 711 | 263 | 235 | 406 | 372 |
| **ENSG00000212907** | MT-ND4L | protein_coding | 171 | 615 | 387 | 330 | 558 | 540 | 388 | 380 |
| **ENSG00000179218** | CALR | retained_intron | 459 | 791 | 404 | 650 | 211 | 306 | 370 | 397 |
| **ENSG00000200320** | SNORA63 | snoRNA | 2625 | 2670 | 5366 | 4003 | 747 | 626 | 436 | 329 |
| **ENSG00000087365** | SF3B2 | processed_transcript | 270 | 176 | 690 | 711 | 232 | 327 | 472 | 279 |
| **ENSG00000210082** | MT-RNR2 | Mt_rRNA | 279 | 808 | 421 | 490 | 495 | 377 | 293 | 448 |
| **ENSG00000138434** | SSFA2 | retained_intron | 90 | 1054 | 320 | 260 | 105 | 57 | 299 | 439 |
| **ENSG00000135100** | HNF1A | protein_coding | 746 | 2143 | 236 | 650 | 947 | 1017 | 197 | 541 |
| **ENSG00000274582** | SNORA16A | snoRNA | 800 | 1388 | 2439 | 530 | 326 | 441 | 484 | 253 |
| **ENSG00000280498** | SNORA16A | snoRNA | 800 | 1388 | 2439 | 530 | 326 | 441 | 484 | 253 |
| **ENSG00000174231** | PRPF8 | protein_coding | 369 | 422 | 639 | 520 | 421 | 299 | 418 | 312 |
| **ENSG00000198899** | MT-ATP6 | protein_coding | 180 | 703 | 454 | 260 | 505 | 526 | 346 | 380 |
| **ENSG00000154380** | ENAH | protein_coding | 539 | 509 | 555 | 1131 | 189 | 128 | 364 | 312 |
| **ENSG00000196576** | PLXNB2 | retained_intron | 629 | 615 | 303 | 310 | 232 | 199 | 305 | 372 |
| **ENSG00000167526** | RPL13 | protein_coding | 836 | 843 | 454 | 240 | 263 | 348 | 334 | 321 |
| **ENSG00000167468** | GPX4 | protein_coding | 198 | 193 | 454 | 600 | 232 | 156 | 311 | 338 |
| **ENSG00000060237** | WNK1 | protein_coding | 315 | 316 | 656 | 991 | 337 | 220 | 322 | 321 |
| **ENSG00000143569** | UBAP2L | protein_coding | 441 | 369 | 1110 | 650 | 295 | 220 | 328 | 304 |
| **ENSG00000209582** | SNORA48 | snoRNA | 2526 | 3707 | 4155 | 2542 | 832 | 839 | 376 | 253 |
| **ENSG00000167978** | SRRM2 | protein_coding | 656 | 457 | 656 | 400 | 484 | 548 | 358 | 270 |
| **ENSG00000197249** | SERPINA1 | protein_coding | 90 | 158 | 589 | 1751 | 1474 | 1102 | 287 | 338 |
| **ENSG00000130600** | H19 | lincRNA | 36 | 35 | 4273 | 7426 | 0 | 7 | 334 | 287 |
| **ENSG00000221500** | SNORD100 | snoRNA | 1394 | 2003 | 1649 | 1871 | 853 | 697 | 316 | 304 |
| **ENSG00000233016** | SNHG7 | antisense | 1070 | 1230 | 841 | 420 | 337 | 228 | 322 | 279 |
| **ENSG00000101745** | ANKRD12 | protein_coding | 818 | 1739 | 1749 | 470 | 463 | 533 | 287 | 304 |
| **ENSG00000112245** | PTP4A1 | processed_transcript | 468 | 562 | 454 | 420 | 442 | 348 | 322 | 262 |
| **ENSG00000151914** | DST | protein_coding | 459 | 755 | 1194 | 1161 | 621 | 661 | 245 | 304 |
| **ENSG00000197102** | DYNC1H1 | protein_coding | 539 | 474 | 437 | 811 | 263 | 370 | 328 | 220 |
| **ENSG00000273544** | SNORA44 | snoRNA | 998 | 1792 | 1968 | 741 | 653 | 775 | 352 | 177 |
| **ENSG00000278274** | SNORA61 | snoRNA | 441 | 369 | 774 | 460 | 232 | 249 | 239 | 287 |
| **ENSG00000168028** | RPSA | protein_coding | 1007 | 1142 | 690 | 671 | 147 | 142 | 269 | 245 |
| **ENSG00000253729** | PRKDC | protein_coding | 360 | 422 | 505 | 841 | 189 | 213 | 269 | 228 |
| **ENSG00000202031** | SNORD38A | snoRNA | 602 | 457 | 404 | 200 | 179 | 206 | 251 | 236 |
| **ENSG00000264549** | SNORD95 | snoRNA | 486 | 404 | 538 | 490 | 221 | 64 | 251 | 236 |
| **ENSG00000136205** | TNS3 | protein_coding | 279 | 281 | 538 | 921 | 168 | 206 | 215 | 270 |
| **ENSG00000175216** | CKAP5 | protein_coding | 162 | 211 | 387 | 640 | 137 | 135 | 257 | 203 |
| **ENSG00000141367** | CLTC | protein_coding | 450 | 773 | 589 | 490 | 537 | 420 | 215 | 228 |
| **ENSG00000224078** | SNHG14 | retained_intron | 9 | 35 | 454 | 640 | 105 | 50 | 149 | 279 |
| **ENSG00000270647** | TAF15 | retained_intron | 72 | 105 | 202 | 871 | 179 | 114 | 155 | 270 |
| **ENSG00000155657** | TTN | protein_coding | 432 | 597 | 303 | 260 | 147 | 199 | 185 | 220 |
| **ENSG00000142002** | DPP9 | protein_coding | 548 | 492 | 622 | 711 | 221 | 228 | 185 | 203 |
| **ENSG00000047410** | TPR | protein_coding | 180 | 193 | 387 | 941 | 147 | 199 | 173 | 203 |
| **ENSG00000275996** | SNORD27 | snoRNA | 432 | 334 | 589 | 580 | 74 | 128 | 161 | 203 |
| **ENSG00000201302** | SNORA65 | snoRNA | 926 | 1581 | 1346 | 420 | 316 | 363 | 185 | 177 |
| **ENSG00000147403** | RPL10 | retained_intron | 611 | 685 | 589 | 280 | 242 | 277 | 167 | 194 |
| **ENSG00000199631** | SNORD33 | snoRNA | 1214 | 1089 | 807 | 821 | 400 | 292 | 173 | 186 |
| **ENSG00000199293** | SNORA21 | snoRNA | 728 | 1423 | 2624 | 881 | 610 | 676 | 221 | 135 |
| **ENSG00000198563** | DDX39B | retained_intron | 414 | 562 | 774 | 470 | 168 | 235 | 167 | 186 |
| **ENSG00000130396** | AFDN | protein_coding | 306 | 457 | 690 | 440 | 316 | 220 | 161 | 186 |
| **ENSG00000120885** | CLU | protein_coding | 414 | 527 | 320 | 851 | 74 | 57 | 269 | 68 |
| **ENSG00000173120** | KDM2A | protein_coding | 737 | 703 | 336 | 430 | 116 | 135 | 179 | 152 |
| **ENSG00000207181** | SNORA14B | snoRNA | 521 | 632 | 892 | 630 | 126 | 128 | 203 | 118 |
| **ENSG00000221420** | SNORA81 | snoRNA | 1600 | 1177 | 1968 | 3353 | 179 | 206 | 173 | 144 |
| **ENSG00000130517** | PGPEP1 | protein_coding | 575 | 439 | 320 | 801 | 211 | 292 | 102 | 211 |
| **ENSG00000140988** | RPS2 | protein_coding | 701 | 720 | 858 | 370 | 158 | 299 | 143 | 169 |
| **ENSG00000130203** | APOE | protein_coding | 81 | 176 | 336 | 731 | 253 | 135 | 149 | 160 |
| **ENSG00000131711** | MAP1B | protein_coding | 1079 | 650 | 84 | 70 | 389 | 398 | 173 | 135 |
| **ENSG00000200983** | SNORA3A | snoRNA | 2167 | 3355 | 3903 | 1001 | 474 | 462 | 143 | 160 |
| **ENSG00000274998** | SNORA17A | snoRNA | 450 | 597 | 286 | 170 | 147 | 92 | 137 | 160 |
| **ENSG00000281808** | SNORA17 | snoRNA | 450 | 597 | 286 | 170 | 147 | 92 | 137 | 160 |
| **ENSG00000104964** | AES | retained_intron | 306 | 158 | 336 | 731 | 147 | 142 | 185 | 110 |
| **ENSG00000131016** | AKAP12 | protein_coding | 54 | 0 | 67 | 100 | 621 | 612 | 191 | 101 |
| **ENSG00000114942** | EEF1B2 | protein_coding | 261 | 334 | 521 | 540 | 137 | 171 | 131 | 160 |
| **ENSG00000133026** | MYH10 | protein_coding | 216 | 88 | 336 | 701 | 95 | 178 | 173 | 118 |
| **ENSG00000117724** | CENPF | processed_transcript | 180 | 299 | 387 | 911 | 147 | 199 | 137 | 152 |
| **ENSG00000105640** | RPL18A | protein_coding | 683 | 1282 | 1783 | 681 | 211 | 263 | 167 | 118 |
| **ENSG00000280884** | AC091053.1 |  | 1483 | 2442 | 3146 | 751 | 305 | 384 | 119 | 160 |
| **ENSG00000200156** | RNU5B-1 | snRNA | 2185 | 2108 | 4946 | 2322 | 1189 | 1344 | 185 | 93 |
| **ENSG00000281017** | MIR1248 |  | 1259 | 896 | 1464 | 2562 | 168 | 164 | 137 | 135 |
| **ENSG00000209480** | SNORD83B | snoRNA | 800 | 896 | 404 | 691 | 137 | 121 | 102 | 169 |
| **ENSG00000203875** | SNHG5 | lincRNA | 881 | 492 | 219 | 290 | 221 | 107 | 149 | 118 |
| **ENSG00000199568** | RNU5A-1 | snRNA | 1133 | 861 | 2759 | 1161 | 1084 | 1294 | 102 | 144 |
| **ENSG00000214756** | METTL12 | processed_transcript | 261 | 299 | 723 | 380 | 126 | 85 | 113 | 127 |
| **ENSG00000201457** | SNORA55 | snoRNA | 539 | 580 | 791 | 470 | 189 | 178 | 155 | 84 |
| **ENSG00000276161** | SNORA17B | snoRNA | 602 | 597 | 521 | 240 | 126 | 128 | 155 | 84 |
| **ENSG00000280496** | SNORA17B | snoRNA | 602 | 597 | 521 | 240 | 126 | 128 | 155 | 84 |
| **ENSG00000092148** | HECTD1 | protein_coding | 701 | 738 | 269 | 350 | 453 | 455 | 113 | 118 |
| **ENSG00000152818** | UTRN | protein_coding | 728 | 632 | 387 | 1031 | 347 | 363 | 84 | 144 |
| **ENSG00000206597** | SNORA57 | snoRNA | 261 | 264 | 690 | 380 | 126 | 78 | 107 | 118 |
| **ENSG00000277846** | SNORD30 | snoRNA | 369 | 492 | 673 | 711 | 105 | 43 | 102 | 118 |
| **ENSG00000207166** | SNORA68 | snoRNA | 683 | 1265 | 1783 | 681 | 211 | 256 | 125 | 93 |
| **ENSG00000142192** | APP | protein_coding | 243 | 228 | 538 | 811 | 158 | 71 | 131 | 76 |
| **ENSG00000207523** | SNORA66 | snoRNA | 683 | 949 | 1312 | 701 | 274 | 292 | 102 | 101 |
| **ENSG00000207165** | SNORA70 | snoRNA | 539 | 650 | 572 | 260 | 168 | 178 | 102 | 101 |
| **ENSG00000137801** | THBS1 | protein_coding | 189 | 351 | 841 | 540 | 158 | 164 | 155 | 34 |
| **ENSG00000201675** | SNORD32A | snoRNA | 872 | 1476 | 908 | 921 | 326 | 356 | 96 | 93 |
| **ENSG00000207475** | SNORA80E | snoRNA | 297 | 422 | 1497 | 1391 | 95 | 85 | 78 | 110 |
| **ENSG00000199347** | RNU5E-1 | snRNA | 1564 | 1230 | 4323 | 1831 | 347 | 277 | 66 | 118 |
| **ENSG00000201998** | SNORA23 | snoRNA | 387 | 685 | 959 | 600 | 84 | 135 | 72 | 110 |
| **ENSG00000206799** | SNORA32 | snoRNA | 602 | 1054 | 942 | 560 | 189 | 142 | 102 | 76 |
| **ENSG00000200084** | SNORD68 | snoRNA | 647 | 615 | 421 | 160 | 126 | 156 | 84 | 93 |
| **ENSG00000064393** | HIPK2 | protein_coding | 683 | 439 | 252 | 630 | 379 | 555 | 90 | 84 |
| **ENSG00000202363** | SNORA62 | snoRNA | 521 | 597 | 286 | 400 | 105 | 50 | 78 | 93 |
| **ENSG00000225091** | SNORA71A | snoRNA | 396 | 668 | 606 | 220 | 263 | 299 | 90 | 76 |
| **ENSG00000200534** | SNORA33 | snoRNA | 423 | 545 | 622 | 1071 | 53 | 78 | 72 | 93 |
| **ENSG00000234912** | SNHG20 | lincRNA | 234 | 316 | 992 | 390 | 126 | 263 | 90 | 68 |
| **ENSG00000100941** | PNN | protein_coding | 225 | 123 | 336 | 380 | 558 | 491 | 102 | 51 |
| **ENSG00000199477** | SNORA31 | snoRNA | 656 | 1686 | 824 | 310 | 368 | 242 | 102 | 51 |
| **ENSG00000207304** | SNORA8 | snoRNA | 414 | 404 | 572 | 500 | 179 | 78 | 48 | 101 |
| **ENSG00000275143** | SCARNA16 | scaRNA | 234 | 316 | 992 | 390 | 116 | 242 | 78 | 68 |
| **ENSG00000252481** | SCARNA13 | scaRNA | 171 | 422 | 1632 | 590 | 168 | 206 | 42 | 93 |
| **ENSG00000207008** | SNORA54 | snoRNA | 423 | 615 | 875 | 610 | 253 | 135 | 107 | 25 |
| **ENSG00000263776** | SNORA4 | snoRNA | 324 | 439 | 690 | 530 | 242 | 64 | 48 | 84 |
| **ENSG00000112972** | HMGCS1 | protein_coding | 647 | 650 | 135 | 160 | 168 | 107 | 48 | 76 |
| **ENSG00000251733** | SCARNA8 | scaRNA | 378 | 334 | 2187 | 460 | 189 | 334 | 72 | 51 |
| **ENSG00000206838** | SNORA5A | snoRNA | 306 | 316 | 1581 | 901 | 116 | 78 | 72 | 51 |
| **ENSG00000212135** | SNORD67 | snoRNA | 252 | 264 | 370 | 630 | 63 | 78 | 96 | 25 |
| **ENSG00000235408** | SNORA71B | snoRNA | 710 | 791 | 1211 | 831 | 442 | 398 | 78 | 42 |
| **ENSG00000200354** | SNORA71D | snoRNA | 620 | 949 | 404 | 290 | 421 | 313 | 72 | 42 |
| **ENSG00000239002** | SCARNA10 | snoRNA | 396 | 509 | 757 | 560 | 74 | 128 | 36 | 68 |
| **ENSG00000252010** | SCARNA5 | scaRNA | 243 | 439 | 707 | 390 | 337 | 206 | 60 | 42 |
| **ENSG00000201129** | SNORA58 | snoRNA | 252 | 246 | 757 | 420 | 63 | 28 | 66 | 34 |
| **ENSG00000222489** | SNORA79 | snoRNA | 261 | 597 | 1161 | 630 | 179 | 135 | 54 | 34 |
| **ENSG00000200394** | SNORA38B | snoRNA | 243 | 439 | 992 | 250 | 74 | 57 | 48 | 34 |
| **ENSG00000207392** | SNORA20 | snoRNA | 656 | 791 | 1043 | 831 | 116 | 92 | 54 | 25 |
| **ENSG00000251898** | SCARNA11 | scaRNA | 324 | 984 | 2473 | 630 | 232 | 149 | 42 | 34 |
| **ENSG00000281684** | AL355075.1 |  | 216 | 545 | 1060 | 520 | 179 | 121 | 42 | 34 |
| **ENSG00000280466** | SCARNA4 | scaRNA | 198 | 351 | 639 | 480 | 116 | 107 | 42 | 34 |
| **ENSG00000281394** | SCARNA4 | scaRNA | 198 | 351 | 639 | 480 | 116 | 107 | 42 | 34 |
| **ENSG00000202538** | RNU4-2 | snRNA | 1142 | 580 | 959 | 540 | 242 | 391 | 60 | 8 |
| **ENSG00000212588** | SNORA26 | snoRNA | 962 | 931 | 757 | 620 | 74 | 100 | 60 | 8 |
| **ENSG00000196586** | MYO6 | protein_coding | 81 | 16689 | 34 | 110 | 63 | 43 | 42 | 25 |
| **ENSG00000276232** | SCARNA10 | sense_intronic | 315 | 457 | 740 | 510 | 74 | 114 | 24 | 34 |
| **ENSG00000125730** | C3 | protein_coding | 710 | 492 | 1161 | 3252 | 442 | 412 | 54 | 0 |
| **ENSG00000207406** | SNORA41 | snoRNA | 252 | 316 | 505 | 520 | 105 | 114 | 36 | 17 |
| **ENSG00000206538** | VGLL3 | nonsense_mediated_decay | 2302 | 2512 | 67 | 0 | 21 | 7 | 18 | 25 |
| **ENSG00000091622** | PITPNM3 | protein_coding | 1007 | 984 | 219 | 400 | 0 | 7 | 18 | 17 |
| **ENSG00000167244** | IGF2 | protein_coding | 1313 | 1089 | 4542 | 7145 | 2074 | 1856 | 24 | 8 |
| **ENSG00000165092** | ALDH1A1 | protein_coding | 530 | 562 | 437 | 570 | 232 | 242 | 6 | 17 |
| **ENSG00000163631** | ALB | protein_coding | 1187 | 1563 | 1985 | 3463 | 853 | 804 | 0 | 17 |
| **ENSG00000129965** | INS-IGF2 | nonsense_mediated_decay | 432 | 246 | 959 | 1701 | 484 | 583 | 12 | 0 |
| **ENSG00000100867** | DHRS2 | processed_transcript | 0 | 0 | 0 | 10 | 1621 | 1593 | 6 | 0 |
| **ENSG00000240801** | AC132217.4 | 3prime_overlapping_ncRNA | 117 | 158 | 572 | 791 | 211 | 199 | 0 | 0 |
| **ENSG00000145321** | GC | protein_coding | 54 | 158 | 606 | 1011 | 0 | 7 | 0 | 0 |
