# Supplementary material for: RNA-seq reveals distinctive RNA profiles of small extracellular vesicles from different human liver cancer cell lines
Source: Oncotarget. 2017 Aug 24;8(47):82920–39. doi: 10.18632/oncotarget.20503 (PMC5669939; doi:10.18632/oncotarget.20503)
Supplement: Supplementary file 3 [file oncotarget-08-82920-s003.docx]

**Table S2. miRNAs CPM**

| **Name** | **HuH7-EVs_1** | **HuH7-EVs_2** | **Hep3B-EVs_1** | **Hep3B-EVs_2** | **HepG2-EVs_1** | **HepG2-EVs_2** | **HuH6-EVs_1** | **HuH6-EVs_2** |
| --- | --- | --- | --- | --- | --- | --- | --- | --- |
| **hsa-miR-21-5p** | 203766 | 216738 | 152506 | 113584 | 277722 | 301430 | 230608 | 184196 |
| **hsa-miR-23a-3p** | 51312 | 42891 | 14839 | 20802 | 22371 | 21892 | 6582 | 6605 |
| **hsa-miR-93-5p** | 55513 | 29771 | 26790 | 8754 | 32521 | 32207 | 9940 | 7714 |
| **hsa-miR-19b-3p** | 43061 | 30097 | 78587 | 86282 | 20841 | 20606 | 15374 | 11999 |
| **hsa-miR-92a-3p** | 29318 | 35285 | 99576 | 90955 | 54135 | 51677 | 18357 | 25092 |
| **hsa-miR-17-5p** | 36646 | 24040 | 102847 | 107227 | 28828 | 28927 | 13068 | 9027 |
| **hsa-miR-192-5p** | 37066 | 20997 | 36315 | 33052 | 36280 | 37982 | 7508 | 9057 |
| **hsa-miR-103a-3p** | 30888 | 20794 | 28143 | 54570 | 21105 | 19566 | 25836 | 13717 |
| **hsa-miR-320a** | 30594 | 16027 | 5183 | 5992 | 22181 | 17636 | 5767 | 8577 |
| **hsa-miR-16-5p** | 29398 | 16475 | 13534 | 14788 | 9498 | 10810 | 8640 | 7291 |
| **hsa-miR-122-5p** | 22975 | 13269 | 6539 | 1809 | 9985 | 11547 | 22712 | 25537 |
| **hsa-miR-29a-3p** | 17312 | 16339 | 6432 | 5330 | 40479 | 32478 | 22953 | 23141 |
| **hsa-miR-4301** | 13680 | 19802 | 3755 | 20686 | 1395 | 1022 | 2308 | 1197 |
| **hsa-miR-20a-5p** | 18587 | 11857 | 57281 | 44641 | 11658 | 14536 | 7266 | 5364 |
| **hsa-miR-30e-5p** | 6358 | 21921 | 6686 | 4008 | 12697 | 14039 | 4349 | 4708 |
| **hsa-miR-891a-5p** | 4144 | 23985 | 10687 | 4107 | 4703 | 3787 | 6235 | 2926 |
| **hsa-miR-191-5p** | 16028 | 10974 | 7796 | 9513 | 16577 | 17219 | 2314 | 2265 |
| **hsa-miR-19a-3p** | 16091 | 10879 | 23705 | 28176 | 6325 | 6948 | 5157 | 4145 |
| **hsa-miR-99a-5p** | 12613 | 10417 | 6686 | 12106 | 447 | 688 | 193 | 181 |
| **hsa-miR-23b-3p** | 9921 | 11952 | 10080 | 16834 | 7024 | 7116 | 3436 | 3025 |
| **hsa-miR-125a-5p** | 9947 | 11517 | 9505 | 12289 | 2039 | 1939 | 3115 | 2208 |
| **hsa-miR-125b-5p** | 10817 | 9371 | 4810 | 8140 | 458 | 852 | 1007 | 991 |
| **hsa-miR-30d-5p** | 11137 | 8950 | 4588 | 5397 | 4385 | 4340 | 3090 | 1823 |
| **hsa-miR-106b-5p** | 10679 | 7769 | 8359 | 6316 | 8265 | 8476 | 1968 | 2508 |
| **hsa-miR-193b-3p** | 7496 | 8760 | 3620 | 6006 | 17295 | 15035 | 1130 | 1309 |
| **hsa-miR-25-3p** | 8958 | 6153 | 3585 | 3693 | 5128 | 5873 | 2090 | 2430 |
| **hsa-miR-15b-5p** | 7839 | 6967 | 5897 | 9684 | 3184 | 3574 | 2862 | 3380 |
| **hsa-miR-140-3p** | 9893 | 4808 | 1106 | 913 | 7606 | 7536 | 748 | 631 |
| **hsa-miR-30b-5p** | 7522 | 6967 | 3394 | 3665 | 3162 | 3656 | 850 | 777 |
| **hsa-miR-451a** | 7413 | 7035 | 12681 | 4786 | 36972 | 39699 | 2326 | 2420 |
| **hsa-miR-27a-3p** | 7942 | 5867 | 3442 | 3399 | 4023 | 4036 | 1094 | 1610 |
| **hsa-miR-3182** | 3735 | 9154 | 5837 | 6912 | 9930 | 6430 | 12742 | 3461 |
| **hsa-miR-424-5p** | 7645 | 4713 | 1340 | 1389 | 622 | 807 | 1638 | 1891 |
| **hsa-miR-181b-5p** | 5806 | 5990 | 13260 | 13334 | 878 | 712 | 911 | 1001 |
| **hsa-miR-590-5p** | 7519 | 3545 | 1277 | 1259 | 3807 | 3955 | 1472 | 1954 |
| **hsa-miR-148a-3p** | 6652 | 4346 | 2724 | 2265 | 1351 | 1505 | 1361 | 1412 |
| **hsa-miR-18a-5p** | 6898 | 4047 | 30803 | 41874 | 8162 | 6423 | 4867 | 2880 |
| **hsa-miR-221-3p** | 10322 | 312 | 6372 | 5789 | 9703 | 9076 | 3883 | 5162 |
| **hsa-miR-27b-3p** | 4230 | 4713 | 3430 | 4209 | 3682 | 3707 | 964 | 1717 |
| **hsa-miR-185-5p** | 5620 | 2852 | 2375 | 2836 | 3733 | 3871 | 1286 | 1946 |
| **hsa-miR-34a-5p** | 4725 | 3531 | 4762 | 4395 | 17844 | 16484 | 15553 | 18818 |
| **hsa-miR-182-5p** | 4928 | 2988 | 1741 | 3082 | 1567 | 1430 | 3270 | 1625 |
| **hsa-miR-1469** | 2960 | 3803 | 1479 | 332 | 2229 | 1475 | 1396 | 329 |
| **hsa-miR-101-3p** | 3546 | 2540 | 1923 | 2541 | 5289 | 5336 | 2292 | 2766 |
| **hsa-miR-128-3p** | 3484 | 2404 | 1483 | 2002 | 3960 | 3985 | 459 | 936 |
| **hsa-miR-130b-3p** | 4244 | 1399 | 2256 | 2724 | 2976 | 3112 | 1227 | 1061 |
| **hsa-miR-574-3p** | 3123 | 2513 | 1753 | 2769 | 3715 | 3808 | 988 | 803 |
| **hsa-miR-186-5p** | 3489 | 2064 | 1229 | 1146 | 2255 | 2312 | 1293 | 1744 |
| **hsa-miR-26b-5p** | 2843 | 2513 | 3180 | 2464 | 3148 | 3752 | 492 | 749 |
| **hsa-miR-15a-5p** | 2806 | 2363 | 1182 | 997 | 1951 | 2046 | 766 | 870 |
| **hsa-miR-425-5p** | 2889 | 2214 | 1935 | 2101 | 4250 | 4356 | 1344 | 1002 |
| **hsa-miR-222-3p** | 2437 | 2363 | 1872 | 1918 | 2401 | 2203 | 1512 | 1242 |
| **hsa-miR-190a-3p** | 2485 | 2268 | 2375 | 825 | 1530 | 929 | 94 | 379 |
| **hsa-miR-210-3p** | 2846 | 1901 | 2570 | 2692 | 2965 | 2716 | 1195 | 1120 |
| **hsa-miR-4792** | 950 | 3721 | 2229 | 1023 | 2247 | 1797 | 45 | 46 |
| **hsa-miR-22-5p** | 2728 | 1888 | 730 | 622 | 780 | 691 | 19 | 1168 |
| **hsa-miR-335-5p** | 2843 | 1657 | 278 | 310 | 216 | 240 | 13 | 9 |
| **hsa-miR-218-5p** | 2769 | 1603 | 567 | 833 | 62 | 40 | 505 | 653 |
| **hsa-miR-301a-3p** | 2588 | 1684 | 9493 | 9853 | 7482 | 6955 | 2119 | 1029 |
| **hsa-miR-126-3p** | 2932 | 1304 | 2518 | 964 | 3583 | 4615 | 271 | 397 |
| **hsa-miR-4716-5p** | 492 | 3450 | 1340 | 1164 | 575 | 401 | 656 | 445 |
| **hsa-miR-4291** | 255 | 3640 | 1547 | 2194 | 366 | 338 | 338 | 439 |
| **hsa-miR-18b-5p** | 2062 | 1820 | 2768 | 5868 | 1468 | 1288 | 577 | 1214 |
| **hsa-miR-484** | 1650 | 2227 | 1420 | 1941 | 3331 | 3329 | 845 | 898 |
| **hsa-miR-532-5p** | 2134 | 1589 | 868 | 1005 | 2471 | 2060 | 3162 | 3394 |
| **hsa-miR-126-5p** | 2197 | 1467 | 1967 | 581 | 2943 | 4151 | 136 | 337 |
| **hsa-miR-22-3p** | 1693 | 1929 | 1007 | 885 | 732 | 866 | 1037 | 1080 |
| **hsa-miR-483-5p** | 1770 | 1834 | 4414 | 1350 | 4817 | 4151 | 8 | 7 |
| **hsa-miR-99b-5p** | 1845 | 1752 | 1598 | 3099 | 523 | 642 | 655 | 641 |
| **hsa-miR-30a-5p** | 1839 | 1725 | 3303 | 2789 | 329 | 390 | 674 | 433 |
| **hsa-miR-194-5p** | 1359 | 2037 | 2994 | 2834 | 1204 | 1244 | 941 | 758 |
| **hsa-miR-6768-5p** | 400 | 2947 | 662 | 412 | 304 | 219 | 247 | 229 |
| **hsa-miR-365b-3p** | 841 | 2472 | 623 | 1408 | 1409 | 1447 | 236 | 415 |
| **hsa-miR-660-5p** | 1739 | 1494 | 1158 | 1113 | 3272 | 3157 | 6710 | 6015 |
| **hsa-miR-138-5p** | 2062 | 1019 | 40 | 45 | 871 | 1050 | 489 | 402 |
| **hsa-miR-183-5p** | 1933 | 1005 | 1443 | 2340 | 1442 | 1489 | 1361 | 1416 |
| **hsa-miR-331-3p** | 1490 | 1290 | 1344 | 3116 | 919 | 826 | 639 | 812 |
| **hsa-miR-215-5p** | 1742 | 1032 | 107 | 150 | 70 | 37 | 9 | 7 |
| **hsa-miR-455-3p** | 1370 | 1399 | 1265 | 2564 | 2024 | 1916 | 877 | 818 |
| **hsa-miR-193a-3p** | 460 | 2282 | 706 | 403 | 110 | 168 | 231 | 271 |
| **hsa-miR-374b-5p** | 1519 | 1168 | 567 | 871 | 1047 | 905 | 542 | 633 |
| **hsa-miR-4320** | 475 | 2146 | 1221 | 874 | 1870 | 4529 | 55 | 89 |
| **hsa-miR-423-3p** | 1193 | 1399 | 793 | 1192 | 1351 | 1094 | 503 | 359 |
| **hsa-miR-146a-5p** | 1481 | 1059 | 4366 | 4812 | 2434 | 2060 | 139 | 128 |
| **hsa-miR-6721-5p** | 1261 | 1236 | 1388 | 1225 | 10278 | 4417 | 162 | 106 |
| **hsa-miR-423-5p** | 1356 | 1127 | 714 | 822 | 1102 | 1085 | 491 | 470 |
| **hsa-miR-371a-5p** | 340 | 2132 | 246 | 73 | 922 | 616 | 115940 | 172579 |
| **hsa-miR-17-3p** | 1221 | 1141 | 3664 | 3099 | 944 | 903 | 492 | 648 |
| **hsa-miR-1260a** | 395 | 1861 | 278 | 326 | 1113 | 1048 | 112 | 197 |
| **hsa-miR-744-5p** | 1115 | 1046 | 555 | 853 | 1438 | 1013 | 186 | 16 |
| **hsa-miR-106b-3p** | 1187 | 869 | 428 | 600 | 630 | 628 | 201 | 374 |
| **hsa-miR-129-2-3p** | 109 | 1901 | 432 | 223 | 1354 | 966 | 290 | 340 |
| **hsa-miR-4516** | 452 | 1453 | 658 | 600 | 1179 | 114 | 190 | 144 |
| **hsa-miR-421** | 1296 | 584 | 1134 | 994 | 1548 | 1645 | 1249 | 1118 |
| **hsa-miR-671-5p** | 1050 | 828 | 194 | 197 | 498 | 481 | 784 | 278 |
| **hsa-miR-373-3p** | 257 | 1535 | 289 | 93 | 1047 | 775 | 208268 | 224334 |
| **hsa-miR-99b-3p** | 970 | 693 | 623 | 690 | 351 | 243 | 92 | 215 |
| **hsa-miR-4315** | 406 | 1250 | 892 | 561 | 1149 | 1808 | 196 | 215 |
| **hsa-miR-200a-3p** | 858 | 788 | 5337 | 6140 | 1940 | 1995 | 7813 | 6407 |
| **hsa-miR-92b-3p** | 778 | 842 | 178 | 343 | 454 | 380 | 54 | 103 |
| **hsa-miR-339-5p** | 581 | 978 | 345 | 533 | 926 | 611 | 325 | 298 |
| **hsa-miR-452-5p** | 924 | 584 | 12 | 0 | 966 | 950 | 13 | 7 |
| **hsa-miR-107** | 815 | 679 | 1416 | 2628 | 1080 | 924 | 564 | 483 |
| **hsa-miR-7-5p** | 744 | 733 | 123 | 150 | 443 | 418 | 1548 | 1135 |
| **hsa-miR-371a-3p** | 112 | 1345 | 87 | 23 | 256 | 271 | 50060 | 57400 |
| **hsa-miR-24-3p** | 672 | 747 | 171 | 344 | 150 | 135 | 79 | 32 |
| **hsa-miR-16-2-3p** | 692 | 706 | 674 | 479 | 421 | 401 | 128 | 366 |
| **hsa-miR-487b-5p** | 112 | 1195 | 416 | 364 | 227 | 105 | 183 | 96 |
| **hsa-miR-197-3p** | 552 | 638 | 262 | 437 | 937 | 922 | 459 | 496 |
| **hsa-let-7e-5p** | 455 | 611 | 3426 | 6687 | 556 | 614 | 156 | 173 |
| **hsa-miR-140-5p** | 638 | 407 | 167 | 85 | 443 | 546 | 78 | 56 |
| **hsa-miR-551b-3p** | 641 | 380 | 837 | 1440 | 15 | 0 | 3 | 3 |
| **hsa-miR-455-5p** | 632 | 380 | 567 | 636 | 1244 | 1097 | 297 | 217 |
| **hsa-miR-320b** | 503 | 503 | 488 | 141 | 567 | 670 | 139 | 253 |
| **hsa-let-7f-5p** | 446 | 557 | 1983 | 3042 | 1969 | 1710 | 72 | 162 |
| **hsa-miR-181a-3p** | 403 | 584 | 904 | 676 | 315 | 285 | 158 | 135 |
| **hsa-miR-93-3p** | 612 | 353 | 258 | 307 | 794 | 681 | 141 | 156 |
| **hsa-miR-3975** | 63 | 869 | 4017 | 98 | 362 | 882 | 85 | 105 |
| **hsa-miR-4295** | 541 | 326 | 305 | 73 | 5571 | 6514 | 14870 | 12734 |
| **hsa-miR-21-3p** | 412 | 448 | 369 | 279 | 1263 | 1255 | 382 | 492 |
| **hsa-miR-200b-3p** | 501 | 340 | 2581 | 3068 | 1358 | 1295 | 3903 | 3618 |
| **hsa-miR-454-3p** | 443 | 353 | 698 | 1315 | 428 | 261 | 223 | 91 |
| **hsa-miR-200c-3p** | 66 | 720 | 194 | 79 | 168 | 128 | 3650 | 2861 |
| **hsa-miR-184** | 360 | 394 | 1174 | 56 | 4 | 37 | 4 | 6 |
| **hsa-miR-34a-3p** | 309 | 421 | 361 | 248 | 1548 | 1503 | 1457 | 1552 |
| **hsa-miR-378c** | 415 | 312 | 163 | 105 | 2119 | 2387 | 1644 | 1813 |
| **hsa-miR-192-3p** | 458 | 258 | 444 | 428 | 666 | 427 | 138 | 153 |
| **hsa-let-7g-5p** | 306 | 407 | 3260 | 3189 | 4414 | 3010 | 109 | 150 |
| **hsa-miR-4532** | 143 | 557 | 115 | 253 | 472 | 546 | 79 | 67 |
| **hsa-miR-596** | 189 | 462 | 214 | 206 | 1490 | 513 | 39 | 12 |
| **hsa-miR-100-5p** | 363 | 285 | 412 | 834 | 318 | 343 | 774 | 694 |
| **hsa-let-7c-5p** | 292 | 312 | 2510 | 2535 | 77 | 82 | 11 | 11 |
| **hsa-miR-145-5p** | 303 | 299 | 567 | 166 | 5776 | 5693 | 373 | 353 |
| **hsa-let-7a-5p** | 223 | 367 | 3664 | 6003 | 1534 | 1297 | 183 | 193 |
| **hsa-miR-181d-5p** | 335 | 244 | 571 | 639 | 212 | 308 | 34 | 12 |
| **hsa-miR-29c-3p** | 329 | 244 | 1487 | 1980 | 3049 | 2566 | 1350 | 1393 |
| **hsa-miR-4488** | 132 | 435 | 123 | 310 | 1018 | 329 | 74 | 58 |
| **hsa-miR-223-3p** | 197 | 353 | 658 | 149 | 1545 | 1685 | 95 | 104 |
| **hsa-miR-324-5p** | 154 | 367 | 448 | 688 | 348 | 369 | 193 | 83 |
| **hsa-miR-144-3p** | 206 | 299 | 603 | 141 | 1131 | 1029 | 25 | 83 |
| **hsa-miR-144-5p** | 323 | 81 | 361 | 95 | 882 | 1062 | 30 | 48 |
| **hsa-miR-188-5p** | 174 | 204 | 99 | 135 | 436 | 322 | 718 | 899 |
| **hsa-miR-301b-3p** | 232 | 81 | 559 | 654 | 725 | 744 | 267 | 205 |
| **hsa-miR-372-3p** | 0 | 312 | 24 | 2 | 33 | 49 | 18898 | 14795 |
| **hsa-let-7b-5p** | 89 | 217 | 1075 | 895 | 388 | 425 | 15 | 25 |
| **hsa-miR-502-3p** | 149 | 149 | 95 | 188 | 395 | 322 | 594 | 631 |
| **hsa-miR-199b-5p** | 112 | 177 | 159 | 67 | 835 | 607 | 105 | 108 |
| **hsa-miR-31-5p** | 200 | 68 | 24 | 9 | 48 | 23 | 523 | 573 |
| **hsa-miR-369-3p** | 209 | 54 | 254 | 59 | 359 | 695 | 26 | 61 |
| **hsa-miR-429** | 100 | 163 | 246 | 555 | 231 | 362 | 609 | 643 |
| **hsa-miR-146b-5p** | 40 | 177 | 83 | 87 | 1069 | 1090 | 669 | 291 |
| **hsa-miR-20b-5p** | 103 | 109 | 99 | 140 | 110 | 112 | 2255 | 1306 |
| **hsa-miR-199b-3p** | 126 | 81 | 147 | 29 | 498 | 576 | 51 | 47 |
| **hsa-miR-10a-5p** | 54 | 122 | 210 | 25 | 135 | 154 | 3251 | 3084 |
| **hsa-miR-501-5p** | 77 | 68 | 95 | 109 | 161 | 135 | 768 | 472 |
| **hsa-miR-205-5p** | 29 | 81 | 131 | 17 | 381 | 119 | 493 | 598 |
| **hsa-let-7d-5p** | 80 | 27 | 591 | 592 | 523 | 569 | 38 | 46 |
| **hsa-miR-135b-5p** | 9 | 95 | 32 | 42 | 1991 | 2300 | 2586 | 2034 |
| **hsa-miR-139-5p** | 54 | 41 | 147 | 25 | 443 | 581 | 449 | 341 |
| **hsa-miR-141-3p** | 9 | 81 | 16 | 25 | 22 | 12 | 1348 | 1022 |
| **hsa-miR-150-5p** | 49 | 41 | 182 | 42 | 681 | 665 | 56 | 37 |
| **hsa-miR-143-3p** | 37 | 41 | 95 | 20 | 663 | 579 | 38 | 79 |
| **hsa-miR-372-5p** | 3 | 54 | 0 | 3 | 7 | 7 | 581 | 739 |
| **hsa-miR-10b-5p** | 43 | 14 | 155 | 42 | 311 | 404 | 1566 | 1571 |
| **hsa-miR-505-3p** | 20 | 27 | 282 | 293 | 1010 | 1157 | 31 | 44 |
| **hsa-miR-551a** | 17 | 27 | 8 | 5 | 3397 | 3024 | 261 | 449 |
| **hsa-miR-373-5p** | 0 | 41 | 4 | 3 | 33 | 7 | 1448 | 1159 |
