# Supplementary material for: RNA-seq reveals distinctive RNA profiles of small extracellular vesicles from different human liver cancer cell lines
Source: Oncotarget. 2017 Aug 24;8(47):82920–39. doi: 10.18632/oncotarget.20503 (PMC5669939; doi:10.18632/oncotarget.20503)
Supplement: Supplementary file 4 [file oncotarget-08-82920-s004.docx]

**Table S3 A. IsomiR CPM**

| **IsomiR Short ID** | **HuH7-EVs_1** | **HuH7-EVs_2** | **Hep3B-EVs_1** | **Hep3B-EVs_2** | **HepG2-EVs_1** | **HepG2-EVs_2** | **HuH6-EVs_1** | **HuH6-EVs_2** | **Sequence** |
| --- | --- | --- | --- | --- | --- | --- | --- | --- | --- |
| **hsa-let-7a-5p.3.P0.M.0** | 26 | 77 | 253 | 1.148 | 276 | 200 | 29 | 27 | tgaggtagtaggttgtatagtta |
| **hsa-let-7a-5p.3.P0.M.5** | 258 | 116 | 1.580 | 2.867 | 1.152 | 1.194 | 175 | 165 | tgaggtagtaggttgtatagt |
| **hsa-let-7a-5p.3.P1.M.4** | 33 | 0 | 280 | 722 | 59 | 62 | 10 | 17 | tgaggtaggaggttgtatagttt |
| **hsa-let-7e-5p.3.P0.S.1** | 26 | 116 | 316 | 931 | 98 | 76 | 22 | 23 | tgaggtaggaggttgtatagtta |
| **hsa-let-7e-5p.3.P0.S.12** | 7 | 39 | 334 | 870 | 59 | 14 | 26 | 16 | tgaggtaggaggttgtatagttg |
| **hsa-let-7e-5p.3.P0.S.14** | 99 | 0 | 605 | 1.918 | 148 | 131 | 38 | 33 | tgaggtaggaggttgtatagt |
| **hsa-let-7f-5p.3.P0.M.5** | 53 | 116 | 298 | 522 | 699 | 456 | 10 | 53 | tgaggtagtagattgtatagt |
| **hsa-let-7g-5p.3.P0.S.11** | 86 | 310 | 442 | 579 | 1.467 | 635 | 29 | 53 | tgaggtagtagtttgtacagt |
| **hsa-miR-100-5p.3.P0.S.4** | 185 | 232 | 271 | 400 | 541 | 580 | 344 | 431 | aacccgtagatccgaacttgt |
| **hsa-miR-101-3p.53.P0.M.9** | 2.437 | 2.517 | 1.092 | 1.383 | 1.614 | 4.018 | 609 | 1.690 | gtacagtactgtgataactga |
| **hsa-miR-103a-3p.0.P1.M.58** | 1.103 | 387 | 614 | 726 | 325 | 435 | 558 | 3.106 | agcagcattgtacagggctatgc |
| **hsa-miR-103a-3p.0.P1.M.60** | 4.181 | 3.175 | 2.320 | 5.529 | 1.939 | 1.926 | 3.763 | 1.069 | agcagcattgtacagggctatgt |
| **hsa-miR-103a-3p.3.P0.M.4** | 429 | 310 | 1.147 | 2.701 | 650 | 532 | 1.451 | 383 | agcagcattgtacagggctatgat |
| **hsa-miR-103a-3p.3.P0.M.5** | 3.230 | 2.672 | 2.645 | 6.421 | 2.500 | 2.458 | 3.569 | 2.779 | agcagcattgtacagggctatg |
| **hsa-miR-103a-3p.3.P0.M.6** | 284 | 503 | 163 | 1.205 | 364 | 338 | 1.292 | 235 | agcagcattgtacagggctatgaa |
| **hsa-miR-103a-3p.3.P0.M.7** | 443 | 503 | 343 | 609 | 679 | 1.063 | 354 | 596 | agcagcattgtacagggct |
| **hsa-miR-103a-3p.3.P0.M.8** | 4.987 | 6.118 | 2.681 | 4.459 | 8.120 | 6.911 | 4.446 | 6.160 | agcagcattgtacagggctat |
| **hsa-miR-103a-3p.3.P0.M.9** | 271 | 310 | 280 | 113 | 561 | 960 | 99 | 132 | agcagcattgtacagggc |
| **hsa-miR-103a-3p.5.P0.M.2** | 1.057 | 503 | 867 | 1.266 | 2.215 | 2.354 | 705 | 497 | cagcattgtacagggctatga |
| **hsa-miR-106a-5p.3.P1.M.1** | 13 | 0 | 1.291 | 13 | 0 | 14 | 3 | 0 | caaagtgcttacagtgcaggtagc |
| **hsa-miR-106b-3p.3.P0.S.2** | 1.466 | 1.084 | 533 | 1.101 | 768 | 1.070 | 325 | 539 | ccgcactgtgggtacttgctg |
| **hsa-miR-106b-5p.0.P1.S.30** | 73 | 774 | 298 | 1.683 | 1.437 | 166 | 19 | 14 | taaagtgctgacagtgcagac |
| **hsa-miR-106b-5p.3.P0.S.11** | 7.530 | 5.692 | 6.347 | 5.351 | 6.152 | 6.393 | 2.047 | 2.703 | taaagtgctgacagtgcagata |
| **hsa-miR-106b-5p.3.P0.S.14** | 1.387 | 1.588 | 1.327 | 1.074 | 1.408 | 2.672 | 185 | 378 | taaagtgctgacagtgcaga |
| **hsa-miR-106b-5p.3.P0.S.8** | 839 | 697 | 379 | 100 | 315 | 490 | 233 | 163 | taaagtgctgacagtgcagatag |
| **hsa-miR-107.3.P0.S.5** | 654 | 620 | 930 | 1.692 | 709 | 635 | 373 | 258 | agcagcattgtacagggctatc |
| **hsa-miR-10a-5p.3.P0.S.3** | 73 | 39 | 90 | 17 | 187 | 193 | 4.101 | 4.509 | taccctgtagatccgaatttgt |
| **hsa-miR-10b-5p.3.P0.S.3** | 59 | 0 | 190 | 48 | 384 | 518 | 2.427 | 2.535 | taccctgtagaaccgaatttgt |
| **hsa-miR-122-5p.3.P0.S.1** | 3.197 | 3.369 | 289 | 226 | 59 | 166 | 9.752 | 6.607 | tggagtgtgacaatggtgtttga |
| **hsa-miR-122-5p.3.P0.S.13** | 10.343 | 7.241 | 9.985 | 2.588 | 18.347 | 3.514 | 13.831 | 20.557 | tggagtgtgacaatggtgttt |
| **hsa-miR-122-5p.3.P0.S.14** | 363 | 348 | 289 | 48 | 207 | 28 | 836 | 1.185 | tggagtgtgacaatggtgtt |
| **hsa-miR-1246.3.P0.S.3** | 2.305 | 1.394 | 713 | 1.040 | 2.126 | 1.643 | 918 | 623 | aatggatttttggagcaggg |
| **hsa-miR-1246.53.P0.S.1** | 845 | 194 | 0 | 0 | 20 | 14 | 96 | 60 | atggatttttggagcagggg |
| **hsa-miR-125a-5p.0.P1.S.32** | 535 | 387 | 397 | 644 | 30 | 55 | 195 | 135 | tccctgagaccctttaacctgtgt |
| **hsa-miR-125a-5p.3.P0.S.4** | 4.894 | 6.118 | 4.623 | 8.383 | 955 | 580 | 1.534 | 1.088 | tccctgagaccctttaacctgtg |
| **hsa-miR-125a-5p.3.P0.S.6** | 4.379 | 7.783 | 3.494 | 8.309 | 2.284 | 2.437 | 1.588 | 1.773 | tccctgagaccctttaacctgt |
| **hsa-miR-125b-5p.3.P0.M.3** | 1.374 | 1.433 | 542 | 1.375 | 30 | 124 | 48 | 75 | tccctgagaccctaacttgtg |
| **hsa-miR-125b-5p.3.P0.M.4** | 614 | 968 | 334 | 365 | 394 | 621 | 22 | 73 | tccctgagaccctaacttgt |
| **hsa-miR-1260a.0.P1.M.0** | 753 | 4.569 | 542 | 709 | 1.831 | 2.209 | 195 | 394 | atcccaccgctgccacca |
| **hsa-miR-1260b.3.P0.S.1** | 139 | 929 | 18 | 30 | 157 | 159 | 3 | 16 | atcccaccactgccacca |
| **hsa-miR-126-3p.3.P0.S.0** | 456 | 426 | 677 | 187 | 1.171 | 1.899 | 112 | 113 | tcgtaccgtgagtaataatgcga |
| **hsa-miR-126-3p.5.P0.S.3** | 1.770 | 1.162 | 1.165 | 753 | 1.742 | 2.541 | 150 | 241 | cgtaccgtgagtaataatgcg |
| **hsa-miR-126-3p.53.P0.S.3** | 1.341 | 348 | 1.300 | 278 | 1.988 | 3.093 | 140 | 221 | cgtaccgtgagtaataatgcga |
| **hsa-miR-126-3p.53.P0.S.5** | 1.057 | 348 | 533 | 178 | 876 | 1.132 | 108 | 115 | cgtaccgtgagtaataatgcgt |
| **hsa-miR-126-5p.3.P0.S.0** | 601 | 620 | 785 | 222 | 1.575 | 1.823 | 83 | 169 | cattattacttttggtacgcga |
| **hsa-miR-128-3p.3.P0.M.4** | 674 | 465 | 253 | 392 | 1.014 | 967 | 163 | 267 | tcacagtgaaccggtctctt |
| **hsa-miR-128-3p.3.P0.S.5** | 1.380 | 852 | 415 | 696 | 817 | 711 | 112 | 123 | tcacagtgaaccggtctctttt |
| **hsa-miR-130b-3p.3.P0.S.12** | 1.229 | 387 | 641 | 644 | 925 | 1.250 | 332 | 328 | cagtgcaatgatgaaagggca |
| **hsa-miR-130b-3p.3.P0.S.6** | 2.147 | 581 | 867 | 1.231 | 1.122 | 918 | 364 | 156 | cagtgcaatgatgaaagggcatt |
| **hsa-miR-135b-5p.3.P0.S.4** | 0 | 0 | 27 | 13 | 502 | 532 | 615 | 600 | tatggcttttcattcctatgtg |
| **hsa-miR-138-5p.3.P0.S.3** | 898 | 310 | 0 | 26 | 187 | 242 | 204 | 69 | agctggtgttgtgaatcaggccgt |
| **hsa-miR-140-3p.3.P0.S.15** | 1.625 | 736 | 172 | 204 | 1.329 | 1.616 | 137 | 116 | taccacagggtagaaccacgga |
| **hsa-miR-140-3p.5.P0.S.1** | 773 | 542 | 54 | 57 | 433 | 614 | 26 | 49 | accacagggtagaaccacgg |
| **hsa-miR-140-3p.53.P0.S.2** | 984 | 620 | 135 | 126 | 906 | 1.270 | 89 | 107 | accacagggtagaaccacggaa |
| **hsa-miR-140-3p.53.P0.S.20** | 2.391 | 1.317 | 135 | 270 | 2.362 | 2.451 | 198 | 118 | accacagggtagaaccacggaca |
| **hsa-miR-140-3p.53.P0.S.21** | 6.420 | 2.981 | 632 | 783 | 6.427 | 5.792 | 399 | 358 | accacagggtagaaccacggac |
| **hsa-miR-140-3p.53.P0.S.22** | 5.264 | 3.330 | 515 | 496 | 4.055 | 4.460 | 364 | 361 | accacagggtagaaccacgga |
| **hsa-miR-144-5p.53.P0.S.4** | 363 | 194 | 497 | 100 | 1.388 | 1.595 | 35 | 75 | tgggatatcatcatatactgt |
| **hsa-miR-145-5p.3.P0.S.12** | 86 | 271 | 172 | 74 | 1.654 | 1.767 | 51 | 64 | gtccagttttcccaggaatccc |
| **hsa-miR-145-5p.3.P0.S.13** | 7 | 0 | 9 | 9 | 896 | 739 | 51 | 20 | gtccagttttcccaggaatccctt |
| **hsa-miR-145-5p.3.P0.S.6** | 198 | 155 | 397 | 104 | 1.467 | 1.332 | 115 | 195 | gtccagttttcccaggaat |
| **hsa-miR-145-5p.3.P0.S.8** | 125 | 39 | 208 | 61 | 1.772 | 2.403 | 131 | 163 | gtccagttttcccaggaatcc |
| **hsa-miR-1469.53.P1.S.4** | 1.334 | 116 | 442 | 109 | 128 | 28 | 1.046 | 3 | cggcgcggggcgcgggat |
| **hsa-miR-1469.53.P1.S.7** | 66 | 1.162 | 18 | 22 | 472 | 97 | 45 | 0 | cggcgcggggcgcgggca |
| **hsa-miR-146a-5p.3.P0.S.0** | 205 | 39 | 659 | 487 | 315 | 180 | 10 | 11 | tgagaactgaattccatgggtta |
| **hsa-miR-146a-5p.3.P0.S.11** | 1.361 | 620 | 2.997 | 3.615 | 2.264 | 1.823 | 86 | 19 | tgagaactgaattccatgggttg |
| **hsa-miR-146a-5p.3.P0.S.16** | 159 | 155 | 641 | 905 | 384 | 387 | 13 | 16 | tgagaactgaattccatgggt |
| **hsa-miR-146a-5p.3.P0.S.7** | 92 | 116 | 650 | 718 | 305 | 345 | 6 | 9 | tgagaactgaattccatgggttt |
| **hsa-miR-146b-5p.3.P0.S.10** | 13 | 39 | 18 | 70 | 522 | 677 | 325 | 32 | tgagaactgaattccataggctg |
| **hsa-miR-146b-5p.3.P0.S.12** | 59 | 0 | 99 | 44 | 1.368 | 1.443 | 883 | 211 | tgagaactgaattccataggctgt |
| **hsa-miR-148a-3p.3.P0.S.11** | 2.721 | 3.717 | 1.165 | 992 | 974 | 1.022 | 1.052 | 1.069 | tcagtgcactacagaactttg |
| **hsa-miR-148a-3p.3.P0.S.7** | 925 | 581 | 578 | 979 | 138 | 152 | 351 | 222 | tcagtgcactacagaactttgtc |
| **hsa-miR-15a-5p.3.P0.S.8** | 4.551 | 4.143 | 1.255 | 1.718 | 3.711 | 4.570 | 1.135 | 1.552 | tagcagcacataatggtttgt |
| **hsa-miR-15b-3p.3.P0.S.3** | 654 | 620 | 271 | 396 | 157 | 297 | 92 | 178 | cgaatcattatttgctgctct |
| **hsa-miR-15b-5p.3.P0.S.7** | 542 | 503 | 217 | 383 | 315 | 428 | 92 | 374 | tagcagcacatcatggttt |
| **hsa-miR-15b-5p.3.P0.S.8** | 5.053 | 4.879 | 289 | 6.451 | 3.160 | 3.811 | 1.952 | 2.674 | tagcagcacatcatggtttac |
| **hsa-miR-15b-5p.3.P0.S.9** | 1.380 | 2.130 | 650 | 1.114 | 640 | 766 | 494 | 919 | tagcagcacatcatggttta |
| **hsa-miR-16-2-3p.53.P0.S.3** | 859 | 774 | 876 | 731 | 177 | 580 | 252 | 443 | accaatattactgtgctgcttt |
| **hsa-miR-16-5p.3.P0.M.0** | 2.186 | 2.478 | 343 | 2.262 | 1.024 | 808 | 1.033 | 808 | tagcagcacgtaaatattggcga |
| **hsa-miR-16-5p.3.P0.M.11** | 6.189 | 3.950 | 2.736 | 7.452 | 2.579 | 1.836 | 3.680 | 2.477 | tagcagcacgtaaatattggcgt |
| **hsa-miR-16-5p.3.P0.M.7** | 1.869 | 1.433 | 795 | 835 | 541 | 739 | 660 | 572 | tagcagcacgtaaatattgg |
| **hsa-miR-16-5p.3.P0.M.8** | 1.598 | 1.084 | 1.354 | 831 | 600 | 808 | 526 | 334 | tagcagcacgtaaatattg |
| **hsa-miR-16-5p.3.P1.M.15** | 1.321 | 465 | 542 | 261 | 285 | 366 | 204 | 208 | tagcagcacgtaaatattgc |
| **hsa-miR-16-5p.3.P1.M.48** | 92 | 77 | 99 | 13 | 610 | 946 | 29 | 26 | tagcagcacgtaaatattggt |
| **hsa-miR-17-3p.3.P0.S.10** | 152 | 39 | 298 | 722 | 118 | 83 | 70 | 107 | actgcagtgaaggcacttgta |
| **hsa-miR-17-3p.3.P0.S.12** | 198 | 155 | 885 | 174 | 276 | 421 | 41 | 109 | actgcagtgaaggcacttg |
| **hsa-miR-17-3p.3.P0.S.6** | 1.347 | 1.549 | 3.205 | 3.650 | 1.122 | 1.153 | 322 | 695 | actgcagtgaaggcacttgt |
| **hsa-miR-17-5p.0.P1.S.45** | 1.129 | 2.091 | 4.830 | 4.428 | 1.457 | 1.201 | 1.005 | 735 | caaagtgcttacagtgcaggtaa |
| **hsa-miR-17-5p.0.P1.S.46** | 106 | 155 | 2.113 | 8.122 | 98 | 83 | 77 | 44 | caaagtgcttacagtgcaggtac |
| **hsa-miR-17-5p.0.P1.S.47** | 2.404 | 3.524 | 13.407 | 18.640 | 4.606 | 3.770 | 3.836 | 2.445 | caaagtgcttacagtgcaggtat |
| **hsa-miR-17-5p.3.P0.S.11** | 11.453 | 9.138 | 32.015 | 47.360 | 19.942 | 21.036 | 2.784 | 3.281 | caaagtgcttacagtgcaggt |
| **hsa-miR-17-5p.3.P0.S.13** | 3.580 | 2.091 | 13.498 | 20.337 | 2.599 | 3.915 | 1.202 | 1.539 | caaagtgcttacagtgcaggta |
| **hsa-miR-17-5p.3.P0.S.15** | 627 | 658 | 2.257 | 1.697 | 965 | 967 | 297 | 404 | caaagtgcttacagtgcagg |
| **hsa-miR-17-5p.3.P0.S.19** | 2.483 | 1.936 | 8.415 | 12.485 | 2.963 | 2.416 | 3.049 | 1.323 | caaagtgcttacagtgcaggtagt |
| **hsa-miR-17-5p.3.P0.S.2** | 1.176 | 1.239 | 2.429 | 2.632 | 591 | 601 | 903 | 334 | caaagtgcttacagtgcaggtaga |
| **hsa-miR-17-5p.3.P1.S.29** | 33 | 0 | 5.832 | 57 | 10 | 41 | 0 | 11 | caaagtgcttacagtgcaggc |
| **hsa-miR-17-5p.3.P1.S.66** | 139 | 232 | 677 | 970 | 128 | 338 | 102 | 79 | caaagtgcttacagtgcaggtt |
| **hsa-miR-17-5p.5.P0.S.4** | 575 | 581 | 903 | 622 | 236 | 311 | 210 | 79 | tcaaagtgcttacagtgcaggtag |
| **hsa-miR-17-5p.5.P1.S.6** | 225 | 271 | 831 | 735 | 482 | 359 | 230 | 93 | tcaaagtgcttacagtgcaggtat |
| **hsa-miR-17-5p.53.P0.S.12** | 1.433 | 1.510 | 3.963 | 3.793 | 2.599 | 2.838 | 309 | 391 | tcaaagtgcttacagtgcaggt |
| **hsa-miR-17-5p.53.P0.S.14** | 258 | 387 | 1.264 | 1.462 | 354 | 262 | 124 | 166 | tcaaagtgcttacagtgcaggta |
| **hsa-miR-181a-5p.3.P0.M.19** | 152 | 39 | 713 | 400 | 30 | 152 | 19 | 36 | aacattcaacgctgtcggtg |
| **hsa-miR-181a-5p.3.P0.M.21** | 436 | 1.123 | 831 | 1.862 | 236 | 269 | 115 | 218 | aacattcaacgctgtcggtga |
| **hsa-miR-181a-5p.3.P0.M.23** | 1.242 | 2.052 | 4.388 | 5.016 | 187 | 276 | 258 | 305 | aacattcaacgctgtcggtgag |
| **hsa-miR-181a-5p.3.P0.M.24** | 3.950 | 3.485 | 8.387 | 6.164 | 354 | 331 | 861 | 552 | aacattcaacgctgtcggtgagtt |
| **hsa-miR-181a-5p.3.P0.M.27** | 2.655 | 2.401 | 5.742 | 2.349 | 197 | 186 | 820 | 387 | aacattcaacgctgtcggtgagttt |
| **hsa-miR-181a-5p.3.P0.M.4** | 13 | 39 | 81 | 1.575 | 30 | 0 | 70 | 6 | aacattcaacgctgtcggtgagtg |
| **hsa-miR-181b-5p.0.P1.M.30** | 7 | 0 | 1.345 | 9 | 0 | 0 | 0 | 3 | aacattcattgctgtcggtgggc |
| **hsa-miR-181b-5p.3.P0.M.1** | 86 | 39 | 1.237 | 200 | 0 | 0 | 6 | 10 | aacattcattgctgtcggtgggtc |
| **hsa-miR-181b-5p.3.P0.M.11** | 251 | 271 | 569 | 822 | 148 | 69 | 70 | 175 | aacattcattgctgtcggtgg |
| **hsa-miR-181b-5p.3.P0.M.13** | 410 | 503 | 1.183 | 1.288 | 59 | 14 | 29 | 116 | aacattcattgctgtcggtggg |
| **hsa-miR-181b-5p.3.P0.M.14** | 3.547 | 4.608 | 6.961 | 4.963 | 463 | 566 | 450 | 592 | aacattcattgctgtcggtgggtt |
| **hsa-miR-181b-5p.3.P0.M.2** | 951 | 736 | 2.528 | 1.449 | 138 | 69 | 169 | 77 | aacattcattgctgtcggtgggtg |
| **hsa-miR-181b-5p.3.P0.S.26** | 872 | 891 | 2.095 | 1.096 | 49 | 55 | 131 | 107 | aacattcattgctgtcggtgggttt |
| **hsa-miR-181d-5p.3.P0.S.12** | 264 | 194 | 433 | 579 | 217 | 304 | 16 | 0 | aacattcattgttgtcggtgggtt |
| **hsa-miR-182-5p.3.P0.S.12** | 581 | 426 | 271 | 561 | 285 | 387 | 491 | 375 | tttggcaatggtagaactcaca |
| **hsa-miR-182-5p.3.P0.S.13** | 991 | 929 | 379 | 457 | 374 | 269 | 730 | 347 | tttggcaatggtagaactcacactgg |
| **hsa-miR-182-5p.3.P0.S.17** | 1.638 | 1.355 | 605 | 1.401 | 797 | 704 | 2.057 | 893 | tttggcaatggtagaactcacactg |
| **hsa-miR-183-5p.3.P0.S.8** | 951 | 774 | 524 | 1.105 | 571 | 483 | 893 | 803 | tatggcactggtagaattcactg |
| **hsa-miR-183-5p.53.P0.S.13** | 918 | 503 | 894 | 1.457 | 778 | 1.146 | 877 | 823 | atggcactggtagaattcactg |
| **hsa-miR-185-5p.3.P0.S.7** | 251 | 348 | 235 | 313 | 650 | 677 | 163 | 242 | tggagagaaaggcagttcctg |
| **hsa-miR-186-5p.3.P0.S.14** | 2.206 | 1.433 | 451 | 1.127 | 1.919 | 1.650 | 797 | 821 | caaagaattctccttttgggctt |
| **hsa-miR-188-5p.3.P0.S.19** | 86 | 77 | 90 | 61 | 285 | 255 | 450 | 573 | catcccttgcatggtggagggt |
| **hsa-miR-18a-5p.3.P0.S.1** | 40 | 39 | 451 | 818 | 49 | 200 | 233 | 19 | taaggtgcatctagtgcagataga |
| **hsa-miR-18a-5p.3.P0.S.13** | 99 | 310 | 460 | 1.997 | 197 | 304 | 395 | 49 | taaggtgcatctagtgcagatagt |
| **hsa-miR-18a-5p.3.P0.S.4** | 634 | 426 | 5.164 | 8.870 | 2.648 | 2.251 | 577 | 257 | taaggtgcatctagtgcagat |
| **hsa-miR-18a-5p.3.P0.S.5** | 740 | 387 | 8.189 | 16.304 | 2.284 | 1.947 | 1.626 | 334 | taaggtgcatctagtgcagata |
| **hsa-miR-18a-5p.3.P0.S.6** | 291 | 77 | 1.905 | 2.645 | 925 | 545 | 252 | 123 | taaggtgcatctagtgcaga |
| **hsa-miR-18a-5p.5.P0.S.5** | 192 | 39 | 650 | 687 | 108 | 55 | 73 | 17 | aaggtgcatctagtgcagatag |
| **hsa-miR-18b-5p.3.P0.S.2** | 456 | 77 | 605 | 1.331 | 374 | 249 | 96 | 226 | taaggtgcatctagtgcagt |
| **hsa-miR-18b-5p.3.P1.S.0** | 192 | 77 | 271 | 818 | 226 | 214 | 22 | 146 | taaggtgcatctagtgcagta |
| **hsa-miR-191-5p.3.P0.S.10** | 443 | 503 | 172 | 361 | 846 | 932 | 201 | 341 | caacggaatcccaaaagcag |
| **hsa-miR-191-5p.3.P0.S.12** | 11.473 | 10.222 | 4.848 | 9.583 | 18.711 | 22.058 | 1.795 | 1.823 | caacggaatcccaaaagcagct |
| **hsa-miR-191-5p.3.P0.S.13** | 938 | 1.200 | 795 | 779 | 1.191 | 1.691 | 226 | 251 | caacggaatcccaaaagcagc |
| **hsa-miR-191-5p.3.P0.S.14** | 4.815 | 4.066 | 2.384 | 2.140 | 2.726 | 3.238 | 667 | 391 | caacggaatcccaaaagcagctgt |
| **hsa-miR-192-5p.3.P0.S.14** | 482 | 542 | 659 | 635 | 591 | 656 | 102 | 82 | ctgacctatgaattgacagcct |
| **hsa-miR-192-5p.3.P0.S.15** | 469 | 774 | 433 | 609 | 738 | 822 | 220 | 123 | ctgacctatgaattgacagccag |
| **hsa-miR-192-5p.3.P0.S.19** | 12.186 | 10.532 | 11.620 | 18.092 | 19.617 | 21.554 | 2.972 | 3.381 | ctgacctatgaattgacagcca |
| **hsa-miR-192-5p.3.P0.S.20** | 733 | 503 | 605 | 679 | 797 | 718 | 370 | 461 | ctgacctatgaattgacag |
| **hsa-miR-192-5p.3.P0.S.21** | 4.201 | 3.988 | 5.255 | 4.776 | 4.685 | 5.875 | 1.317 | 2.044 | ctgacctatgaattgacagc |
| **hsa-miR-192-5p.3.P0.S.8** | 581 | 929 | 1.526 | 1.823 | 1.211 | 1.077 | 561 | 248 | ctgacctatgaattgacagccat |
| **hsa-miR-192-5p.5.P0.S.1** | 1.816 | 1.665 | 1.968 | 2.045 | 1.437 | 1.830 | 399 | 579 | tgacctatgaattgacagcc |
| **hsa-miR-192-5p.53.P0.S.22** | 10.951 | 8.673 | 11.421 | 14.608 | 18.908 | 21.685 | 4.206 | 4.714 | tgacctatgaattgacagccag |
| **hsa-miR-192-5p.53.P0.S.26** | 522 | 310 | 1.201 | 1.605 | 1.289 | 1.629 | 702 | 317 | tgacctatgaattgacagccagt |
| **hsa-miR-192-5p.53.P0.S.27** | 5.059 | 5.227 | 5.146 | 7.178 | 7.097 | 8.651 | 1.336 | 1.905 | tgacctatgaattgacagcca |
| **hsa-miR-192-5p.53.P0.S.7** | 1.215 | 581 | 1.869 | 2.736 | 1.988 | 2.727 | 781 | 397 | tgacctatgaattgacagccaga |
| **hsa-miR-193b-3p.0.P1.S.34** | 205 | 77 | 36 | 213 | 571 | 545 | 29 | 29 | aactggccctcaaagtcccgca |
| **hsa-miR-193b-3p.3.P0.S.0** | 192 | 503 | 117 | 1.040 | 482 | 456 | 67 | 85 | aactggccctcaaagtcccgcta |
| **hsa-miR-193b-3p.3.P0.S.14** | 396 | 620 | 262 | 257 | 679 | 842 | 45 | 85 | aactggccctcaaagtcc |
| **hsa-miR-193b-3p.3.P0.S.15** | 806 | 852 | 307 | 840 | 2.234 | 3.072 | 214 | 464 | aactggccctcaaagtcccg |
| **hsa-miR-193b-3p.3.P0.S.17** | 641 | 1.433 | 352 | 809 | 2.441 | 2.340 | 188 | 226 | aactggccctcaaagtcccgc |
| **hsa-miR-193b-3p.3.P0.S.18** | 1.044 | 2.401 | 497 | 1.340 | 2.323 | 1.988 | 207 | 136 | aactggccctcaaagtcccgctt |
| **hsa-miR-193b-3p.5.P0.S.5** | 456 | 581 | 81 | 152 | 551 | 511 | 35 | 20 | actggccctcaaagtcccgct |
| **hsa-miR-194-5p.3.P0.M.3** | 317 | 232 | 713 | 1.048 | 935 | 1.257 | 255 | 361 | tgtaacagcaactccatgtgg |
| **hsa-miR-194-5p.3.P0.S.9** | 410 | 697 | 560 | 783 | 148 | 242 | 357 | 204 | tgtaacagcaactccatgtggaa |
| **hsa-miR-195-5p.3.P0.S.6** | 469 | 620 | 172 | 470 | 167 | 186 | 86 | 96 | tagcagcacagaaatattggca |
| **hsa-miR-196a-5p.3.P0.M.5** | 839 | 232 | 0 | 0 | 0 | 0 | 96 | 222 | taggtagtttcatgttgttgg |
| **hsa-miR-199a-3p.3.P0.M.3** | 225 | 0 | 217 | 70 | 1.093 | 1.194 | 41 | 90 | acagtagtctgcacattggtt |
| **hsa-miR-199b-5p.3.P0.S.2** | 73 | 116 | 54 | 70 | 768 | 511 | 73 | 106 | cccagtgtttagactatctgtt |
| **hsa-miR-19a-3p.3.P0.S.4** | 0 | 0 | 18 | 1.235 | 197 | 7 | 0 | 10 | tgtgcaaatctatgcaaa |
| **hsa-miR-19a-3p.3.P0.S.5** | 1.915 | 2.672 | 6.473 | 1.022 | 344 | 3.473 | 2.006 | 1.677 | tgtgcaaatctatgcaaaactg |
| **hsa-miR-19a-3p.3.P0.S.7** | 588 | 581 | 849 | 2.997 | 472 | 621 | 303 | 411 | tgtgcaaatctatgcaaaact |
| **hsa-miR-19b-3p.0.P1.M.50** | 1.017 | 852 | 1.860 | 6.473 | 561 | 746 | 1.225 | 664 | tgtgcaaatccatgcaaaactgt |
| **hsa-miR-19b-3p.3.P0.M.3** | 53 | 155 | 126 | 4.476 | 187 | 21 | 16 | 7 | tgtgcaaatccatgcaaa |
| **hsa-miR-19b-3p.3.P0.M.4** | 9.280 | 13.514 | 31.238 | 4.837 | 4.124 | 12.006 | 8.681 | 7.796 | tgtgcaaatccatgcaaaactg |
| **hsa-miR-19b-3p.3.P0.M.5** | 40 | 0 | 63 | 974 | 69 | 117 | 16 | 17 | tgtgcaaatccatgcaaaa |
| **hsa-miR-19b-3p.3.P0.M.6** | 1.598 | 1.626 | 4.072 | 8.417 | 2.087 | 1.850 | 756 | 1.126 | tgtgcaaatccatgcaaaact |
| **hsa-miR-19b-3p.3.P0.M.7** | 73 | 155 | 172 | 935 | 197 | 159 | 48 | 69 | tgtgcaaatccatgcaaaac |
| **hsa-miR-19b-3p.5.P0.M.8** | 997 | 774 | 1.463 | 1.318 | 364 | 532 | 182 | 254 | gtgcaaatccatgcaaaactga |
| **hsa-miR-200a-3p.3.P0.S.13** | 859 | 852 | 6.609 | 9.427 | 1.949 | 2.327 | 11.158 | 7.752 | taacactgtctggtaacgatgtt |
| **hsa-miR-200b-3p.3.P0.S.10** | 86 | 0 | 433 | 374 | 128 | 366 | 813 | 1.051 | taatactgcctggtaatgatg |
| **hsa-miR-200c-3p.3.P0.S.7** | 0 | 0 | 0 | 0 | 0 | 21 | 526 | 760 | taatactgccgggtaatgatg |
| **hsa-miR-20a-5p.0.P1.S.40** | 588 | 503 | 2.952 | 3.806 | 659 | 939 | 797 | 440 | taaagtgcttatagtgcaggtaa |
| **hsa-miR-20a-5p.0.P1.S.42** | 561 | 1.084 | 3.575 | 3.250 | 492 | 642 | 769 | 547 | taaagtgcttatagtgcaggtat |
| **hsa-miR-20a-5p.3.P0.S.11** | 2.133 | 2.168 | 8.839 | 7.769 | 3.268 | 4.715 | 823 | 1.086 | taaagtgcttatagtgcaggt |
| **hsa-miR-20a-5p.3.P0.S.13** | 568 | 620 | 3.341 | 2.066 | 492 | 587 | 230 | 272 | taaagtgcttatagtgcaggta |
| **hsa-miR-20a-5p.3.P0.S.15** | 238 | 310 | 1.120 | 526 | 394 | 435 | 108 | 206 | taaagtgcttatagtgcagg |
| **hsa-miR-20a-5p.3.P0.S.19** | 277 | 232 | 1.174 | 1.931 | 226 | 324 | 545 | 179 | taaagtgcttatagtgcaggtagt |
| **hsa-miR-20a-5p.3.P0.S.2** | 218 | 155 | 840 | 770 | 187 | 255 | 204 | 97 | taaagtgcttatagtgcaggtaga |
| **hsa-miR-20a-5p.3.P1.S.24** | 7 | 39 | 1.345 | 13 | 0 | 21 | 0 | 1 | taaagtgcttatagtgcaggc |
| **hsa-miR-20a-5p.5.P0.S.9** | 898 | 542 | 2.718 | 1.857 | 492 | 891 | 351 | 248 | aaagtgcttatagtgcaggtag |
| **hsa-miR-212-5p.53.P1.S.3** | 112 | 348 | 117 | 218 | 659 | 594 | 35 | 6 | gcgcgccggcgccttggc |
| **hsa-miR-215-5p.3.P0.S.6** | 1.790 | 852 | 63 | 204 | 30 | 0 | 0 | 1 | atgacctatgaattgacagaca |
| **hsa-miR-21-5p.0.P1.S.47** | 1.057 | 39 | 18 | 113 | 965 | 35 | 10 | 14 | tagcttatcagactgatgttgc |
| **hsa-miR-21-5p.3.P0.S.0** | 59 | 2.130 | 1.408 | 222 | 89 | 400 | 1.588 | 685 | tagcttatcagactgatgttgaa |
| **hsa-miR-21-5p.3.P0.S.19** | 5.541 | 581 | 957 | 539 | 8.868 | 2.347 | 887 | 546 | tagcttatcagactgatgttg |
| **hsa-miR-21-5p.3.P0.S.20** | 49.576 | 52.738 | 220.105 | 8.122 | 33.279 | 70.427 | 173.851 | 62.675 | tagcttatcagactgatgttgac |
| **hsa-miR-21-5p.3.P0.S.31** | 5.852 | 2.323 | 2.700 | 983 | 6.240 | 2.851 | 8.295 | 2.520 | tagcttatcagactgatgttgact |
| **hsa-miR-21-5p.3.P0.S.32** | 13.903 | 1.007 | 614 | 1.366 | 20.631 | 1.077 | 427 | 267 | tagcttatcagactgatgtt |
| **hsa-miR-21-5p.3.P0.S.33** | 4.868 | 116 | 695 | 83 | 3.593 | 131 | 61 | 46 | tagcttatcagactgatgt |
| **hsa-miR-21-5p.3.P0.S.34** | 12.285 | 3.795 | 1.706 | 3.445 | 26.664 | 4.039 | 1.381 | 1.308 | tagcttatcagactgatg |
| **hsa-miR-21-5p.3.P0.S.4** | 2.477 | 5.576 | 3.268 | 418 | 1.221 | 7.732 | 20.388 | 5.036 | tagcttatcagactgatgttgaca |
| **hsa-miR-21-5p.3.P0.S.5** | 112 | 155 | 1.101 | 39 | 79 | 394 | 2.114 | 401 | tagcttatcagactgatgttgacc |
| **hsa-miR-21-5p.3.P1.S.248** | 198 | 155 | 81 | 44 | 896 | 400 | 67 | 86 | tagcttatcagactgatc |
| **hsa-miR-21-5p.3.P1.S.87** | 1.162 | 0 | 9 | 13 | 463 | 0 | 0 | 1 | tagcttatcagactgatgtttac |
| **hsa-miR-21-5p.5.P0.S.3** | 687 | 1.278 | 533 | 270 | 1.024 | 3.321 | 2.105 | 1.422 | gtagcttatcagactgatgttga |
| **hsa-miR-21-5p.5.P0.S.7** | 727 | 1.123 | 623 | 322 | 925 | 3.342 | 883 | 995 | agcttatcagactgatgttga |
| **hsa-miR-21-5p.53.P0.S.13** | 258 | 194 | 451 | 74 | 344 | 469 | 957 | 490 | gtagcttatcagactgatgttgac |
| **hsa-miR-21-5p.53.P0.S.29** | 185 | 0 | 54 | 57 | 728 | 559 | 77 | 163 | agcttatcagactgatgttg |
| **hsa-miR-21-5p.53.P0.S.30** | 1.684 | 3.717 | 4.000 | 1.257 | 1.998 | 5.730 | 4.155 | 4.357 | agcttatcagactgatgttgac |
| **hsa-miR-221-3p.0.P1.S.25** | 1.361 | 0 | 758 | 827 | 532 | 366 | 472 | 566 | agctacattgtctgctgggtttt |
| **hsa-miR-221-3p.3.P0.S.10** | 1.281 | 0 | 605 | 544 | 787 | 828 | 513 | 1.019 | agctacattgtctgctgggtt |
| **hsa-miR-221-3p.3.P0.S.4** | 462 | 0 | 361 | 670 | 256 | 186 | 188 | 109 | agctacattgtctgctgggtttct |
| **hsa-miR-221-3p.3.P0.S.8** | 10.601 | 0 | 4.975 | 5.551 | 9.154 | 6.959 | 5.351 | 7.602 | agctacattgtctgctgggttt |
| **hsa-miR-222-3p.3.P0.S.25** | 515 | 774 | 397 | 418 | 709 | 704 | 332 | 269 | agctacatctggctactgggtc |
| **hsa-miR-222-3p.3.P0.S.26** | 806 | 1.123 | 244 | 927 | 896 | 718 | 466 | 272 | agctacatctggctactgggtctc |
| **hsa-miR-222-3p.3.P0.S.30** | 3.190 | 2.478 | 1.309 | 2.532 | 3.002 | 2.879 | 1.799 | 1.171 | agctacatctggctactgggtct |
| **hsa-miR-223-3p.3.P0.S.7** | 99 | 232 | 298 | 109 | 846 | 1.284 | 67 | 69 | tgtcagtttgtcaaataccccaa |
| **hsa-miR-223-3p.53.P0.S.9** | 99 | 116 | 226 | 57 | 600 | 746 | 10 | 37 | gtcagtttgtcaaataccccaa |
| **hsa-miR-22-5p.3.P0.S.3** | 1.929 | 1.433 | 415 | 405 | 453 | 670 | 6 | 843 | agttcttcagtggcaagcttt |
| **hsa-miR-23a-3p.3.P0.M.1** | 1.202 | 2.401 | 1.291 | 800 | 2.087 | 2.189 | 284 | 692 | atcacattgccagggatt |
| **hsa-miR-23a-3p.3.P0.S.10** | 4.709 | 4.569 | 1.824 | 3.506 | 1.329 | 1.284 | 695 | 371 | atcacattgccagggatttccat |
| **hsa-miR-23a-3p.3.P0.S.19** | 11.578 | 8.906 | 3.846 | 4.685 | 3.475 | 3.597 | 2.641 | 1.618 | atcacattgccagggatttccaa |
| **hsa-miR-23a-3p.3.P0.S.20** | 66.643 | 63.424 | 16.847 | 34.596 | 35.818 | 38.365 | 8.955 | 9.032 | atcacattgccagggatttcca |
| **hsa-miR-23a-3p.3.P0.S.21** | 5.806 | 8.131 | 1.598 | 2.166 | 3.721 | 4.066 | 673 | 1.284 | atcacattgccagggattt |
| **hsa-miR-23a-3p.3.P0.S.22** | 10.370 | 13.978 | 2.817 | 4.428 | 5.817 | 6.690 | 1.119 | 1.809 | atcacattgccagggatttc |
| **hsa-miR-23a-3p.3.P0.S.4** | 2.827 | 2.130 | 876 | 1.362 | 492 | 608 | 638 | 257 | atcacattgccagggatttccaat |
| **hsa-miR-23a-3p.53.P0.S.18** | 720 | 891 | 18 | 170 | 226 | 283 | 29 | 57 | tcacattgccagggatttcca |
| **hsa-miR-23b-3p.3.P0.S.15** | 588 | 1.162 | 1.264 | 3.476 | 1.191 | 1.208 | 635 | 368 | atcacattgccagggattaccac |
| **hsa-miR-23b-3p.3.P0.S.16** | 5.991 | 9.099 | 7.024 | 19.845 | 6.093 | 6.510 | 2.669 | 2.124 | atcacattgccagggattacca |
| **hsa-miR-23b-3p.3.P0.S.17** | 1.255 | 2.130 | 1.336 | 1.701 | 1.033 | 960 | 399 | 665 | atcacattgccagggatta |
| **hsa-miR-23b-3p.3.P0.S.18** | 1.869 | 2.556 | 876 | 4.115 | 2.028 | 1.885 | 848 | 1.135 | atcacattgccagggattac |
| **hsa-miR-23b-3p.3.P0.S.6** | 4.888 | 6.311 | 2.907 | 6.751 | 1.900 | 1.816 | 1.492 | 639 | atcacattgccagggattaccact |
| **hsa-miR-23b-3p.3.P0.S.9** | 839 | 1.045 | 921 | 1.918 | 463 | 545 | 501 | 300 | atcacattgccagggattaccat |
| **hsa-miR-24-3p.3.P0.M.0** | 2.305 | 658 | 334 | 1.240 | 482 | 476 | 249 | 24 | tggctcagttcagcaggaacaga |
| **hsa-miR-24-3p.3.P0.M.3** | 97.488 | 78.719 | 2.546 | 47.425 | 32.560 | 30.108 | 10.942 | 5.565 | tggctcagttcagcaggaacagt |
| **hsa-miR-24-3p.3.P0.M.4** | 654 | 968 | 921 | 361 | 974 | 953 | 137 | 257 | tggctcagttcagcagga |
| **hsa-miR-24-3p.3.P0.M.5** | 2.061 | 1.626 | 370 | 2.114 | 1.595 | 1.836 | 341 | 449 | tggctcagttcagcaggaac |
| **hsa-miR-24-3p.3.P0.M.6** | 687 | 1.007 | 253 | 700 | 738 | 808 | 163 | 272 | tggctcagttcagcaggaa |
| **hsa-miR-24-3p.3.P0.M.7** | 3.956 | 2.749 | 54 | 4.911 | 3.455 | 3.238 | 622 | 312 | tggctcagttcagcaggaaca |
| **hsa-miR-24-3p.3.P1.M.29** | 376 | 620 | 117 | 291 | 610 | 587 | 105 | 216 | tggctcagttcagcaggaaa |
| **hsa-miR-24-3p.53.P0.M.12** | 2.979 | 2.246 | 316 | 1.083 | 984 | 877 | 233 | 188 | ggctcagttcagcaggaacagt |
| **hsa-miR-25-3p.0.P1.S.32** | 753 | 542 | 497 | 200 | 581 | 469 | 191 | 224 | cattgcacttgtctcggtctgt |
| **hsa-miR-25-3p.3.P0.S.4** | 2.259 | 2.168 | 885 | 1.414 | 2.490 | 2.044 | 510 | 774 | cattgcacttgtctcggtctg |
| **hsa-miR-25-3p.3.P0.S.8** | 687 | 1.045 | 479 | 439 | 1.132 | 1.250 | 255 | 371 | cattgcacttgtctcggtct |
| **hsa-miR-26a-5p.0.P1.M.42** | 555 | 620 | 343 | 357 | 295 | 435 | 182 | 152 | ttcaagtaatccaggataggca |
| **hsa-miR-26a-5p.0.P1.M.43** | 165 | 194 | 3.593 | 57 | 69 | 62 | 22 | 19 | ttcaagtaatccaggataggcc |
| **hsa-miR-26a-5p.3.P0.M.0** | 469 | 581 | 533 | 1.101 | 413 | 559 | 195 | 202 | ttcaagtaatccaggataggcta |
| **hsa-miR-26a-5p.3.P0.M.12** | 1.387 | 697 | 975 | 748 | 354 | 428 | 172 | 318 | ttcaagtaatccaggataggc |
| **hsa-miR-26a-5p.3.P0.M.5** | 892 | 1.007 | 578 | 1.275 | 482 | 539 | 252 | 181 | ttcaagtaatccaggataggctt |
| **hsa-miR-26a-5p.3.P0.M.8** | 522 | 426 | 325 | 204 | 463 | 1.001 | 258 | 434 | ttcaagtaatccaggatag |
| **hsa-miR-26a-5p.5.P0.M.3** | 1.195 | 968 | 433 | 840 | 502 | 725 | 134 | 209 | tcaagtaatccaggataggct |
| **hsa-miR-26b-5p.3.P0.S.16** | 3.573 | 2.788 | 3.684 | 3.763 | 3.612 | 4.667 | 459 | 755 | ttcaagtaattcaggataggtt |
| **hsa-miR-26b-5p.3.P0.S.9** | 416 | 503 | 469 | 539 | 374 | 373 | 96 | 118 | ttcaagtaattcaggataggttt |
| **hsa-miR-27a-3p.0.P1.S.5** | 2.021 | 1.665 | 659 | 896 | 935 | 1.215 | 144 | 214 | ttcacagtggctaagttccga |
| **hsa-miR-27a-3p.0.P1.S.7** | 4.716 | 2.362 | 1.363 | 1.588 | 2.471 | 2.734 | 549 | 922 | ttcacagtggctaagttccgt |
| **hsa-miR-27a-3p.3.P0.M.2** | 885 | 2.788 | 722 | 522 | 1.033 | 1.360 | 198 | 340 | ttcacagtggctaagttc |
| **hsa-miR-27a-3p.3.P0.S.8** | 3.712 | 5.886 | 1.255 | 1.892 | 3.386 | 3.024 | 702 | 1.147 | ttcacagtggctaagttccg |
| **hsa-miR-27b-3p.3.P0.S.10** | 694 | 1.007 | 713 | 1.005 | 1.211 | 1.174 | 236 | 552 | ttcacagtggctaagttct |
| **hsa-miR-27b-3p.3.P0.S.8** | 588 | 813 | 551 | 831 | 354 | 173 | 153 | 212 | ttcacagtggctaagttctgca |
| **hsa-miR-27b-3p.3.P0.S.9** | 3.983 | 3.988 | 2.537 | 4.050 | 3.789 | 3.473 | 899 | 1.794 | ttcacagtggctaagttctg |
| **hsa-miR-28-3p.3.P0.S.4** | 324 | 194 | 280 | 287 | 591 | 594 | 201 | 357 | cactagattgtgagctcctgg |
| **hsa-miR-29a-3p.3.P0.S.0** | 462 | 891 | 108 | 183 | 915 | 649 | 1.920 | 1.108 | tagcaccatctgaaatcggttaa |
| **hsa-miR-29a-3p.3.P0.S.3** | 7.998 | 9.022 | 1.065 | 2.662 | 35.316 | 14.547 | 7.835 | 12.453 | tagcaccatctgaaatcggtt |
| **hsa-miR-29a-3p.3.P0.S.5** | 410 | 465 | 181 | 161 | 797 | 835 | 214 | 427 | tagcaccatctgaaatcggt |
| **hsa-miR-29a-3p.3.P0.S.6** | 231 | 232 | 163 | 104 | 541 | 345 | 1.014 | 566 | tagcaccatctgaaatcggttat |
| **hsa-miR-29a-3p.5.P0.S.0** | 363 | 155 | 163 | 135 | 600 | 532 | 1.126 | 530 | ctagcaccatctgaaatcggtta |
| **hsa-miR-29a-3p.5.P0.S.4** | 462 | 116 | 153 | 152 | 719 | 670 | 523 | 536 | agcaccatctgaaatcggtta |
| **hsa-miR-29a-3p.53.P0.S.13** | 277 | 77 | 45 | 83 | 768 | 338 | 150 | 294 | agcaccatctgaaatcggtt |
| **hsa-miR-29a-3p.53.P0.S.5** | 885 | 503 | 108 | 413 | 3.012 | 2.037 | 1.601 | 1.198 | ctagcaccatctgaaatcggtt |
| **hsa-miR-29b-3p.3.P0.M.4** | 73 | 348 | 9 | 83 | 561 | 614 | 150 | 267 | tagcaccatttgaaatcagt |
| **hsa-miR-29b-3p.3.P0.M.5** | 13 | 0 | 27 | 9 | 679 | 601 | 83 | 159 | tagcaccatttgaaatcag |
| **hsa-miR-29b-3p.3.P0.M.6** | 1.301 | 1.626 | 1.029 | 970 | 6.811 | 6.635 | 3.757 | 3.372 | tagcaccatttgaaatcagtgt |
| **hsa-miR-29b-3p.5.P0.M.7** | 258 | 542 | 199 | 183 | 571 | 663 | 682 | 772 | agcaccatttgaaatcagtgtt |
| **hsa-miR-29b-3p.53.P0.M.3** | 7 | 0 | 280 | 509 | 797 | 635 | 322 | 179 | ctagcaccatttgaaatcagtgt |
| **hsa-miR-29c-3p.3.P0.S.4** | 192 | 194 | 343 | 631 | 2.608 | 1.222 | 615 | 854 | tagcaccatttgaaatcggtt |
| **hsa-miR-301a-3p.3.P0.S.14** | 343 | 232 | 930 | 1.762 | 856 | 635 | 281 | 80 | cagtgcaatagtattgtcaaagca |
| **hsa-miR-301a-3p.3.P0.S.17** | 951 | 774 | 5.002 | 7.513 | 3.071 | 2.768 | 1.464 | 413 | cagtgcaatagtattgtcaaagcat |
| **hsa-miR-301a-3p.3.P0.S.18** | 429 | 426 | 1.147 | 1.697 | 2.815 | 2.734 | 714 | 513 | cagtgcaatagtattgtcaaag |
| **hsa-miR-301a-3p.3.P0.S.6** | 641 | 155 | 1.697 | 1.836 | 709 | 587 | 262 | 166 | cagtgcaatagtattgtcaaagcatt |
| **hsa-miR-302c-5p.53.P0.S.0** | 0 | 2.130 | 0 | 0 | 0 | 0 | 0 | 1 | tgctgggtgaaacaaaag |
| **hsa-miR-30a-5p.3.P0.S.13** | 766 | 774 | 1.454 | 2.610 | 197 | 97 | 453 | 353 | tgtaaacatcctcgactggaagc |
| **hsa-miR-30a-5p.3.P0.S.15** | 1.757 | 1.781 | 2.943 | 3.184 | 167 | 173 | 619 | 247 | tgtaaacatcctcgactggaagct |
| **hsa-miR-30b-5p.3.P0.S.13** | 443 | 813 | 307 | 248 | 177 | 297 | 83 | 77 | tgtaaacatcctacactcagc |
| **hsa-miR-30c-5p.3.P0.M.15** | 5.139 | 5.731 | 3.584 | 7.700 | 5.463 | 6.013 | 5.970 | 3.160 | tgtaaacatcctacactctcagct |
| **hsa-miR-30d-5p.3.P0.S.16** | 5.383 | 6.273 | 1.129 | 4.985 | 3.199 | 3.383 | 2.124 | 1.078 | tgtaaacatccccgactggaagc |
| **hsa-miR-30d-5p.3.P0.S.20** | 11.823 | 11.345 | 3.666 | 5.581 | 4.774 | 4.791 | 2.570 | 1.356 | tgtaaacatccccgactggaagct |
| **hsa-miR-30e-3p.0.P1.S.6** | 337 | 194 | 433 | 518 | 630 | 787 | 262 | 332 | ctttcagtcggatgtttacagt |
| **hsa-miR-30e-5p.3.P0.S.17** | 1.664 | 2.052 | 1.309 | 2.623 | 2.943 | 3.362 | 2.567 | 1.819 | tgtaaacatccttgactggaagc |
| **hsa-miR-30e-5p.3.P0.S.20** | 4.689 | 4.221 | 2.095 | 2.728 | 3.524 | 3.873 | 3.186 | 1.763 | tgtaaacatccttgactggaagct |
| **hsa-miR-30e-5p.5.P0.S.0** | 297 | 3.446 | 704 | 291 | 1.801 | 1.484 | 274 | 493 | taaacatccttgactggaag |
| **hsa-miR-30e-5p.5.P0.S.1** | 403 | 10.106 | 2.113 | 631 | 5.000 | 4.874 | 520 | 1.576 | aacatccttgactggaag |
| **hsa-miR-30e-5p.5.P0.S.2** | 700 | 7.473 | 1.544 | 779 | 4.144 | 3.631 | 453 | 1.324 | aaacatccttgactggaag |
| **hsa-miR-30e-5p.5.P0.S.3** | 654 | 3.678 | 867 | 396 | 1.663 | 1.581 | 313 | 516 | gtaaacatccttgactggaag |
| **hsa-miR-30e-5p.53.P0.S.19** | 964 | 852 | 388 | 805 | 1.555 | 1.588 | 753 | 639 | gtaaacatccttgactggaagct |
| **hsa-miR-30e-5p.53.P0.S.36** | 429 | 11.849 | 1.878 | 461 | 4.823 | 7.228 | 523 | 982 | acatccttgactggaagc |
| **hsa-miR-30e-5p.53.P0.S.40** | 159 | 3.291 | 488 | 200 | 1.417 | 3.486 | 210 | 259 | catccttgactggaagcg |
| **hsa-miR-31-5p.3.P0.S.12** | 40 | 0 | 0 | 9 | 59 | 21 | 590 | 605 | aggcaagatgctggcatagctg |
| **hsa-miR-320a.0.P1.S.37** | 1.394 | 697 | 307 | 318 | 1.368 | 877 | 277 | 172 | aaaagctgggttgagagggcgt |
| **hsa-miR-320a.3.P0.M.3** | 317 | 581 | 298 | 96 | 512 | 815 | 102 | 311 | aaaagctgggttgagaggg |
| **hsa-miR-320a.3.P0.S.19** | 1.030 | 194 | 190 | 48 | 423 | 83 | 159 | 145 | aaaagctgggttgagagggcgat |
| **hsa-miR-320a.3.P0.S.23** | 3.368 | 697 | 217 | 974 | 3.041 | 1.422 | 1.492 | 1.000 | aaaagctgggttgagagggcgaaa |
| **hsa-miR-320a.3.P0.S.24** | 1.123 | 542 | 226 | 170 | 797 | 877 | 175 | 193 | aaaagctgggttgagagggcg |
| **hsa-miR-320a.3.P0.S.25** | 9.531 | 3.446 | 506 | 2.540 | 7.264 | 2.817 | 1.891 | 2.456 | aaaagctgggttgagagggcgaa |
| **hsa-miR-320a.5.P0.S.5** | 581 | 503 | 153 | 252 | 610 | 539 | 70 | 142 | aagctgggttgagagggcga |
| **hsa-miR-320a.5.P0.S.6** | 3.375 | 1.317 | 442 | 700 | 2.500 | 2.264 | 389 | 639 | aaagctgggttgagagggcga |
| **hsa-miR-320a.53.P0.S.22** | 416 | 426 | 45 | 231 | 591 | 456 | 144 | 149 | aaagctgggttgagagggcgaaa |
| **hsa-miR-320a.53.P0.S.24** | 1.004 | 813 | 72 | 387 | 1.408 | 925 | 217 | 367 | aaagctgggttgagagggcgaa |
| **hsa-miR-320a.53.P0.S.42** | 522 | 387 | 63 | 287 | 817 | 469 | 115 | 202 | aagctgggttgagagggcgaaa |
| **hsa-miR-320a.53.P0.S.44** | 581 | 232 | 99 | 170 | 620 | 545 | 137 | 171 | aagctgggttgagagggcgaa |
| **hsa-miR-326.3.P0.S.4** | 165 | 465 | 45 | 117 | 876 | 608 | 287 | 294 | cctctgggcccttcctccagt |
| **hsa-miR-331-3p.3.P0.S.6** | 581 | 426 | 433 | 1.005 | 305 | 235 | 249 | 272 | gcccctgggcctatcctagaat |
| **hsa-miR-331-3p.3.P0.S.8** | 931 | 1.007 | 776 | 2.601 | 699 | 822 | 293 | 430 | gcccctgggcctatcctaga |
| **hsa-miR-335-5p.3.P0.S.4** | 1.295 | 774 | 27 | 35 | 59 | 62 | 0 | 4 | tcaagagcaataacgaaaaatg |
| **hsa-miR-338-3p.3.P0.S.6** | 20 | 116 | 325 | 605 | 669 | 753 | 195 | 196 | tccagcatcagtgattttgttga |
| **hsa-miR-339-5p.3.P0.S.5** | 310 | 542 | 226 | 326 | 581 | 490 | 239 | 244 | tccctgtcctccaggagctca |
| **hsa-miR-34a-3p.3.P0.S.9** | 92 | 39 | 54 | 65 | 659 | 587 | 427 | 519 | caatcagcaagtatactgcccta |
| **hsa-miR-34a-3p.53.P0.S.10** | 40 | 465 | 117 | 52 | 502 | 428 | 638 | 1.129 | aatcagcaagtatactgcccta |
| **hsa-miR-34a-5p.3.P0.S.15** | 482 | 155 | 451 | 318 | 2.451 | 1.809 | 1.671 | 2.581 | tggcagtgtcttagctggttg |
| **hsa-miR-34a-5p.3.P0.S.16** | 1.341 | 1.084 | 1.192 | 2.610 | 2.776 | 3.148 | 7.080 | 5.756 | tggcagtgtcttagctggttgtt |
| **hsa-miR-34a-5p.3.P0.S.8** | 86 | 155 | 99 | 170 | 148 | 124 | 682 | 364 | tggcagtgtcttagctggttgttt |
| **hsa-miR-365a-3p.3.P0.M.3** | 244 | 929 | 99 | 470 | 472 | 435 | 32 | 99 | taatgcccctaaaaatcctta |
| **hsa-miR-371a-3p.0.P1.S.47** | 0 | 0 | 0 | 0 | 0 | 0 | 1.630 | 903 | aagtgccgccatcttttgagtga |
| **hsa-miR-371a-3p.3.P0.S.6** | 0 | 39 | 0 | 0 | 0 | 0 | 1.225 | 1.710 | aagtgccgccatcttttgagt |
| **hsa-miR-371a-3p.3.P0.S.9** | 0 | 0 | 0 | 0 | 0 | 0 | 2.232 | 2.047 | aagtgccgccatcttttgagtg |
| **hsa-miR-371a-3p.5.P0.S.7** | 26 | 542 | 36 | 13 | 108 | 131 | 26.641 | 43.006 | gtgccgccatcttttgagtgt |
| **hsa-miR-371a-3p.5.P0.S.9** | 46 | 697 | 9 | 17 | 335 | 180 | 52.004 | 61.437 | agtgccgccatcttttgagtgt |
| **hsa-miR-371a-3p.5.P1.S.102** | 7 | 0 | 0 | 0 | 0 | 0 | 622 | 636 | agtgccgccatcttttgagtga |
| **hsa-miR-371a-3p.53.P0.S.14** | 0 | 0 | 0 | 0 | 0 | 0 | 839 | 861 | agtgccgccatcttttgagtg |
| **hsa-miR-371a-5p.0.P1.S.33** | 0 | 0 | 0 | 0 | 0 | 0 | 520 | 1.129 | actcaaactgtgggggcaca |
| **hsa-miR-371a-5p.3.P0.S.0** | 0 | 0 | 0 | 0 | 0 | 0 | 1.451 | 2.363 | actcaaactgtgggggcacta |
| **hsa-miR-371a-5p.3.P0.S.10** | 13 | 116 | 0 | 4 | 49 | 69 | 9.838 | 13.111 | actcaaactgtgggggcactta |
| **hsa-miR-371a-5p.3.P0.S.15** | 0 | 0 | 0 | 0 | 0 | 0 | 727 | 963 | actcaaactgtgggggcacttg |
| **hsa-miR-371a-5p.3.P0.S.16** | 0 | 0 | 9 | 0 | 20 | 14 | 5.189 | 4.268 | actcaaactgtgggggcacttta |
| **hsa-miR-371a-5p.3.P0.S.17** | 13 | 0 | 9 | 0 | 39 | 0 | 647 | 408 | actcaaactgtgggggcactttat |
| **hsa-miR-371a-5p.3.P0.S.2** | 0 | 0 | 0 | 0 | 20 | 7 | 1.186 | 1.096 | actcaaactgtgggggcactat |
| **hsa-miR-371a-5p.3.P0.S.25** | 0 | 0 | 9 | 0 | 20 | 7 | 4.717 | 4.173 | actcaaactgtgggggcactttt |
| **hsa-miR-371a-5p.3.P0.S.27** | 0 | 39 | 0 | 0 | 30 | 14 | 2.200 | 2.623 | actcaaactgtgggggca |
| **hsa-miR-371a-5p.3.P0.S.28** | 0 | 77 | 0 | 0 | 30 | 14 | 5.361 | 13.996 | actcaaactgtgggggcac |
| **hsa-miR-371a-5p.3.P0.S.29** | 132 | 774 | 63 | 22 | 709 | 324 | 71.361 | 154.488 | actcaaactgtgggggcactt |
| **hsa-miR-371a-5p.3.P0.S.30** | 7 | 0 | 18 | 0 | 20 | 55 | 8.154 | 7.308 | actcaaactgtgggggcactttc |
| **hsa-miR-371a-5p.3.P0.S.33** | 112 | 774 | 54 | 13 | 463 | 359 | 90.327 | 115.893 | actcaaactgtgggggcacttt |
| **hsa-miR-371a-5p.3.P1.S.83** | 0 | 39 | 0 | 0 | 20 | 7 | 660 | 2.267 | actcaaactgtgggggcaa |
| **hsa-miR-371a-5p.3.P1.S.87** | 0 | 77 | 0 | 0 | 0 | 14 | 960 | 2.808 | actcaaactgtgggggcat |
| **hsa-miR-371a-5p.53.P0.S.32** | 0 | 0 | 0 | 0 | 0 | 0 | 600 | 1.761 | ctcaaactgtgggggcactt |
| **hsa-miR-371a-5p.53.P0.S.33** | 7 | 0 | 0 | 0 | 0 | 28 | 692 | 1.340 | ctcaaactgtgggggcactttc |
| **hsa-miR-371a-5p.53.P0.S.35** | 0 | 77 | 9 | 4 | 39 | 21 | 3.463 | 6.537 | ctcaaactgtgggggcacttt |
| **hsa-miR-372-3p.0.P1.S.42** | 0 | 0 | 0 | 0 | 0 | 0 | 848 | 420 | aaagtgctgcgacatttgagcga |
| **hsa-miR-372-3p.3.P0.S.12** | 0 | 0 | 0 | 0 | 0 | 0 | 651 | 424 | aaagtgctgcgacatttgag |
| **hsa-miR-372-3p.3.P0.S.13** | 0 | 39 | 0 | 0 | 10 | 0 | 1.059 | 317 | aaagtgctgcgacatttgagcgtc |
| **hsa-miR-372-3p.3.P0.S.14** | 0 | 0 | 0 | 0 | 0 | 0 | 676 | 694 | aaagtgctgcgacatttga |
| **hsa-miR-372-3p.3.P0.S.16** | 0 | 0 | 0 | 0 | 0 | 0 | 998 | 497 | aaagtgctgcgacatttgagcg |
| **hsa-miR-372-3p.5.P0.S.8** | 0 | 39 | 0 | 0 | 0 | 7 | 7.322 | 6.981 | aagtgctgcgacatttgagcgt |
| **hsa-miR-372-5p.3.P0.S.2** | 0 | 0 | 0 | 0 | 0 | 0 | 756 | 884 | cctcaaatgtggagcactatt |
| **hsa-miR-373-3p.0.P1.S.63** | 7 | 77 | 0 | 0 | 69 | 48 | 11.841 | 8.345 | gaagtgcttcgattttggggtga |
| **hsa-miR-373-3p.0.P1.S.65** | 0 | 0 | 0 | 0 | 0 | 0 | 354 | 676 | gaagtgcttcgattttggggtgg |
| **hsa-miR-373-3p.3.P0.S.12** | 0 | 0 | 0 | 4 | 0 | 0 | 491 | 995 | gaagtgcttcgattttgg |
| **hsa-miR-373-3p.3.P0.S.13** | 0 | 39 | 0 | 0 | 20 | 7 | 1.572 | 1.794 | gaagtgcttcgattttggg |
| **hsa-miR-373-3p.3.P0.S.14** | 20 | 39 | 0 | 0 | 59 | 55 | 12.651 | 17.741 | gaagtgcttcgattttggggt |
| **hsa-miR-373-3p.3.P0.S.15** | 0 | 39 | 0 | 0 | 10 | 0 | 1.655 | 1.750 | gaagtgcttcgattttgggg |
| **hsa-miR-373-3p.3.P0.S.16** | 0 | 39 | 18 | 4 | 118 | 48 | 12.495 | 5.919 | gaagtgcttcgattttggggtg |
| **hsa-miR-373-3p.3.P0.S.8** | 0 | 0 | 0 | 0 | 20 | 14 | 2.226 | 957 | gaagtgcttcgattttggggtgtt |
| **hsa-miR-373-3p.3.P1.S.113** | 7 | 39 | 0 | 0 | 39 | 0 | 539 | 910 | gaagtgcttcgattttggga |
| **hsa-miR-373-3p.3.P1.S.87** | 0 | 0 | 0 | 0 | 30 | 0 | 1.256 | 1.701 | gaagtgcttcgattttgggga |
| **hsa-miR-373-3p.5.P0.S.7** | 0 | 0 | 9 | 4 | 0 | 0 | 2.653 | 3.113 | aagtgcttcgattttggggtgt |
| **hsa-miR-374b-5p.3.P0.S.0** | 793 | 581 | 298 | 365 | 689 | 725 | 466 | 378 | atataatacaacctgctaagtga |
| **hsa-miR-376b-3p.3.P0.S.1** | 139 | 0 | 81 | 0 | 482 | 545 | 35 | 19 | atcatagaggaaaatccatgt |
| **hsa-miR-378a-3p.3.P0.M.1** | 59 | 39 | 45 | 13 | 532 | 580 | 446 | 580 | actggacttggagtcagaag |
| **hsa-miR-378a-3p.3.P0.S.4** | 396 | 232 | 108 | 100 | 1.624 | 1.988 | 1.151 | 1.435 | actggacttggagtcagaagg |
| **hsa-miR-421.3.P0.S.6** | 522 | 39 | 515 | 557 | 876 | 925 | 794 | 572 | atcaacagacattaattgggcg |
| **hsa-miR-423-3p.3.P0.S.10** | 522 | 1.084 | 262 | 422 | 433 | 414 | 163 | 146 | agctcggtctgaggcccctca |
| **hsa-miR-423-5p.3.P0.S.9** | 561 | 968 | 289 | 448 | 738 | 670 | 179 | 254 | tgaggggcagagagcgagactt |
| **hsa-miR-424-5p.3.P0.S.7** | 13.210 | 9.912 | 2.194 | 2.536 | 1.467 | 1.940 | 2.261 | 3.080 | cagcagcaattcatgttttga |
| **hsa-miR-425-5p.3.P0.S.5** | 779 | 736 | 840 | 1.140 | 3.730 | 3.797 | 682 | 841 | aatgacacgatcactcccgttg |
| **hsa-miR-4301.5.P1.S.0** | 5.759 | 39 | 0 | 57 | 0 | 14 | 6 | 1 | ctcccactgcttcacttgtga |
| **hsa-miR-4301.53.P1.S.4** | 16.737 | 37.830 | 3.991 | 31.786 | 2.185 | 1.802 | 3.891 | 1.975 | ctcccactgcttcacttg |
| **hsa-miR-4497.53.P1.S.2** | 0 | 39 | 9 | 0 | 1.329 | 0 | 0 | 0 | cgggcgccggcggccggg |
| **hsa-miR-451a.3.P0.S.23** | 1.063 | 1.162 | 1.625 | 774 | 6.221 | 7.525 | 564 | 358 | aaaccgttaccattactgagtttag |
| **hsa-miR-451a.3.P0.S.28** | 680 | 194 | 677 | 265 | 1.447 | 1.912 | 64 | 47 | aaaccgttaccattactgagtttagt |
| **hsa-miR-451a.3.P0.S.29** | 667 | 1.123 | 1.661 | 383 | 3.849 | 4.646 | 561 | 360 | aaaccgttaccattactgagttta |
| **hsa-miR-451a.3.P0.S.30** | 482 | 620 | 560 | 104 | 1.486 | 3.190 | 163 | 82 | aaaccgttaccattactgagt |
| **hsa-miR-451a.3.P0.S.31** | 5.931 | 6.892 | 14.337 | 4.107 | 46.261 | 48.811 | 3.020 | 3.361 | aaaccgttaccattactgagttt |
| **hsa-miR-451a.3.P0.S.32** | 125 | 348 | 117 | 52 | 502 | 1.257 | 73 | 33 | aaaccgttaccattactgag |
| **hsa-miR-4532.5.P0.S.0** | 139 | 620 | 126 | 418 | 699 | 677 | 67 | 83 | cccccggggagcccggcg |
| **hsa-miR-455-3p.3.P0.S.10** | 528 | 620 | 524 | 1.657 | 1.240 | 1.422 | 389 | 492 | gcagtccatgggcatataca |
| **hsa-miR-455-3p.3.P0.S.11** | 1.083 | 1.084 | 325 | 740 | 699 | 960 | 262 | 182 | gcagtccatgggcatatacact |
| **hsa-miR-455-3p.53.P0.S.7** | 291 | 542 | 262 | 822 | 413 | 442 | 313 | 258 | atgcagtccatgggcatataca |
| **hsa-miR-4792.0.P1.S.1** | 509 | 3.640 | 1.851 | 722 | 2.185 | 2.030 | 48 | 24 | cggtgagctctcgctggc |
| **hsa-miR-4792.5.P1.S.1** | 291 | 1.200 | 813 | 470 | 846 | 587 | 6 | 3 | ccggtgagctctcgctggc |
| **hsa-miR-4792.53.P1.S.2** | 192 | 1.084 | 433 | 244 | 856 | 711 | 10 | 13 | ccggtgagctctcgctgg |
| **hsa-miR-4792.53.P1.S.4** | 343 | 1.355 | 1.020 | 548 | 965 | 1.098 | 6 | 20 | ggtgagctctcgctggcc |
| **hsa-miR-483-5p.3.P0.S.10** | 291 | 542 | 1.219 | 39 | 1.683 | 918 | 6 | 0 | aagacgggaggaaagaaggga |
| **hsa-miR-484.3.P0.S.17** | 456 | 581 | 533 | 613 | 1.545 | 1.864 | 376 | 486 | tcaggctcagtcccctcccga |
| **hsa-miR-501-5p.3.P0.S.22** | 106 | 77 | 54 | 126 | 207 | 83 | 753 | 447 | aatcctttgtccctgggtgagagt |
| **hsa-miR-532-5p.53.P1.S.5** | 575 | 1.084 | 5.914 | 796 | 413 | 83 | 89 | 133 | agtggaggaccgttggcat |
| **hsa-miR-532-5p.53.P1.S.8** | 535 | 891 | 2.772 | 418 | 335 | 193 | 77 | 106 | gtggaggaccgttggcat |
| **hsa-miR-551a.3.P0.S.3** | 0 | 0 | 0 | 0 | 492 | 511 | 32 | 32 | gcgacccactcttggtttccat |
| **hsa-miR-551a.3.P0.S.4** | 26 | 0 | 0 | 4 | 1.457 | 1.408 | 137 | 204 | gcgacccactcttggtttcc |
| **hsa-miR-551b-3p.3.P0.S.2** | 1.057 | 697 | 1.138 | 2.819 | 20 | 0 | 0 | 0 | gcgacccatacttggtttcagt |
| **hsa-miR-574-3p.3.P0.S.6** | 469 | 658 | 307 | 687 | 1.014 | 1.139 | 169 | 169 | cacgctcatgcacacacccac |
| **hsa-miR-574-3p.3.P0.S.8** | 535 | 813 | 244 | 400 | 591 | 711 | 147 | 215 | cacgctcatgcacacaccca |
| **hsa-miR-574-5p.3.P0.S.3** | 482 | 271 | 199 | 239 | 630 | 773 | 223 | 440 | tgagtgtgtgtgtgtgagtgt |
| **hsa-miR-574-5p.3.P0.S.4** | 561 | 348 | 307 | 157 | 630 | 635 | 64 | 120 | tgagtgtgtgtgtgtgagtg |
| **hsa-miR-596.53.P1.S.12** | 53 | 194 | 27 | 65 | 472 | 532 | 22 | 4 | tccgaagcctgcccggcg |
| **hsa-miR-660-5p.3.P0.S.11** | 2.589 | 2.478 | 1.110 | 1.701 | 4.921 | 4.805 | 11.560 | 8.625 | tacccattgcatatcggagttgt |
| **hsa-miR-7-5p.3.P0.M.1** | 396 | 310 | 45 | 113 | 167 | 283 | 1.225 | 800 | tggaagactagtgattttgttgtc |
| **hsa-miR-7-5p.3.P0.M.9** | 667 | 1.045 | 226 | 135 | 738 | 338 | 1.725 | 1.498 | tggaagactagtgattttgttgtt |
| **hsa-miR-92a-3p.0.P1.M.53** | 555 | 813 | 1.923 | 3.450 | 1.437 | 1.892 | 800 | 1.191 | tattgcacttgtcccggcctga |
| **hsa-miR-92a-3p.0.P1.M.54** | 172 | 387 | 23.591 | 613 | 325 | 511 | 112 | 287 | tattgcacttgtcccggcctgc |
| **hsa-miR-92a-3p.0.P1.M.55** | 178 | 77 | 632 | 848 | 394 | 414 | 80 | 320 | tattgcacttgtcccggcctgg |
| **hsa-miR-92a-3p.3.P0.M.4** | 258 | 1.084 | 1.300 | 3.315 | 827 | 808 | 281 | 292 | tattgcacttgtcccggc |
| **hsa-miR-92a-3p.3.P0.M.5** | 310 | 271 | 1.065 | 2.406 | 797 | 635 | 255 | 665 | tattgcacttgtcccggcct |
| **hsa-miR-92a-3p.3.P0.M.6** | 746 | 1.859 | 3.259 | 7.460 | 1.545 | 1.270 | 631 | 556 | tattgcacttgtcccggcc |
| **hsa-miR-92a-3p.3.P0.M.7** | 1.546 | 1.510 | 4.586 | 5.525 | 2.717 | 2.962 | 1.394 | 2.015 | tattgcacttgtcccggcctg |
| **hsa-miR-92a-3p.3.P0.S.11** | 1.268 | 2.091 | 4.397 | 4.876 | 1.171 | 1.070 | 1.138 | 1.015 | tattgcacttgtcccggcctgtt |
| **hsa-miR-92a-3p.3.P0.S.8** | 119 | 426 | 867 | 1.270 | 98 | 166 | 172 | 120 | tattgcacttgtcccggcctgttt |
| **hsa-miR-92a-3p.5.P0.M.7** | 1.328 | 1.549 | 3.025 | 2.649 | 2.195 | 2.306 | 485 | 1.190 | attgcacttgtcccggcctgt |
| **hsa-miR-93-3p.3.P0.S.7** | 588 | 194 | 334 | 413 | 1.024 | 1.118 | 204 | 193 | actgctgagctagcacttcccga |
| **hsa-miR-93-5p.0.P1.S.51** | 66 | 77 | 9 | 9 | 118 | 2.610 | 0 | 6 | caaagtgctgttcgtgcatgtag |
| **hsa-miR-93-5p.0.P1.S.60** | 502 | 348 | 479 | 117 | 591 | 566 | 255 | 229 | caaagtgctgttcgtgcaggtaa |
| **hsa-miR-93-5p.0.P1.S.61** | 6.215 | 310 | 2.501 | 448 | 3.701 | 697 | 727 | 669 | caaagtgctgttcgtgcaggtac |
| **hsa-miR-93-5p.0.P1.S.62** | 601 | 194 | 614 | 87 | 807 | 193 | 293 | 268 | caaagtgctgttcgtgcaggtat |
| **hsa-miR-93-5p.3.P0.S.10** | 5.614 | 736 | 2.591 | 3.036 | 3.120 | 1.553 | 794 | 780 | caaagtgctgttcgtgcaggta |
| **hsa-miR-93-5p.3.P0.S.17** | 535 | 852 | 524 | 87 | 787 | 759 | 351 | 188 | caaagtgctgttcgtgcaggtagt |
| **hsa-miR-93-5p.3.P0.S.8** | 1.559 | 1.123 | 1.345 | 348 | 1.969 | 2.741 | 309 | 361 | caaagtgctgttcgtgcaggt |
| **hsa-miR-99a-5p.3.P0.S.0** | 4.379 | 4.027 | 1.697 | 5.847 | 177 | 338 | 99 | 56 | aacccgtagatccgatcttgtga |
| **hsa-miR-99a-5p.3.P0.S.3** | 720 | 1.162 | 614 | 2.240 | 39 | 166 | 22 | 9 | aacccgtagatccgatcttgtgt |
| **hsa-miR-99a-5p.3.P0.S.5** | 7.681 | 7.551 | 2.907 | 6.978 | 502 | 532 | 89 | 122 | aacccgtagatccgatcttgt |
| **hsa-miR-99b-5p.3.P0.S.0** | 429 | 697 | 235 | 1.109 | 108 | 104 | 185 | 93 | cacccgtagaaccgaccttgcga |
| **hsa-miR-99b-5p.3.P0.S.3** | 634 | 426 | 388 | 687 | 108 | 97 | 118 | 126 | cacccgtagaaccgaccttg |
| **hsa-miR-99b-5p.3.P0.S.6** | 594 | 620 | 479 | 1.031 | 266 | 414 | 265 | 308 | cacccgtagaaccgaccttgc |
