# Supplementary material for: RNA-seq reveals distinctive RNA profiles of small extracellular vesicles from different human liver cancer cell lines
Source: Oncotarget. 2017 Aug 24;8(47):82920–39. doi: 10.18632/oncotarget.20503 (PMC5669939; doi:10.18632/oncotarget.20503)
Supplement: Supplementary file 5 [file oncotarget-08-82920-s005.docx]

**Table S3 B. 10 most expressed isomiRs**

| **miRNA** | **isomiR** | **Counts** | | | |
| --- | --- | --- | --- | --- | --- |
|  |  | **HuH7** | **Hep3B** | **HepG2** | **HuH6** |
| ***hsa-miR-23a-3p*** | *hsa-miR-23a-3p.3.P0.S.0* | 171 | 93 | 29 | 47 |
|  | *hsa-miR-23a-3p.3.P0.S.4* | 2478 | 1119 | 550 | 447 |
|  | *hsa-miR-23a-3p.3.P0.S.8* | 114 | 48 | 131 | 25 |
|  | *hsa-miR-23a-3p.3.P0.S.10* | 4639 | 2665 | 1306 | 533 |
|  | *hsa-miR-23a-3p.3.P0.S.14* | 124 | 39 | 53 | 19 |
|  | *hsa-miR-23a-3p.3.P0.S.19* | 10242 | 4266 | 3536 | 2129 |
|  | *hsa-miR-23a-3p.3.P0.S.20* | 65034 | 25722 | 37092 | 8993 |
|  | *hsa-miR-23a-3p.3.P0.S.21* | 6968 | 1882 | 3893 | 979 |
|  | *hsa-miR-23a-3p.3.P0.S.22* | 12174 | 3623 | 6254 | 1464 |
|  | *hsa-miR-23a-3p.3.P1.S.125* | 156 | 33 | 41 | 6 |
|  | *hsa-miR-23a-3p.3.P0.M.1* | 1801 | 1046 | 2138 | 488 |
|  | *hsa-miR-23a-3p.53.P0.S.16* | 206 | 35 | 98 | 31 |
|  | *hsa-miR-23a-3p.53.P0.S.18* | 805 | 94 | 255 | 43 |
|  | *hsa-miR-23a-3p.53.P0.S.21* | 130 | 44 | 47 | 6 |
|  | *hsa-miR-23a-3p.53.P0.S.28* | 178 | 24 | 33 | 15 |
|  | *hsa-miR-23a-3p.0.P1.S.20* | 356 | 125 | 198 | 20 |
|  | *hsa-miR-23a-3p.0.P1.S.22* | 143 | 35 | 27 | 13 |
| ***hsa-miR-20a-5p*** | *hsa-miR-20a-5p.3.P0.S.2* | 186 | 805 | 221 | 151 |
|  | *hsa-miR-20a-5p.3.P0.S.11* | 2151 | 8304 | 3992 | 955 |
|  | *hsa-miR-20a-5p.3.P1.S.24* | 23 | 679 | 10 | 1 |
|  | *hsa-miR-20a-5p.3.P0.S.13* | 594 | 2703 | 539 | 251 |
|  | *hsa-miR-20a-5p.3.P1.S.46* | 7 | 193 | 71 | 13 |
|  | *hsa-miR-20a-5p.3.P0.S.15* | 274 | 823 | 414 | 157 |
|  | *hsa-miR-20a-5p.3.P0.S.19* | 255 | 1553 | 275 | 362 |
|  | *hsa-miR-20a-5p.53.P0.S.6* | 20 | 67 | 139 | 13 |
|  | *hsa-miR-20a-5p.5.P1.S.1* | 13 | 125 | 0 | 14 |
|  | *hsa-miR-20a-5p.53.P0.S.11* | 75 | 266 | 207 | 40 |
|  | *hsa-miR-20a-5p.5.P0.S.9* | 720 | 2288 | 691 | 299 |
|  | *hsa-miR-20a-5p.5.P1.S.17* | 26 | 113 | 33 | 22 |
|  | *hsa-miR-20a-5p.0.P1.S.40* | 546 | 3379 | 799 | 619 |
|  | *hsa-miR-20a-5p.0.P1.S.41* | 33 | 357 | 12 | 5 |
|  | *hsa-miR-20a-5p.0.P1.S.42* | 823 | 3412 | 567 | 658 |
| ***hsa-miR-19b-3p*** | *hsa-miR-19b-3p.3.P0.M.0* | 135 | 325 | 25 | 124 |
|  | *hsa-miR-19b-3p.3.P0.M.4* | 11397 | 18038 | 8065 | 8239 |
|  | *hsa-miR-19b-3p.3.P1.M.42* | 36 | 116 | 73 | 7 |
|  | *hsa-miR-19b-3p.3.P1.M.45* | 3 | 126 | 12 | 0 |
|  | *hsa-miR-19b-3p.3.P1.M.55* | 111 | 146 | 143 | 28 |
|  | *hsa-miR-19b-3p.3.P1.M.56* | 199 | 146 | 157 | 29 |
|  | *hsa-miR-19b-3p.3.P1.M.57* | 171 | 105 | 189 | 34 |
|  | *hsa-miR-19b-3p.3.P0.M.5* | 20 | 519 | 93 | 17 |
|  | *hsa-miR-19b-3p.3.P0.M.6* | 1612 | 6245 | 1968 | 941 |
|  | *hsa-miR-19b-3p.3.P0.M.7* | 114 | 553 | 178 | 58 |
|  | *hsa-miR-19b-3p.53.P0.M.7* | 131 | 260 | 222 | 102 |
|  | *hsa-miR-19b-3p.5.P0.M.8* | 886 | 1390 | 448 | 218 |
|  | *hsa-miR-19b-3p.0.P1.M.30* | 10 | 240 | 17 | 5 |
|  | *hsa-miR-19b-3p.0.P1.M.31* | 270 | 137 | 37 | 15 |
|  | *hsa-miR-19b-3p.0.P1.M.32* | 20 | 112 | 13 | 4 |
|  | *hsa-miR-19b-3p.0.P1.M.48* | 160 | 431 | 44 | 50 |
|  | *hsa-miR-19b-3p.0.P1.M.49* | 164 | 364 | 84 | 39 |
|  | *hsa-miR-19b-3p.0.P1.M.50* | 935 | 4166 | 653 | 944 |
|  | *hsa-miR-19b-3p.3.P0.S.4* | 467 | 428 | 75 | 257 |
| ***hsa-miR-19a-3p*** | *hsa-miR-19a-3p.3.P0.S.0* | 36 | 103 | 24 | 42 |
|  | *hsa-miR-19a-3p.3.P0.S.4* | 0 | 627 | 102 | 5 |
|  | *hsa-miR-19a-3p.3.P0.S.5* | 2294 | 3748 | 1909 | 1841 |
|  | *hsa-miR-19a-3p.3.P0.S.7* | 584 | 1923 | 547 | 357 |
|  | *hsa-miR-19a-3p.3.P0.S.11* | 476 | 439 | 59 | 136 |
|  | *hsa-miR-19a-3p.53.P0.S.8* | 117 | 66 | 20 | 17 |
|  | *hsa-miR-19a-3p.5.P0.S.5* | 297 | 370 | 128 | 66 |
|  | *hsa-miR-19a-3p.0.P1.S.37* | 110 | 68 | 23 | 13 |
|  | *hsa-miR-19a-3p.0.P1.S.38* | 272 | 432 | 125 | 168 |
| ***hsa-miR-17-5p*** | *hsa-miR-17-5p.3.P0.S.2* | 1207 | 2530 | 596 | 618 |
|  | *hsa-miR-17-5p.3.P0.S.11* | 10295 | 39687 | 20489 | 3032 |
|  | *hsa-miR-17-5p.3.P1.S.28* | 33 | 455 | 212 | 65 |
|  | *hsa-miR-17-5p.3.P1.S.29* | 17 | 2944 | 26 | 6 |
|  | *hsa-miR-17-5p.3.P0.S.13* | 2835 | 16917 | 3257 | 1371 |
|  | *hsa-miR-17-5p.3.P1.S.61* | 0 | 161 | 42 | 33 |
|  | *hsa-miR-17-5p.3.P1.S.65* | 7 | 148 | 107 | 6 |
|  | *hsa-miR-17-5p.3.P1.S.66* | 186 | 824 | 233 | 90 |
|  | *hsa-miR-17-5p.3.P0.S.14* | 113 | 190 | 212 | 79 |
|  | *hsa-miR-17-5p.3.P0.S.15* | 643 | 1977 | 966 | 350 |
|  | *hsa-miR-17-5p.3.P0.S.19* | 2210 | 10450 | 2690 | 2186 |
|  | *hsa-miR-17-5p.53.P0.S.12* | 1472 | 3878 | 2718 | 350 |
|  | *hsa-miR-17-5p.53.P0.S.14* | 322 | 1363 | 308 | 145 |
|  | *hsa-miR-17-5p.53.P1.S.32* | 56 | 153 | 49 | 21 |
|  | *hsa-miR-17-5p.53.P0.S.16* | 177 | 234 | 240 | 73 |
|  | *hsa-miR-17-5p.5.P0.S.4* | 578 | 762 | 273 | 145 |
|  | *hsa-miR-17-5p.0.P1.S.45* | 1610 | 4629 | 1329 | 870 |
|  | *hsa-miR-17-5p.0.P1.S.46* | 130 | 5117 | 91 | 60 |
|  | *hsa-miR-17-5p.0.P1.S.47* | 2964 | 16024 | 4188 | 3141 |
| ***hsa-miR-18a-5p*** | *hsa-miR-18a-5p.3.P0.S.1* | 39 | 635 | 125 | 126 |
|  | *hsa-miR-18a-5p.3.P0.S.4* | 530 | 7017 | 2449 | 417 |
|  | *hsa-miR-18a-5p.3.P1.S.16* | 236 | 170 | 38 | 10 |
|  | *hsa-miR-18a-5p.3.P1.S.17* | 0 | 7 | 12 | 2 |
|  | *hsa-miR-18a-5p.3.P0.M.0* | 277 | 293 | 214 | 95 |
|  | *hsa-miR-18a-5p.3.P0.S.5* | 563 | 12247 | 2115 | 980 |
|  | *hsa-miR-18a-5p.3.P0.S.6* | 184 | 2275 | 735 | 188 |
|  | *hsa-miR-18a-5p.3.P0.S.13* | 204 | 1229 | 250 | 222 |
|  | *hsa-miR-18a-5p.53.P0.S.2* | 10 | 117 | 22 | 5 |
|  | *hsa-miR-18a-5p.5.P0.S.5* | 115 | 669 | 82 | 45 |
|  | *hsa-miR-18a-5p.0.P1.S.32* | 10 | 410 | 66 | 80 |
|  | *hsa-miR-18a-5p.0.P1.S.34* | 45 | 267 | 59 | 31 |
| ***hsa-miR-92a-3p*** | *hsa-miR-92a-3p.3.P0.M.0* | 0 | 42 | 10 | 6 |
|  | *hsa-miR-92a-3p.3.P0.M.1* | 49 | 122 | 48 | 17 |
|  | *hsa-miR-92a-3p.3.P0.M.2* | 17 | 217 | 62 | 30 |
|  | *hsa-miR-92a-3p.3.P0.M.3* | 17 | 276 | 54 | 9 |
|  | *hsa-miR-92a-3p.3.P0.S.0* | 0 | 0 | 0 | 5 |
|  | *hsa-miR-92a-3p.3.P0.S.1* | 3 | 39 | 0 | 10 |
|  | *hsa-miR-92a-3p.3.P0.S.2* | 75 | 336 | 104 | 50 |
|  | *hsa-miR-92a-3p.3.P0.S.3* | 7 | 29 | 0 | 1 |
|  | *hsa-miR-92a-3p.3.P0.S.4* | 0 | 5 | 0 | 0 |
|  | *hsa-miR-92a-3p.3.P0.S.5* | 0 | 7 | 0 | 0 |
|  | *hsa-miR-92a-3p.3.P0.S.6* | 0 | 2 | 0 | 0 |
|  | *hsa-miR-92a-3p.3.P0.S.7* | 0 | 2 | 0 | 1 |
|  | *hsa-miR-92a-3p.3.P0.S.8* | 272 | 1068 | 132 | 146 |
|  | *hsa-miR-92a-3p.3.P0.M.4* | 671 | 2307 | 817 | 287 |
|  | *hsa-miR-92a-3p.3.P1.M.0* | 0 | 5 | 3 | 0 |
|  | *hsa-miR-92a-3p.3.P1.M.1* | 0 | 0 | 3 | 0 |
|  | *hsa-miR-92a-3p.3.P1.M.2* | 0 | 0 | 0 | 1 |
|  | *hsa-miR-92a-3p.3.P1.M.3* | 0 | 2 | 0 | 1 |
|  | *hsa-miR-92a-3p.3.P1.M.4* | 0 | 2 | 0 | 0 |
|  | *hsa-miR-92a-3p.3.P1.M.5* | 0 | 4 | 0 | 0 |
|  | *hsa-miR-92a-3p.3.P1.M.6* | 0 | 2 | 0 | 0 |
|  | *hsa-miR-92a-3p.3.P1.M.7* | 0 | 5 | 0 | 0 |
|  | *hsa-miR-92a-3p.3.P1.M.8* | 0 | 4 | 0 | 0 |
|  | *hsa-miR-92a-3p.3.P1.M.9* | 0 | 2 | 0 | 0 |
|  | *hsa-miR-92a-3p.3.P1.M.10* | 0 | 2 | 5 | 0 |
|  | *hsa-miR-92a-3p.3.P1.M.11* | 0 | 5 | 0 | 0 |
|  | *hsa-miR-92a-3p.3.P1.M.12* | 0 | 4 | 0 | 0 |
|  | *hsa-miR-92a-3p.3.P1.M.13* | 3 | 20 | 10 | 0 |
|  | *hsa-miR-92a-3p.3.P1.M.14* | 0 | 26 | 12 | 5 |
|  | *hsa-miR-92a-3p.3.P1.M.15* | 19 | 13 | 3 | 1 |
|  | *hsa-miR-92a-3p.3.P1.M.16* | 3 | 20 | 5 | 1 |
|  | *hsa-miR-92a-3p.3.P1.M.17* | 0 | 20 | 5 | 3 |
|  | *hsa-miR-92a-3p.3.P1.M.18* | 0 | 4 | 0 | 1 |
|  | *hsa-miR-92a-3p.3.P1.M.19* | 19 | 13 | 10 | 4 |
|  | *hsa-miR-92a-3p.3.P1.M.20* | 0 | 41 | 3 | 0 |
|  | *hsa-miR-92a-3p.3.P1.M.21* | 49 | 55 | 14 | 5 |
|  | *hsa-miR-92a-3p.3.P0.M.5* | 291 | 1735 | 716 | 460 |
|  | *hsa-miR-92a-3p.3.P1.M.22* | 0 | 2 | 0 | 0 |
|  | *hsa-miR-92a-3p.3.P1.M.23* | 0 | 2 | 0 | 0 |
|  | *hsa-miR-92a-3p.3.P1.M.24* | 0 | 5 | 0 | 0 |
|  | *hsa-miR-92a-3p.3.P1.M.25* | 0 | 2 | 0 | 0 |
|  | *hsa-miR-92a-3p.3.P1.M.26* | 0 | 2 | 0 | 0 |
|  | *hsa-miR-92a-3p.3.P1.M.27* | 0 | 0 | 5 | 0 |
|  | *hsa-miR-92a-3p.3.P1.M.28* | 0 | 2 | 0 | 0 |
|  | *hsa-miR-92a-3p.3.P1.M.29* | 0 | 4 | 0 | 1 |
|  | *hsa-miR-92a-3p.3.P1.M.30* | 0 | 15 | 0 | 1 |
|  | *hsa-miR-92a-3p.3.P1.M.31* | 0 | 7 | 0 | 1 |
|  | *hsa-miR-92a-3p.3.P1.M.32* | 19 | 2 | 5 | 0 |
|  | *hsa-miR-92a-3p.3.P1.M.33* | 0 | 4 | 0 | 0 |
|  | *hsa-miR-92a-3p.3.P1.M.34* | 0 | 4 | 0 | 2 |
|  | *hsa-miR-92a-3p.3.P1.M.35* | 0 | 2 | 0 | 0 |
|  | *hsa-miR-92a-3p.3.P1.M.36* | 0 | 0 | 0 | 1 |
|  | *hsa-miR-92a-3p.3.P1.M.37* | 0 | 0 | 0 | 1 |
|  | *hsa-miR-92a-3p.3.P1.M.38* | 0 | 24 | 29 | 1 |
|  | *hsa-miR-92a-3p.3.P1.M.39* | 0 | 25 | 5 | 1 |
|  | *hsa-miR-92a-3p.3.P1.M.40* | 0 | 2 | 0 | 1 |
|  | *hsa-miR-92a-3p.3.P0.S.9* | 0 | 7 | 0 | 0 |
|  | *hsa-miR-92a-3p.3.P1.S.0* | 0 | 0 | 0 | 2 |
|  | *hsa-miR-92a-3p.3.P0.S.10* | 0 | 24 | 3 | 6 |
|  | *hsa-miR-92a-3p.3.P1.S.1* | 0 | 2 | 0 | 0 |
|  | *hsa-miR-92a-3p.3.P1.S.2* | 0 | 11 | 0 | 0 |
|  | *hsa-miR-92a-3p.3.P0.M.6* | 1302 | 5360 | 1408 | 594 |
|  | *hsa-miR-92a-3p.3.P1.M.41* | 0 | 0 | 3 | 1 |
|  | *hsa-miR-92a-3p.3.P1.M.42* | 0 | 0 | 0 | 2 |
|  | *hsa-miR-92a-3p.3.P1.M.43* | 0 | 2 | 0 | 0 |
|  | *hsa-miR-92a-3p.3.P1.M.44* | 0 | 2 | 0 | 0 |
|  | *hsa-miR-92a-3p.3.P1.M.45* | 0 | 0 | 3 | 0 |
|  | *hsa-miR-92a-3p.3.P1.M.46* | 0 | 0 | 3 | 0 |
|  | *hsa-miR-92a-3p.3.P1.M.47* | 0 | 0 | 0 | 2 |
|  | *hsa-miR-92a-3p.3.P1.M.48* | 0 | 4 | 3 | 1 |
|  | *hsa-miR-92a-3p.3.P1.M.49* | 0 | 5 | 0 | 0 |
|  | *hsa-miR-92a-3p.3.P1.M.50* | 0 | 4 | 0 | 0 |
|  | *hsa-miR-92a-3p.3.P1.M.51* | 0 | 5 | 0 | 0 |
|  | *hsa-miR-92a-3p.3.P1.M.52* | 0 | 2 | 3 | 1 |
|  | *hsa-miR-92a-3p.3.P1.M.53* | 0 | 2 | 0 | 0 |
|  | *hsa-miR-92a-3p.3.P1.M.54* | 0 | 0 | 0 | 1 |
|  | *hsa-miR-92a-3p.3.P1.M.55* | 19 | 17 | 0 | 1 |
|  | *hsa-miR-92a-3p.3.P1.M.56* | 0 | 20 | 0 | 4 |
|  | *hsa-miR-92a-3p.3.P1.M.57* | 0 | 15 | 0 | 2 |
|  | *hsa-miR-92a-3p.3.P1.M.58* | 0 | 2 | 7 | 0 |
|  | *hsa-miR-92a-3p.3.P1.M.59* | 0 | 11 | 3 | 1 |
|  | *hsa-miR-92a-3p.3.P1.M.60* | 0 | 11 | 0 | 2 |
|  | *hsa-miR-92a-3p.3.P1.M.61* | 0 | 22 | 0 | 5 |
|  | *hsa-miR-92a-3p.3.P1.M.62* | 0 | 4 | 10 | 0 |
|  | *hsa-miR-92a-3p.3.P1.M.63* | 0 | 18 | 0 | 0 |
|  | *hsa-miR-92a-3p.3.P0.M.7* | 1528 | 5056 | 2839 | 1704 |
|  | *hsa-miR-92a-3p.3.P0.S.11* | 1680 | 4637 | 1121 | 1077 |
|  | *hsa-miR-92a-3p.3.P1.S.3* | 0 | 0 | 0 | 2 |
|  | *hsa-miR-92a-3p.3.P1.S.4* | 0 | 2 | 0 | 0 |
|  | *hsa-miR-92a-3p.3.P1.M.64* | 0 | 2 | 3 | 2 |
|  | *hsa-miR-92a-3p.3.P1.M.65* | 0 | 2 | 0 | 0 |
|  | *hsa-miR-92a-3p.3.P1.S.5* | 0 | 0 | 0 | 1 |
|  | *hsa-miR-92a-3p.3.P1.M.66* | 0 | 0 | 0 | 3 |
|  | *hsa-miR-92a-3p.3.P1.M.67* | 0 | 0 | 3 | 1 |
|  | *hsa-miR-92a-3p.3.P1.S.6* | 0 | 0 | 0 | 1 |
|  | *hsa-miR-92a-3p.3.P1.M.68* | 0 | 4 | 0 | 4 |
|  | *hsa-miR-92a-3p.3.P1.S.7* | 19 | 0 | 0 | 0 |
|  | *hsa-miR-92a-3p.3.P1.M.69* | 3 | 0 | 0 | 0 |
|  | *hsa-miR-92a-3p.3.P1.M.70* | 0 | 0 | 0 | 1 |
|  | *hsa-miR-92a-3p.3.P1.M.71* | 3 | 0 | 5 | 0 |
|  | *hsa-miR-92a-3p.3.P1.S.8* | 0 | 0 | 0 | 1 |
|  | *hsa-miR-92a-3p.3.P1.S.9* | 0 | 4 | 0 | 0 |
|  | *hsa-miR-92a-3p.3.P1.M.72* | 19 | 0 | 0 | 1 |
|  | *hsa-miR-92a-3p.3.P1.S.10* | 7 | 0 | 0 | 0 |
|  | *hsa-miR-92a-3p.3.P1.M.73* | 0 | 5 | 0 | 0 |
|  | *hsa-miR-92a-3p.3.P1.M.74* | 0 | 0 | 0 | 1 |
|  | *hsa-miR-92a-3p.3.P1.M.75* | 0 | 0 | 0 | 1 |
|  | *hsa-miR-92a-3p.3.P1.S.11* | 0 | 0 | 0 | 1 |
|  | *hsa-miR-92a-3p.3.P1.M.76* | 0 | 20 | 8 | 3 |
|  | *hsa-miR-92a-3p.3.P1.S.12* | 3 | 7 | 0 | 1 |
|  | *hsa-miR-92a-3p.3.P1.M.77* | 3 | 22 | 15 | 4 |
|  | *hsa-miR-92a-3p.3.P1.S.13* | 0 | 9 | 3 | 1 |
|  | *hsa-miR-92a-3p.3.P1.M.78* | 0 | 5 | 3 | 1 |
|  | *hsa-miR-92a-3p.3.P1.S.14* | 0 | 2 | 0 | 0 |
|  | *hsa-miR-92a-3p.3.P1.M.79* | 0 | 2 | 0 | 0 |
|  | *hsa-miR-92a-3p.3.P1.M.80* | 0 | 5 | 0 | 1 |
|  | *hsa-miR-92a-3p.3.P1.M.81* | 3 | 4 | 0 | 0 |
|  | *hsa-miR-92a-3p.3.P1.M.82* | 3 | 4 | 0 | 1 |
|  | *hsa-miR-92a-3p.3.P1.S.15* | 0 | 5 | 0 | 0 |
|  | *hsa-miR-92a-3p.3.P1.M.83* | 0 | 0 | 0 | 1 |
|  | *hsa-miR-92a-3p.3.P1.S.16* | 0 | 5 | 0 | 0 |
|  | *hsa-miR-92a-3p.3.P1.M.84* | 0 | 0 | 3 | 0 |
|  | *hsa-miR-92a-3p.3.P1.S.17* | 0 | 5 | 0 | 2 |
|  | *hsa-miR-92a-3p.3.P1.M.85* | 0 | 2 | 0 | 1 |
|  | *hsa-miR-92a-3p.3.P1.S.18* | 0 | 7 | 7 | 4 |
|  | *hsa-miR-92a-3p.3.P1.S.19* | 0 | 2 | 0 | 0 |
|  | *hsa-miR-92a-3p.3.P1.M.86* | 131 | 252 | 236 | 97 |
|  | *hsa-miR-92a-3p.3.P1.S.20* | 0 | 13 | 0 | 2 |
|  | *hsa-miR-92a-3p.3.P1.M.87* | 0 | 11 | 15 | 4 |
|  | *hsa-miR-92a-3p.3.P1.S.21* | 0 | 0 | 0 | 2 |
|  | *hsa-miR-92a-3p.3.P1.M.88* | 10 | 53 | 60 | 38 |
|  | *hsa-miR-92a-3p.3.P1.S.22* | 0 | 2 | 0 | 0 |
|  | *hsa-miR-92a-3p.3.P1.S.23* | 17 | 156 | 29 | 47 |
|  | *hsa-miR-92a-3p.3.P1.S.24* | 19 | 2 | 5 | 2 |
|  | *hsa-miR-92a-3p.3.P1.S.25* | 0 | 0 | 0 | 1 |
|  | *hsa-miR-92a-3p.53.P0.M.0* | 0 | 2 | 0 | 0 |
|  | *hsa-miR-92a-3p.5.P0.M.0* | 3 | 9 | 3 | 4 |
|  | *hsa-miR-92a-3p.53.P0.M.1* | 0 | 2 | 0 | 0 |
|  | *hsa-miR-92a-3p.5.P0.M.1* | 0 | 2 | 0 | 0 |
|  | *hsa-miR-92a-3p.53.P0.M.2* | 0 | 0 | 3 | 0 |
|  | *hsa-miR-92a-3p.53.P0.M.3* | 0 | 0 | 0 | 2 |
|  | *hsa-miR-92a-3p.5.P0.M.2* | 0 | 9 | 0 | 2 |
|  | *hsa-miR-92a-3p.53.P0.S.0* | 0 | 0 | 0 | 1 |
|  | *hsa-miR-92a-3p.5.P0.M.3* | 0 | 4 | 0 | 0 |
|  | *hsa-miR-92a-3p.5.P1.M.0* | 0 | 5 | 0 | 0 |
|  | *hsa-miR-92a-3p.53.P0.M.4* | 0 | 5 | 0 | 1 |
|  | *hsa-miR-92a-3p.53.P0.M.5* | 0 | 0 | 0 | 2 |
|  | *hsa-miR-92a-3p.53.P0.M.6* | 7 | 9 | 0 | 0 |
|  | *hsa-miR-92a-3p.53.P0.S.1* | 0 | 0 | 5 | 0 |
|  | *hsa-miR-92a-3p.53.P0.S.2* | 7 | 11 | 0 | 1 |
|  | *hsa-miR-92a-3p.53.P0.S.3* | 0 | 0 | 0 | 2 |
|  | *hsa-miR-92a-3p.53.P0.S.4* | 0 | 0 | 5 | 0 |
|  | *hsa-miR-92a-3p.53.P0.S.5* | 0 | 22 | 0 | 12 |
|  | *hsa-miR-92a-3p.53.P0.M.7* | 77 | 20 | 13 | 16 |
|  | *hsa-miR-92a-3p.53.P0.S.6* | 0 | 0 | 3 | 1 |
|  | *hsa-miR-92a-3p.53.P0.M.8* | 65 | 148 | 84 | 15 |
|  | *hsa-miR-92a-3p.53.P1.M.0* | 0 | 7 | 0 | 0 |
|  | *hsa-miR-92a-3p.53.P1.M.1* | 0 | 0 | 3 | 0 |
|  | *hsa-miR-92a-3p.53.P1.M.2* | 0 | 7 | 0 | 1 |
|  | *hsa-miR-92a-3p.53.P1.M.3* | 0 | 0 | 5 | 1 |
|  | *hsa-miR-92a-3p.53.P1.M.4* | 0 | 2 | 3 | 1 |
|  | *hsa-miR-92a-3p.53.P0.S.7* | 52 | 128 | 28 | 66 |
|  | *hsa-miR-92a-3p.53.P0.M.9* | 29 | 133 | 155 | 43 |
|  | *hsa-miR-92a-3p.53.P1.S.0* | 0 | 0 | 0 | 1 |
|  | *hsa-miR-92a-3p.53.P1.M.5* | 0 | 0 | 0 | 1 |
|  | *hsa-miR-92a-3p.53.P1.S.1* | 0 | 4 | 0 | 0 |
|  | *hsa-miR-92a-3p.53.P1.M.6* | 0 | 2 | 8 | 5 |
|  | *hsa-miR-92a-3p.53.P1.M.7* | 0 | 0 | 3 | 0 |
|  | *hsa-miR-92a-3p.53.P1.S.2* | 0 | 2 | 0 | 0 |
|  | *hsa-miR-92a-3p.53.P0.S.8* | 0 | 0 | 0 | 1 |
|  | *hsa-miR-92a-3p.53.P0.S.9* | 0 | 0 | 0 | 1 |
|  | *hsa-miR-92a-3p.53.P0.M.10* | 0 | 5 | 0 | 0 |
|  | *hsa-miR-92a-3p.53.P0.M.11* | 0 | 4 | 0 | 0 |
|  | *hsa-miR-92a-3p.53.P0.S.10* | 0 | 0 | 0 | 1 |
|  | *hsa-miR-92a-3p.53.P1.M.8* | 0 | 2 | 0 | 0 |
|  | *hsa-miR-92a-3p.5.P0.M.4* | 23 | 13 | 25 | 5 |
|  | *hsa-miR-92a-3p.5.P1.M.1* | 0 | 2 | 0 | 0 |
|  | *hsa-miR-92a-3p.53.P0.M.12* | 0 | 0 | 3 | 0 |
|  | *hsa-miR-92a-3p.53.P1.M.9* | 0 | 0 | 3 | 0 |
|  | *hsa-miR-92a-3p.5.P0.M.5* | 3 | 38 | 38 | 4 |
|  | *hsa-miR-92a-3p.53.P0.M.13* | 0 | 5 | 0 | 0 |
|  | *hsa-miR-92a-3p.53.P0.S.11* | 0 | 2 | 0 | 0 |
|  | *hsa-miR-92a-3p.53.P0.S.12* | 0 | 9 | 0 | 2 |
|  | *hsa-miR-92a-3p.53.P0.M.14* | 19 | 20 | 3 | 0 |
|  | *hsa-miR-92a-3p.53.P0.M.15* | 0 | 5 | 0 | 1 |
|  | *hsa-miR-92a-3p.53.P0.S.13* | 0 | 0 | 0 | 2 |
|  | *hsa-miR-92a-3p.5.P0.M.6* | 3 | 49 | 91 | 22 |
|  | *hsa-miR-92a-3p.5.P1.M.2* | 0 | 0 | 5 | 0 |
|  | *hsa-miR-92a-3p.5.P1.M.3* | 0 | 5 | 10 | 0 |
|  | *hsa-miR-92a-3p.5.P0.M.7* | 1438 | 2837 | 2250 | 837 |
|  | *hsa-miR-92a-3p.5.P1.M.4* | 3 | 2 | 0 | 0 |
|  | *hsa-miR-92a-3p.5.P1.M.5* | 0 | 0 | 0 | 2 |
|  | *hsa-miR-92a-3p.5.P1.M.6* | 0 | 0 | 3 | 0 |
|  | *hsa-miR-92a-3p.5.P1.M.7* | 0 | 0 | 10 | 0 |
|  | *hsa-miR-92a-3p.5.P1.M.8* | 0 | 4 | 3 | 0 |
|  | *hsa-miR-92a-3p.5.P1.M.9* | 0 | 2 | 0 | 1 |
|  | *hsa-miR-92a-3p.5.P1.M.10* | 0 | 0 | 0 | 1 |
|  | *hsa-miR-92a-3p.5.P1.M.11* | 0 | 0 | 3 | 0 |
|  | *hsa-miR-92a-3p.5.P1.M.12* | 0 | 0 | 0 | 1 |
|  | *hsa-miR-92a-3p.5.P1.M.13* | 0 | 5 | 3 | 0 |
|  | *hsa-miR-92a-3p.5.P1.M.14* | 0 | 7 | 0 | 1 |
|  | *hsa-miR-92a-3p.5.P1.M.15* | 0 | 15 | 0 | 1 |
|  | *hsa-miR-92a-3p.5.P1.M.16* | 0 | 2 | 0 | 0 |
|  | *hsa-miR-92a-3p.5.P1.M.17* | 0 | 9 | 0 | 0 |
|  | *hsa-miR-92a-3p.5.P1.M.18* | 0 | 2 | 3 | 1 |
|  | *hsa-miR-92a-3p.5.P1.M.19* | 0 | 9 | 3 | 0 |
|  | *hsa-miR-92a-3p.5.P1.M.20* | 19 | 73 | 66 | 13 |
|  | *hsa-miR-92a-3p.5.P1.M.21* | 19 | 377 | 12 | 10 |
|  | *hsa-miR-92a-3p.5.P1.M.22* | 0 | 4 | 0 | 2 |
|  | *hsa-miR-92a-3p.0.P1.M.0* | 0 | 2 | 0 | 0 |
|  | *hsa-miR-92a-3p.0.P1.M.1* | 59 | 29 | 17 | 4 |
|  | *hsa-miR-92a-3p.0.P1.M.2* | 39 | 2 | 3 | 0 |
|  | *hsa-miR-92a-3p.0.P1.M.3* | 20 | 16 | 8 | 5 |
|  | *hsa-miR-92a-3p.0.P1.M.4* | 0 | 5 | 0 | 0 |
|  | *hsa-miR-92a-3p.0.P1.M.5* | 3 | 0 | 0 | 0 |
|  | *hsa-miR-92a-3p.0.P1.M.6* | 17 | 24 | 20 | 13 |
|  | *hsa-miR-92a-3p.0.P1.M.7* | 0 | 0 | 5 | 0 |
|  | *hsa-miR-92a-3p.0.P1.M.8* | 0 | 0 | 3 | 1 |
|  | *hsa-miR-92a-3p.0.P1.M.9* | 19 | 29 | 29 | 8 |
|  | *hsa-miR-92a-3p.0.P1.M.10* | 7 | 5 | 3 | 2 |
|  | *hsa-miR-92a-3p.0.P1.M.11* | 23 | 27 | 54 | 13 |
|  | *hsa-miR-92a-3p.0.P1.M.12* | 3 | 0 | 0 | 0 |
|  | *hsa-miR-92a-3p.0.P1.M.13* | 10 | 47 | 7 | 5 |
|  | *hsa-miR-92a-3p.0.P1.M.14* | 55 | 40 | 50 | 19 |
|  | *hsa-miR-92a-3p.0.P1.M.15* | 0 | 0 | 3 | 1 |
|  | *hsa-miR-92a-3p.0.P1.M.16* | 3 | 2 | 8 | 4 |
|  | *hsa-miR-92a-3p.0.P1.M.17* | 0 | 2 | 0 | 0 |
|  | *hsa-miR-92a-3p.0.P1.M.18* | 85 | 39 | 49 | 17 |
|  | *hsa-miR-92a-3p.0.P1.M.19* | 0 | 0 | 0 | 2 |
|  | *hsa-miR-92a-3p.0.P1.M.20* | 0 | 2 | 0 | 0 |
|  | *hsa-miR-92a-3p.0.P1.M.21* | 29 | 22 | 49 | 9 |
|  | *hsa-miR-92a-3p.0.P1.M.22* | 0 | 7 | 3 | 0 |
|  | *hsa-miR-92a-3p.0.P1.M.23* | 13 | 34 | 25 | 8 |
|  | *hsa-miR-92a-3p.0.P1.M.24* | 0 | 11 | 10 | 6 |
|  | *hsa-miR-92a-3p.0.P1.M.25* | 0 | 0 | 0 | 3 |
|  | *hsa-miR-92a-3p.0.P1.M.26* | 29 | 76 | 43 | 18 |
|  | *hsa-miR-92a-3p.0.P1.M.27* | 0 | 7 | 0 | 0 |
|  | *hsa-miR-92a-3p.0.P1.M.28* | 0 | 0 | 0 | 2 |
|  | *hsa-miR-92a-3p.0.P1.M.29* | 0 | 0 | 0 | 2 |
|  | *hsa-miR-92a-3p.0.P1.M.30* | 19 | 16 | 28 | 7 |
|  | *hsa-miR-92a-3p.0.P1.M.31* | 0 | 5 | 0 | 0 |
|  | *hsa-miR-92a-3p.0.P1.M.32* | 7 | 0 | 10 | 0 |
|  | *hsa-miR-92a-3p.0.P1.M.33* | 10 | 20 | 34 | 10 |
|  | *hsa-miR-92a-3p.0.P1.M.34* | 0 | 4 | 0 | 3 |
|  | *hsa-miR-92a-3p.0.P1.M.35* | 46 | 73 | 66 | 12 |
|  | *hsa-miR-92a-3p.0.P1.M.36* | 17 | 119 | 73 | 25 |
|  | *hsa-miR-92a-3p.0.P1.M.37* | 23 | 162 | 72 | 35 |
|  | *hsa-miR-92a-3p.0.P1.M.38* | 13 | 51 | 10 | 24 |
|  | *hsa-miR-92a-3p.0.P1.M.39* | 7 | 49 | 42 | 13 |
|  | *hsa-miR-92a-3p.0.P1.M.40* | 0 | 4 | 3 | 0 |
|  | *hsa-miR-92a-3p.0.P1.M.41* | 19 | 33 | 5 | 4 |
|  | *hsa-miR-92a-3p.0.P1.M.42* | 0 | 0 | 0 | 1 |
|  | *hsa-miR-92a-3p.0.P1.M.43* | 7 | 22 | 12 | 5 |
|  | *hsa-miR-92a-3p.0.P1.M.44* | 0 | 2 | 5 | 4 |
|  | *hsa-miR-92a-3p.0.P1.M.45* | 3 | 7 | 3 | 1 |
|  | *hsa-miR-92a-3p.0.P1.M.46* | 7 | 44 | 27 | 7 |
|  | *hsa-miR-92a-3p.0.P1.M.47* | 7 | 7 | 0 | 1 |
|  | *hsa-miR-92a-3p.0.P1.M.48* | 75 | 164 | 158 | 34 |
|  | *hsa-miR-92a-3p.0.P1.M.49* | 3 | 0 | 8 | 0 |
|  | *hsa-miR-92a-3p.0.P1.M.50* | 165 | 146 | 68 | 45 |
|  | *hsa-miR-92a-3p.0.P1.M.51* | 3 | 2 | 0 | 2 |
|  | *hsa-miR-92a-3p.0.P1.M.52* | 26 | 66 | 37 | 21 |
|  | *hsa-miR-92a-3p.0.P1.M.53* | 684 | 2686 | 1664 | 996 |
|  | *hsa-miR-92a-3p.0.P1.M.54* | 279 | 12102 | 418 | 199 |
|  | *hsa-miR-92a-3p.0.P1.M.55* | 128 | 740 | 404 | 200 |
|  | *hsa-miR-92a-2-5p.3.P0.S.0* | 0 | 0 | 0 | 8 |
|  | *hsa-miR-92a-2-5p.3.P0.S.1* | 0 | 0 | 0 | 3 |
|  | *hsa-miR-92a-2-5p.3.P0.S.2* | 0 | 0 | 0 | 1 |
|  | *hsa-miR-92a-2-5p.53.P0.S.0* | 0 | 0 | 0 | 2 |
|  | *hsa-miR-92a-3p.3.P0.S.12* | 0 | 2 | 0 | 0 |
|  | *hsa-miR-92a-3p.3.P0.S.13* | 0 | 2 | 0 | 0 |
|  | *hsa-miR-92a-3p.3.P0.S.14* | 0 | 4 | 0 | 2 |
|  | *hsa-miR-92a-3p.3.P0.S.15* | 13 | 99 | 38 | 24 |
|  | *hsa-miR-92a-3p.3.P0.S.16* | 0 | 39 | 15 | 8 |
|  | *hsa-miR-92a-3p.3.P0.S.17* | 0 | 29 | 5 | 12 |
|  | *hsa-miR-92a-3p.3.P1.S.26* | 0 | 18 | 10 | 5 |
|  | *hsa-miR-92a-3p.3.P1.S.27* | 0 | 9 | 0 | 1 |
|  | *hsa-miR-92a-3p.3.P0.S.18* | 0 | 77 | 20 | 9 |
|  | *hsa-miR-92a-3p.3.P1.S.28* | 0 | 0 | 0 | 1 |
|  | *hsa-miR-92a-3p.3.P1.S.29* | 0 | 0 | 0 | 1 |
|  | *hsa-miR-92a-3p.3.P1.S.30* | 0 | 31 | 3 | 6 |
|  | *hsa-miR-92a-3p.3.P1.S.31* | 0 | 5 | 0 | 0 |
|  | *hsa-miR-92a-3p.3.P1.S.32* | 0 | 39 | 5 | 5 |
|  | *hsa-miR-92a-3p.53.P0.S.14* | 0 | 0 | 7 | 0 |
|  | *hsa-miR-92a-3p.53.P0.S.15* | 7 | 7 | 0 | 0 |
|  | *hsa-miR-92a-3p.53.P0.S.16* | 0 | 0 | 10 | 0 |
|  | *hsa-miR-92a-3p.53.P0.S.17* | 0 | 5 | 0 | 1 |
| ***hsa-miR-192-5p*** | *hsa-miR-192-5p.3.P0.S.0* | 125 | 432 | 474 | 74 |
|  | *hsa-miR-192-5p.3.P0.S.4* | 49 | 182 | 86 | 24 |
|  | *hsa-miR-192-5p.3.P0.S.8* | 755 | 1674 | 1144 | 405 |
|  | *hsa-miR-192-5p.3.P0.S.9* | 139 | 86 | 34 | 35 |
|  | *hsa-miR-192-5p.3.P0.S.12* | 36 | 62 | 124 | 8 |
|  | *hsa-miR-192-5p.3.P0.S.14* | 512 | 647 | 623 | 92 |
|  | *hsa-miR-192-5p.3.P0.S.15* | 622 | 521 | 780 | 172 |
|  | *hsa-miR-192-5p.3.P0.S.19* | 11359 | 14856 | 20585 | 3177 |
|  | *hsa-miR-192-5p.3.P0.S.20* | 618 | 642 | 758 | 416 |
|  | *hsa-miR-192-5p.3.P0.S.21* | 4094 | 5015 | 5280 | 1680 |
|  | *hsa-miR-192-5p.53.P0.S.7* | 898 | 2303 | 2358 | 589 |
|  | *hsa-miR-192-5p.53.P0.S.9* | 17 | 110 | 49 | 18 |
|  | *hsa-miR-192-5p.53.P0.S.22* | 9812 | 13014 | 20297 | 4460 |
|  | *hsa-miR-192-5p.53.P0.S.26* | 416 | 1403 | 1459 | 509 |
|  | *hsa-miR-192-5p.53.P0.S.27* | 5143 | 6162 | 7874 | 1620 |
|  | *hsa-miR-192-5p.5.P0.S.1* | 1741 | 2006 | 1633 | 489 |
|  | *hsa-miR-192-5p.53.P0.S.31* | 66 | 55 | 115 | 17 |
|  | *hsa-miR-192-5p.0.P1.S.35* | 111 | 106 | 96 | 35 |
| ***hsa-miR-103a-3p*** | *hsa-miR-103a-3p.3.P0.M.4* | 370 | 1924 | 591 | 917 |
|  | *hsa-miR-103a-3p.3.P0.M.5* | 2951 | 4533 | 2479 | 3174 |
|  | *hsa-miR-103a-3p.3.P0.M.6* | 394 | 684 | 351 | 763 |
|  | *hsa-miR-103a-3p.3.P1.M.41* | 190 | 213 | 81 | 82 |
|  | *hsa-miR-103a-3p.3.P1.M.43* | 382 | 183 | 275 | 154 |
|  | *hsa-miR-103a-3p.3.P0.M.7* | 473 | 476 | 871 | 475 |
|  | *hsa-miR-103a-3p.3.P0.M.8* | 5552 | 3570 | 7516 | 5303 |
|  | *hsa-miR-103a-3p.3.P0.M.9* | 290 | 196 | 760 | 115 |
|  | *hsa-miR-103a-3p.3.P0.M.10* | 276 | 285 | 373 | 384 |
|  | *hsa-miR-103a-3p.5.P0.M.2* | 780 | 1066 | 2284 | 601 |
|  | *hsa-miR-103a-3p.0.P1.M.58* | 745 | 670 | 380 | 1832 |
|  | *hsa-miR-103a-3p.0.P1.M.59* | 219 | 189 | 125 | 123 |
|  | *hsa-miR-103a-3p.0.P1.M.60* | 3678 | 3925 | 1933 | 2416 |
| ***hsa-miR-21-5p*** | *hsa-miR-21-5p.3.P0.S.0* | 1095 | 815 | 245 | 1137 |
|  | *hsa-miR-21-5p.3.P0.S.4* | 4026 | 1843 | 4476 | 12712 |
|  | *hsa-miR-21-5p.3.P0.S.5* | 134 | 570 | 236 | 1258 |
|  | *hsa-miR-21-5p.3.P0.S.6* | 20 | 13 | 98 | 152 |
|  | *hsa-miR-21-5p.3.P0.S.16* | 19 | 289 | 46 | 40 |
|  | *hsa-miR-21-5p.3.P0.S.19* | 3061 | 748 | 5608 | 716 |
|  | *hsa-miR-21-5p.3.P1.S.32* | 121 | 53 | 177 | 13 |
|  | *hsa-miR-21-5p.3.P0.S.20* | 51157 | 114113 | 51853 | 118263 |
|  | *hsa-miR-21-5p.3.P1.S.56* | 112 | 33 | 43 | 25 |
|  | *hsa-miR-21-5p.3.P1.S.58* | 112 | 13 | 62 | 14 |
|  | *hsa-miR-21-5p.3.P1.S.66* | 351 | 100 | 312 | 195 |
|  | *hsa-miR-21-5p.3.P1.S.74* | 164 | 68 | 209 | 145 |
|  | *hsa-miR-21-5p.3.P1.S.76* | 114 | 70 | 167 | 79 |
|  | *hsa-miR-21-5p.3.P1.S.79* | 42 | 153 | 13 | 53 |
|  | *hsa-miR-21-5p.3.P1.S.81* | 52 | 237 | 61 | 153 |
|  | *hsa-miR-21-5p.3.P1.S.87* | 581 | 11 | 231 | 1 |
|  | *hsa-miR-21-5p.3.P0.S.21* | 125 | 49 | 211 | 45 |
|  | *hsa-miR-21-5p.3.P0.S.31* | 4088 | 1841 | 4546 | 5407 |
|  | *hsa-miR-21-5p.3.P0.S.32* | 7455 | 990 | 10854 | 347 |
|  | *hsa-miR-21-5p.3.P0.S.33* | 2492 | 389 | 1862 | 53 |
|  | *hsa-miR-21-5p.3.P1.S.208* | 94 | 34 | 215 | 58 |
|  | *hsa-miR-21-5p.3.P0.S.34* | 8040 | 2576 | 15351 | 1345 |
|  | *hsa-miR-21-5p.3.P1.S.227* | 175 | 41 | 259 | 10 |
|  | *hsa-miR-21-5p.3.P1.S.228* | 76 | 17 | 130 | 3 |
|  | *hsa-miR-21-5p.3.P1.S.229* | 155 | 26 | 203 | 3 |
|  | *hsa-miR-21-5p.3.P1.S.234* | 98 | 67 | 398 | 27 |
|  | *hsa-miR-21-5p.3.P1.S.236* | 29 | 16 | 124 | 38 |
|  | *hsa-miR-21-5p.3.P1.S.246* | 75 | 11 | 201 | 16 |
|  | *hsa-miR-21-5p.3.P1.S.247* | 104 | 29 | 242 | 13 |
|  | *hsa-miR-21-5p.3.P1.S.248* | 177 | 62 | 648 | 76 |
|  | *hsa-miR-21-5p.3.P1.S.249* | 36 | 18 | 116 | 2 |
|  | *hsa-miR-21-5p.53.P0.S.12* | 59 | 29 | 290 | 108 |
|  | *hsa-miR-21-5p.53.P0.S.13* | 226 | 263 | 407 | 723 |
|  | *hsa-miR-21-5p.53.P0.S.15* | 10 | 13 | 100 | 23 |
|  | *hsa-miR-21-5p.5.P0.S.3* | 982 | 401 | 2172 | 1763 |
|  | *hsa-miR-21-5p.53.P0.S.23* | 121 | 107 | 304 | 417 |
|  | *hsa-miR-21-5p.53.P0.S.29* | 92 | 55 | 644 | 120 |
|  | *hsa-miR-21-5p.53.P0.S.30* | 2701 | 2628 | 3864 | 4256 |
|  | *hsa-miR-21-5p.53.P0.S.33* | 154 | 105 | 255 | 195 |
|  | *hsa-miR-21-5p.53.P0.S.34* | 95 | 51 | 240 | 22 |
|  | *hsa-miR-21-5p.5.P0.S.7* | 925 | 472 | 2133 | 939 |
|  | *hsa-miR-21-5p.0.P1.S.26* | 143 | 18 | 69 | 25 |
|  | *hsa-miR-21-5p.0.P1.S.36* | 78 | 13 | 168 | 14 |
|  | *hsa-miR-21-5p.0.P1.S.37* | 26 | 9 | 193 | 16 |
|  | *hsa-miR-21-5p.0.P1.S.41* | 59 | 18 | 132 | 66 |
|  | *hsa-miR-21-5p.0.P1.S.47* | 548 | 66 | 500 | 12 |
|  | *hsa-miR-21-5p.0.P1.S.48* | 280 | 29 | 321 | 46 |
|  | *hsa-miR-21-5p.0.P1.S.49* | 215 | 16 | 309 | 15 |
| ***hsa-miR-29a-3p*** | *hsa-miR-29a-3p.3.P0.S.0* | 676 | 146 | 782 | 1514 |
|  | *hsa-miR-29a-3p.3.P0.S.3* | 8510 | 1864 | 24931 | 10144 |
|  | *hsa-miR-29a-3p.3.P1.S.21* | 3 | 4 | 211 | 18 |
|  | *hsa-miR-29a-3p.3.P1.S.23* | 3 | 0 | 388 | 6 |
|  | *hsa-miR-29a-3p.3.P0.S.4* | 55 | 49 | 188 | 119 |
|  | *hsa-miR-29a-3p.3.P0.S.5* | 437 | 171 | 816 | 320 |
|  | *hsa-miR-29a-3p.3.P0.S.6* | 232 | 133 | 443 | 790 |
|  | *hsa-miR-29a-3p.53.P0.S.5* | 694 | 261 | 2524 | 1400 |
|  | *hsa-miR-29a-3p.5.P0.S.0* | 259 | 149 | 566 | 828 |
|  | *hsa-miR-29a-3p.5.P1.S.11* | 107 | 0 | 76 | 130 |
|  | *hsa-miR-29a-3p.53.P0.S.13* | 177 | 64 | 553 | 222 |
|  | *hsa-miR-29a-3p.5.P0.S.4* | 289 | 153 | 694 | 530 |
|  | *hsa-miR-29a-3p.0.P1.S.21* | 26 | 18 | 159 | 14 |
|  | *hsa-miR-29a-3p.0.P1.S.41* | 274 | 35 | 179 | 304 |
| ***hsa-miR-93-5p*** | *hsa-miR-93-5p.3.P0.S.1* | 271 | 70 | 177 | 42 |
|  | *hsa-miR-93-5p.3.P0.S.2* | 0 | 68 | 3 | 1 |
|  | *hsa-miR-93-5p.3.P0.S.3* | 3 | 0 | 0 | 0 |
|  | *hsa-miR-93-5p.3.P0.S.4* | 3 | 2 | 0 | 0 |
|  | *hsa-miR-93-5p.3.P0.S.5* | 0 | 0 | 3 | 0 |
|  | *hsa-miR-93-5p.3.P0.S.6* | 0 | 0 | 3 | 0 |
|  | *hsa-miR-93-5p.3.P0.S.7* | 0 | 0 | 10 | 1 |
|  | *hsa-miR-93-5p.3.P0.S.8* | 1341 | 847 | 2355 | 335 |
|  | *hsa-miR-93-5p.3.P1.S.0* | 0 | 0 | 3 | 0 |
|  | *hsa-miR-93-5p.3.P1.S.1* | 0 | 0 | 0 | 1 |
|  | *hsa-miR-93-5p.3.P1.S.2* | 0 | 2 | 0 | 1 |
|  | *hsa-miR-93-5p.3.P1.S.3* | 3 | 0 | 0 | 0 |
|  | *hsa-miR-93-5p.3.P1.S.4* | 0 | 0 | 0 | 1 |
|  | *hsa-miR-93-5p.3.P1.S.5* | 10 | 0 | 5 | 0 |
|  | *hsa-miR-93-5p.3.P1.S.6* | 19 | 0 | 3 | 0 |
|  | *hsa-miR-93-5p.3.P1.S.7* | 0 | 0 | 5 | 1 |
|  | *hsa-miR-93-5p.3.P1.S.8* | 3 | 0 | 0 | 0 |
|  | *hsa-miR-93-5p.3.P1.S.9* | 19 | 0 | 0 | 0 |
|  | *hsa-miR-93-5p.3.P1.S.10* | 0 | 0 | 3 | 0 |
|  | *hsa-miR-93-5p.3.P1.S.11* | 0 | 5 | 0 | 0 |
|  | *hsa-miR-93-5p.3.P1.S.12* | 0 | 11 | 5 | 2 |
|  | *hsa-miR-93-5p.3.P1.S.13* | 3 | 0 | 7 | 0 |
|  | *hsa-miR-93-5p.3.P1.S.14* | 0 | 0 | 5 | 2 |
|  | *hsa-miR-93-5p.3.P1.S.15* | 0 | 0 | 3 | 0 |
|  | *hsa-miR-93-5p.3.P1.S.16* | 0 | 0 | 5 | 0 |
|  | *hsa-miR-93-5p.3.P1.S.17* | 0 | 0 | 12 | 0 |
|  | *hsa-miR-93-5p.3.P1.S.18* | 0 | 0 | 0 | 1 |
|  | *hsa-miR-93-5p.3.P1.S.19* | 13 | 9 | 25 | 19 |
|  | *hsa-miR-93-5p.3.P1.S.20* | 3 | 174 | 15 | 5 |
|  | *hsa-miR-93-5p.3.P1.S.21* | 7 | 0 | 20 | 1 |
|  | *hsa-miR-93-5p.3.P0.S.9* | 55 | 35 | 480 | 1 |
|  | *hsa-miR-93-5p.3.P1.S.22* | 3 | 0 | 0 | 0 |
|  | *hsa-miR-93-5p.3.P1.S.23* | 3 | 0 | 0 | 0 |
|  | *hsa-miR-93-5p.3.P1.S.24* | 0 | 0 | 7 | 0 |
|  | *hsa-miR-93-5p.3.P1.S.25* | 0 | 0 | 3 | 0 |
|  | *hsa-miR-93-5p.3.P1.S.26* | 0 | 2 | 14 | 0 |
|  | *hsa-miR-93-5p.3.P1.S.27* | 7 | 7 | 49 | 1 |
|  | *hsa-miR-93-5p.3.P1.S.28* | 0 | 9 | 164 | 2 |
|  | *hsa-miR-93-5p.3.P0.S.10* | 3175 | 2814 | 2337 | 787 |
|  | *hsa-miR-93-5p.3.P1.S.29* | 0 | 0 | 10 | 0 |
|  | *hsa-miR-93-5p.3.P1.S.30* | 0 | 2 | 0 | 0 |
|  | *hsa-miR-93-5p.3.P1.S.31* | 7 | 0 | 0 | 2 |
|  | *hsa-miR-93-5p.3.P1.S.32* | 3 | 5 | 0 | 0 |
|  | *hsa-miR-93-5p.3.P1.S.33* | 3 | 0 | 0 | 0 |
|  | *hsa-miR-93-5p.3.P1.S.34* | 3 | 0 | 3 | 2 |
|  | *hsa-miR-93-5p.3.P1.S.35* | 0 | 0 | 0 | 1 |
|  | *hsa-miR-93-5p.3.P1.S.36* | 3 | 0 | 0 | 1 |
|  | *hsa-miR-93-5p.3.P1.S.37* | 3 | 0 | 0 | 0 |
|  | *hsa-miR-93-5p.3.P1.S.38* | 0 | 5 | 0 | 0 |
|  | *hsa-miR-93-5p.3.P1.S.39* | 0 | 0 | 5 | 0 |
|  | *hsa-miR-93-5p.3.P1.S.40* | 0 | 2 | 0 | 0 |
|  | *hsa-miR-93-5p.3.P1.S.41* | 0 | 0 | 0 | 1 |
|  | *hsa-miR-93-5p.3.P1.S.42* | 0 | 0 | 0 | 1 |
|  | *hsa-miR-93-5p.3.P1.S.43* | 3 | 5 | 0 | 0 |
|  | *hsa-miR-93-5p.3.P1.S.44* | 0 | 0 | 0 | 2 |
|  | *hsa-miR-93-5p.3.P1.S.45* | 3 | 2 | 0 | 0 |
|  | *hsa-miR-93-5p.3.P1.S.46* | 10 | 9 | 0 | 1 |
|  | *hsa-miR-93-5p.3.P1.S.47* | 0 | 7 | 0 | 0 |
|  | *hsa-miR-93-5p.3.P1.S.48* | 0 | 4 | 0 | 0 |
|  | *hsa-miR-93-5p.3.P1.S.49* | 0 | 2 | 0 | 0 |
|  | *hsa-miR-93-5p.3.P1.S.50* | 0 | 2 | 0 | 0 |
|  | *hsa-miR-93-5p.3.P1.S.51* | 0 | 0 | 3 | 0 |
|  | *hsa-miR-93-5p.3.P1.S.52* | 0 | 0 | 0 | 1 |
|  | *hsa-miR-93-5p.3.P1.S.53* | 3 | 0 | 0 | 0 |
|  | *hsa-miR-93-5p.3.P1.S.54* | 3 | 9 | 3 | 0 |
|  | *hsa-miR-93-5p.3.P1.S.55* | 0 | 0 | 0 | 1 |
|  | *hsa-miR-93-5p.3.P1.S.56* | 0 | 0 | 10 | 0 |
|  | *hsa-miR-93-5p.3.P1.S.57* | 0 | 4 | 13 | 0 |
|  | *hsa-miR-93-5p.3.P1.S.58* | 0 | 9 | 3 | 0 |
|  | *hsa-miR-93-5p.3.P1.S.59* | 0 | 2 | 0 | 0 |
|  | *hsa-miR-93-5p.3.P1.S.60* | 3 | 7 | 39 | 0 |
|  | *hsa-miR-93-5p.3.P1.S.61* | 19 | 0 | 0 | 2 |
|  | *hsa-miR-93-5p.3.P1.S.62* | 0 | 0 | 5 | 2 |
|  | *hsa-miR-93-5p.3.P1.S.63* | 0 | 0 | 3 | 0 |
|  | *hsa-miR-93-5p.3.P1.S.64* | 3 | 5 | 3 | 0 |
|  | *hsa-miR-93-5p.3.P1.S.65* | 7 | 5 | 12 | 0 |
|  | *hsa-miR-93-5p.3.P1.S.66* | 33 | 36 | 22 | 3 |
|  | *hsa-miR-93-5p.3.P0.S.11* | 222 | 178 | 474 | 28 |
|  | *hsa-miR-93-5p.3.P1.S.67* | 0 | 2 | 0 | 0 |
|  | *hsa-miR-93-5p.3.P1.S.68* | 3 | 0 | 0 | 0 |
|  | *hsa-miR-93-5p.3.P1.S.69* | 3 | 0 | 0 | 0 |
|  | *hsa-miR-93-5p.3.P1.S.70* | 0 | 0 | 5 | 0 |
|  | *hsa-miR-93-5p.3.P1.S.71* | 0 | 0 | 5 | 2 |
|  | *hsa-miR-93-5p.3.P1.S.72* | 0 | 0 | 0 | 1 |
|  | *hsa-miR-93-5p.3.P1.S.73* | 0 | 0 | 7 | 0 |
|  | *hsa-miR-93-5p.3.P1.S.74* | 3 | 2 | 12 | 0 |
|  | *hsa-miR-93-5p.3.P0.S.12* | 224 | 153 | 221 | 108 |
|  | *hsa-miR-93-5p.3.P1.S.75* | 3 | 0 | 0 | 0 |
|  | *hsa-miR-93-5p.3.P1.S.76* | 0 | 2 | 0 | 0 |
|  | *hsa-miR-93-5p.3.P1.S.77* | 0 | 0 | 0 | 1 |
|  | *hsa-miR-93-5p.3.P1.S.78* | 0 | 0 | 0 | 1 |
|  | *hsa-miR-93-5p.3.P1.S.79* | 3 | 0 | 0 | 0 |
|  | *hsa-miR-93-5p.3.P1.S.80* | 0 | 0 | 0 | 2 |
|  | *hsa-miR-93-5p.3.P1.S.81* | 0 | 4 | 0 | 0 |
|  | *hsa-miR-93-5p.3.P1.S.82* | 0 | 0 | 0 | 1 |
|  | *hsa-miR-93-5p.3.P1.S.83* | 3 | 0 | 0 | 0 |
|  | *hsa-miR-93-5p.3.P1.S.84* | 0 | 2 | 0 | 0 |
|  | *hsa-miR-93-5p.3.P1.S.85* | 0 | 0 | 0 | 1 |
|  | *hsa-miR-93-5p.3.P1.S.86* | 7 | 5 | 7 | 8 |
|  | *hsa-miR-93-5p.3.P0.S.13* | 13 | 13 | 13 | 5 |
|  | *hsa-miR-93-5p.3.P1.S.87* | 3 | 0 | 0 | 0 |
|  | *hsa-miR-93-5p.3.P1.S.88* | 3 | 0 | 0 | 0 |
|  | *hsa-miR-93-5p.3.P0.S.14* | 0 | 9 | 0 | 0 |
|  | *hsa-miR-93-5p.3.P0.S.15* | 0 | 5 | 0 | 0 |
|  | *hsa-miR-93-5p.3.P0.S.16* | 59 | 4 | 19 | 5 |
|  | *hsa-miR-93-5p.3.P0.S.17* | 693 | 305 | 773 | 269 |
|  | *hsa-miR-93-5p.3.P1.S.89* | 3 | 0 | 0 | 0 |
|  | *hsa-miR-93-5p.3.P1.S.90* | 23 | 0 | 3 | 0 |
|  | *hsa-miR-93-5p.3.P1.S.91* | 0 | 0 | 0 | 2 |
|  | *hsa-miR-93-5p.3.P1.S.92* | 3 | 0 | 0 | 0 |
|  | *hsa-miR-93-5p.3.P1.S.93* | 3 | 0 | 0 | 0 |
|  | *hsa-miR-93-5p.3.P1.S.94* | 0 | 0 | 0 | 2 |
|  | *hsa-miR-93-5p.3.P1.S.95* | 39 | 0 | 0 | 0 |
|  | *hsa-miR-93-5p.3.P1.S.96* | 0 | 5 | 0 | 0 |
|  | *hsa-miR-93-5p.3.P1.S.97* | 0 | 0 | 7 | 1 |
|  | *hsa-miR-93-5p.3.P1.S.98* | 0 | 0 | 5 | 0 |
|  | *hsa-miR-93-5p.3.P1.S.99* | 0 | 0 | 3 | 0 |
|  | *hsa-miR-93-5p.3.P1.S.100* | 0 | 0 | 0 | 2 |
|  | *hsa-miR-93-5p.3.P1.S.101* | 0 | 0 | 0 | 1 |
|  | *hsa-miR-93-5p.3.P1.S.102* | 0 | 0 | 3 | 0 |
|  | *hsa-miR-93-5p.3.P1.S.103* | 0 | 2 | 0 | 0 |
|  | *hsa-miR-93-5p.3.P1.S.104* | 0 | 0 | 12 | 3 |
|  | *hsa-miR-93-5p.3.P1.S.105* | 0 | 0 | 0 | 3 |
|  | *hsa-miR-93-5p.3.P1.S.106* | 0 | 0 | 3 | 0 |
|  | *hsa-miR-93-5p.5.P0.S.0* | 0 | 0 | 3 | 1 |
|  | *hsa-miR-93-5p.53.P0.S.0* | 0 | 0 | 5 | 0 |
|  | *hsa-miR-93-5p.53.P0.S.1* | 0 | 2 | 0 | 2 |
|  | *hsa-miR-93-5p.53.P0.S.2* | 0 | 0 | 3 | 0 |
|  | *hsa-miR-93-5p.53.P0.S.3* | 3 | 0 | 0 | 1 |
|  | *hsa-miR-93-5p.53.P0.S.4* | 7 | 0 | 0 | 0 |
|  | *hsa-miR-93-5p.53.P0.S.5* | 3 | 0 | 0 | 0 |
|  | *hsa-miR-93-5p.53.P0.S.6* | 7 | 9 | 0 | 2 |
|  | *hsa-miR-93-5p.53.P0.S.7* | 0 | 0 | 5 | 0 |
|  | *hsa-miR-93-5p.53.P0.S.8* | 0 | 2 | 0 | 0 |
|  | *hsa-miR-93-5p.5.P0.S.1* | 0 | 2 | 0 | 0 |
|  | *hsa-miR-93-5p.53.P0.S.9* | 13 | 0 | 10 | 1 |
|  | *hsa-miR-93-5p.53.P0.S.10* | 7 | 0 | 24 | 0 |
|  | *hsa-miR-93-5p.53.P0.S.11* | 0 | 0 | 5 | 1 |
|  | *hsa-miR-93-5p.5.P0.S.2* | 0 | 2 | 0 | 0 |
|  | *hsa-miR-93-5p.5.P1.S.0* | 0 | 0 | 5 | 0 |
|  | *hsa-miR-93-5p.5.P0.S.3* | 3 | 2 | 0 | 1 |
|  | *hsa-miR-93-5p.5.P0.S.4* | 335 | 54 | 120 | 74 |
|  | *hsa-miR-93-5p.5.P1.S.1* | 3 | 0 | 0 | 0 |
|  | *hsa-miR-93-5p.5.P1.S.2* | 3 | 0 | 0 | 1 |
|  | *hsa-miR-93-5p.5.P1.S.3* | 0 | 0 | 3 | 1 |
|  | *hsa-miR-93-5p.0.P1.S.0* | 0 | 0 | 0 | 1 |
|  | *hsa-miR-93-5p.0.P1.S.1* | 19 | 0 | 8 | 3 |
|  | *hsa-miR-93-5p.0.P1.S.2* | 0 | 0 | 5 | 1 |
|  | *hsa-miR-93-5p.0.P1.S.3* | 7 | 7 | 8 | 8 |
|  | *hsa-miR-93-5p.0.P1.S.4* | 52 | 31 | 18 | 4 |
|  | *hsa-miR-93-5p.0.P1.S.5* | 36 | 9 | 13 | 11 |
|  | *hsa-miR-93-5p.0.P1.S.6* | 10 | 11 | 15 | 6 |
|  | *hsa-miR-93-5p.0.P1.S.7* | 33 | 4 | 18 | 6 |
|  | *hsa-miR-93-5p.0.P1.S.8* | 10 | 0 | 0 | 2 |
|  | *hsa-miR-93-5p.0.P1.S.9* | 3 | 0 | 0 | 1 |
|  | *hsa-miR-93-5p.0.P1.S.10* | 23 | 0 | 17 | 1 |
|  | *hsa-miR-93-5p.0.P1.S.11* | 0 | 0 | 7 | 5 |
|  | *hsa-miR-93-5p.0.P1.S.12* | 115 | 22 | 58 | 22 |
|  | *hsa-miR-93-5p.0.P1.S.13* | 3 | 5 | 0 | 1 |
|  | *hsa-miR-93-5p.0.P1.S.14* | 43 | 38 | 27 | 8 |
|  | *hsa-miR-93-5p.0.P1.S.15* | 7 | 0 | 0 | 0 |
|  | *hsa-miR-93-5p.0.P1.S.16* | 19 | 4 | 0 | 0 |
|  | *hsa-miR-93-5p.0.P1.S.17* | 0 | 0 | 3 | 0 |
|  | *hsa-miR-93-5p.0.P1.S.18* | 0 | 2 | 0 | 1 |
|  | *hsa-miR-93-5p.0.P1.S.19* | 23 | 16 | 43 | 4 |
|  | *hsa-miR-93-5p.0.P1.S.20* | 3 | 0 | 0 | 0 |
|  | *hsa-miR-93-5p.0.P1.S.21* | 72 | 11 | 43 | 12 |
|  | *hsa-miR-93-5p.0.P1.S.22* | 0 | 0 | 5 | 1 |
|  | *hsa-miR-93-5p.0.P1.S.23* | 36 | 18 | 27 | 15 |
|  | *hsa-miR-93-5p.0.P1.S.24* | 0 | 0 | 0 | 2 |
|  | *hsa-miR-93-5p.0.P1.S.25* | 0 | 0 | 0 | 1 |
|  | *hsa-miR-93-5p.0.P1.S.26* | 7 | 5 | 0 | 3 |
|  | *hsa-miR-93-5p.0.P1.S.27* | 59 | 25 | 22 | 15 |
|  | *hsa-miR-93-5p.0.P1.S.28* | 19 | 5 | 20 | 1 |
|  | *hsa-miR-93-5p.0.P1.S.29* | 7 | 18 | 8 | 2 |
|  | *hsa-miR-93-5p.0.P1.S.30* | 40 | 9 | 15 | 6 |
|  | *hsa-miR-93-5p.0.P1.S.31* | 143 | 72 | 28 | 16 |
|  | *hsa-miR-93-5p.0.P1.S.32* | 3 | 4 | 0 | 0 |
|  | *hsa-miR-93-5p.0.P1.S.33* | 0 | 2 | 0 | 0 |
|  | *hsa-miR-93-5p.0.P1.S.34* | 56 | 29 | 35 | 4 |
|  | *hsa-miR-93-5p.0.P1.S.35* | 71 | 9 | 29 | 4 |
|  | *hsa-miR-93-5p.0.P1.S.36* | 3 | 0 | 5 | 2 |
|  | *hsa-miR-93-5p.0.P1.S.37* | 10 | 2 | 3 | 0 |
|  | *hsa-miR-93-5p.0.P1.S.38* | 30 | 9 | 24 | 5 |
|  | *hsa-miR-93-5p.0.P1.S.39* | 0 | 0 | 7 | 0 |
|  | *hsa-miR-93-5p.0.P1.S.40* | 26 | 0 | 0 | 2 |
|  | *hsa-miR-93-5p.0.P1.S.41* | 7 | 0 | 3 | 2 |
|  | *hsa-miR-93-5p.0.P1.S.42* | 0 | 5 | 12 | 0 |
|  | *hsa-miR-93-5p.0.P1.S.43* | 19 | 0 | 0 | 1 |
|  | *hsa-miR-93-5p.0.P1.S.44* | 49 | 9 | 7 | 2 |
|  | *hsa-miR-93-5p.0.P1.S.45* | 13 | 11 | 15 | 6 |
|  | *hsa-miR-93-5p.0.P1.S.46* | 3 | 7 | 29 | 0 |
|  | *hsa-miR-93-5p.0.P1.S.47* | 66 | 13 | 41 | 14 |
|  | *hsa-miR-93-5p.0.P1.S.48* | 17 | 16 | 39 | 0 |
|  | *hsa-miR-93-5p.0.P1.S.49* | 0 | 0 | 10 | 0 |
|  | *hsa-miR-93-5p.0.P1.S.50* | 0 | 0 | 7 | 0 |
|  | *hsa-miR-93-5p.0.P1.S.51* | 72 | 9 | 1364 | 3 |
|  | *hsa-miR-93-5p.0.P1.S.52* | 46 | 5 | 20 | 2 |
|  | *hsa-miR-93-5p.0.P1.S.53* | 19 | 0 | 10 | 1 |
|  | *hsa-miR-93-5p.0.P1.S.54* | 40 | 2 | 27 | 7 |
|  | *hsa-miR-93-5p.0.P1.S.55* | 10 | 0 | 3 | 2 |
|  | *hsa-miR-93-5p.0.P1.S.56* | 3 | 0 | 0 | 1 |
|  | *hsa-miR-93-5p.0.P1.S.57* | 3 | 0 | 0 | 0 |
|  | *hsa-miR-93-5p.0.P1.S.58* | 7 | 0 | 8 | 3 |
|  | *hsa-miR-93-5p.0.P1.S.59* | 3 | 5 | 0 | 3 |
|  | *hsa-miR-93-5p.0.P1.S.60* | 425 | 298 | 578 | 242 |
|  | *hsa-miR-93-5p.0.P1.S.61* | 3262 | 1474 | 2199 | 698 |
|  | *hsa-miR-93-5p.0.P1.S.62* | 397 | 350 | 500 | 281 |
| ***hsa-miR-451a*** | *hsa-miR-451a.3.P0.S.0* | 49 | 64 | 308 | 5 |
|  | *hsa-miR-451a.3.P0.S.1* | 7 | 5 | 0 | 0 |
|  | *hsa-miR-451a.3.P0.S.2* | 13 | 437 | 185 | 3 |
|  | *hsa-miR-451a.3.P0.S.3* | 0 | 2 | 0 | 0 |
|  | *hsa-miR-451a.3.P0.S.4* | 30 | 72 | 218 | 6 |
|  | *hsa-miR-451a.3.P0.S.5* | 3 | 7 | 56 | 2 |
|  | *hsa-miR-451a.3.P0.S.6* | 3 | 0 | 0 | 0 |
|  | *hsa-miR-451a.3.P0.S.7* | 19 | 0 | 12 | 0 |
|  | *hsa-miR-451a.3.P0.S.8* | 33 | 43 | 108 | 0 |
|  | *hsa-miR-451a.3.P0.S.9* | 0 | 41 | 10 | 2 |
|  | *hsa-miR-451a.3.P0.S.10* | 0 | 0 | 3 | 0 |
|  | *hsa-miR-451a.3.P0.S.11* | 7 | 38 | 42 | 0 |
|  | *hsa-miR-451a.3.P0.S.12* | 7 | 2 | 21 | 0 |
|  | *hsa-miR-451a.3.P0.S.13* | 0 | 5 | 0 | 0 |
|  | *hsa-miR-451a.3.P0.S.14* | 23 | 61 | 228 | 13 |
|  | *hsa-miR-451a.3.P0.S.15* | 3 | 13 | 12 | 4 |
|  | *hsa-miR-451a.3.P0.S.16* | 0 | 25 | 43 | 6 |
|  | *hsa-miR-451a.3.P0.S.17* | 0 | 0 | 7 | 0 |
|  | *hsa-miR-451a.3.P0.S.18* | 0 | 0 | 0 | 2 |
|  | *hsa-miR-451a.3.P0.S.19* | 19 | 29 | 23 | 4 |
|  | *hsa-miR-451a.3.P0.S.20* | 17 | 29 | 115 | 4 |
|  | *hsa-miR-451a.3.P0.S.21* | 3 | 29 | 48 | 1 |
|  | *hsa-miR-451a.3.P1.S.0* | 0 | 0 | 5 | 0 |
|  | *hsa-miR-451a.3.P1.S.1* | 0 | 0 | 0 | 1 |
|  | *hsa-miR-451a.3.P1.S.2* | 0 | 0 | 8 | 0 |
|  | *hsa-miR-451a.3.P1.S.3* | 0 | 5 | 0 | 0 |
|  | *hsa-miR-451a.3.P1.S.4* | 0 | 2 | 3 | 0 |
|  | *hsa-miR-451a.3.P1.S.5* | 3 | 27 | 60 | 5 |
|  | *hsa-miR-451a.3.P0.S.22* | 3 | 27 | 107 | 15 |
|  | *hsa-miR-451a.3.P1.S.6* | 0 | 0 | 0 | 1 |
|  | *hsa-miR-451a.3.P1.S.7* | 0 | 0 | 0 | 1 |
|  | *hsa-miR-451a.3.P1.S.8* | 19 | 0 | 8 | 0 |
|  | *hsa-miR-451a.3.P1.S.9* | 0 | 0 | 3 | 0 |
|  | *hsa-miR-451a.3.P1.S.10* | 0 | 0 | 3 | 0 |
|  | *hsa-miR-451a.3.P1.S.11* | 0 | 0 | 5 | 0 |
|  | *hsa-miR-451a.3.P0.S.23* | 1113 | 1200 | 6873 | 461 |
|  | *hsa-miR-451a.3.P1.S.12* | 0 | 0 | 3 | 0 |
|  | *hsa-miR-451a.3.P1.S.13* | 0 | 0 | 3 | 0 |
|  | *hsa-miR-451a.3.P1.S.14* | 0 | 0 | 13 | 0 |
|  | *hsa-miR-451a.3.P1.S.15* | 0 | 0 | 5 | 0 |
|  | *hsa-miR-451a.3.P1.S.16* | 0 | 5 | 0 | 1 |
|  | *hsa-miR-451a.3.P1.S.17* | 0 | 0 | 3 | 0 |
|  | *hsa-miR-451a.3.P1.S.18* | 3 | 5 | 13 | 0 |
|  | *hsa-miR-451a.3.P1.S.19* | 0 | 0 | 5 | 0 |
|  | *hsa-miR-451a.3.P1.S.20* | 0 | 0 | 0 | 2 |
|  | *hsa-miR-451a.3.P1.S.21* | 0 | 0 | 3 | 0 |
|  | *hsa-miR-451a.3.P1.S.22* | 0 | 4 | 24 | 0 |
|  | *hsa-miR-451a.3.P1.S.23* | 3 | 9 | 42 | 0 |
|  | *hsa-miR-451a.3.P1.S.24* | 3 | 0 | 8 | 1 |
|  | *hsa-miR-451a.3.P1.S.25* | 0 | 0 | 25 | 0 |
|  | *hsa-miR-451a.3.P1.S.26* | 0 | 2 | 5 | 0 |
|  | *hsa-miR-451a.3.P1.S.27* | 0 | 0 | 15 | 0 |
|  | *hsa-miR-451a.3.P1.S.28* | 0 | 0 | 7 | 2 |
|  | *hsa-miR-451a.3.P1.S.29* | 0 | 0 | 0 | 2 |
|  | *hsa-miR-451a.3.P1.S.30* | 0 | 0 | 7 | 0 |
|  | *hsa-miR-451a.3.P1.S.31* | 0 | 0 | 0 | 2 |
|  | *hsa-miR-451a.3.P1.S.32* | 0 | 0 | 5 | 0 |
|  | *hsa-miR-451a.3.P1.S.33* | 0 | 0 | 7 | 0 |
|  | *hsa-miR-451a.3.P1.S.34* | 0 | 0 | 0 | 1 |
|  | *hsa-miR-451a.3.P1.S.35* | 0 | 5 | 0 | 0 |
|  | *hsa-miR-451a.3.P1.S.36* | 0 | 5 | 0 | 0 |
|  | *hsa-miR-451a.3.P1.S.37* | 0 | 0 | 12 | 0 |
|  | *hsa-miR-451a.3.P1.S.38* | 0 | 0 | 5 | 0 |
|  | *hsa-miR-451a.3.P0.S.24* | 0 | 4 | 27 | 2 |
|  | *hsa-miR-451a.3.P1.S.39* | 3 | 0 | 0 | 0 |
|  | *hsa-miR-451a.3.P0.S.25* | 0 | 5 | 0 | 0 |
|  | *hsa-miR-451a.3.P0.S.26* | 43 | 47 | 355 | 12 |
|  | *hsa-miR-451a.3.P1.S.40* | 0 | 0 | 5 | 0 |
|  | *hsa-miR-451a.3.P1.S.41* | 3 | 0 | 0 | 0 |
|  | *hsa-miR-451a.3.P1.S.42* | 3 | 0 | 0 | 0 |
|  | *hsa-miR-451a.3.P1.S.43* | 0 | 0 | 5 | 0 |
|  | *hsa-miR-451a.3.P0.S.27* | 10 | 16 | 36 | 4 |
|  | *hsa-miR-451a.3.P1.S.44* | 0 | 0 | 3 | 0 |
|  | *hsa-miR-451a.3.P0.S.28* | 437 | 471 | 1680 | 56 |
|  | *hsa-miR-451a.3.P1.S.45* | 0 | 0 | 3 | 0 |
|  | *hsa-miR-451a.3.P1.S.46* | 0 | 5 | 0 | 0 |
|  | *hsa-miR-451a.3.P1.S.47* | 0 | 0 | 10 | 1 |
|  | *hsa-miR-451a.3.P1.S.48* | 0 | 0 | 3 | 0 |
|  | *hsa-miR-451a.3.P1.S.49* | 0 | 0 | 3 | 0 |
|  | *hsa-miR-451a.3.P1.S.50* | 0 | 2 | 8 | 0 |
|  | *hsa-miR-451a.3.P1.S.51* | 0 | 0 | 5 | 0 |
|  | *hsa-miR-451a.3.P1.S.52* | 0 | 2 | 13 | 0 |
|  | *hsa-miR-451a.3.P1.S.53* | 0 | 0 | 5 | 0 |
|  | *hsa-miR-451a.3.P1.S.54* | 0 | 0 | 33 | 1 |
|  | *hsa-miR-451a.3.P1.S.55* | 0 | 0 | 5 | 0 |
|  | *hsa-miR-451a.3.P1.S.56* | 3 | 5 | 0 | 0 |
|  | *hsa-miR-451a.3.P1.S.57* | 0 | 0 | 3 | 0 |
|  | *hsa-miR-451a.3.P1.S.58* | 0 | 0 | 3 | 0 |
|  | *hsa-miR-451a.3.P1.S.59* | 0 | 0 | 5 | 0 |
|  | *hsa-miR-451a.3.P0.S.29* | 895 | 1022 | 4247 | 460 |
|  | *hsa-miR-451a.3.P1.S.60* | 0 | 0 | 5 | 0 |
|  | *hsa-miR-451a.3.P1.S.61* | 0 | 5 | 8 | 0 |
|  | *hsa-miR-451a.3.P1.S.62* | 0 | 0 | 5 | 0 |
|  | *hsa-miR-451a.3.P1.S.63* | 0 | 0 | 5 | 0 |
|  | *hsa-miR-451a.3.P1.S.64* | 0 | 5 | 0 | 0 |
|  | *hsa-miR-451a.3.P1.S.65* | 0 | 0 | 5 | 0 |
|  | *hsa-miR-451a.3.P1.S.66* | 0 | 2 | 0 | 0 |
|  | *hsa-miR-451a.3.P1.S.67* | 0 | 0 | 3 | 0 |
|  | *hsa-miR-451a.3.P1.S.68* | 0 | 0 | 5 | 0 |
|  | *hsa-miR-451a.3.P1.S.69* | 0 | 0 | 7 | 0 |
|  | *hsa-miR-451a.3.P1.S.70* | 26 | 28 | 66 | 0 |
|  | *hsa-miR-451a.3.P1.S.71* | 0 | 2 | 5 | 0 |
|  | *hsa-miR-451a.3.P1.S.72* | 0 | 2 | 34 | 0 |
|  | *hsa-miR-451a.3.P1.S.73* | 0 | 0 | 7 | 0 |
|  | *hsa-miR-451a.3.P1.S.74* | 0 | 0 | 5 | 0 |
|  | *hsa-miR-451a.3.P1.S.75* | 0 | 0 | 5 | 0 |
|  | *hsa-miR-451a.3.P1.S.76* | 0 | 0 | 0 | 2 |
|  | *hsa-miR-451a.3.P1.S.77* | 0 | 2 | 0 | 0 |
|  | *hsa-miR-451a.3.P1.S.78* | 0 | 0 | 5 | 1 |
|  | *hsa-miR-451a.3.P1.S.79* | 0 | 0 | 3 | 2 |
|  | *hsa-miR-451a.3.P1.S.80* | 0 | 5 | 7 | 1 |
|  | *hsa-miR-451a.3.P1.S.81* | 0 | 0 | 8 | 0 |
|  | *hsa-miR-451a.3.P0.S.30* | 551 | 332 | 2338 | 122 |
|  | *hsa-miR-451a.3.P0.S.31* | 6412 | 9222 | 47536 | 3190 |
|  | *hsa-miR-451a.3.P1.S.82* | 0 | 0 | 19 | 0 |
|  | *hsa-miR-451a.3.P1.S.83* | 0 | 0 | 7 | 0 |
|  | *hsa-miR-451a.3.P1.S.84* | 0 | 0 | 14 | 0 |
|  | *hsa-miR-451a.3.P1.S.85* | 0 | 0 | 40 | 2 |
|  | *hsa-miR-451a.3.P1.S.86* | 0 | 0 | 7 | 1 |
|  | *hsa-miR-451a.3.P1.S.87* | 0 | 0 | 3 | 0 |
|  | *hsa-miR-451a.3.P1.S.88* | 7 | 5 | 45 | 0 |
|  | *hsa-miR-451a.3.P1.S.89* | 0 | 5 | 0 | 0 |
|  | *hsa-miR-451a.3.P1.S.90* | 0 | 2 | 31 | 1 |
|  | *hsa-miR-451a.3.P1.S.91* | 0 | 0 | 0 | 2 |
|  | *hsa-miR-451a.3.P1.S.92* | 0 | 0 | 22 | 1 |
|  | *hsa-miR-451a.3.P1.S.93* | 0 | 5 | 17 | 0 |
|  | *hsa-miR-451a.3.P1.S.94* | 0 | 0 | 5 | 0 |
|  | *hsa-miR-451a.3.P1.S.95* | 3 | 0 | 15 | 2 |
|  | *hsa-miR-451a.3.P1.S.96* | 0 | 0 | 12 | 0 |
|  | *hsa-miR-451a.3.P1.S.97* | 7 | 9 | 49 | 1 |
|  | *hsa-miR-451a.3.P1.S.98* | 0 | 0 | 17 | 1 |
|  | *hsa-miR-451a.3.P1.S.99* | 3 | 5 | 13 | 1 |
|  | *hsa-miR-451a.3.P1.S.100* | 0 | 5 | 0 | 0 |
|  | *hsa-miR-451a.3.P1.S.101* | 0 | 0 | 60 | 1 |
|  | *hsa-miR-451a.3.P1.S.102* | 0 | 27 | 39 | 1 |
|  | *hsa-miR-451a.3.P1.S.103* | 0 | 0 | 10 | 0 |
|  | *hsa-miR-451a.3.P1.S.104* | 0 | 5 | 29 | 3 |
|  | *hsa-miR-451a.3.P1.S.105* | 0 | 0 | 5 | 2 |
|  | *hsa-miR-451a.3.P1.S.106* | 0 | 2 | 0 | 0 |
|  | *hsa-miR-451a.3.P1.S.107* | 0 | 0 | 13 | 0 |
|  | *hsa-miR-451a.3.P1.S.108* | 13 | 15 | 20 | 1 |
|  | *hsa-miR-451a.3.P1.S.109* | 63 | 192 | 231 | 0 |
|  | *hsa-miR-451a.3.P1.S.110* | 0 | 5 | 10 | 0 |
|  | *hsa-miR-451a.3.P1.S.111* | 17 | 9 | 50 | 2 |
|  | *hsa-miR-451a.3.P1.S.112* | 7 | 9 | 50 | 1 |
|  | *hsa-miR-451a.3.P1.S.113* | 36 | 42 | 255 | 7 |
|  | *hsa-miR-451a.3.P1.S.114* | 0 | 0 | 5 | 0 |
|  | *hsa-miR-451a.3.P1.S.115* | 19 | 9 | 14 | 0 |
|  | *hsa-miR-451a.3.P1.S.116* | 0 | 2 | 0 | 0 |
|  | *hsa-miR-451a.3.P1.S.117* | 0 | 2 | 26 | 3 |
|  | *hsa-miR-451a.3.P1.S.118* | 3 | 0 | 0 | 2 |
|  | *hsa-miR-451a.3.P1.S.119* | 0 | 0 | 7 | 0 |
|  | *hsa-miR-451a.3.P1.S.120* | 0 | 5 | 32 | 1 |
|  | *hsa-miR-451a.3.P1.S.121* | 3 | 0 | 0 | 0 |
|  | *hsa-miR-451a.3.P1.S.122* | 7 | 0 | 27 | 3 |
|  | *hsa-miR-451a.3.P1.S.123* | 0 | 0 | 3 | 0 |
|  | *hsa-miR-451a.3.P1.S.124* | 0 | 4 | 20 | 0 |
|  | *hsa-miR-451a.3.P1.S.125* | 0 | 2 | 0 | 0 |
|  | *hsa-miR-451a.3.P1.S.126* | 0 | 2 | 0 | 0 |
|  | *hsa-miR-451a.3.P1.S.127* | 0 | 31 | 32 | 2 |
|  | *hsa-miR-451a.3.P1.S.128* | 0 | 0 | 15 | 1 |
|  | *hsa-miR-451a.3.P1.S.129* | 3 | 0 | 0 | 0 |
|  | *hsa-miR-451a.3.P1.S.130* | 0 | 5 | 0 | 0 |
|  | *hsa-miR-451a.3.P1.S.131* | 0 | 0 | 24 | 0 |
|  | *hsa-miR-451a.3.P1.S.132* | 3 | 0 | 17 | 0 |
|  | *hsa-miR-451a.3.P1.S.133* | 0 | 0 | 7 | 0 |
|  | *hsa-miR-451a.3.P1.S.134* | 0 | 36 | 5 | 0 |
|  | *hsa-miR-451a.3.P1.S.135* | 3 | 22 | 68 | 4 |
|  | *hsa-miR-451a.3.P1.S.136* | 0 | 0 | 0 | 1 |
|  | *hsa-miR-451a.3.P1.S.137* | 7 | 5 | 19 | 1 |
|  | *hsa-miR-451a.3.P0.S.32* | 237 | 85 | 879 | 53 |
|  | *hsa-miR-451a.3.P1.S.138* | 0 | 0 | 0 | 2 |
|  | *hsa-miR-451a.3.P1.S.139* | 0 | 0 | 3 | 0 |
|  | *hsa-miR-451a.3.P1.S.140* | 0 | 0 | 3 | 0 |
|  | *hsa-miR-451a.3.P1.S.141* | 0 | 0 | 3 | 0 |
|  | *hsa-miR-451a.3.P1.S.142* | 0 | 0 | 3 | 0 |
|  | *hsa-miR-451a.3.P1.S.143* | 0 | 0 | 17 | 0 |
|  | *hsa-miR-451a.3.P1.S.144* | 0 | 0 | 3 | 0 |
|  | *hsa-miR-451a.3.P1.S.145* | 0 | 5 | 34 | 2 |
|  | *hsa-miR-451a.3.P1.S.146* | 0 | 0 | 10 | 0 |
|  | *hsa-miR-451a.53.P0.S.0* | 0 | 5 | 0 | 0 |
|  | *hsa-miR-451a.53.P0.S.1* | 0 | 0 | 3 | 0 |
|  | *hsa-miR-451a.53.P0.S.2* | 0 | 0 | 0 | 2 |
|  | *hsa-miR-451a.5.P0.S.0* | 0 | 5 | 0 | 0 |
|  | *hsa-miR-451a.53.P0.S.3* | 0 | 5 | 5 | 0 |
|  | *hsa-miR-451a.53.P0.S.4* | 0 | 5 | 20 | 1 |
|  | *hsa-miR-451a.53.P0.S.5* | 3 | 0 | 0 | 0 |
|  | *hsa-miR-451a.53.P0.S.6* | 0 | 9 | 8 | 0 |
|  | *hsa-miR-451a.53.P0.S.7* | 0 | 0 | 8 | 0 |
|  | *hsa-miR-451a.53.P0.S.8* | 0 | 22 | 75 | 2 |
|  | *hsa-miR-451a.53.P0.S.9* | 0 | 0 | 3 | 0 |
|  | *hsa-miR-451a.53.P0.S.10* | 0 | 0 | 5 | 0 |
|  | *hsa-miR-451a.53.P0.S.11* | 7 | 0 | 0 | 0 |
|  | *hsa-miR-451a.53.P0.S.12* | 0 | 0 | 3 | 0 |
|  | *hsa-miR-451a.53.P0.S.13* | 0 | 0 | 3 | 0 |
|  | *hsa-miR-451a.53.P0.S.14* | 7 | 0 | 0 | 0 |
|  | *hsa-miR-451a.53.P0.S.15* | 0 | 0 | 7 | 1 |
|  | *hsa-miR-451a.53.P0.S.16* | 7 | 16 | 35 | 1 |
|  | *hsa-miR-451a.53.P0.S.17* | 0 | 2 | 7 | 0 |
|  | *hsa-miR-451a.53.P0.S.18* | 7 | 0 | 0 | 0 |
|  | *hsa-miR-451a.53.P0.S.19* | 0 | 14 | 10 | 0 |
|  | *hsa-miR-451a.53.P0.S.20* | 0 | 0 | 7 | 0 |
|  | *hsa-miR-451a.53.P0.S.21* | 0 | 0 | 3 | 0 |
|  | *hsa-miR-451a.53.P0.S.22* | 0 | 0 | 3 | 0 |
|  | *hsa-miR-451a.53.P0.S.23* | 0 | 0 | 8 | 2 |
|  | *hsa-miR-451a.53.P0.S.24* | 0 | 0 | 3 | 0 |
|  | *hsa-miR-451a.53.P0.S.25* | 0 | 0 | 0 | 1 |
|  | *hsa-miR-451a.53.P0.S.26* | 0 | 2 | 54 | 4 |
|  | *hsa-miR-451a.53.P0.S.27* | 0 | 0 | 0 | 1 |
|  | *hsa-miR-451a.53.P0.S.28* | 0 | 0 | 7 | 0 |
|  | *hsa-miR-451a.53.P0.S.29* | 0 | 0 | 5 | 0 |
|  | *hsa-miR-451a.53.P0.S.30* | 0 | 0 | 0 | 2 |
|  | *hsa-miR-451a.53.P0.S.31* | 0 | 2 | 0 | 1 |
|  | *hsa-miR-451a.5.P0.S.1* | 0 | 9 | 15 | 0 |
|  | *hsa-miR-451a.5.P0.S.2* | 0 | 0 | 5 | 0 |
|  | *hsa-miR-451a.5.P0.S.3* | 0 | 0 | 5 | 0 |
|  | *hsa-miR-451a.5.P0.S.4* | 0 | 0 | 40 | 0 |
|  | *hsa-miR-451a.53.P0.S.32* | 0 | 0 | 5 | 0 |
|  | *hsa-miR-451a.0.P1.S.0* | 0 | 0 | 7 | 1 |
|  | *hsa-miR-451a.0.P1.S.1* | 0 | 0 | 3 | 0 |
|  | *hsa-miR-451a.0.P1.S.2* | 0 | 0 | 12 | 0 |
|  | *hsa-miR-451a.0.P1.S.3* | 10 | 0 | 17 | 0 |
|  | *hsa-miR-451a.0.P1.S.4* | 0 | 0 | 0 | 2 |
|  | *hsa-miR-451a.0.P1.S.5* | 19 | 0 | 24 | 4 |
|  | *hsa-miR-451a.0.P1.S.6* | 3 | 0 | 0 | 0 |
|  | *hsa-miR-451a.0.P1.S.7* | 0 | 0 | 17 | 0 |
|  | *hsa-miR-451a.0.P1.S.8* | 0 | 0 | 7 | 3 |
|  | *hsa-miR-451a.0.P1.S.9* | 3 | 5 | 0 | 1 |
|  | *hsa-miR-451a.0.P1.S.10* | 0 | 0 | 3 | 0 |
|  | *hsa-miR-451a.0.P1.S.11* | 0 | 0 | 18 | 0 |
|  | *hsa-miR-451a.0.P1.S.12* | 0 | 0 | 3 | 1 |
|  | *hsa-miR-451a.0.P1.S.13* | 0 | 0 | 8 | 0 |
|  | *hsa-miR-451a.0.P1.S.14* | 0 | 0 | 10 | 0 |
|  | *hsa-miR-451a.0.P1.S.15* | 97 | 2 | 60 | 1 |
|  | *hsa-miR-451a.0.P1.S.16* | 0 | 0 | 5 | 0 |
|  | *hsa-miR-451a.0.P1.S.17* | 0 | 5 | 24 | 2 |
|  | *hsa-miR-451a.0.P1.S.18* | 0 | 0 | 3 | 0 |
|  | *hsa-miR-451a.0.P1.S.19* | 0 | 5 | 12 | 1 |
|  | *hsa-miR-451a.0.P1.S.20* | 0 | 5 | 0 | 0 |
|  | *hsa-miR-451a.0.P1.S.21* | 205 | 70 | 238 | 1 |
|  | *hsa-miR-451a.0.P1.S.22* | 29 | 13 | 50 | 1 |
|  | *hsa-miR-451a.0.P1.S.23* | 111 | 22 | 490 | 4 |
|  | *hsa-miR-451a.0.P1.S.24* | 3 | 0 | 0 | 0 |
|  | *hsa-miR-451a.0.P1.S.25* | 23 | 0 | 3 | 0 |
|  | *hsa-miR-451a.0.P1.S.26* | 19 | 0 | 0 | 0 |
|  | *hsa-miR-451a.0.P1.S.27* | 0 | 0 | 24 | 2 |
|  | *hsa-miR-451a.0.P1.S.28* | 7 | 9 | 10 | 1 |
|  | *hsa-miR-451a.0.P1.S.29* | 0 | 0 | 7 | 0 |
|  | *hsa-miR-451a.0.P1.S.30* | 0 | 2 | 22 | 2 |
|  | *hsa-miR-451a.0.P1.S.31* | 3 | 0 | 3 | 0 |
|  | *hsa-miR-451a.0.P1.S.32* | 0 | 5 | 30 | 1 |
|  | *hsa-miR-451a.0.P1.S.33* | 0 | 0 | 7 | 2 |
|  | *hsa-miR-451a.0.P1.S.34* | 0 | 0 | 5 | 0 |
|  | *hsa-miR-451a.0.P1.S.35* | 0 | 0 | 5 | 0 |
|  | *hsa-miR-451a.0.P1.S.36* | 7 | 5 | 33 | 0 |
|  | *hsa-miR-451a.0.P1.S.37* | 0 | 5 | 19 | 2 |
|  | *hsa-miR-451a.0.P1.S.38* | 3 | 156 | 37 | 1 |
|  | *hsa-miR-451a.0.P1.S.39* | 3 | 5 | 12 | 1 |
| ***hsa-miR-16-5p*** | *hsa-miR-16-5p.3.P0.M.0* | 2332 | 1303 | 916 | 921 |
|  | *hsa-miR-16-5p.3.P0.M.7* | 1651 | 815 | 640 | 616 |
|  | *hsa-miR-16-5p.3.P1.M.15* | 893 | 401 | 326 | 206 |
|  | *hsa-miR-16-5p.3.P0.M.8* | 1341 | 1093 | 704 | 430 |
|  | *hsa-miR-16-5p.3.P1.M.48* | 85 | 56 | 778 | 27 |
|  | *hsa-miR-16-5p.3.P0.M.11* | 5069 | 5094 | 2208 | 3078 |
|  | *hsa-miR-16-5p.3.P0.S.4* | 105 | 189 | 61 | 63 |
|  | *hsa-miR-16-5p.53.P0.M.8* | 134 | 83 | 44 | 50 |
|  | *hsa-miR-16-5p.0.P1.M.30* | 205 | 57 | 67 | 55 |
| ***hsa-miR-320a*** | *hsa-miR-320a.3.P0.S.0* | 0 | 0 | 0 | 2 |
|  | *hsa-miR-320a.3.P0.S.1* | 0 | 0 | 5 | 2 |
|  | *hsa-miR-320a.3.P0.S.2* | 30 | 2 | 13 | 2 |
|  | *hsa-miR-320a.3.P0.S.3* | 3 | 0 | 0 | 0 |
|  | *hsa-miR-320a.3.P0.M.0* | 10 | 0 | 8 | 1 |
|  | *hsa-miR-320a.3.P0.S.4* | 112 | 17 | 72 | 49 |
|  | *hsa-miR-320a.3.P0.M.1* | 7 | 0 | 0 | 0 |
|  | *hsa-miR-320a.3.P0.M.2* | 3 | 4 | 15 | 6 |
|  | *hsa-miR-320a.3.P0.S.5* | 0 | 2 | 0 | 0 |
|  | *hsa-miR-320a.3.P0.S.6* | 3 | 2 | 5 | 0 |
|  | *hsa-miR-320a.3.P0.S.7* | 111 | 18 | 100 | 37 |
|  | *hsa-miR-320a.3.P0.S.8* | 3 | 7 | 5 | 0 |
|  | *hsa-miR-320a.3.P0.S.9* | 392 | 74 | 296 | 407 |
|  | *hsa-miR-320a.3.P0.S.10* | 19 | 0 | 0 | 0 |
|  | *hsa-miR-320a.3.P0.S.11* | 3 | 2 | 5 | 1 |
|  | *hsa-miR-320a.3.P0.S.12* | 29 | 36 | 10 | 8 |
|  | *hsa-miR-320a.3.P0.S.13* | 7 | 0 | 0 | 0 |
|  | *hsa-miR-320a.3.P0.S.14* | 40 | 2 | 17 | 4 |
|  | *hsa-miR-320a.3.P0.S.15* | 40 | 11 | 51 | 10 |
|  | *hsa-miR-320a.3.P0.S.16* | 0 | 0 | 0 | 2 |
|  | *hsa-miR-320a.3.P0.S.17* | 7 | 4 | 15 | 10 |
|  | *hsa-miR-320a.3.P0.S.18* | 99 | 24 | 38 | 20 |
|  | *hsa-miR-320a.3.P0.S.19* | 612 | 119 | 253 | 152 |
|  | *hsa-miR-320a.3.P0.S.20* | 0 | 0 | 7 | 0 |
|  | *hsa-miR-320a.3.P0.S.21* | 7 | 2 | 0 | 6 |
|  | *hsa-miR-320a.3.P0.S.22* | 111 | 40 | 65 | 29 |
|  | *hsa-miR-320a.3.P1.S.0* | 3 | 0 | 0 | 0 |
|  | *hsa-miR-320a.3.P1.S.1* | 3 | 0 | 0 | 0 |
|  | *hsa-miR-320a.3.P1.S.2* | 0 | 0 | 0 | 1 |
|  | *hsa-miR-320a.3.P1.S.3* | 0 | 0 | 3 | 0 |
|  | *hsa-miR-320a.3.P1.S.4* | 0 | 4 | 0 | 0 |
|  | *hsa-miR-320a.3.P0.S.23* | 2033 | 596 | 2232 | 1246 |
|  | *hsa-miR-320a.3.P1.S.5* | 3 | 0 | 0 | 0 |
|  | *hsa-miR-320a.3.P1.S.6* | 3 | 2 | 0 | 2 |
|  | *hsa-miR-320a.3.P1.S.7* | 0 | 0 | 5 | 0 |
|  | *hsa-miR-320a.3.P1.S.8* | 0 | 0 | 0 | 2 |
|  | *hsa-miR-320a.3.P1.S.9* | 0 | 0 | 3 | 0 |
|  | *hsa-miR-320a.3.P1.S.10* | 3 | 0 | 7 | 3 |
|  | *hsa-miR-320a.3.P1.S.11* | 0 | 0 | 3 | 0 |
|  | *hsa-miR-320a.3.P1.S.12* | 19 | 0 | 0 | 0 |
|  | *hsa-miR-320a.3.P1.S.13* | 0 | 0 | 5 | 0 |
|  | *hsa-miR-320a.3.P1.S.14* | 0 | 0 | 5 | 0 |
|  | *hsa-miR-320a.3.P1.S.15* | 3 | 0 | 0 | 0 |
|  | *hsa-miR-320a.3.P1.S.16* | 3 | 0 | 8 | 1 |
|  | *hsa-miR-320a.3.P1.S.17* | 0 | 0 | 0 | 1 |
|  | *hsa-miR-320a.3.P1.S.18* | 3 | 0 | 0 | 3 |
|  | *hsa-miR-320a.3.P1.S.19* | 3 | 0 | 0 | 0 |
|  | *hsa-miR-320a.3.P1.S.20* | 0 | 0 | 3 | 0 |
|  | *hsa-miR-320a.3.P1.S.21* | 0 | 0 | 3 | 0 |
|  | *hsa-miR-320a.3.P1.S.22* | 0 | 0 | 0 | 1 |
|  | *hsa-miR-320a.3.P1.M.0* | 0 | 0 | 0 | 1 |
|  | *hsa-miR-320a.3.P1.S.23* | 0 | 2 | 0 | 0 |
|  | *hsa-miR-320a.3.P1.S.24* | 68 | 25 | 75 | 43 |
|  | *hsa-miR-320a.3.P0.S.24* | 832 | 198 | 837 | 184 |
|  | *hsa-miR-320a.3.P0.S.25* | 6488 | 1523 | 5040 | 2174 |
|  | *hsa-miR-320a.3.P1.S.25* | 0 | 2 | 0 | 0 |
|  | *hsa-miR-320a.3.P1.S.26* | 3 | 2 | 5 | 1 |
|  | *hsa-miR-320a.3.P1.S.27* | 7 | 0 | 0 | 0 |
|  | *hsa-miR-320a.3.P1.S.28* | 3 | 2 | 5 | 0 |
|  | *hsa-miR-320a.3.P1.S.29* | 3 | 0 | 3 | 0 |
|  | *hsa-miR-320a.3.P1.S.30* | 0 | 0 | 8 | 0 |
|  | *hsa-miR-320a.3.P1.S.31* | 3 | 0 | 15 | 0 |
|  | *hsa-miR-320a.3.P1.S.32* | 0 | 0 | 7 | 0 |
|  | *hsa-miR-320a.3.P1.S.33* | 10 | 2 | 25 | 1 |
|  | *hsa-miR-320a.3.P1.S.34* | 3 | 0 | 17 | 0 |
|  | *hsa-miR-320a.3.P1.S.35* | 3 | 0 | 0 | 0 |
|  | *hsa-miR-320a.3.P1.S.36* | 0 | 2 | 0 | 3 |
|  | *hsa-miR-320a.3.P1.S.37* | 3 | 0 | 5 | 0 |
|  | *hsa-miR-320a.3.P1.S.38* | 19 | 0 | 3 | 1 |
|  | *hsa-miR-320a.3.P1.S.39* | 0 | 0 | 0 | 1 |
|  | *hsa-miR-320a.3.P1.S.40* | 3 | 2 | 3 | 1 |
|  | *hsa-miR-320a.3.P1.S.41* | 0 | 2 | 0 | 0 |
|  | *hsa-miR-320a.3.P1.S.42* | 0 | 0 | 3 | 1 |
|  | *hsa-miR-320a.3.P1.S.43* | 0 | 0 | 5 | 0 |
|  | *hsa-miR-320a.3.P1.S.44* | 0 | 0 | 0 | 1 |
|  | *hsa-miR-320a.3.P1.S.45* | 7 | 0 | 3 | 0 |
|  | *hsa-miR-320a.3.P1.S.46* | 3 | 0 | 0 | 0 |
|  | *hsa-miR-320a.3.P1.S.47* | 0 | 0 | 0 | 1 |
|  | *hsa-miR-320a.3.P1.S.48* | 0 | 0 | 3 | 0 |
|  | *hsa-miR-320a.3.P1.S.49* | 0 | 0 | 0 | 1 |
|  | *hsa-miR-320a.3.P1.S.50* | 0 | 0 | 7 | 0 |
|  | *hsa-miR-320a.3.P1.S.51* | 7 | 0 | 3 | 2 |
|  | *hsa-miR-320a.3.P1.S.52* | 19 | 0 | 0 | 0 |
|  | *hsa-miR-320a.3.P1.S.53* | 3 | 0 | 0 | 0 |
|  | *hsa-miR-320a.3.P1.S.54* | 0 | 0 | 0 | 1 |
|  | *hsa-miR-320a.3.P1.S.55* | 26 | 2 | 18 | 1 |
|  | *hsa-miR-320a.3.P1.S.56* | 0 | 0 | 0 | 1 |
|  | *hsa-miR-320a.3.P1.S.57* | 10 | 5 | 0 | 1 |
|  | *hsa-miR-320a.3.P1.S.58* | 0 | 0 | 0 | 1 |
|  | *hsa-miR-320a.3.P1.S.59* | 0 | 0 | 0 | 1 |
|  | *hsa-miR-320a.3.P1.S.60* | 3 | 0 | 0 | 0 |
|  | *hsa-miR-320a.3.P1.S.61* | 0 | 0 | 0 | 1 |
|  | *hsa-miR-320a.3.P1.S.62* | 0 | 2 | 0 | 0 |
|  | *hsa-miR-320a.3.P1.S.63* | 0 | 0 | 3 | 1 |
|  | *hsa-miR-320a.3.P1.S.64* | 0 | 0 | 7 | 0 |
|  | *hsa-miR-320a.3.P1.S.65* | 3 | 0 | 0 | 2 |
|  | *hsa-miR-320a.3.P1.M.1* | 3 | 0 | 0 | 0 |
|  | *hsa-miR-320a.3.P1.M.2* | 26 | 7 | 5 | 0 |
|  | *hsa-miR-320a.3.P1.M.3* | 13 | 2 | 54 | 6 |
|  | *hsa-miR-320a.3.P1.M.4* | 0 | 2 | 0 | 0 |
|  | *hsa-miR-320a.3.P1.S.66* | 7 | 0 | 0 | 0 |
|  | *hsa-miR-320a.3.P1.S.67* | 0 | 0 | 3 | 2 |
|  | *hsa-miR-320a.3.P1.S.68* | 30 | 4 | 15 | 2 |
|  | *hsa-miR-320a.3.P0.M.3* | 449 | 197 | 663 | 207 |
|  | *hsa-miR-320a.3.P1.M.5* | 19 | 0 | 0 | 0 |
|  | *hsa-miR-320a.3.P1.M.6* | 0 | 0 | 3 | 0 |
|  | *hsa-miR-320a.3.P1.M.7* | 0 | 0 | 3 | 0 |
|  | *hsa-miR-320a.3.P1.M.8* | 0 | 0 | 3 | 0 |
|  | *hsa-miR-320a.3.P1.M.9* | 0 | 0 | 0 | 1 |
|  | *hsa-miR-320a.3.P1.M.10* | 0 | 0 | 7 | 0 |
|  | *hsa-miR-320a.3.P1.M.11* | 0 | 0 | 3 | 0 |
|  | *hsa-miR-320a.3.P1.M.12* | 0 | 0 | 3 | 1 |
|  | *hsa-miR-320a.3.P1.M.13* | 0 | 0 | 3 | 1 |
|  | *hsa-miR-320a.3.P1.M.14* | 0 | 0 | 0 | 1 |
|  | *hsa-miR-320a.3.P1.M.15* | 0 | 0 | 0 | 1 |
|  | *hsa-miR-320a.3.P1.M.16* | 0 | 0 | 5 | 1 |
|  | *hsa-miR-320a.3.P0.M.4* | 72 | 25 | 74 | 11 |
|  | *hsa-miR-320a.3.P1.M.17* | 40 | 9 | 37 | 18 |
|  | *hsa-miR-320a.3.P1.M.18* | 0 | 0 | 0 | 1 |
|  | *hsa-miR-320a.3.P0.M.5* | 150 | 83 | 137 | 74 |
|  | *hsa-miR-320a.3.P1.M.19* | 3 | 0 | 0 | 1 |
|  | *hsa-miR-320a.3.P1.M.20* | 0 | 0 | 0 | 1 |
|  | *hsa-miR-320a.3.P1.M.21* | 0 | 0 | 0 | 1 |
|  | *hsa-miR-320a.3.P1.M.22* | 0 | 0 | 3 | 0 |
|  | *hsa-miR-320a.3.P1.M.23* | 0 | 0 | 0 | 1 |
|  | *hsa-miR-320a.3.P1.M.24* | 3 | 5 | 0 | 0 |
|  | *hsa-miR-320a.3.P1.M.25* | 3 | 2 | 14 | 1 |
|  | *hsa-miR-320a.3.P1.M.26* | 10 | 2 | 0 | 1 |
|  | *hsa-miR-320a.3.P1.M.27* | 55 | 7 | 52 | 10 |
|  | *hsa-miR-320a.3.P1.M.28* | 0 | 5 | 7 | 1 |
|  | *hsa-miR-320a.53.P0.S.0* | 0 | 0 | 5 | 0 |
|  | *hsa-miR-320a.5.P0.S.0* | 0 | 2 | 0 | 0 |
|  | *hsa-miR-320a.53.P0.S.1* | 0 | 0 | 3 | 0 |
|  | *hsa-miR-320a.5.P0.S.1* | 0 | 14 | 0 | 0 |
|  | *hsa-miR-320a.53.P0.S.2* | 0 | 0 | 3 | 0 |
|  | *hsa-miR-320a.53.P0.S.3* | 3 | 0 | 3 | 0 |
|  | *hsa-miR-320a.5.P0.S.2* | 10 | 0 | 0 | 0 |
|  | *hsa-miR-320a.53.P0.S.4* | 0 | 0 | 0 | 1 |
|  | *hsa-miR-320a.53.P0.S.5* | 0 | 5 | 3 | 1 |
|  | *hsa-miR-320a.53.P0.S.6* | 0 | 0 | 3 | 0 |
|  | *hsa-miR-320a.53.P0.S.7* | 0 | 9 | 0 | 0 |
|  | *hsa-miR-320a.53.P0.S.8* | 33 | 11 | 30 | 27 |
|  | *hsa-miR-320a.53.P0.M.0* | 0 | 0 | 5 | 3 |
|  | *hsa-miR-320a.53.P0.S.9* | 0 | 2 | 18 | 0 |
|  | *hsa-miR-320a.53.P0.S.10* | 29 | 13 | 32 | 5 |
|  | *hsa-miR-320a.53.P0.S.11* | 0 | 0 | 3 | 0 |
|  | *hsa-miR-320a.53.P0.S.12* | 0 | 0 | 3 | 0 |
|  | *hsa-miR-320a.53.P0.S.13* | 158 | 80 | 190 | 105 |
|  | *hsa-miR-320a.53.P0.S.14* | 3 | 0 | 5 | 1 |
|  | *hsa-miR-320a.53.P0.S.15* | 29 | 0 | 3 | 0 |
|  | *hsa-miR-320a.53.P0.S.16* | 0 | 9 | 8 | 3 |
|  | *hsa-miR-320a.53.P0.S.17* | 7 | 0 | 0 | 5 |
|  | *hsa-miR-320a.53.P0.S.18* | 7 | 9 | 25 | 0 |
|  | *hsa-miR-320a.53.P0.S.19* | 168 | 26 | 113 | 23 |
|  | *hsa-miR-320a.53.P0.S.20* | 3 | 0 | 0 | 0 |
|  | *hsa-miR-320a.53.P0.S.21* | 3 | 18 | 35 | 11 |
|  | *hsa-miR-320a.53.P0.S.22* | 421 | 138 | 523 | 146 |
|  | *hsa-miR-320a.53.P1.S.0* | 19 | 0 | 3 | 0 |
|  | *hsa-miR-320a.53.P1.S.1* | 0 | 0 | 5 | 0 |
|  | *hsa-miR-320a.53.P1.S.2* | 3 | 0 | 0 | 0 |
|  | *hsa-miR-320a.53.P1.S.3* | 0 | 0 | 3 | 0 |
|  | *hsa-miR-320a.53.P1.S.4* | 39 | 0 | 3 | 0 |
|  | *hsa-miR-320a.53.P1.S.5* | 0 | 5 | 0 | 0 |
|  | *hsa-miR-320a.53.P1.S.6* | 0 | 0 | 0 | 2 |
|  | *hsa-miR-320a.53.P1.S.7* | 62 | 9 | 0 | 4 |
|  | *hsa-miR-320a.53.P0.S.23* | 197 | 66 | 210 | 72 |
|  | *hsa-miR-320a.53.P0.S.24* | 909 | 230 | 1166 | 292 |
|  | *hsa-miR-320a.53.P1.S.8* | 0 | 0 | 0 | 1 |
|  | *hsa-miR-320a.53.P1.S.9* | 0 | 0 | 0 | 2 |
|  | *hsa-miR-320a.53.P1.S.10* | 0 | 0 | 0 | 2 |
|  | *hsa-miR-320a.53.P1.S.11* | 0 | 2 | 0 | 1 |
|  | *hsa-miR-320a.53.P1.S.12* | 0 | 0 | 3 | 0 |
|  | *hsa-miR-320a.53.P1.S.13* | 3 | 0 | 0 | 0 |
|  | *hsa-miR-320a.53.P1.S.14* | 0 | 0 | 0 | 1 |
|  | *hsa-miR-320a.53.P1.S.15* | 3 | 0 | 0 | 0 |
|  | *hsa-miR-320a.53.P1.S.16* | 0 | 0 | 0 | 1 |
|  | *hsa-miR-320a.53.P1.S.17* | 0 | 0 | 7 | 0 |
|  | *hsa-miR-320a.53.P1.M.0* | 0 | 0 | 0 | 2 |
|  | *hsa-miR-320a.53.P1.M.1* | 0 | 0 | 0 | 2 |
|  | *hsa-miR-320a.53.P0.M.1* | 149 | 27 | 80 | 19 |
|  | *hsa-miR-320a.53.P1.M.2* | 0 | 0 | 0 | 2 |
|  | *hsa-miR-320a.53.P1.M.3* | 0 | 5 | 0 | 0 |
|  | *hsa-miR-320a.53.P1.M.4* | 3 | 4 | 40 | 0 |
|  | *hsa-miR-320a.53.P1.M.5* | 3 | 0 | 0 | 0 |
|  | *hsa-miR-320a.53.P0.M.2* | 7 | 5 | 17 | 4 |
|  | *hsa-miR-320a.53.P1.M.6* | 0 | 0 | 0 | 1 |
|  | *hsa-miR-320a.53.P1.M.7* | 20 | 7 | 3 | 1 |
|  | *hsa-miR-320a.53.P1.M.8* | 0 | 9 | 25 | 2 |
|  | *hsa-miR-320a.53.P0.S.25* | 0 | 5 | 0 | 0 |
|  | *hsa-miR-320a.53.P0.S.26* | 0 | 0 | 3 | 0 |
|  | *hsa-miR-320a.53.P0.S.27* | 0 | 0 | 15 | 0 |
|  | *hsa-miR-320a.53.P0.M.3* | 19 | 0 | 10 | 1 |
|  | *hsa-miR-320a.53.P0.S.28* | 0 | 7 | 0 | 2 |
|  | *hsa-miR-320a.53.P0.S.29* | 17 | 24 | 47 | 24 |
|  | *hsa-miR-320a.53.P0.M.4* | 3 | 0 | 3 | 0 |
|  | *hsa-miR-320a.53.P0.S.30* | 3 | 2 | 20 | 1 |
|  | *hsa-miR-320a.53.P0.S.31* | 0 | 0 | 0 | 2 |
|  | *hsa-miR-320a.53.P0.S.32* | 0 | 0 | 0 | 1 |
|  | *hsa-miR-320a.53.P0.S.33* | 131 | 22 | 85 | 35 |
|  | *hsa-miR-320a.53.P0.S.34* | 0 | 2 | 10 | 0 |
|  | *hsa-miR-320a.53.P0.S.35* | 0 | 2 | 3 | 3 |
|  | *hsa-miR-320a.53.P0.S.36* | 0 | 0 | 0 | 1 |
|  | *hsa-miR-320a.53.P0.S.37* | 7 | 0 | 0 | 0 |
|  | *hsa-miR-320a.53.P0.S.38* | 42 | 9 | 17 | 2 |
|  | *hsa-miR-320a.53.P0.S.39* | 0 | 2 | 0 | 0 |
|  | *hsa-miR-320a.53.P0.S.40* | 19 | 5 | 13 | 0 |
|  | *hsa-miR-320a.53.P0.S.41* | 13 | 9 | 37 | 18 |
|  | *hsa-miR-320a.53.P1.S.18* | 0 | 5 | 0 | 0 |
|  | *hsa-miR-320a.53.P1.S.19* | 0 | 2 | 0 | 0 |
|  | *hsa-miR-320a.53.P0.S.42* | 454 | 175 | 643 | 158 |
|  | *hsa-miR-320a.53.P1.S.20* | 0 | 0 | 0 | 2 |
|  | *hsa-miR-320a.53.P1.S.21* | 0 | 0 | 5 | 0 |
|  | *hsa-miR-320a.53.P1.S.22* | 0 | 0 | 0 | 1 |
|  | *hsa-miR-320a.53.P1.S.23* | 3 | 0 | 0 | 0 |
|  | *hsa-miR-320a.53.P1.S.24* | 0 | 2 | 0 | 0 |
|  | *hsa-miR-320a.53.P1.S.25* | 0 | 0 | 0 | 1 |
|  | *hsa-miR-320a.53.P1.S.26* | 0 | 0 | 3 | 0 |
|  | *hsa-miR-320a.53.P1.S.27* | 0 | 2 | 0 | 0 |
|  | *hsa-miR-320a.53.P0.S.43* | 68 | 27 | 82 | 6 |
|  | *hsa-miR-320a.53.P0.S.44* | 407 | 134 | 583 | 154 |
|  | *hsa-miR-320a.53.P1.S.28* | 3 | 0 | 0 | 0 |
|  | *hsa-miR-320a.53.P1.S.29* | 0 | 0 | 3 | 0 |
|  | *hsa-miR-320a.53.P1.S.30* | 0 | 0 | 3 | 1 |
|  | *hsa-miR-320a.53.P1.S.31* | 0 | 0 | 0 | 1 |
|  | *hsa-miR-320a.53.P1.S.32* | 3 | 0 | 0 | 0 |
|  | *hsa-miR-320a.53.P1.S.33* | 0 | 0 | 0 | 1 |
|  | *hsa-miR-320a.53.P1.S.34* | 0 | 0 | 3 | 0 |
|  | *hsa-miR-320a.53.P1.S.35* | 7 | 0 | 0 | 0 |
|  | *hsa-miR-320a.53.P1.M.9* | 0 | 2 | 0 | 0 |
|  | *hsa-miR-320a.53.P1.S.36* | 3 | 0 | 0 | 0 |
|  | *hsa-miR-320a.53.P0.M.5* | 0 | 7 | 20 | 1 |
|  | *hsa-miR-320a.53.P1.M.10* | 0 | 0 | 5 | 0 |
|  | *hsa-miR-320a.53.P1.M.11* | 3 | 2 | 0 | 0 |
|  | *hsa-miR-320a.53.P1.M.12* | 0 | 0 | 0 | 1 |
|  | *hsa-miR-320a.53.P0.S.45* | 0 | 0 | 3 | 0 |
|  | *hsa-miR-320a.53.P0.S.46* | 19 | 0 | 0 | 0 |
|  | *hsa-miR-320a.53.P0.S.47* | 0 | 0 | 5 | 0 |
|  | *hsa-miR-320a.53.P0.S.48* | 0 | 0 | 0 | 1 |
|  | *hsa-miR-320a.53.P0.S.49* | 7 | 0 | 0 | 0 |
|  | *hsa-miR-320a.53.P0.S.50* | 0 | 0 | 3 | 0 |
|  | *hsa-miR-320a.53.P0.S.51* | 0 | 0 | 0 | 1 |
|  | *hsa-miR-320a.53.P1.S.37* | 19 | 0 | 0 | 0 |
|  | *hsa-miR-320a.53.P0.S.52* | 19 | 0 | 0 | 0 |
|  | *hsa-miR-320a.5.P0.S.3* | 0 | 2 | 13 | 4 |
|  | *hsa-miR-320a.5.P1.S.0* | 0 | 0 | 3 | 0 |
|  | *hsa-miR-320a.5.P1.S.1* | 19 | 0 | 0 | 0 |
|  | *hsa-miR-320a.5.P0.S.4* | 0 | 0 | 3 | 4 |
|  | *hsa-miR-320a.5.P1.S.2* | 3 | 0 | 0 | 0 |
|  | *hsa-miR-320a.5.P0.S.5* | 542 | 203 | 574 | 106 |
|  | *hsa-miR-320a.5.P1.S.3* | 0 | 0 | 0 | 1 |
|  | *hsa-miR-320a.5.P1.S.4* | 0 | 0 | 0 | 1 |
|  | *hsa-miR-320a.5.P1.S.5* | 3 | 0 | 0 | 0 |
|  | *hsa-miR-320a.5.P1.S.6* | 0 | 0 | 0 | 1 |
|  | *hsa-miR-320a.5.P1.S.7* | 0 | 0 | 3 | 0 |
|  | *hsa-miR-320a.5.P1.S.8* | 0 | 0 | 5 | 1 |
|  | *hsa-miR-320a.5.P1.S.9* | 0 | 0 | 5 | 0 |
|  | *hsa-miR-320a.5.P1.S.10* | 0 | 0 | 0 | 1 |
|  | *hsa-miR-320a.5.P1.S.11* | 0 | 5 | 0 | 2 |
|  | *hsa-miR-320a.5.P1.S.12* | 0 | 0 | 3 | 0 |
|  | *hsa-miR-320a.5.P1.S.13* | 17 | 9 | 15 | 1 |
|  | *hsa-miR-320a.5.P0.S.6* | 2346 | 571 | 2382 | 514 |
|  | *hsa-miR-320a.5.P1.S.14* | 0 | 2 | 0 | 0 |
|  | *hsa-miR-320a.5.P1.S.15* | 0 | 0 | 3 | 2 |
|  | *hsa-miR-320a.5.P1.S.16* | 3 | 0 | 8 | 0 |
|  | *hsa-miR-320a.5.P1.S.17* | 3 | 0 | 0 | 0 |
|  | *hsa-miR-320a.5.P1.S.18* | 0 | 0 | 0 | 2 |
|  | *hsa-miR-320a.5.P1.S.19* | 0 | 0 | 0 | 1 |
|  | *hsa-miR-320a.5.P1.S.20* | 7 | 0 | 0 | 0 |
|  | *hsa-miR-320a.5.P1.S.21* | 0 | 0 | 0 | 1 |
|  | *hsa-miR-320a.5.P1.S.22* | 0 | 0 | 5 | 0 |
|  | *hsa-miR-320a.5.P1.S.23* | 0 | 0 | 3 | 0 |
|  | *hsa-miR-320a.5.P1.S.24* | 0 | 0 | 0 | 1 |
|  | *hsa-miR-320a.5.P1.S.25* | 0 | 0 | 0 | 4 |
|  | *hsa-miR-320a.5.P1.S.26* | 0 | 0 | 0 | 1 |
|  | *hsa-miR-320a.5.P1.S.27* | 3 | 0 | 0 | 0 |
|  | *hsa-miR-320a.5.P1.S.28* | 0 | 0 | 0 | 1 |
|  | *hsa-miR-320a.5.P1.S.29* | 0 | 0 | 3 | 0 |
|  | *hsa-miR-320a.5.P1.S.30* | 0 | 0 | 0 | 2 |
|  | *hsa-miR-320a.5.P1.S.31* | 0 | 0 | 3 | 0 |
|  | *hsa-miR-320a.5.P1.S.32* | 0 | 9 | 8 | 1 |
|  | *hsa-miR-320a.5.P1.S.33* | 7 | 0 | 5 | 0 |
|  | *hsa-miR-320a.5.P1.S.34* | 128 | 16 | 81 | 29 |
|  | *hsa-miR-320a.53.P0.S.53* | 0 | 0 | 5 | 0 |
|  | *hsa-miR-320a.53.P0.S.54* | 19 | 0 | 0 | 0 |
|  | *hsa-miR-320a.53.P0.S.55* | 10 | 0 | 0 | 1 |
|  | *hsa-miR-320a.53.P0.S.56* | 3 | 0 | 0 | 0 |
|  | *hsa-miR-320a.53.P0.S.57* | 19 | 2 | 0 | 2 |
|  | *hsa-miR-320a.53.P0.S.58* | 36 | 14 | 32 | 23 |
|  | *hsa-miR-320a.53.P0.S.59* | 10 | 0 | 8 | 5 |
|  | *hsa-miR-320a.53.P1.S.38* | 0 | 0 | 3 | 0 |
|  | *hsa-miR-320a.53.P1.M.13* | 0 | 0 | 14 | 0 |
|  | *hsa-miR-320a.53.P1.S.39* | 0 | 0 | 5 | 0 |
|  | *hsa-miR-320a.53.P0.M.6* | 7 | 2 | 7 | 15 |
|  | *hsa-miR-320a.53.P1.M.14* | 0 | 0 | 0 | 1 |
|  | *hsa-miR-320a.53.P0.M.7* | 0 | 7 | 3 | 0 |
|  | *hsa-miR-320a.53.P1.M.15* | 0 | 0 | 0 | 1 |
|  | *hsa-miR-320a.53.P0.M.8* | 0 | 0 | 14 | 1 |
|  | *hsa-miR-320a.53.P0.M.9* | 0 | 0 | 0 | 1 |
|  | *hsa-miR-320a.5.P0.S.7* | 262 | 42 | 178 | 117 |
|  | *hsa-miR-320a.5.P1.S.35* | 0 | 0 | 0 | 1 |
|  | *hsa-miR-320a.5.P1.S.36* | 0 | 0 | 0 | 1 |
|  | *hsa-miR-320a.5.P1.S.37* | 0 | 5 | 0 | 0 |
|  | *hsa-miR-320a.5.P1.S.38* | 0 | 0 | 3 | 0 |
|  | *hsa-miR-320a.5.P1.S.39* | 0 | 0 | 0 | 1 |
|  | *hsa-miR-320a.5.P1.S.40* | 3 | 0 | 0 | 0 |
|  | *hsa-miR-320a.5.P1.S.41* | 36 | 4 | 41 | 17 |
|  | *hsa-miR-320a.53.P1.S.40* | 0 | 2 | 0 | 0 |
|  | *hsa-miR-320a.0.P1.S.0* | 3 | 4 | 5 | 0 |
|  | *hsa-miR-320a.0.P1.S.1* | 26 | 0 | 10 | 0 |
|  | *hsa-miR-320a.0.P1.S.2* | 3 | 2 | 10 | 2 |
|  | *hsa-miR-320a.0.P1.S.3* | 3 | 0 | 3 | 0 |
|  | *hsa-miR-320a.0.P1.S.4* | 3 | 0 | 3 | 2 |
|  | *hsa-miR-320a.0.P1.S.5* | 10 | 2 | 17 | 0 |
|  | *hsa-miR-320a.0.P1.S.6* | 0 | 0 | 5 | 0 |
|  | *hsa-miR-320a.0.P1.S.7* | 7 | 0 | 41 | 0 |
|  | *hsa-miR-320a.0.P1.S.8* | 55 | 0 | 53 | 4 |
|  | *hsa-miR-320a.0.P1.S.9* | 3 | 0 | 34 | 0 |
|  | *hsa-miR-320a.0.P1.S.10* | 0 | 0 | 3 | 0 |
|  | *hsa-miR-320a.0.P1.S.11* | 3 | 2 | 3 | 0 |
|  | *hsa-miR-320a.0.P1.S.12* | 3 | 0 | 0 | 0 |
|  | *hsa-miR-320a.0.P1.S.13* | 55 | 4 | 32 | 1 |
|  | *hsa-miR-320a.0.P1.S.14* | 0 | 5 | 0 | 0 |
|  | *hsa-miR-320a.0.P1.S.15* | 3 | 0 | 3 | 2 |
|  | *hsa-miR-320a.0.P1.S.16* | 0 | 2 | 3 | 1 |
|  | *hsa-miR-320a.0.P1.S.17* | 3 | 0 | 3 | 0 |
|  | *hsa-miR-320a.0.P1.S.18* | 26 | 0 | 3 | 0 |
|  | *hsa-miR-320a.0.P1.S.19* | 10 | 0 | 0 | 0 |
|  | *hsa-miR-320a.0.P1.S.20* | 19 | 5 | 0 | 6 |
|  | *hsa-miR-320a.0.P1.S.21* | 0 | 0 | 0 | 1 |
|  | *hsa-miR-320a.0.P1.S.22* | 7 | 5 | 8 | 9 |
|  | *hsa-miR-320a.0.P1.S.23* | 0 | 0 | 0 | 1 |
|  | *hsa-miR-320a.0.P1.S.24* | 0 | 0 | 3 | 0 |
|  | *hsa-miR-320a.0.P1.S.25* | 0 | 0 | 3 | 1 |
|  | *hsa-miR-320a.0.P1.S.26* | 13 | 7 | 5 | 4 |
|  | *hsa-miR-320a.0.P1.S.27* | 0 | 0 | 3 | 1 |
|  | *hsa-miR-320a.0.P1.S.28* | 13 | 0 | 5 | 1 |
|  | *hsa-miR-320a.0.P1.S.29* | 3 | 5 | 0 | 0 |
|  | *hsa-miR-320a.0.P1.S.30* | 0 | 0 | 8 | 3 |
|  | *hsa-miR-320a.0.P1.S.31* | 26 | 0 | 0 | 2 |
|  | *hsa-miR-320a.0.P1.S.32* | 3 | 0 | 0 | 1 |
|  | *hsa-miR-320a.0.P1.S.33* | 0 | 0 | 21 | 1 |
|  | *hsa-miR-320a.0.P1.S.34* | 3 | 0 | 13 | 4 |
|  | *hsa-miR-320a.0.P1.S.35* | 17 | 18 | 47 | 8 |
|  | *hsa-miR-320a.0.P1.S.36* | 43 | 9 | 75 | 4 |
|  | *hsa-miR-320a.0.P1.S.37* | 1045 | 312 | 1122 | 225 |
| ***hsa-miR-373-3p*** | *hsa-miR-373-3p.3.P0.S.0* | 0 | 0 | 0 | 9 |
|  | *hsa-miR-373-3p.3.P0.S.1* | 0 | 0 | 0 | 38 |
|  | *hsa-miR-373-3p.3.P0.S.2* | 0 | 0 | 0 | 104 |
|  | *hsa-miR-373-3p.3.P0.S.3* | 0 | 0 | 0 | 10 |
|  | *hsa-miR-373-3p.3.P0.S.4* | 0 | 0 | 0 | 302 |
|  | *hsa-miR-373-3p.3.P0.S.5* | 0 | 0 | 0 | 13 |
|  | *hsa-miR-373-3p.3.P0.S.6* | 0 | 0 | 0 | 42 |
|  | *hsa-miR-373-3p.3.P0.S.7* | 0 | 0 | 0 | 37 |
|  | *hsa-miR-373-3p.3.P0.S.8* | 0 | 0 | 17 | 1592 |
|  | *hsa-miR-373-3p.3.P0.S.9* | 0 | 0 | 0 | 4 |
|  | *hsa-miR-373-3p.3.P1.S.0* | 0 | 0 | 0 | 1 |
|  | *hsa-miR-373-3p.3.P0.S.10* | 0 | 0 | 0 | 19 |
|  | *hsa-miR-373-3p.3.P1.S.1* | 0 | 0 | 0 | 1 |
|  | *hsa-miR-373-3p.3.P1.S.2* | 0 | 0 | 0 | 1 |
|  | *hsa-miR-373-3p.3.P0.S.11* | 0 | 0 | 3 | 171 |
|  | *hsa-miR-373-3p.3.P1.S.3* | 0 | 0 | 0 | 1 |
|  | *hsa-miR-373-3p.3.P1.S.4* | 0 | 0 | 0 | 1 |
|  | *hsa-miR-373-3p.3.P1.S.5* | 0 | 0 | 0 | 1 |
|  | *hsa-miR-373-3p.3.P1.S.6* | 0 | 0 | 0 | 1 |
|  | *hsa-miR-373-3p.3.P1.S.7* | 0 | 0 | 0 | 1 |
|  | *hsa-miR-373-3p.3.P1.S.8* | 0 | 0 | 0 | 7 |
|  | *hsa-miR-373-3p.3.P1.S.9* | 0 | 0 | 0 | 2 |
|  | *hsa-miR-373-3p.3.P1.S.10* | 0 | 0 | 0 | 1 |
|  | *hsa-miR-373-3p.3.P0.S.12* | 0 | 2 | 0 | 743 |
|  | *hsa-miR-373-3p.3.P1.S.11* | 0 | 0 | 0 | 2 |
|  | *hsa-miR-373-3p.3.P1.S.12* | 0 | 0 | 0 | 1 |
|  | *hsa-miR-373-3p.3.P1.S.13* | 0 | 0 | 0 | 2 |
|  | *hsa-miR-373-3p.3.P1.S.14* | 0 | 0 | 0 | 11 |
|  | *hsa-miR-373-3p.3.P1.S.15* | 0 | 0 | 0 | 3 |
|  | *hsa-miR-373-3p.3.P1.S.16* | 0 | 0 | 0 | 4 |
|  | *hsa-miR-373-3p.3.P1.S.17* | 0 | 2 | 0 | 2 |
|  | *hsa-miR-373-3p.3.P1.S.18* | 0 | 0 | 0 | 3 |
|  | *hsa-miR-373-3p.3.P1.S.19* | 0 | 0 | 0 | 11 |
|  | *hsa-miR-373-3p.3.P1.S.20* | 0 | 0 | 0 | 7 |
|  | *hsa-miR-373-3p.3.P1.S.21* | 0 | 0 | 0 | 6 |
|  | *hsa-miR-373-3p.3.P1.S.22* | 0 | 0 | 0 | 2 |
|  | *hsa-miR-373-3p.3.P1.S.23* | 0 | 0 | 0 | 3 |
|  | *hsa-miR-373-3p.3.P1.S.24* | 0 | 0 | 0 | 18 |
|  | *hsa-miR-373-3p.3.P1.S.25* | 0 | 0 | 3 | 26 |
|  | *hsa-miR-373-3p.3.P1.S.26* | 0 | 0 | 0 | 4 |
|  | *hsa-miR-373-3p.3.P1.S.27* | 0 | 5 | 0 | 29 |
|  | *hsa-miR-373-3p.3.P1.S.28* | 3 | 0 | 0 | 68 |
|  | *hsa-miR-373-3p.3.P1.S.29* | 19 | 0 | 0 | 15 |
|  | *hsa-miR-373-3p.3.P0.S.13* | 19 | 0 | 13 | 1683 |
|  | *hsa-miR-373-3p.3.P1.S.30* | 0 | 0 | 0 | 3 |
|  | *hsa-miR-373-3p.3.P1.S.31* | 0 | 0 | 0 | 1 |
|  | *hsa-miR-373-3p.3.P1.S.32* | 0 | 0 | 0 | 1 |
|  | *hsa-miR-373-3p.3.P1.S.33* | 0 | 0 | 0 | 1 |
|  | *hsa-miR-373-3p.3.P1.S.34* | 0 | 0 | 0 | 2 |
|  | *hsa-miR-373-3p.3.P1.S.35* | 0 | 0 | 3 | 4 |
|  | *hsa-miR-373-3p.3.P1.S.36* | 0 | 0 | 0 | 6 |
|  | *hsa-miR-373-3p.3.P1.S.37* | 0 | 0 | 0 | 4 |
|  | *hsa-miR-373-3p.3.P1.S.38* | 0 | 0 | 0 | 6 |
|  | *hsa-miR-373-3p.3.P1.S.39* | 0 | 0 | 0 | 2 |
|  | *hsa-miR-373-3p.3.P1.S.40* | 0 | 0 | 0 | 3 |
|  | *hsa-miR-373-3p.3.P1.S.41* | 0 | 0 | 0 | 2 |
|  | *hsa-miR-373-3p.3.P1.S.42* | 0 | 0 | 0 | 1 |
|  | *hsa-miR-373-3p.3.P1.S.43* | 0 | 0 | 0 | 1 |
|  | *hsa-miR-373-3p.3.P1.S.44* | 0 | 0 | 0 | 2 |
|  | *hsa-miR-373-3p.3.P1.S.45* | 0 | 0 | 0 | 1 |
|  | *hsa-miR-373-3p.3.P1.S.46* | 0 | 0 | 0 | 3 |
|  | *hsa-miR-373-3p.3.P1.S.47* | 0 | 0 | 0 | 1 |
|  | *hsa-miR-373-3p.3.P1.S.48* | 0 | 0 | 0 | 182 |
|  | *hsa-miR-373-3p.3.P1.S.49* | 0 | 0 | 0 | 111 |
|  | *hsa-miR-373-3p.3.P1.S.50* | 0 | 0 | 0 | 32 |
|  | *hsa-miR-373-3p.3.P0.S.14* | 29 | 0 | 57 | 15196 |
|  | *hsa-miR-373-3p.3.P1.S.51* | 0 | 0 | 0 | 3 |
|  | *hsa-miR-373-3p.3.P1.S.52* | 0 | 0 | 0 | 4 |
|  | *hsa-miR-373-3p.3.P1.S.53* | 0 | 0 | 0 | 15 |
|  | *hsa-miR-373-3p.3.P1.S.54* | 0 | 0 | 0 | 1 |
|  | *hsa-miR-373-3p.3.P1.S.55* | 0 | 0 | 0 | 2 |
|  | *hsa-miR-373-3p.3.P1.S.56* | 0 | 0 | 0 | 5 |
|  | *hsa-miR-373-3p.3.P1.S.57* | 0 | 0 | 0 | 1 |
|  | *hsa-miR-373-3p.3.P1.S.58* | 0 | 0 | 0 | 9 |
|  | *hsa-miR-373-3p.3.P1.S.59* | 0 | 0 | 0 | 1 |
|  | *hsa-miR-373-3p.3.P1.S.60* | 0 | 0 | 0 | 4 |
|  | *hsa-miR-373-3p.3.P1.S.61* | 0 | 0 | 0 | 3 |
|  | *hsa-miR-373-3p.3.P1.S.62* | 0 | 0 | 0 | 11 |
|  | *hsa-miR-373-3p.3.P1.S.63* | 0 | 0 | 0 | 2 |
|  | *hsa-miR-373-3p.3.P1.S.64* | 0 | 0 | 0 | 4 |
|  | *hsa-miR-373-3p.3.P1.S.65* | 0 | 0 | 0 | 7 |
|  | *hsa-miR-373-3p.3.P1.S.66* | 0 | 0 | 0 | 7 |
|  | *hsa-miR-373-3p.3.P1.S.67* | 0 | 0 | 0 | 2 |
|  | *hsa-miR-373-3p.3.P1.S.68* | 0 | 0 | 0 | 6 |
|  | *hsa-miR-373-3p.3.P1.S.69* | 0 | 0 | 0 | 13 |
|  | *hsa-miR-373-3p.3.P1.S.70* | 0 | 0 | 0 | 29 |
|  | *hsa-miR-373-3p.3.P1.S.71* | 0 | 0 | 0 | 8 |
|  | *hsa-miR-373-3p.3.P1.S.72* | 0 | 0 | 0 | 21 |
|  | *hsa-miR-373-3p.3.P1.S.73* | 0 | 0 | 0 | 21 |
|  | *hsa-miR-373-3p.3.P1.S.74* | 0 | 0 | 0 | 2 |
|  | *hsa-miR-373-3p.3.P1.S.75* | 0 | 0 | 0 | 1 |
|  | *hsa-miR-373-3p.3.P1.S.76* | 0 | 0 | 0 | 4 |
|  | *hsa-miR-373-3p.3.P1.S.77* | 0 | 0 | 0 | 1 |
|  | *hsa-miR-373-3p.3.P1.S.78* | 0 | 0 | 0 | 3 |
|  | *hsa-miR-373-3p.3.P1.S.79* | 0 | 0 | 0 | 4 |
|  | *hsa-miR-373-3p.3.P1.S.80* | 0 | 0 | 3 | 13 |
|  | *hsa-miR-373-3p.3.P1.S.81* | 0 | 0 | 0 | 11 |
|  | *hsa-miR-373-3p.3.P1.S.82* | 0 | 0 | 0 | 1 |
|  | *hsa-miR-373-3p.3.P1.S.83* | 0 | 0 | 0 | 4 |
|  | *hsa-miR-373-3p.3.P1.S.84* | 0 | 0 | 0 | 8 |
|  | *hsa-miR-373-3p.3.P1.S.85* | 0 | 0 | 0 | 1 |
|  | *hsa-miR-373-3p.3.P1.S.86* | 0 | 0 | 0 | 16 |
|  | *hsa-miR-373-3p.3.P1.S.87* | 0 | 0 | 15 | 1479 |
|  | *hsa-miR-373-3p.3.P1.S.88* | 0 | 9 | 0 | 23 |
|  | *hsa-miR-373-3p.3.P1.S.89* | 0 | 0 | 0 | 1 |
|  | *hsa-miR-373-3p.3.P0.S.15* | 19 | 0 | 5 | 1703 |
|  | *hsa-miR-373-3p.3.P1.S.90* | 19 | 0 | 0 | 0 |
|  | *hsa-miR-373-3p.3.P1.S.91* | 0 | 0 | 0 | 1 |
|  | *hsa-miR-373-3p.3.P1.S.92* | 0 | 0 | 0 | 2 |
|  | *hsa-miR-373-3p.3.P1.S.93* | 0 | 0 | 0 | 1 |
|  | *hsa-miR-373-3p.3.P1.S.94* | 0 | 0 | 0 | 3 |
|  | *hsa-miR-373-3p.3.P1.S.95* | 0 | 0 | 0 | 1 |
|  | *hsa-miR-373-3p.3.P1.S.96* | 0 | 0 | 0 | 2 |
|  | *hsa-miR-373-3p.3.P1.S.97* | 0 | 0 | 0 | 1 |
|  | *hsa-miR-373-3p.3.P1.S.98* | 0 | 0 | 0 | 2 |
|  | *hsa-miR-373-3p.3.P1.S.99* | 0 | 0 | 0 | 3 |
|  | *hsa-miR-373-3p.3.P1.S.100* | 0 | 0 | 0 | 2 |
|  | *hsa-miR-373-3p.3.P1.S.101* | 0 | 0 | 0 | 7 |
|  | *hsa-miR-373-3p.3.P1.S.102* | 0 | 0 | 0 | 3 |
|  | *hsa-miR-373-3p.3.P1.S.103* | 0 | 0 | 0 | 1 |
|  | *hsa-miR-373-3p.3.P1.S.104* | 0 | 0 | 0 | 1 |
|  | *hsa-miR-373-3p.3.P1.S.105* | 0 | 0 | 0 | 2 |
|  | *hsa-miR-373-3p.3.P1.S.106* | 0 | 0 | 0 | 2 |
|  | *hsa-miR-373-3p.3.P1.S.107* | 0 | 0 | 0 | 1 |
|  | *hsa-miR-373-3p.3.P1.S.108* | 0 | 0 | 0 | 3 |
|  | *hsa-miR-373-3p.3.P1.S.109* | 0 | 0 | 0 | 1 |
|  | *hsa-miR-373-3p.3.P1.S.110* | 0 | 0 | 5 | 253 |
|  | *hsa-miR-373-3p.3.P1.S.111* | 0 | 0 | 0 | 1 |
|  | *hsa-miR-373-3p.3.P1.S.112* | 0 | 0 | 0 | 3 |
|  | *hsa-miR-373-3p.3.P1.S.113* | 23 | 0 | 20 | 725 |
|  | *hsa-miR-373-3p.3.P1.S.114* | 0 | 0 | 0 | 3 |
|  | *hsa-miR-373-3p.3.P1.S.115* | 0 | 5 | 0 | 130 |
|  | *hsa-miR-373-3p.3.P0.S.16* | 19 | 11 | 83 | 9207 |
|  | *hsa-miR-373-3p.3.P1.S.116* | 0 | 0 | 0 | 1 |
|  | *hsa-miR-373-3p.3.P1.S.117* | 0 | 0 | 0 | 5 |
|  | *hsa-miR-373-3p.3.P1.S.118* | 0 | 0 | 0 | 2 |
|  | *hsa-miR-373-3p.3.P1.S.119* | 0 | 0 | 0 | 2 |
|  | *hsa-miR-373-3p.3.P1.S.120* | 0 | 0 | 0 | 3 |
|  | *hsa-miR-373-3p.3.P1.S.121* | 0 | 0 | 0 | 1 |
|  | *hsa-miR-373-3p.3.P1.S.122* | 0 | 0 | 0 | 5 |
|  | *hsa-miR-373-3p.3.P1.S.123* | 0 | 0 | 0 | 6 |
|  | *hsa-miR-373-3p.3.P1.S.124* | 0 | 0 | 0 | 1 |
|  | *hsa-miR-373-3p.3.P1.S.125* | 0 | 0 | 0 | 1 |
|  | *hsa-miR-373-3p.3.P1.S.126* | 0 | 0 | 0 | 2 |
|  | *hsa-miR-373-3p.3.P1.S.127* | 0 | 0 | 0 | 5 |
|  | *hsa-miR-373-3p.3.P1.S.128* | 0 | 0 | 0 | 2 |
|  | *hsa-miR-373-3p.3.P1.S.129* | 0 | 0 | 0 | 1 |
|  | *hsa-miR-373-3p.3.P1.S.130* | 0 | 0 | 0 | 6 |
|  | *hsa-miR-373-3p.3.P1.S.131* | 0 | 0 | 0 | 4 |
|  | *hsa-miR-373-3p.3.P1.S.132* | 0 | 0 | 0 | 4 |
|  | *hsa-miR-373-3p.3.P1.S.133* | 0 | 0 | 0 | 16 |
|  | *hsa-miR-373-3p.3.P1.S.134* | 0 | 0 | 3 | 23 |
|  | *hsa-miR-373-3p.3.P1.S.135* | 0 | 0 | 0 | 9 |
|  | *hsa-miR-373-3p.3.P1.S.136* | 0 | 0 | 0 | 20 |
|  | *hsa-miR-373-3p.3.P1.S.137* | 19 | 0 | 0 | 22 |
|  | *hsa-miR-373-3p.3.P1.S.138* | 0 | 0 | 0 | 17 |
|  | *hsa-miR-373-3p.3.P1.S.139* | 0 | 0 | 0 | 1 |
|  | *hsa-miR-373-3p.3.P1.S.140* | 0 | 0 | 0 | 1 |
|  | *hsa-miR-373-3p.3.P1.S.141* | 0 | 0 | 0 | 1 |
|  | *hsa-miR-373-3p.3.P1.S.142* | 0 | 0 | 0 | 4 |
|  | *hsa-miR-373-3p.3.P1.S.143* | 0 | 0 | 0 | 29 |
|  | *hsa-miR-373-3p.3.P1.S.144* | 0 | 0 | 0 | 11 |
|  | *hsa-miR-373-3p.3.P1.S.145* | 0 | 0 | 0 | 47 |
|  | *hsa-miR-373-3p.3.P1.S.146* | 0 | 0 | 0 | 24 |
|  | *hsa-miR-373-3p.3.P1.S.147* | 0 | 0 | 0 | 13 |
|  | *hsa-miR-373-3p.3.P1.S.148* | 0 | 0 | 0 | 5 |
|  | *hsa-miR-373-3p.3.P1.S.149* | 0 | 0 | 0 | 1 |
|  | *hsa-miR-373-3p.3.P1.S.150* | 0 | 0 | 0 | 6 |
|  | *hsa-miR-373-3p.3.P1.S.151* | 0 | 0 | 0 | 7 |
|  | *hsa-miR-373-3p.3.P1.S.152* | 0 | 0 | 0 | 2 |
|  | *hsa-miR-373-3p.3.P1.S.153* | 0 | 0 | 0 | 3 |
|  | *hsa-miR-373-3p.3.P1.S.154* | 0 | 0 | 0 | 3 |
|  | *hsa-miR-373-3p.3.P1.S.155* | 0 | 0 | 0 | 68 |
|  | *hsa-miR-373-3p.3.P1.S.156* | 0 | 0 | 0 | 67 |
|  | *hsa-miR-373-3p.3.P1.S.157* | 3 | 0 | 3 | 464 |
|  | *hsa-miR-373-3p.53.P0.S.0* | 0 | 0 | 0 | 1 |
|  | *hsa-miR-373-3p.5.P0.S.0* | 0 | 0 | 0 | 5 |
|  | *hsa-miR-373-3p.5.P0.S.1* | 0 | 0 | 0 | 1 |
|  | *hsa-miR-373-3p.53.P0.S.1* | 0 | 0 | 0 | 1 |
|  | *hsa-miR-373-3p.53.P0.S.2* | 0 | 0 | 0 | 6 |
|  | *hsa-miR-373-3p.53.P0.S.3* | 0 | 0 | 0 | 1 |
|  | *hsa-miR-373-3p.53.P0.S.4* | 0 | 0 | 0 | 5 |
|  | *hsa-miR-373-3p.5.P0.S.2* | 0 | 0 | 0 | 19 |
|  | *hsa-miR-373-3p.53.P0.S.5* | 0 | 0 | 0 | 1 |
|  | *hsa-miR-373-3p.53.P0.S.6* | 0 | 0 | 3 | 90 |
|  | *hsa-miR-373-3p.53.P0.S.7* | 0 | 0 | 0 | 75 |
|  | *hsa-miR-373-3p.53.P0.S.8* | 0 | 0 | 0 | 4 |
|  | *hsa-miR-373-3p.53.P0.S.9* | 0 | 0 | 0 | 68 |
|  | *hsa-miR-373-3p.53.P0.S.10* | 0 | 0 | 0 | 57 |
|  | *hsa-miR-373-3p.53.P1.S.0* | 0 | 0 | 0 | 2 |
|  | *hsa-miR-373-3p.53.P1.S.1* | 0 | 0 | 0 | 1 |
|  | *hsa-miR-373-3p.53.P0.S.11* | 0 | 0 | 0 | 6 |
|  | *hsa-miR-373-3p.53.P1.S.2* | 0 | 0 | 0 | 1 |
|  | *hsa-miR-373-3p.53.P0.S.12* | 0 | 0 | 0 | 111 |
|  | *hsa-miR-373-3p.53.P1.S.3* | 0 | 0 | 0 | 1 |
|  | *hsa-miR-373-3p.53.P1.S.4* | 0 | 0 | 0 | 16 |
|  | *hsa-miR-373-3p.53.P1.S.5* | 0 | 0 | 0 | 2 |
|  | *hsa-miR-373-3p.53.P1.S.6* | 0 | 0 | 0 | 3 |
|  | *hsa-miR-373-3p.53.P0.S.13* | 0 | 0 | 0 | 12 |
|  | *hsa-miR-373-3p.53.P1.S.7* | 0 | 0 | 0 | 1 |
|  | *hsa-miR-373-3p.53.P1.S.8* | 0 | 0 | 0 | 9 |
|  | *hsa-miR-373-3p.53.P1.S.9* | 0 | 0 | 0 | 1 |
|  | *hsa-miR-373-3p.53.P0.S.14* | 0 | 0 | 0 | 85 |
|  | *hsa-miR-373-3p.53.P1.S.10* | 0 | 0 | 0 | 1 |
|  | *hsa-miR-373-3p.53.P1.S.11* | 0 | 0 | 0 | 1 |
|  | *hsa-miR-373-3p.53.P1.S.12* | 0 | 0 | 0 | 1 |
|  | *hsa-miR-373-3p.53.P1.S.13* | 0 | 0 | 0 | 1 |
|  | *hsa-miR-373-3p.53.P1.S.14* | 0 | 0 | 0 | 2 |
|  | *hsa-miR-373-3p.53.P1.S.15* | 0 | 0 | 0 | 3 |
|  | *hsa-miR-373-3p.53.P0.S.15* | 0 | 0 | 0 | 2 |
|  | *hsa-miR-373-3p.53.P0.S.16* | 0 | 0 | 0 | 3 |
|  | *hsa-miR-373-3p.53.P1.S.16* | 0 | 0 | 0 | 1 |
|  | *hsa-miR-373-3p.53.P0.S.17* | 0 | 0 | 0 | 1 |
|  | *hsa-miR-373-3p.53.P0.S.18* | 0 | 0 | 0 | 4 |
|  | *hsa-miR-373-3p.53.P0.S.19* | 0 | 0 | 0 | 4 |
|  | *hsa-miR-373-3p.53.P1.S.17* | 0 | 0 | 0 | 1 |
|  | *hsa-miR-373-3p.53.P0.S.20* | 0 | 0 | 0 | 4 |
|  | *hsa-miR-373-3p.53.P1.S.18* | 0 | 0 | 0 | 2 |
|  | *hsa-miR-373-3p.5.P0.S.3* | 0 | 0 | 0 | 23 |
|  | *hsa-miR-373-3p.5.P1.S.0* | 0 | 0 | 0 | 1 |
|  | *hsa-miR-373-3p.5.P1.S.1* | 0 | 0 | 0 | 1 |
|  | *hsa-miR-373-3p.5.P1.S.2* | 0 | 0 | 0 | 1 |
|  | *hsa-miR-373-3p.53.P0.S.21* | 0 | 0 | 0 | 1 |
|  | *hsa-miR-373-3p.53.P0.S.22* | 0 | 0 | 0 | 2 |
|  | *hsa-miR-373-3p.53.P0.S.23* | 3 | 0 | 0 | 24 |
|  | *hsa-miR-373-3p.53.P1.S.19* | 0 | 0 | 0 | 1 |
|  | *hsa-miR-373-3p.53.P1.S.20* | 0 | 0 | 0 | 2 |
|  | *hsa-miR-373-3p.53.P0.S.24* | 0 | 0 | 0 | 1 |
|  | *hsa-miR-373-3p.53.P0.S.25* | 0 | 0 | 0 | 2 |
|  | *hsa-miR-373-3p.5.P0.S.4* | 0 | 0 | 0 | 94 |
|  | *hsa-miR-373-3p.5.P1.S.3* | 0 | 0 | 0 | 1 |
|  | *hsa-miR-373-3p.5.P1.S.4* | 0 | 0 | 0 | 1 |
|  | *hsa-miR-373-3p.5.P1.S.5* | 0 | 0 | 0 | 1 |
|  | *hsa-miR-373-3p.5.P1.S.6* | 0 | 0 | 0 | 6 |
|  | *hsa-miR-373-3p.5.P1.S.7* | 0 | 0 | 0 | 1 |
|  | *hsa-miR-373-3p.5.P1.S.8* | 0 | 0 | 0 | 2 |
|  | *hsa-miR-373-3p.5.P0.S.5* | 0 | 0 | 0 | 70 |
|  | *hsa-miR-373-3p.5.P1.S.9* | 0 | 0 | 0 | 1 |
|  | *hsa-miR-373-3p.5.P1.S.10* | 0 | 0 | 0 | 1 |
|  | *hsa-miR-373-3p.5.P1.S.11* | 0 | 0 | 0 | 1 |
|  | *hsa-miR-373-3p.5.P0.S.6* | 0 | 0 | 0 | 79 |
|  | *hsa-miR-373-3p.5.P1.S.12* | 0 | 0 | 0 | 1 |
|  | *hsa-miR-373-3p.5.P1.S.13* | 0 | 0 | 0 | 1 |
|  | *hsa-miR-373-3p.5.P1.S.14* | 0 | 0 | 0 | 4 |
|  | *hsa-miR-373-3p.5.P0.S.7* | 0 | 7 | 0 | 2883 |
|  | *hsa-miR-373-3p.5.P0.S.8* | 0 | 0 | 0 | 3 |
|  | *hsa-miR-373-3p.5.P1.S.15* | 0 | 0 | 0 | 3 |
|  | *hsa-miR-373-3p.5.P1.S.16* | 0 | 0 | 0 | 2 |
|  | *hsa-miR-373-3p.5.P1.S.17* | 0 | 0 | 0 | 1 |
|  | *hsa-miR-373-3p.5.P1.S.18* | 0 | 0 | 0 | 2 |
|  | *hsa-miR-373-3p.5.P1.S.19* | 0 | 0 | 0 | 4 |
|  | *hsa-miR-373-3p.5.P1.S.20* | 0 | 0 | 0 | 1 |
|  | *hsa-miR-373-3p.5.P1.S.21* | 0 | 0 | 0 | 4 |
|  | *hsa-miR-373-3p.5.P1.S.22* | 0 | 0 | 0 | 2 |
|  | *hsa-miR-373-3p.5.P1.S.23* | 0 | 0 | 0 | 2 |
|  | *hsa-miR-373-3p.5.P1.S.24* | 0 | 0 | 0 | 1 |
|  | *hsa-miR-373-3p.5.P1.S.25* | 0 | 0 | 0 | 3 |
|  | *hsa-miR-373-3p.5.P1.S.26* | 0 | 0 | 0 | 5 |
|  | *hsa-miR-373-3p.5.P1.S.27* | 0 | 0 | 0 | 1 |
|  | *hsa-miR-373-3p.5.P1.S.28* | 0 | 0 | 0 | 1 |
|  | *hsa-miR-373-3p.5.P1.S.29* | 0 | 0 | 0 | 3 |
|  | *hsa-miR-373-3p.5.P1.S.30* | 0 | 0 | 0 | 2 |
|  | *hsa-miR-373-3p.5.P1.S.31* | 0 | 0 | 0 | 1 |
|  | *hsa-miR-373-3p.5.P1.S.32* | 0 | 0 | 0 | 3 |
|  | *hsa-miR-373-3p.5.P1.S.33* | 0 | 0 | 0 | 4 |
|  | *hsa-miR-373-3p.5.P1.S.34* | 0 | 0 | 0 | 4 |
|  | *hsa-miR-373-3p.5.P1.S.35* | 0 | 0 | 0 | 1 |
|  | *hsa-miR-373-3p.5.P1.S.36* | 0 | 0 | 0 | 2 |
|  | *hsa-miR-373-3p.5.P1.S.37* | 0 | 0 | 0 | 1 |
|  | *hsa-miR-373-3p.5.P1.S.38* | 0 | 0 | 0 | 1 |
|  | *hsa-miR-373-3p.5.P1.S.39* | 0 | 0 | 0 | 1 |
|  | *hsa-miR-373-3p.5.P1.S.40* | 0 | 0 | 0 | 1 |
|  | *hsa-miR-373-3p.5.P1.S.41* | 0 | 0 | 0 | 1 |
|  | *hsa-miR-373-3p.5.P1.S.42* | 0 | 0 | 0 | 1 |
|  | *hsa-miR-373-3p.5.P1.S.43* | 0 | 0 | 0 | 1 |
|  | *hsa-miR-373-3p.5.P1.S.44* | 0 | 0 | 0 | 1 |
|  | *hsa-miR-373-3p.5.P1.S.45* | 0 | 0 | 0 | 3 |
|  | *hsa-miR-373-3p.5.P1.S.46* | 0 | 0 | 0 | 5 |
|  | *hsa-miR-373-3p.5.P1.S.47* | 0 | 0 | 0 | 76 |
|  | *hsa-miR-373-3p.5.P1.S.48* | 0 | 0 | 0 | 9 |
|  | *hsa-miR-373-3p.5.P1.S.49* | 0 | 0 | 0 | 25 |
|  | *hsa-miR-373-3p.0.P1.S.0* | 0 | 0 | 0 | 52 |
|  | *hsa-miR-373-3p.0.P1.S.1* | 3 | 0 | 0 | 5 |
|  | *hsa-miR-373-3p.0.P1.S.2* | 0 | 0 | 0 | 8 |
|  | *hsa-miR-373-3p.0.P1.S.3* | 0 | 0 | 0 | 4 |
|  | *hsa-miR-373-3p.0.P1.S.4* | 0 | 0 | 0 | 71 |
|  | *hsa-miR-373-3p.0.P1.S.5* | 0 | 0 | 0 | 5 |
|  | *hsa-miR-373-3p.0.P1.S.6* | 0 | 0 | 0 | 2 |
|  | *hsa-miR-373-3p.0.P1.S.7* | 0 | 0 | 0 | 305 |
|  | *hsa-miR-373-3p.0.P1.S.8* | 0 | 0 | 0 | 1 |
|  | *hsa-miR-373-3p.0.P1.S.9* | 0 | 0 | 0 | 57 |
|  | *hsa-miR-373-3p.0.P1.S.10* | 0 | 0 | 5 | 2 |
|  | *hsa-miR-373-3p.0.P1.S.11* | 0 | 0 | 0 | 2 |
|  | *hsa-miR-373-3p.0.P1.S.12* | 0 | 0 | 0 | 1 |
|  | *hsa-miR-373-3p.0.P1.S.13* | 0 | 0 | 0 | 14 |
|  | *hsa-miR-373-3p.0.P1.S.14* | 0 | 0 | 0 | 2 |
|  | *hsa-miR-373-3p.0.P1.S.15* | 0 | 0 | 0 | 63 |
|  | *hsa-miR-373-3p.0.P1.S.16* | 0 | 0 | 0 | 9 |
|  | *hsa-miR-373-3p.0.P1.S.17* | 0 | 0 | 0 | 3 |
|  | *hsa-miR-373-3p.0.P1.S.18* | 0 | 0 | 0 | 3 |
|  | *hsa-miR-373-3p.0.P1.S.19* | 0 | 0 | 0 | 79 |
|  | *hsa-miR-373-3p.0.P1.S.20* | 0 | 0 | 0 | 1 |
|  | *hsa-miR-373-3p.0.P1.S.21* | 0 | 0 | 10 | 234 |
|  | *hsa-miR-373-3p.0.P1.S.22* | 0 | 0 | 0 | 7 |
|  | *hsa-miR-373-3p.0.P1.S.23* | 0 | 0 | 0 | 8 |
|  | *hsa-miR-373-3p.0.P1.S.24* | 0 | 0 | 0 | 38 |
|  | *hsa-miR-373-3p.0.P1.S.25* | 0 | 0 | 0 | 13 |
|  | *hsa-miR-373-3p.0.P1.S.26* | 0 | 0 | 0 | 3 |
|  | *hsa-miR-373-3p.0.P1.S.27* | 0 | 0 | 0 | 99 |
|  | *hsa-miR-373-3p.0.P1.S.28* | 0 | 0 | 0 | 58 |
|  | *hsa-miR-373-3p.0.P1.S.29* | 0 | 0 | 0 | 2 |
|  | *hsa-miR-373-3p.0.P1.S.30* | 0 | 0 | 14 | 52 |
|  | *hsa-miR-373-3p.0.P1.S.31* | 19 | 0 | 0 | 87 |
|  | *hsa-miR-373-3p.0.P1.S.32* | 0 | 0 | 0 | 99 |
|  | *hsa-miR-373-3p.0.P1.S.33* | 0 | 0 | 0 | 45 |
|  | *hsa-miR-373-3p.0.P1.S.34* | 0 | 0 | 0 | 121 |
|  | *hsa-miR-373-3p.0.P1.S.35* | 0 | 0 | 0 | 332 |
|  | *hsa-miR-373-3p.0.P1.S.36* | 0 | 0 | 0 | 206 |
|  | *hsa-miR-373-3p.0.P1.S.37* | 0 | 0 | 0 | 399 |
|  | *hsa-miR-373-3p.0.P1.S.38* | 19 | 0 | 0 | 469 |
|  | *hsa-miR-373-3p.0.P1.S.39* | 0 | 0 | 0 | 340 |
|  | *hsa-miR-373-3p.0.P1.S.40* | 0 | 0 | 0 | 3 |
|  | *hsa-miR-373-3p.0.P1.S.41* | 0 | 0 | 0 | 26 |
|  | *hsa-miR-373-3p.0.P1.S.42* | 0 | 0 | 0 | 10 |
|  | *hsa-miR-373-3p.0.P1.S.43* | 0 | 0 | 0 | 3 |
|  | *hsa-miR-373-3p.0.P1.S.44* | 0 | 0 | 0 | 69 |
|  | *hsa-miR-373-3p.0.P1.S.45* | 0 | 0 | 0 | 24 |
|  | *hsa-miR-373-3p.0.P1.S.46* | 0 | 0 | 0 | 56 |
|  | *hsa-miR-373-3p.0.P1.S.47* | 19 | 0 | 0 | 76 |
|  | *hsa-miR-373-3p.0.P1.S.48* | 0 | 0 | 0 | 78 |
|  | *hsa-miR-373-3p.0.P1.S.49* | 19 | 0 | 0 | 291 |
|  | *hsa-miR-373-3p.0.P1.S.50* | 19 | 0 | 0 | 191 |
|  | *hsa-miR-373-3p.0.P1.S.51* | 0 | 0 | 0 | 88 |
|  | *hsa-miR-373-3p.0.P1.S.52* | 0 | 0 | 0 | 204 |
|  | *hsa-miR-373-3p.0.P1.S.53* | 0 | 0 | 0 | 8 |
|  | *hsa-miR-373-3p.0.P1.S.54* | 19 | 0 | 0 | 17 |
|  | *hsa-miR-373-3p.0.P1.S.55* | 0 | 0 | 0 | 56 |
|  | *hsa-miR-373-3p.0.P1.S.56* | 0 | 0 | 0 | 6 |
|  | *hsa-miR-373-3p.0.P1.S.57* | 0 | 0 | 0 | 10 |
|  | *hsa-miR-373-3p.0.P1.S.58* | 0 | 0 | 0 | 181 |
|  | *hsa-miR-373-3p.0.P1.S.59* | 0 | 0 | 0 | 9 |
|  | *hsa-miR-373-3p.0.P1.S.60* | 0 | 0 | 0 | 365 |
|  | *hsa-miR-373-3p.0.P1.S.61* | 0 | 0 | 0 | 24 |
|  | *hsa-miR-373-3p.0.P1.S.62* | 0 | 0 | 0 | 183 |
|  | *hsa-miR-373-3p.0.P1.S.63* | 42 | 0 | 59 | 10093 |
|  | *hsa-miR-373-3p.0.P1.S.64* | 3 | 16 | 3 | 486 |
|  | *hsa-miR-373-3p.0.P1.S.65* | 0 | 0 | 0 | 515 |
| ***hsa-miR-372-3p*** | *hsa-miR-372-3p.3.P0.S.0* | 0 | 0 | 0 | 74 |
|  | *hsa-miR-372-3p.3.P0.S.1* | 0 | 0 | 0 | 19 |
|  | *hsa-miR-372-3p.3.P0.S.2* | 0 | 0 | 0 | 1 |
|  | *hsa-miR-372-3p.3.P0.S.3* | 0 | 0 | 0 | 8 |
|  | *hsa-miR-372-3p.3.P0.S.4* | 0 | 0 | 0 | 2 |
|  | *hsa-miR-372-3p.3.P0.S.5* | 0 | 0 | 0 | 9 |
|  | *hsa-miR-372-3p.3.P0.S.6* | 0 | 0 | 0 | 121 |
|  | *hsa-miR-372-3p.3.P0.S.7* | 0 | 0 | 0 | 14 |
|  | *hsa-miR-372-3p.3.P0.S.8* | 19 | 0 | 0 | 482 |
|  | *hsa-miR-372-3p.3.P0.S.9* | 0 | 0 | 0 | 48 |
|  | *hsa-miR-372-3p.3.P1.S.0* | 0 | 0 | 0 | 2 |
|  | *hsa-miR-372-3p.3.P1.S.1* | 0 | 0 | 0 | 1 |
|  | *hsa-miR-372-3p.3.P1.S.2* | 0 | 0 | 0 | 1 |
|  | *hsa-miR-372-3p.3.P1.S.3* | 0 | 0 | 0 | 80 |
|  | *hsa-miR-372-3p.3.P1.S.4* | 0 | 0 | 0 | 2 |
|  | *hsa-miR-372-3p.3.P1.S.5* | 0 | 0 | 0 | 6 |
|  | *hsa-miR-372-3p.3.P1.S.6* | 0 | 0 | 0 | 11 |
|  | *hsa-miR-372-3p.3.P0.S.10* | 0 | 0 | 0 | 1 |
|  | *hsa-miR-372-3p.3.P0.S.11* | 0 | 0 | 0 | 1 |
|  | *hsa-miR-372-3p.3.P0.S.12* | 0 | 0 | 0 | 537 |
|  | *hsa-miR-372-3p.3.P1.S.7* | 0 | 0 | 0 | 1 |
|  | *hsa-miR-372-3p.3.P1.S.8* | 0 | 0 | 0 | 2 |
|  | *hsa-miR-372-3p.3.P1.S.9* | 0 | 0 | 0 | 1 |
|  | *hsa-miR-372-3p.3.P1.S.10* | 0 | 0 | 0 | 1 |
|  | *hsa-miR-372-3p.3.P1.S.11* | 0 | 0 | 0 | 1 |
|  | *hsa-miR-372-3p.3.P1.S.12* | 0 | 0 | 0 | 1 |
|  | *hsa-miR-372-3p.3.P1.S.13* | 0 | 0 | 0 | 1 |
|  | *hsa-miR-372-3p.3.P1.S.14* | 0 | 0 | 0 | 2 |
|  | *hsa-miR-372-3p.3.P1.S.15* | 0 | 0 | 0 | 1 |
|  | *hsa-miR-372-3p.3.P1.S.16* | 0 | 0 | 0 | 1 |
|  | *hsa-miR-372-3p.3.P1.S.17* | 0 | 0 | 0 | 5 |
|  | *hsa-miR-372-3p.3.P1.S.18* | 0 | 0 | 0 | 4 |
|  | *hsa-miR-372-3p.3.P1.S.19* | 0 | 0 | 0 | 28 |
|  | *hsa-miR-372-3p.3.P0.S.13* | 19 | 0 | 5 | 688 |
|  | *hsa-miR-372-3p.3.P1.S.20* | 0 | 0 | 0 | 1 |
|  | *hsa-miR-372-3p.3.P1.S.21* | 0 | 0 | 0 | 1 |
|  | *hsa-miR-372-3p.3.P1.S.22* | 0 | 0 | 0 | 2 |
|  | *hsa-miR-372-3p.3.P1.S.23* | 0 | 0 | 0 | 20 |
|  | *hsa-miR-372-3p.3.P0.S.14* | 0 | 0 | 0 | 685 |
|  | *hsa-miR-372-3p.3.P1.S.24* | 0 | 0 | 0 | 1 |
|  | *hsa-miR-372-3p.3.P1.S.25* | 0 | 0 | 0 | 1 |
|  | *hsa-miR-372-3p.3.P1.S.26* | 0 | 0 | 0 | 1 |
|  | *hsa-miR-372-3p.3.P1.S.27* | 0 | 0 | 0 | 2 |
|  | *hsa-miR-372-3p.3.P1.S.28* | 0 | 0 | 0 | 1 |
|  | *hsa-miR-372-3p.3.P1.S.29* | 0 | 0 | 0 | 1 |
|  | *hsa-miR-372-3p.3.P1.S.30* | 0 | 0 | 0 | 1 |
|  | *hsa-miR-372-3p.3.P1.S.31* | 0 | 0 | 0 | 1 |
|  | *hsa-miR-372-3p.3.P1.S.32* | 0 | 0 | 0 | 1 |
|  | *hsa-miR-372-3p.3.P1.S.33* | 0 | 0 | 0 | 1 |
|  | *hsa-miR-372-3p.3.P1.S.34* | 0 | 0 | 0 | 4 |
|  | *hsa-miR-372-3p.3.P1.S.35* | 0 | 0 | 0 | 6 |
|  | *hsa-miR-372-3p.3.P1.S.36* | 0 | 0 | 0 | 10 |
|  | *hsa-miR-372-3p.3.P1.S.37* | 0 | 0 | 0 | 165 |
|  | *hsa-miR-372-3p.3.P0.S.15* | 0 | 0 | 0 | 475 |
|  | *hsa-miR-372-3p.3.P1.S.38* | 0 | 0 | 0 | 1 |
|  | *hsa-miR-372-3p.3.P1.S.39* | 0 | 0 | 0 | 2 |
|  | *hsa-miR-372-3p.3.P1.S.40* | 0 | 0 | 0 | 1 |
|  | *hsa-miR-372-3p.3.P1.S.41* | 0 | 0 | 0 | 3 |
|  | *hsa-miR-372-3p.3.P1.S.42* | 0 | 0 | 0 | 1 |
|  | *hsa-miR-372-3p.3.P1.S.43* | 0 | 0 | 0 | 1 |
|  | *hsa-miR-372-3p.3.P1.S.44* | 0 | 0 | 0 | 1 |
|  | *hsa-miR-372-3p.3.P1.S.45* | 0 | 0 | 0 | 44 |
|  | *hsa-miR-372-3p.3.P1.S.46* | 0 | 0 | 0 | 2 |
|  | *hsa-miR-372-3p.3.P1.S.47* | 0 | 0 | 0 | 1 |
|  | *hsa-miR-372-3p.3.P0.S.16* | 0 | 0 | 0 | 748 |
|  | *hsa-miR-372-3p.3.P1.S.48* | 0 | 0 | 0 | 1 |
|  | *hsa-miR-372-3p.3.P1.S.49* | 0 | 0 | 0 | 1 |
|  | *hsa-miR-372-3p.3.P1.S.50* | 0 | 0 | 0 | 1 |
|  | *hsa-miR-372-3p.3.P1.S.51* | 0 | 0 | 0 | 1 |
|  | *hsa-miR-372-3p.3.P1.S.52* | 0 | 0 | 0 | 2 |
|  | *hsa-miR-372-3p.3.P1.S.53* | 0 | 0 | 0 | 1 |
|  | *hsa-miR-372-3p.3.P1.S.54* | 0 | 0 | 0 | 3 |
|  | *hsa-miR-372-3p.3.P1.S.55* | 0 | 0 | 0 | 1 |
|  | *hsa-miR-372-3p.3.P1.S.56* | 0 | 0 | 0 | 7 |
|  | *hsa-miR-372-3p.3.P1.S.57* | 0 | 0 | 0 | 9 |
|  | *hsa-miR-372-3p.53.P0.S.0* | 0 | 0 | 0 | 1 |
|  | *hsa-miR-372-3p.5.P0.S.0* | 0 | 0 | 0 | 2 |
|  | *hsa-miR-372-3p.5.P0.S.1* | 0 | 0 | 0 | 2 |
|  | *hsa-miR-372-3p.53.P0.S.1* | 0 | 0 | 0 | 2 |
|  | *hsa-miR-372-3p.53.P0.S.2* | 0 | 0 | 0 | 1 |
|  | *hsa-miR-372-3p.53.P0.S.3* | 0 | 0 | 0 | 2 |
|  | *hsa-miR-372-3p.53.P0.S.4* | 0 | 0 | 0 | 2 |
|  | *hsa-miR-372-3p.5.P0.S.2* | 0 | 0 | 0 | 2 |
|  | *hsa-miR-372-3p.5.P0.S.3* | 0 | 0 | 0 | 9 |
|  | *hsa-miR-372-3p.53.P0.S.5* | 0 | 0 | 0 | 13 |
|  | *hsa-miR-372-3p.53.P0.S.6* | 0 | 0 | 0 | 6 |
|  | *hsa-miR-372-3p.53.P0.S.7* | 0 | 0 | 0 | 2 |
|  | *hsa-miR-372-3p.53.P0.S.8* | 0 | 0 | 0 | 1 |
|  | *hsa-miR-372-3p.53.P0.S.9* | 0 | 0 | 0 | 1 |
|  | *hsa-miR-372-3p.53.P0.S.10* | 0 | 0 | 0 | 20 |
|  | *hsa-miR-372-3p.53.P0.S.11* | 0 | 0 | 0 | 2 |
|  | *hsa-miR-372-3p.53.P0.S.12* | 0 | 0 | 0 | 98 |
|  | *hsa-miR-372-3p.53.P0.S.13* | 0 | 0 | 0 | 2 |
|  | *hsa-miR-372-3p.53.P0.S.14* | 0 | 0 | 0 | 1 |
|  | *hsa-miR-372-3p.53.P1.S.0* | 0 | 0 | 0 | 2 |
|  | *hsa-miR-372-3p.53.P0.S.15* | 0 | 0 | 0 | 221 |
|  | *hsa-miR-372-3p.53.P1.S.1* | 0 | 0 | 0 | 1 |
|  | *hsa-miR-372-3p.53.P1.S.2* | 0 | 0 | 0 | 1 |
|  | *hsa-miR-372-3p.53.P1.S.3* | 0 | 0 | 0 | 4 |
|  | *hsa-miR-372-3p.53.P1.S.4* | 0 | 0 | 0 | 11 |
|  | *hsa-miR-372-3p.53.P0.S.16* | 0 | 0 | 0 | 71 |
|  | *hsa-miR-372-3p.53.P1.S.5* | 0 | 0 | 0 | 6 |
|  | *hsa-miR-372-3p.53.P0.S.17* | 0 | 0 | 0 | 169 |
|  | *hsa-miR-372-3p.53.P1.S.6* | 0 | 0 | 0 | 1 |
|  | *hsa-miR-372-3p.53.P1.S.7* | 0 | 0 | 0 | 7 |
|  | *hsa-miR-372-3p.53.P1.S.8* | 0 | 0 | 0 | 2 |
|  | *hsa-miR-372-3p.53.P1.S.9* | 0 | 0 | 0 | 10 |
|  | *hsa-miR-372-3p.53.P0.S.18* | 0 | 0 | 0 | 75 |
|  | *hsa-miR-372-3p.53.P1.S.10* | 0 | 0 | 0 | 1 |
|  | *hsa-miR-372-3p.53.P1.S.11* | 0 | 0 | 0 | 21 |
|  | *hsa-miR-372-3p.53.P0.S.19* | 0 | 0 | 0 | 144 |
|  | *hsa-miR-372-3p.53.P1.S.12* | 0 | 0 | 0 | 1 |
|  | *hsa-miR-372-3p.53.P1.S.13* | 0 | 0 | 0 | 2 |
|  | *hsa-miR-372-3p.53.P1.S.14* | 0 | 0 | 0 | 1 |
|  | *hsa-miR-372-3p.53.P0.S.20* | 0 | 0 | 0 | 3 |
|  | *hsa-miR-372-3p.53.P0.S.21* | 0 | 0 | 0 | 1 |
|  | *hsa-miR-372-3p.53.P1.S.15* | 0 | 0 | 0 | 1 |
|  | *hsa-miR-372-3p.53.P0.S.22* | 0 | 0 | 0 | 8 |
|  | *hsa-miR-372-3p.53.P0.S.23* | 0 | 0 | 0 | 1 |
|  | *hsa-miR-372-3p.53.P0.S.24* | 0 | 0 | 0 | 3 |
|  | *hsa-miR-372-3p.53.P0.S.25* | 0 | 0 | 0 | 1 |
|  | *hsa-miR-372-3p.5.P0.S.4* | 0 | 0 | 0 | 7 |
|  | *hsa-miR-372-3p.53.P0.S.26* | 0 | 0 | 0 | 1 |
|  | *hsa-miR-372-3p.53.P0.S.27* | 0 | 0 | 0 | 1 |
|  | *hsa-miR-372-3p.53.P0.S.28* | 0 | 0 | 0 | 3 |
|  | *hsa-miR-372-3p.53.P0.S.29* | 0 | 0 | 0 | 11 |
|  | *hsa-miR-372-3p.53.P0.S.30* | 0 | 0 | 0 | 5 |
|  | *hsa-miR-372-3p.53.P0.S.31* | 0 | 0 | 0 | 20 |
|  | *hsa-miR-372-3p.5.P0.S.5* | 0 | 0 | 0 | 12 |
|  | *hsa-miR-372-3p.5.P1.S.0* | 0 | 0 | 0 | 1 |
|  | *hsa-miR-372-3p.5.P1.S.1* | 0 | 0 | 0 | 11 |
|  | *hsa-miR-372-3p.5.P0.S.6* | 0 | 0 | 0 | 49 |
|  | *hsa-miR-372-3p.5.P1.S.2* | 0 | 0 | 0 | 2 |
|  | *hsa-miR-372-3p.5.P0.S.7* | 0 | 0 | 0 | 76 |
|  | *hsa-miR-372-3p.5.P1.S.3* | 0 | 0 | 0 | 1 |
|  | *hsa-miR-372-3p.5.P0.S.8* | 19 | 0 | 3 | 7152 |
|  | *hsa-miR-372-3p.5.P1.S.4* | 0 | 0 | 0 | 2 |
|  | *hsa-miR-372-3p.5.P1.S.5* | 0 | 0 | 0 | 6 |
|  | *hsa-miR-372-3p.5.P1.S.6* | 0 | 0 | 0 | 2 |
|  | *hsa-miR-372-3p.5.P1.S.7* | 0 | 0 | 0 | 2 |
|  | *hsa-miR-372-3p.5.P1.S.8* | 0 | 0 | 0 | 2 |
|  | *hsa-miR-372-3p.5.P1.S.9* | 0 | 0 | 0 | 4 |
|  | *hsa-miR-372-3p.5.P1.S.10* | 0 | 0 | 0 | 1 |
|  | *hsa-miR-372-3p.5.P1.S.11* | 0 | 0 | 0 | 1 |
|  | *hsa-miR-372-3p.5.P1.S.12* | 0 | 0 | 0 | 3 |
|  | *hsa-miR-372-3p.5.P1.S.13* | 0 | 0 | 0 | 4 |
|  | *hsa-miR-372-3p.5.P1.S.14* | 0 | 0 | 0 | 1 |
|  | *hsa-miR-372-3p.5.P1.S.15* | 0 | 0 | 0 | 2 |
|  | *hsa-miR-372-3p.5.P1.S.16* | 0 | 0 | 0 | 9 |
|  | *hsa-miR-372-3p.5.P1.S.17* | 0 | 0 | 0 | 1 |
|  | *hsa-miR-372-3p.5.P1.S.18* | 0 | 0 | 0 | 5 |
|  | *hsa-miR-372-3p.5.P1.S.19* | 0 | 0 | 0 | 3 |
|  | *hsa-miR-372-3p.5.P1.S.20* | 0 | 0 | 0 | 15 |
|  | *hsa-miR-372-3p.5.P1.S.21* | 0 | 0 | 0 | 2 |
|  | *hsa-miR-372-3p.5.P1.S.22* | 0 | 0 | 0 | 3 |
|  | *hsa-miR-372-3p.5.P1.S.23* | 0 | 0 | 0 | 3 |
|  | *hsa-miR-372-3p.5.P1.S.24* | 0 | 0 | 0 | 1 |
|  | *hsa-miR-372-3p.5.P1.S.25* | 0 | 0 | 0 | 2 |
|  | *hsa-miR-372-3p.5.P1.S.26* | 0 | 0 | 0 | 1 |
|  | *hsa-miR-372-3p.5.P1.S.27* | 0 | 0 | 0 | 1 |
|  | *hsa-miR-372-3p.5.P1.S.28* | 0 | 0 | 0 | 4 |
|  | *hsa-miR-372-3p.5.P1.S.29* | 0 | 0 | 0 | 1 |
|  | *hsa-miR-372-3p.5.P1.S.30* | 0 | 0 | 0 | 1 |
|  | *hsa-miR-372-3p.5.P1.S.31* | 0 | 0 | 0 | 2 |
|  | *hsa-miR-372-3p.5.P1.S.32* | 0 | 0 | 0 | 1 |
|  | *hsa-miR-372-3p.5.P1.S.33* | 0 | 0 | 0 | 1 |
|  | *hsa-miR-372-3p.5.P1.S.34* | 0 | 0 | 0 | 128 |
|  | *hsa-miR-372-3p.5.P1.S.35* | 0 | 0 | 0 | 13 |
|  | *hsa-miR-372-3p.5.P1.S.36* | 0 | 0 | 0 | 19 |
|  | *hsa-miR-372-3p.53.P0.S.32* | 0 | 0 | 0 | 2 |
|  | *hsa-miR-372-3p.53.P0.S.33* | 0 | 0 | 0 | 1 |
|  | *hsa-miR-372-3p.5.P0.S.9* | 0 | 0 | 0 | 2 |
|  | *hsa-miR-372-3p.0.P1.S.0* | 0 | 0 | 0 | 3 |
|  | *hsa-miR-372-3p.0.P1.S.1* | 19 | 0 | 0 | 10 |
|  | *hsa-miR-372-3p.0.P1.S.2* | 0 | 0 | 0 | 4 |
|  | *hsa-miR-372-3p.0.P1.S.3* | 0 | 0 | 0 | 1 |
|  | *hsa-miR-372-3p.0.P1.S.4* | 0 | 0 | 0 | 3 |
|  | *hsa-miR-372-3p.0.P1.S.5* | 0 | 0 | 0 | 2 |
|  | *hsa-miR-372-3p.0.P1.S.6* | 0 | 0 | 0 | 1 |
|  | *hsa-miR-372-3p.0.P1.S.7* | 0 | 0 | 0 | 9 |
|  | *hsa-miR-372-3p.0.P1.S.8* | 0 | 0 | 0 | 11 |
|  | *hsa-miR-372-3p.0.P1.S.9* | 0 | 0 | 0 | 1 |
|  | *hsa-miR-372-3p.0.P1.S.10* | 0 | 0 | 0 | 4 |
|  | *hsa-miR-372-3p.0.P1.S.11* | 0 | 0 | 0 | 16 |
|  | *hsa-miR-372-3p.0.P1.S.12* | 0 | 0 | 0 | 6 |
|  | *hsa-miR-372-3p.0.P1.S.13* | 0 | 0 | 0 | 1 |
|  | *hsa-miR-372-3p.0.P1.S.14* | 0 | 0 | 0 | 4 |
|  | *hsa-miR-372-3p.0.P1.S.15* | 0 | 0 | 0 | 10 |
|  | *hsa-miR-372-3p.0.P1.S.16* | 0 | 0 | 0 | 10 |
|  | *hsa-miR-372-3p.0.P1.S.17* | 0 | 0 | 0 | 3 |
|  | *hsa-miR-372-3p.0.P1.S.18* | 0 | 0 | 0 | 14 |
|  | *hsa-miR-372-3p.0.P1.S.19* | 0 | 0 | 0 | 7 |
|  | *hsa-miR-372-3p.0.P1.S.20* | 0 | 0 | 0 | 3 |
|  | *hsa-miR-372-3p.0.P1.S.21* | 0 | 0 | 0 | 20 |
|  | *hsa-miR-372-3p.0.P1.S.22* | 0 | 0 | 0 | 2 |
|  | *hsa-miR-372-3p.0.P1.S.23* | 0 | 0 | 0 | 4 |
|  | *hsa-miR-372-3p.0.P1.S.24* | 0 | 0 | 0 | 15 |
|  | *hsa-miR-372-3p.0.P1.S.25* | 0 | 0 | 0 | 1 |
|  | *hsa-miR-372-3p.0.P1.S.26* | 0 | 0 | 0 | 7 |
|  | *hsa-miR-372-3p.0.P1.S.27* | 0 | 0 | 0 | 4 |
|  | *hsa-miR-372-3p.0.P1.S.28* | 0 | 0 | 0 | 6 |
|  | *hsa-miR-372-3p.0.P1.S.29* | 0 | 0 | 0 | 7 |
|  | *hsa-miR-372-3p.0.P1.S.30* | 0 | 0 | 0 | 2 |
|  | *hsa-miR-372-3p.0.P1.S.31* | 0 | 0 | 0 | 7 |
|  | *hsa-miR-372-3p.0.P1.S.32* | 0 | 0 | 0 | 4 |
|  | *hsa-miR-372-3p.0.P1.S.33* | 0 | 0 | 0 | 2 |
|  | *hsa-miR-372-3p.0.P1.S.34* | 0 | 0 | 0 | 1 |
|  | *hsa-miR-372-3p.0.P1.S.35* | 0 | 0 | 0 | 2 |
|  | *hsa-miR-372-3p.0.P1.S.36* | 0 | 0 | 0 | 13 |
|  | *hsa-miR-372-3p.0.P1.S.37* | 0 | 0 | 0 | 4 |
|  | *hsa-miR-372-3p.0.P1.S.38* | 0 | 0 | 0 | 8 |
|  | *hsa-miR-372-3p.0.P1.S.39* | 0 | 0 | 0 | 2 |
|  | *hsa-miR-372-3p.0.P1.S.40* | 0 | 0 | 0 | 11 |
|  | *hsa-miR-372-3p.0.P1.S.41* | 0 | 0 | 0 | 6 |
|  | *hsa-miR-372-3p.0.P1.S.42* | 0 | 0 | 0 | 634 |
|  | *hsa-miR-372-3p.0.P1.S.43* | 0 | 0 | 0 | 24 |
|  | *hsa-miR-372-3p.0.P1.S.44* | 0 | 0 | 0 | 61 |
| ***hsa-miR-371a-3p*** | *hsa-miR-371a-3p.0.P1.S.47* | 0 | 0 | 0 | 1266 |
|  | *hsa-miR-371a-3p.5.P0.S.9* | 372 | 13 | 257 | 56720 |
|  | *hsa-miR-371a-3p.5.P0.S.7* | 284 | 25 | 120 | 34824 |
|  | *hsa-miR-371a-3p.3.P0.S.9* | 0 | 0 | 0 | 2139 |
|  | *hsa-miR-371a-3p.3.P0.S.6* | 19 | 0 | 0 | 1467 |
|  | *hsa-miR-371a-3p.3.P0.S.0* | 0 | 0 | 0 | 6 |
|  | *hsa-miR-371a-3p.3.P0.S.1* | 19 | 0 | 0 | 4 |
|  | *hsa-miR-371a-3p.3.P0.S.2* | 0 | 0 | 0 | 6 |
|  | *hsa-miR-371a-3p.3.P0.S.3* | 0 | 0 | 0 | 2 |
|  | *hsa-miR-371a-3p.3.P0.S.4* | 0 | 2 | 0 | 8 |
|  | *hsa-miR-371a-3p.3.P1.S.0* | 0 | 0 | 0 | 11 |
|  | *hsa-miR-371a-3p.3.P0.S.5* | 0 | 0 | 0 | 111 |
|  | *hsa-miR-371a-3p.3.P1.S.1* | 0 | 0 | 0 | 1 |
|  | *hsa-miR-371a-3p.3.P1.S.2* | 0 | 0 | 0 | 1 |
|  | *hsa-miR-371a-3p.3.P1.S.3* | 0 | 0 | 0 | 1 |
|  | *hsa-miR-371a-3p.3.P1.S.4* | 0 | 0 | 0 | 2 |
|  | *hsa-miR-371a-3p.3.P0.S.6* | 19 | 0 | 0 | 1467 |
|  | *hsa-miR-371a-3p.3.P1.S.5* | 0 | 0 | 0 | 1 |
|  | *hsa-miR-371a-3p.3.P1.S.6* | 0 | 0 | 0 | 1 |
|  | *hsa-miR-371a-3p.3.P1.S.7* | 0 | 0 | 0 | 1 |
|  | *hsa-miR-371a-3p.3.P0.S.5* | 0 | 0 | 0 | 111 |
|  | *hsa-miR-371a-3p.3.P0.S.6* | 19 | 0 | 0 | 1467 |
|  | *hsa-miR-371a-3p.3.P0.S.7* | 0 | 0 | 0 | 1 |
|  | *hsa-miR-371a-3p.3.P0.S.8* | 0 | 0 | 0 | 32 |
|  | *hsa-miR-371a-3p.3.P0.S.9* | 0 | 0 | 0 | 2139 |
|  | *hsa-miR-371a-3p.3.P1.S.1* | 0 | 0 | 0 | 1 |
|  | *hsa-miR-371a-3p.3.P0.S.6* | 19 | 0 | 0 | 1467 |
|  | *hsa-miR-371a-3p.3.P1.S.5* | 0 | 0 | 0 | 1 |
|  | *hsa-miR-371a-3p.3.P1.S.6* | 0 | 0 | 0 | 1 |
|  | *hsa-miR-371a-3p.3.P1.S.7* | 0 | 0 | 0 | 1 |
|  | *hsa-miR-371a-3p.3.P1.S.8* | 0 | 0 | 0 | 1 |
|  | *hsa-miR-371a-3p.3.P0.S.7* | 0 | 0 | 0 | 1 |
|  | *hsa-miR-371a-3p.3.P1.S.8* | 0 | 0 | 0 | 1 |
|  | *hsa-miR-371a-3p.3.P1.S.9* | 0 | 0 | 0 | 5 |
|  | *hsa-miR-371a-3p.3.P1.S.10* | 0 | 0 | 0 | 1 |
|  | *hsa-miR-371a-3p.3.P0.S.10* | 0 | 0 | 0 | 25 |
|  | *hsa-miR-371a-3p.3.P1.S.2* | 0 | 0 | 0 | 1 |
|  | *hsa-miR-371a-3p.3.P0.S.7* | 0 | 0 | 0 | 1 |
|  | *hsa-miR-371a-3p.3.P1.S.9* | 0 | 0 | 0 | 5 |
|  | *hsa-miR-371a-3p.3.P1.S.10* | 0 | 0 | 0 | 1 |
|  | *hsa-miR-371a-3p.3.P1.S.11* | 0 | 0 | 0 | 1 |
|  | *hsa-miR-371a-3p.3.P1.S.12* | 0 | 0 | 0 | 1 |
|  | *hsa-miR-371a-3p.3.P0.S.8* | 0 | 0 | 0 | 32 |
|  | *hsa-miR-371a-3p.3.P1.S.11* | 0 | 0 | 0 | 1 |
|  | *hsa-miR-371a-3p.3.P1.S.12* | 0 | 0 | 0 | 1 |
|  | *hsa-miR-371a-3p.3.P1.S.13* | 0 | 0 | 0 | 1 |
|  | *hsa-miR-371a-3p.3.P1.S.3* | 0 | 0 | 0 | 1 |
|  | *hsa-miR-371a-3p.3.P1.S.14* | 0 | 0 | 0 | 2 |
|  | *hsa-miR-371a-3p.3.P1.S.15* | 0 | 0 | 0 | 1 |
|  | *hsa-miR-371a-3p.3.P1.S.16* | 0 | 0 | 0 | 8 |
|  | *hsa-miR-371a-3p.3.P0.S.9* | 0 | 0 | 0 | 2139 |
|  | *hsa-miR-371a-3p.3.P1.S.14* | 0 | 0 | 0 | 2 |
|  | *hsa-miR-371a-3p.3.P1.S.15* | 0 | 0 | 0 | 1 |
|  | *hsa-miR-371a-3p.3.P1.S.16* | 0 | 0 | 0 | 8 |
|  | *hsa-miR-371a-3p.3.P1.S.4* | 0 | 0 | 0 | 2 |
|  | *hsa-miR-371a-3p.3.P1.S.17* | 0 | 0 | 0 | 2 |
|  | *hsa-miR-371a-3p.3.P1.S.18* | 0 | 0 | 0 | 1 |
|  | *hsa-miR-371a-3p.3.P1.S.19* | 0 | 0 | 0 | 1 |
|  | *hsa-miR-371a-3p.3.P1.S.20* | 0 | 0 | 0 | 1 |
|  | *hsa-miR-371a-3p.3.P0.S.10* | 0 | 0 | 0 | 25 |
|  | *hsa-miR-371a-3p.3.P1.S.17* | 0 | 0 | 0 | 2 |
|  | *hsa-miR-371a-3p.3.P1.S.18* | 0 | 0 | 0 | 1 |
|  | *hsa-miR-371a-3p.3.P1.S.19* | 0 | 0 | 0 | 1 |
|  | *hsa-miR-371a-3p.3.P0.S.25* | 0 | 0 | 0 | 7 |
|  | *hsa-miR-371a-3p.3.P0.S.26* | 0 | 0 | 0 | 34 |
|  | *hsa-miR-371a-3p.3.P0.S.27* | 0 | 0 | 0 | 850 |
|  | *hsa-miR-371a-3p.3.P0.S.28* | 0 | 0 | 0 | 68 |
|  | *hsa-miR-371a-3p.3.P0.S.29* | 0 | 0 | 0 | 1 |
|  | *hsa-miR-371a-3p.3.P1.S.5* | 0 | 0 | 0 | 1 |
|  | *hsa-miR-371a-3p.3.P0.S.10* | 0 | 0 | 0 | 25 |
|  | *hsa-miR-371a-3p.3.P1.S.21* | 0 | 0 | 0 | 1 |
|  | *hsa-miR-371a-3p.3.P1.S.22* | 0 | 0 | 0 | 1 |
|  | *hsa-miR-371a-3p.3.P1.S.23* | 0 | 0 | 0 | 1 |
|  | *hsa-miR-371a-3p.3.P1.S.24* | 0 | 0 | 0 | 1 |
|  | *hsa-miR-371a-3p.3.P1.S.20* | 0 | 0 | 0 | 1 |
|  | *hsa-miR-371a-3p.3.P1.S.21* | 0 | 0 | 0 | 1 |
|  | *hsa-miR-371a-3p.3.P1.S.22* | 0 | 0 | 0 | 1 |
|  | *hsa-miR-371a-3p.3.P1.S.6* | 0 | 0 | 0 | 1 |
|  | *hsa-miR-371a-3p.3.P1.S.25* | 0 | 0 | 0 | 3 |
|  | *hsa-miR-371a-3p.3.P1.S.26* | 0 | 0 | 0 | 2 |
|  | *hsa-miR-371a-3p.3.P1.S.27* | 0 | 0 | 0 | 1 |
|  | *hsa-miR-371a-3p.3.P1.S.28* | 0 | 0 | 0 | 3 |
|  | *hsa-miR-371a-3p.3.P1.S.23* | 0 | 0 | 0 | 1 |
|  | *hsa-miR-371a-3p.3.P1.S.24* | 0 | 0 | 0 | 1 |
|  | *hsa-miR-371a-3p.3.P1.S.25* | 0 | 0 | 0 | 3 |
|  | *hsa-miR-371a-3p.3.P0.S.35* | 0 | 0 | 0 | 10 |
|  | *hsa-miR-371a-3p.3.P0.S.36* | 0 | 0 | 0 | 0 |
|  | *hsa-miR-371a-3p.3.P0.S.37* | 0 | 0 | 0 | 1 |
|  | *hsa-miR-371a-3p.3.P0.S.38* | 0 | 0 | 0 | 18 |
|  | *hsa-miR-371a-3p.3.P0.S.39* | 0 | 0 | 0 | 124 |
|  | *hsa-miR-371a-3p.3.P1.S.7* | 0 | 0 | 0 | 1 |
|  | *hsa-miR-371a-3p.3.P1.S.29* | 0 | 0 | 0 | 1 |
|  | *hsa-miR-371a-3p.3.P1.S.30* | 0 | 0 | 0 | 3 |
|  | *hsa-miR-371a-3p.3.P1.S.31* | 0 | 0 | 0 | 1 |
|  | *hsa-miR-371a-3p.3.P1.S.32* | 0 | 0 | 0 | 3 |
|  | *hsa-miR-371a-3p.3.P1.S.26* | 0 | 0 | 0 | 2 |
|  | *hsa-miR-371a-3p.3.P1.S.27* | 0 | 0 | 0 | 1 |
|  | *hsa-miR-371a-3p.3.P1.S.28* | 0 | 0 | 0 | 3 |
|  | *hsa-miR-371a-3p.3.P1.S.8* | 0 | 0 | 0 | 1 |
|  | *hsa-miR-371a-3p.3.P1.S.33* | 0 | 0 | 0 | 1 |
|  | *hsa-miR-371a-3p.3.P1.S.34* | 0 | 0 | 0 | 4 |
|  | *hsa-miR-371a-3p.3.P1.S.35* | 0 | 0 | 0 | 1 |
|  | *hsa-miR-371a-3p.3.P1.S.36* | 0 | 0 | 0 | 25 |
|  | *hsa-miR-371a-3p.3.P1.S.29* | 0 | 0 | 0 | 1 |
|  | *hsa-miR-371a-3p.3.P1.S.30* | 0 | 0 | 0 | 3 |
|  | *hsa-miR-371a-3p.3.P1.S.31* | 0 | 0 | 0 | 1 |
| ***hsa-miR-34a-5p*** | *hsa-miR-34a-5p.3.P0.S.0* | 0 | 2 | 3 | 11 |
|  | *hsa-miR-34a-5p.3.P0.S.1* | 20 | 24 | 18 | 50 |
|  | *hsa-miR-34a-5p.3.P0.S.2* | 0 | 36 | 32 | 26 |
|  | *hsa-miR-34a-5p.3.P0.S.3* | 0 | 4 | 18 | 14 |
|  | *hsa-miR-34a-5p.3.P0.S.4* | 76 | 75 | 56 | 124 |
|  | *hsa-miR-34a-5p.3.P0.S.5* | 0 | 22 | 13 | 43 |
|  | *hsa-miR-34a-5p.3.P0.S.6* | 0 | 2 | 0 | 12 |
|  | *hsa-miR-34a-5p.3.P0.S.7* | 0 | 0 | 0 | 2 |
|  | *hsa-miR-34a-5p.3.P0.S.8* | 120 | 134 | 136 | 523 |
|  | *hsa-miR-34a-5p.3.P0.S.9* | 10 | 4 | 5 | 26 |
|  | *hsa-miR-34a-5p.3.P0.S.10* | 42 | 99 | 453 | 465 |
|  | *hsa-miR-34a-5p.3.P1.S.0* | 0 | 0 | 0 | 1 |
|  | *hsa-miR-34a-5p.3.P1.S.1* | 0 | 2 | 0 | 0 |
|  | *hsa-miR-34a-5p.3.P1.S.2* | 0 | 0 | 0 | 1 |
|  | *hsa-miR-34a-5p.3.P1.S.3* | 0 | 0 | 5 | 0 |
|  | *hsa-miR-34a-5p.3.P1.S.4* | 0 | 0 | 5 | 1 |
|  | *hsa-miR-34a-5p.3.P1.S.5* | 0 | 5 | 0 | 1 |
|  | *hsa-miR-34a-5p.3.P1.S.6* | 0 | 0 | 0 | 1 |
|  | *hsa-miR-34a-5p.3.P1.S.7* | 0 | 0 | 0 | 1 |
|  | *hsa-miR-34a-5p.3.P1.S.8* | 0 | 0 | 0 | 2 |
|  | *hsa-miR-34a-5p.3.P1.S.9* | 0 | 0 | 5 | 1 |
|  | *hsa-miR-34a-5p.3.P1.S.10* | 0 | 0 | 0 | 2 |
|  | *hsa-miR-34a-5p.3.P1.S.11* | 0 | 0 | 0 | 1 |
|  | *hsa-miR-34a-5p.3.P0.S.11* | 39 | 24 | 66 | 83 |
|  | *hsa-miR-34a-5p.3.P1.S.12* | 0 | 0 | 0 | 1 |
|  | *hsa-miR-34a-5p.3.P1.S.13* | 0 | 0 | 5 | 1 |
|  | *hsa-miR-34a-5p.3.P1.S.14* | 0 | 0 | 0 | 1 |
|  | *hsa-miR-34a-5p.3.P0.S.12* | 0 | 0 | 0 | 1 |
|  | *hsa-miR-34a-5p.3.P0.S.13* | 65 | 22 | 196 | 135 |
|  | *hsa-miR-34a-5p.3.P0.S.14* | 10 | 4 | 0 | 9 |
|  | *hsa-miR-34a-5p.3.P1.S.15* | 0 | 0 | 5 | 1 |
|  | *hsa-miR-34a-5p.3.P1.S.16* | 0 | 0 | 0 | 1 |
|  | *hsa-miR-34a-5p.3.P1.S.17* | 0 | 0 | 8 | 1 |
|  | *hsa-miR-34a-5p.3.P1.S.18* | 0 | 0 | 3 | 0 |
|  | *hsa-miR-34a-5p.3.P1.S.19* | 0 | 0 | 0 | 1 |
|  | *hsa-miR-34a-5p.3.P1.S.20* | 3 | 0 | 0 | 0 |
|  | *hsa-miR-34a-5p.3.P1.S.21* | 0 | 0 | 8 | 0 |
|  | *hsa-miR-34a-5p.3.P1.S.22* | 0 | 0 | 5 | 1 |
|  | *hsa-miR-34a-5p.3.P0.S.15* | 319 | 384 | 2130 | 2126 |
|  | *hsa-miR-34a-5p.3.P0.S.16* | 1212 | 1901 | 2962 | 6418 |
|  | *hsa-miR-34a-5p.3.P1.S.23* | 0 | 0 | 3 | 0 |
|  | *hsa-miR-34a-5p.3.P1.S.24* | 0 | 0 | 7 | 1 |
|  | *hsa-miR-34a-5p.3.P1.S.25* | 0 | 0 | 0 | 1 |
|  | *hsa-miR-34a-5p.3.P1.S.26* | 0 | 2 | 0 | 3 |
|  | *hsa-miR-34a-5p.3.P1.S.27* | 0 | 0 | 0 | 1 |
|  | *hsa-miR-34a-5p.3.P1.S.28* | 0 | 0 | 5 | 0 |
|  | *hsa-miR-34a-5p.3.P1.S.29* | 0 | 0 | 0 | 4 |
|  | *hsa-miR-34a-5p.3.P1.S.30* | 0 | 0 | 0 | 1 |
|  | *hsa-miR-34a-5p.3.P1.S.31* | 0 | 0 | 0 | 5 |
|  | *hsa-miR-34a-5p.3.P1.S.32* | 0 | 0 | 0 | 2 |
|  | *hsa-miR-34a-5p.3.P1.S.33* | 0 | 0 | 0 | 4 |
|  | *hsa-miR-34a-5p.3.P1.S.34* | 0 | 0 | 3 | 1 |
|  | *hsa-miR-34a-5p.3.P1.S.35* | 3 | 0 | 0 | 6 |
|  | *hsa-miR-34a-5p.3.P1.S.36* | 3 | 0 | 0 | 3 |
|  | *hsa-miR-34a-5p.3.P1.S.37* | 0 | 0 | 0 | 3 |
|  | *hsa-miR-34a-5p.3.P1.S.38* | 3 | 0 | 0 | 5 |
|  | *hsa-miR-34a-5p.3.P1.S.39* | 0 | 0 | 0 | 1 |
|  | *hsa-miR-34a-5p.3.P1.S.40* | 0 | 0 | 0 | 1 |
|  | *hsa-miR-34a-5p.3.P1.S.41* | 7 | 0 | 3 | 4 |
|  | *hsa-miR-34a-5p.3.P1.S.42* | 0 | 0 | 5 | 1 |
|  | *hsa-miR-34a-5p.3.P1.S.43* | 0 | 0 | 0 | 2 |
|  | *hsa-miR-34a-5p.3.P1.S.44* | 0 | 0 | 3 | 1 |
|  | *hsa-miR-34a-5p.3.P1.S.45* | 0 | 0 | 0 | 2 |
|  | *hsa-miR-34a-5p.3.P1.S.46* | 0 | 0 | 0 | 2 |
|  | *hsa-miR-34a-5p.3.P1.S.47* | 0 | 0 | 3 | 1 |
|  | *hsa-miR-34a-5p.3.P1.S.48* | 0 | 4 | 5 | 3 |
|  | *hsa-miR-34a-5p.3.P1.S.49* | 0 | 2 | 15 | 3 |
|  | *hsa-miR-34a-5p.3.P1.S.50* | 19 | 0 | 5 | 2 |
|  | *hsa-miR-34a-5p.3.P1.S.51* | 0 | 0 | 10 | 2 |
|  | *hsa-miR-34a-5p.3.P1.S.52* | 0 | 0 | 0 | 5 |
|  | *hsa-miR-34a-5p.3.P1.S.53* | 0 | 5 | 0 | 1 |
|  | *hsa-miR-34a-5p.3.P1.S.54* | 0 | 0 | 0 | 1 |
|  | *hsa-miR-34a-5p.3.P1.S.55* | 0 | 0 | 8 | 5 |
|  | *hsa-miR-34a-5p.3.P1.S.56* | 0 | 0 | 0 | 1 |
|  | *hsa-miR-34a-5p.3.P1.S.57* | 0 | 2 | 0 | 4 |
|  | *hsa-miR-34a-5p.3.P1.S.58* | 0 | 0 | 0 | 2 |
|  | *hsa-miR-34a-5p.3.P1.S.59* | 7 | 0 | 0 | 3 |
|  | *hsa-miR-34a-5p.3.P1.S.60* | 19 | 0 | 7 | 2 |
|  | *hsa-miR-34a-5p.3.P1.S.61* | 0 | 0 | 0 | 2 |
|  | *hsa-miR-34a-5p.3.P1.S.62* | 19 | 0 | 0 | 1 |
|  | *hsa-miR-34a-5p.3.P1.S.63* | 0 | 0 | 0 | 5 |
|  | *hsa-miR-34a-5p.3.P1.S.64* | 0 | 0 | 3 | 0 |
|  | *hsa-miR-34a-5p.3.P1.S.65* | 0 | 0 | 3 | 1 |
|  | *hsa-miR-34a-5p.3.P1.S.66* | 0 | 0 | 0 | 2 |
|  | *hsa-miR-34a-5p.3.P1.S.67* | 0 | 0 | 5 | 0 |
|  | *hsa-miR-34a-5p.3.P1.S.68* | 0 | 0 | 0 | 1 |
|  | *hsa-miR-34a-5p.3.P1.S.69* | 0 | 0 | 7 | 2 |
|  | *hsa-miR-34a-5p.3.P1.S.70* | 0 | 9 | 3 | 8 |
|  | *hsa-miR-34a-5p.3.P1.S.71* | 0 | 0 | 0 | 2 |
|  | *hsa-miR-34a-5p.3.P1.S.72* | 0 | 5 | 0 | 1 |
|  | *hsa-miR-34a-5p.3.P1.S.73* | 0 | 2 | 21 | 30 |
|  | *hsa-miR-34a-5p.3.P1.S.74* | 0 | 0 | 0 | 1 |
|  | *hsa-miR-34a-5p.3.P1.S.75* | 0 | 2 | 0 | 14 |
|  | *hsa-miR-34a-5p.3.P0.S.17* | 10 | 13 | 54 | 81 |
|  | *hsa-miR-34a-5p.3.P1.S.76* | 0 | 0 | 0 | 2 |
|  | *hsa-miR-34a-5p.3.P1.S.77* | 0 | 0 | 0 | 1 |
|  | *hsa-miR-34a-5p.3.P1.S.78* | 0 | 0 | 0 | 2 |
|  | *hsa-miR-34a-5p.3.P1.S.79* | 0 | 0 | 0 | 1 |
|  | *hsa-miR-34a-5p.3.P1.S.80* | 0 | 0 | 0 | 3 |
|  | *hsa-miR-34a-5p.3.P1.S.81* | 0 | 0 | 0 | 1 |
|  | *hsa-miR-34a-5p.3.P1.S.82* | 0 | 0 | 0 | 2 |
|  | *hsa-miR-34a-5p.3.P1.S.83* | 0 | 0 | 0 | 1 |
|  | *hsa-miR-34a-5p.3.P1.S.84* | 0 | 0 | 5 | 0 |
|  | *hsa-miR-34a-5p.3.P1.S.85* | 0 | 0 | 0 | 1 |
|  | *hsa-miR-34a-5p.3.P1.S.86* | 0 | 5 | 0 | 10 |
|  | *hsa-miR-34a-5p.3.P1.S.87* | 0 | 2 | 0 | 2 |
|  | *hsa-miR-34a-5p.3.P1.S.88* | 0 | 0 | 8 | 16 |
|  | *hsa-miR-34a-5p.3.P1.S.89* | 3 | 4 | 13 | 6 |
|  | *hsa-miR-34a-5p.53.P0.S.0* | 0 | 0 | 0 | 4 |
|  | *hsa-miR-34a-5p.5.P0.S.0* | 0 | 0 | 0 | 6 |
|  | *hsa-miR-34a-5p.53.P0.S.1* | 0 | 0 | 0 | 2 |
|  | *hsa-miR-34a-5p.5.P0.S.1* | 0 | 0 | 0 | 2 |
|  | *hsa-miR-34a-5p.5.P0.S.2* | 0 | 2 | 0 | 0 |
|  | *hsa-miR-34a-5p.53.P0.S.2* | 0 | 5 | 0 | 1 |
|  | *hsa-miR-34a-5p.53.P0.S.3* | 0 | 5 | 0 | 1 |
|  | *hsa-miR-34a-5p.53.P0.S.4* | 0 | 0 | 0 | 1 |
|  | *hsa-miR-34a-5p.53.P0.S.5* | 0 | 0 | 0 | 1 |
|  | *hsa-miR-34a-5p.53.P0.S.6* | 0 | 0 | 0 | 1 |
|  | *hsa-miR-34a-5p.53.P0.S.7* | 0 | 2 | 3 | 6 |
|  | *hsa-miR-34a-5p.53.P0.S.8* | 0 | 0 | 7 | 0 |
|  | *hsa-miR-34a-5p.53.P0.S.9* | 0 | 13 | 5 | 26 |
|  | *hsa-miR-34a-5p.53.P0.S.10* | 0 | 0 | 10 | 5 |
|  | *hsa-miR-34a-5p.53.P0.S.11* | 7 | 0 | 0 | 2 |
|  | *hsa-miR-34a-5p.53.P1.S.0* | 0 | 2 | 0 | 0 |
|  | *hsa-miR-34a-5p.53.P1.S.1* | 0 | 0 | 0 | 1 |
|  | *hsa-miR-34a-5p.53.P0.S.12* | 0 | 2 | 0 | 2 |
|  | *hsa-miR-34a-5p.53.P0.S.13* | 0 | 0 | 15 | 0 |
|  | *hsa-miR-34a-5p.53.P0.S.14* | 19 | 2 | 10 | 18 |
|  | *hsa-miR-34a-5p.53.P0.S.15* | 66 | 131 | 280 | 316 |
|  | *hsa-miR-34a-5p.53.P0.S.16* | 20 | 0 | 8 | 17 |
|  | *hsa-miR-34a-5p.53.P0.S.17* | 0 | 0 | 0 | 6 |
|  | *hsa-miR-34a-5p.53.P1.S.2* | 0 | 0 | 0 | 1 |
|  | *hsa-miR-34a-5p.53.P1.S.3* | 0 | 0 | 0 | 1 |
|  | *hsa-miR-34a-5p.53.P1.S.4* | 0 | 2 | 0 | 0 |
|  | *hsa-miR-34a-5p.53.P1.S.5* | 0 | 0 | 0 | 1 |
|  | *hsa-miR-34a-5p.53.P1.S.6* | 0 | 0 | 3 | 0 |
|  | *hsa-miR-34a-5p.53.P1.S.7* | 0 | 0 | 3 | 0 |
|  | *hsa-miR-34a-5p.53.P1.S.8* | 0 | 0 | 0 | 1 |
|  | *hsa-miR-34a-5p.53.P1.S.9* | 0 | 0 | 3 | 0 |
|  | *hsa-miR-34a-5p.53.P0.S.18* | 0 | 0 | 0 | 2 |
|  | *hsa-miR-34a-5p.53.P0.S.19* | 0 | 0 | 0 | 6 |
|  | *hsa-miR-34a-5p.53.P0.S.20* | 0 | 0 | 0 | 3 |
|  | *hsa-miR-34a-5p.53.P0.S.21* | 0 | 0 | 0 | 4 |
|  | *hsa-miR-34a-5p.53.P1.S.10* | 0 | 0 | 0 | 1 |
|  | *hsa-miR-34a-5p.5.P0.S.3* | 0 | 0 | 3 | 2 |
|  | *hsa-miR-34a-5p.5.P0.S.4* | 0 | 0 | 13 | 1 |
|  | *hsa-miR-34a-5p.5.P1.S.0* | 0 | 0 | 5 | 0 |
|  | *hsa-miR-34a-5p.53.P1.S.11* | 0 | 0 | 0 | 3 |
|  | *hsa-miR-34a-5p.5.P0.S.5* | 20 | 106 | 459 | 339 |
|  | *hsa-miR-34a-5p.5.P0.S.6* | 0 | 4 | 0 | 8 |
|  | *hsa-miR-34a-5p.5.P1.S.1* | 0 | 0 | 3 | 0 |
|  | *hsa-miR-34a-5p.5.P1.S.2* | 0 | 0 | 0 | 2 |
|  | *hsa-miR-34a-5p.5.P1.S.3* | 0 | 0 | 0 | 1 |
|  | *hsa-miR-34a-5p.5.P1.S.4* | 0 | 0 | 5 | 1 |
|  | *hsa-miR-34a-5p.5.P1.S.5* | 0 | 0 | 0 | 1 |
|  | *hsa-miR-34a-5p.5.P1.S.6* | 0 | 0 | 0 | 1 |
|  | *hsa-miR-34a-5p.5.P1.S.7* | 0 | 0 | 0 | 1 |
|  | *hsa-miR-34a-5p.5.P1.S.8* | 0 | 0 | 0 | 1 |
|  | *hsa-miR-34a-5p.5.P1.S.9* | 0 | 0 | 0 | 2 |
|  | *hsa-miR-34a-5p.5.P1.S.10* | 0 | 9 | 0 | 0 |
|  | *hsa-miR-34a-5p.53.P0.S.22* | 0 | 0 | 0 | 7 |
|  | *hsa-miR-34a-5p.53.P1.S.12* | 0 | 0 | 0 | 1 |
|  | *hsa-miR-34a-5p.53.P0.S.23* | 0 | 0 | 5 | 0 |
|  | *hsa-miR-34a-5p.53.P0.S.24* | 10 | 2 | 8 | 12 |
|  | *hsa-miR-34a-5p.53.P1.S.13* | 0 | 0 | 3 | 0 |
|  | *hsa-miR-34a-5p.53.P1.S.14* | 0 | 0 | 3 | 0 |
|  | *hsa-miR-34a-5p.5.P0.S.7* | 0 | 9 | 5 | 16 |
|  | *hsa-miR-34a-5p.5.P1.S.11* | 0 | 0 | 0 | 2 |
|  | *hsa-miR-34a-5p.0.P1.S.0* | 7 | 4 | 22 | 20 |
|  | *hsa-miR-34a-5p.0.P1.S.1* | 3 | 0 | 13 | 4 |
|  | *hsa-miR-34a-5p.0.P1.S.2* | 0 | 0 | 0 | 1 |
|  | *hsa-miR-34a-5p.0.P1.S.3* | 0 | 4 | 7 | 10 |
|  | *hsa-miR-34a-5p.0.P1.S.4* | 0 | 0 | 0 | 4 |
|  | *hsa-miR-34a-5p.0.P1.S.5* | 0 | 0 | 0 | 1 |
|  | *hsa-miR-34a-5p.0.P1.S.6* | 0 | 0 | 0 | 2 |
|  | *hsa-miR-34a-5p.0.P1.S.7* | 0 | 0 | 3 | 4 |
|  | *hsa-miR-34a-5p.0.P1.S.8* | 0 | 0 | 0 | 2 |
|  | *hsa-miR-34a-5p.0.P1.S.9* | 0 | 4 | 13 | 14 |
|  | *hsa-miR-34a-5p.0.P1.S.10* | 0 | 2 | 3 | 5 |
|  | *hsa-miR-34a-5p.0.P1.S.11* | 0 | 0 | 5 | 4 |
|  | *hsa-miR-34a-5p.0.P1.S.12* | 39 | 4 | 12 | 15 |
|  | *hsa-miR-34a-5p.0.P1.S.13* | 0 | 0 | 0 | 2 |
|  | *hsa-miR-34a-5p.0.P1.S.14* | 0 | 9 | 3 | 7 |
|  | *hsa-miR-34a-5p.0.P1.S.15* | 0 | 0 | 0 | 3 |
|  | *hsa-miR-34a-5p.0.P1.S.16* | 3 | 0 | 0 | 8 |
|  | *hsa-miR-34a-5p.0.P1.S.17* | 0 | 0 | 0 | 2 |
|  | *hsa-miR-34a-5p.0.P1.S.18* | 3 | 2 | 31 | 26 |
|  | *hsa-miR-34a-5p.0.P1.S.19* | 0 | 5 | 8 | 10 |
|  | *hsa-miR-34a-5p.0.P1.S.20* | 0 | 7 | 14 | 20 |
|  | *hsa-miR-34a-5p.0.P1.S.21* | 0 | 0 | 8 | 0 |
|  | *hsa-miR-34a-5p.0.P1.S.22* | 0 | 5 | 7 | 4 |
|  | *hsa-miR-34a-5p.0.P1.S.23* | 0 | 5 | 17 | 6 |
|  | *hsa-miR-34a-5p.0.P1.S.24* | 0 | 0 | 10 | 6 |
|  | *hsa-miR-34a-5p.0.P1.S.25* | 19 | 2 | 74 | 36 |
|  | *hsa-miR-34a-5p.0.P1.S.26* | 42 | 0 | 24 | 21 |
|  | *hsa-miR-34a-5p.0.P1.S.27* | 0 | 2 | 75 | 27 |
|  | *hsa-miR-34a-5p.0.P1.S.28* | 0 | 2 | 15 | 8 |
|  | *hsa-miR-34a-5p.0.P1.S.29* | 0 | 0 | 5 | 4 |
|  | *hsa-miR-34a-5p.0.P1.S.30* | 39 | 0 | 5 | 3 |
|  | *hsa-miR-34a-5p.0.P1.S.31* | 3 | 0 | 17 | 17 |
|  | *hsa-miR-34a-5p.0.P1.S.32* | 3 | 0 | 3 | 1 |
|  | *hsa-miR-34a-5p.0.P1.S.33* | 3 | 2 | 13 | 17 |
|  | *hsa-miR-34a-5p.0.P1.S.34* | 0 | 0 | 0 | 2 |
|  | *hsa-miR-34a-5p.0.P1.S.35* | 0 | 2 | 28 | 7 |
|  | *hsa-miR-34a-5p.0.P1.S.36* | 0 | 0 | 0 | 1 |
|  | *hsa-miR-34a-5p.0.P1.S.37* | 0 | 2 | 22 | 7 |
|  | *hsa-miR-34a-5p.0.P1.S.38* | 0 | 0 | 8 | 5 |
|  | *hsa-miR-34a-5p.0.P1.S.39* | 0 | 2 | 17 | 8 |
|  | *hsa-miR-34a-5p.0.P1.S.40* | 19 | 0 | 0 | 3 |
|  | *hsa-miR-34a-5p.0.P1.S.41* | 3 | 2 | 27 | 14 |
|  | *hsa-miR-34a-5p.0.P1.S.42* | 0 | 0 | 0 | 4 |
|  | *hsa-miR-34a-5p.0.P1.S.43* | 0 | 0 | 0 | 1 |
|  | *hsa-miR-34a-5p.0.P1.S.44* | 0 | 2 | 39 | 11 |
|  | *hsa-miR-34a-5p.0.P1.S.45* | 0 | 7 | 10 | 10 |
|  | *hsa-miR-34a-5p.0.P1.S.46* | 0 | 0 | 0 | 1 |
|  | *hsa-miR-34a-5p.0.P1.S.47* | 7 | 31 | 92 | 88 |
|  | *hsa-miR-34a-5p.0.P1.S.48* | 3 | 435 | 84 | 73 |
|  | *hsa-miR-34a-5p.0.P1.S.49* | 19 | 25 | 18 | 35 |
| ***hsa-miR-122-5p*** | *hsa-miR-122-5p.3.P0.S.1* | 3283 | 258 | 112 | 8180 |
|  | *hsa-miR-122-5p.3.P0.S.13* | 8792 | 6287 | 10931 | 17194 |
|  | *hsa-miR-122-5p.3.P0.S.14* | 356 | 168 | 117 | 1010 |
|  | *hsa-miR-122-5p.3.P0.S.19* | 128 | 13 | 20 | 341 |
|  | *hsa-miR-122-5p.53.P0.S.4* | 69 | 63 | 174 | 122 |
|  | *hsa-miR-122-5p.5.P0.S.4* | 331 | 11 | 12 | 268 |
|  | *hsa-miR-122-5p.0.P1.S.45* | 107 | 2 | 3 | 225 |
|  | *hsa-miR-122-5p.0.P1.S.47* | 105 | 9 | 30 | 174 |
| ***hsa-miR-371a-5p*** | *hsa-miR-371a-5p.3.P0.S.0* | 0 | 0 | 0 | 1907 |
|  | *hsa-miR-371a-5p.3.P0.S.1* | 0 | 0 | 0 | 3 |
|  | *hsa-miR-371a-5p.3.P0.S.2* | 0 | 0 | 13 | 1141 |
|  | *hsa-miR-371a-5p.3.P0.S.3* | 0 | 0 | 3 | 83 |
|  | *hsa-miR-371a-5p.3.P0.S.4* | 0 | 0 | 0 | 3 |
|  | *hsa-miR-371a-5p.3.P0.S.5* | 0 | 0 | 0 | 19 |
|  | *hsa-miR-371a-5p.3.P0.S.6* | 0 | 0 | 5 | 114 |
|  | *hsa-miR-371a-5p.3.P0.S.7* | 0 | 0 | 0 | 41 |
|  | *hsa-miR-371a-5p.3.P0.S.8* | 0 | 0 | 0 | 67 |
|  | *hsa-miR-371a-5p.3.P0.S.9* | 0 | 0 | 0 | 7 |
|  | *hsa-miR-371a-5p.3.P0.S.10* | 65 | 2 | 59 | 11474 |
|  | *hsa-miR-371a-5p.3.P0.S.11* | 0 | 0 | 0 | 7 |
|  | *hsa-miR-371a-5p.3.P0.S.12* | 0 | 0 | 0 | 1 |
|  | *hsa-miR-371a-5p.3.P0.S.13* | 0 | 0 | 0 | 264 |
|  | *hsa-miR-371a-5p.3.P0.S.14* | 0 | 0 | 0 | 7 |
|  | *hsa-miR-371a-5p.3.P0.S.15* | 0 | 0 | 0 | 845 |
|  | *hsa-miR-371a-5p.3.P0.S.16* | 0 | 5 | 17 | 4728 |
|  | *hsa-miR-371a-5p.3.P0.S.17* | 7 | 5 | 20 | 528 |
|  | *hsa-miR-371a-5p.3.P0.S.18* | 0 | 0 | 0 | 313 |
|  | *hsa-miR-371a-5p.3.P0.S.19* | 0 | 0 | 0 | 1 |
|  | *hsa-miR-371a-5p.3.P0.S.20* | 0 | 0 | 0 | 13 |
|  | *hsa-miR-371a-5p.3.P0.S.21* | 0 | 0 | 0 | 3 |
|  | *hsa-miR-371a-5p.3.P0.S.22* | 0 | 0 | 0 | 8 |
|  | *hsa-miR-371a-5p.3.P0.S.23* | 0 | 0 | 0 | 270 |
|  | *hsa-miR-371a-5p.3.P0.S.24* | 0 | 0 | 0 | 60 |
|  | *hsa-miR-371a-5p.3.P0.S.25* | 0 | 5 | 13 | 4445 |
|  | *hsa-miR-371a-5p.3.P0.S.26* | 0 | 0 | 0 | 149 |
|  | *hsa-miR-371a-5p.3.P0.S.27* | 19 | 0 | 22 | 2412 |
|  | *hsa-miR-371a-5p.3.P1.S.0* | 0 | 0 | 0 | 1 |
|  | *hsa-miR-371a-5p.3.P1.S.1* | 0 | 0 | 0 | 2 |
|  | *hsa-miR-371a-5p.3.P1.S.2* | 0 | 0 | 0 | 9 |
|  | *hsa-miR-371a-5p.3.P1.S.3* | 0 | 0 | 0 | 13 |
|  | *hsa-miR-371a-5p.3.P1.S.4* | 0 | 0 | 0 | 6 |
|  | *hsa-miR-371a-5p.3.P1.S.5* | 0 | 0 | 0 | 3 |
|  | *hsa-miR-371a-5p.3.P1.S.6* | 0 | 0 | 0 | 1 |
|  | *hsa-miR-371a-5p.3.P1.S.7* | 19 | 0 | 0 | 0 |
|  | *hsa-miR-371a-5p.3.P1.S.8* | 0 | 0 | 0 | 1 |
|  | *hsa-miR-371a-5p.3.P1.S.9* | 0 | 0 | 0 | 2 |
|  | *hsa-miR-371a-5p.3.P1.S.10* | 0 | 0 | 0 | 1 |
|  | *hsa-miR-371a-5p.3.P1.S.11* | 0 | 0 | 0 | 4 |
|  | *hsa-miR-371a-5p.3.P1.S.12* | 0 | 0 | 0 | 3 |
|  | *hsa-miR-371a-5p.3.P1.S.13* | 0 | 0 | 0 | 3 |
|  | *hsa-miR-371a-5p.3.P1.S.14* | 0 | 0 | 0 | 1 |
|  | *hsa-miR-371a-5p.3.P1.S.15* | 0 | 0 | 0 | 3 |
|  | *hsa-miR-371a-5p.3.P1.S.16* | 0 | 0 | 0 | 3 |
|  | *hsa-miR-371a-5p.3.P1.S.17* | 0 | 0 | 0 | 1 |
|  | *hsa-miR-371a-5p.3.P1.S.18* | 0 | 0 | 0 | 1 |
|  | *hsa-miR-371a-5p.3.P1.S.19* | 0 | 0 | 0 | 12 |
|  | *hsa-miR-371a-5p.3.P1.S.20* | 0 | 0 | 0 | 1 |
|  | *hsa-miR-371a-5p.3.P1.S.21* | 7 | 0 | 0 | 285 |
|  | *hsa-miR-371a-5p.3.P1.S.22* | 0 | 5 | 5 | 229 |
|  | *hsa-miR-371a-5p.3.P1.S.23* | 0 | 0 | 3 | 10 |
|  | *hsa-miR-371a-5p.3.P1.S.24* | 0 | 0 | 0 | 1 |
|  | *hsa-miR-371a-5p.3.P1.S.25* | 0 | 0 | 0 | 1 |
|  | *hsa-miR-371a-5p.3.P0.S.28* | 39 | 0 | 22 | 9679 |
|  | *hsa-miR-371a-5p.3.P0.S.29* | 453 | 42 | 517 | 112925 |
|  | *hsa-miR-371a-5p.3.P1.S.26* | 0 | 0 | 0 | 1 |
|  | *hsa-miR-371a-5p.3.P1.S.27* | 0 | 0 | 0 | 8 |
|  | *hsa-miR-371a-5p.3.P1.S.28* | 0 | 0 | 0 | 5 |
|  | *hsa-miR-371a-5p.3.P1.S.29* | 0 | 0 | 0 | 28 |
|  | *hsa-miR-371a-5p.3.P1.S.30* | 0 | 0 | 0 | 1 |
|  | *hsa-miR-371a-5p.3.P1.S.31* | 0 | 0 | 0 | 3 |
|  | *hsa-miR-371a-5p.3.P1.S.32* | 0 | 0 | 0 | 5 |
|  | *hsa-miR-371a-5p.3.P1.S.33* | 0 | 0 | 0 | 101 |
|  | *hsa-miR-371a-5p.3.P1.S.34* | 0 | 0 | 0 | 13 |
|  | *hsa-miR-371a-5p.3.P1.S.35* | 0 | 5 | 8 | 133 |
|  | *hsa-miR-371a-5p.3.P1.S.36* | 0 | 0 | 0 | 7 |
|  | *hsa-miR-371a-5p.3.P1.S.37* | 19 | 0 | 0 | 128 |
|  | *hsa-miR-371a-5p.3.P1.S.38* | 0 | 0 | 0 | 6 |
|  | *hsa-miR-371a-5p.3.P1.S.39* | 0 | 0 | 0 | 3 |
|  | *hsa-miR-371a-5p.3.P1.S.40* | 0 | 0 | 0 | 45 |
|  | *hsa-miR-371a-5p.3.P1.S.41* | 0 | 0 | 0 | 8 |
|  | *hsa-miR-371a-5p.3.P1.S.42* | 0 | 0 | 0 | 8 |
|  | *hsa-miR-371a-5p.3.P1.S.43* | 0 | 0 | 0 | 4 |
|  | *hsa-miR-371a-5p.3.P1.S.44* | 0 | 0 | 0 | 28 |
|  | *hsa-miR-371a-5p.3.P1.S.45* | 0 | 0 | 0 | 1 |
|  | *hsa-miR-371a-5p.3.P1.S.46* | 0 | 0 | 0 | 5 |
|  | *hsa-miR-371a-5p.3.P1.S.47* | 0 | 0 | 0 | 33 |
|  | *hsa-miR-371a-5p.3.P1.S.48* | 0 | 0 | 0 | 5 |
|  | *hsa-miR-371a-5p.3.P1.S.49* | 0 | 0 | 0 | 1 |
|  | *hsa-miR-371a-5p.3.P1.S.50* | 0 | 0 | 0 | 34 |
|  | *hsa-miR-371a-5p.3.P1.S.51* | 0 | 0 | 0 | 7 |
|  | *hsa-miR-371a-5p.3.P1.S.52* | 0 | 0 | 0 | 1 |
|  | *hsa-miR-371a-5p.3.P1.S.53* | 0 | 0 | 0 | 2 |
|  | *hsa-miR-371a-5p.3.P1.S.54* | 0 | 0 | 0 | 60 |
|  | *hsa-miR-371a-5p.3.P1.S.55* | 0 | 0 | 0 | 1 |
|  | *hsa-miR-371a-5p.3.P1.S.56* | 0 | 0 | 0 | 2 |
|  | *hsa-miR-371a-5p.3.P1.S.57* | 0 | 0 | 0 | 9 |
|  | *hsa-miR-371a-5p.3.P1.S.58* | 0 | 0 | 0 | 6 |
|  | *hsa-miR-371a-5p.3.P1.S.59* | 0 | 0 | 0 | 2 |
|  | *hsa-miR-371a-5p.3.P1.S.60* | 0 | 0 | 0 | 24 |
|  | *hsa-miR-371a-5p.3.P1.S.61* | 0 | 0 | 0 | 1 |
|  | *hsa-miR-371a-5p.3.P1.S.62* | 0 | 0 | 0 | 1 |
|  | *hsa-miR-371a-5p.3.P1.S.63* | 0 | 0 | 0 | 7 |
|  | *hsa-miR-371a-5p.3.P1.S.64* | 0 | 0 | 0 | 3 |
|  | *hsa-miR-371a-5p.3.P1.S.65* | 0 | 0 | 0 | 49 |
|  | *hsa-miR-371a-5p.3.P1.S.66* | 0 | 0 | 0 | 2 |
|  | *hsa-miR-371a-5p.3.P1.S.67* | 0 | 0 | 0 | 2 |
|  | *hsa-miR-371a-5p.3.P1.S.68* | 0 | 0 | 0 | 1 |
|  | *hsa-miR-371a-5p.3.P1.S.69* | 0 | 0 | 0 | 50 |
|  | *hsa-miR-371a-5p.3.P1.S.70* | 0 | 0 | 0 | 1 |
|  | *hsa-miR-371a-5p.3.P1.S.71* | 0 | 0 | 0 | 29 |
|  | *hsa-miR-371a-5p.3.P1.S.72* | 0 | 0 | 0 | 1 |
|  | *hsa-miR-371a-5p.3.P1.S.73* | 0 | 0 | 0 | 8 |
|  | *hsa-miR-371a-5p.3.P1.S.74* | 0 | 0 | 0 | 2 |
|  | *hsa-miR-371a-5p.3.P1.S.75* | 0 | 0 | 0 | 1 |
|  | *hsa-miR-371a-5p.3.P1.S.76* | 0 | 0 | 0 | 4 |
|  | *hsa-miR-371a-5p.3.P1.S.77* | 0 | 0 | 0 | 37 |
|  | *hsa-miR-371a-5p.3.P1.S.78* | 0 | 0 | 0 | 4 |
|  | *hsa-miR-371a-5p.3.P1.S.79* | 0 | 0 | 0 | 1 |
|  | *hsa-miR-371a-5p.3.P1.S.80* | 0 | 0 | 0 | 5 |
|  | *hsa-miR-371a-5p.3.P1.S.81* | 0 | 0 | 0 | 41 |
|  | *hsa-miR-371a-5p.3.P1.S.82* | 0 | 0 | 0 | 4 |
|  | *hsa-miR-371a-5p.3.P1.S.83* | 19 | 0 | 13 | 1464 |
|  | *hsa-miR-371a-5p.3.P1.S.84* | 0 | 0 | 0 | 24 |
|  | *hsa-miR-371a-5p.3.P1.S.85* | 0 | 0 | 0 | 198 |
|  | *hsa-miR-371a-5p.3.P1.S.86* | 0 | 0 | 0 | 6 |
|  | *hsa-miR-371a-5p.3.P1.S.87* | 39 | 0 | 7 | 1884 |
|  | *hsa-miR-371a-5p.3.P1.S.88* | 0 | 0 | 0 | 40 |
|  | *hsa-miR-371a-5p.3.P1.S.89* | 0 | 0 | 0 | 110 |
|  | *hsa-miR-371a-5p.3.P1.S.90* | 0 | 0 | 0 | 113 |
|  | *hsa-miR-371a-5p.3.P1.S.91* | 0 | 0 | 0 | 13 |
|  | *hsa-miR-371a-5p.3.P1.S.92* | 0 | 0 | 0 | 1 |
|  | *hsa-miR-371a-5p.3.P1.S.93* | 0 | 0 | 0 | 5 |
|  | *hsa-miR-371a-5p.3.P1.S.94* | 0 | 0 | 0 | 55 |
|  | *hsa-miR-371a-5p.3.P1.S.95* | 19 | 0 | 0 | 0 |
|  | *hsa-miR-371a-5p.3.P1.S.96* | 0 | 0 | 0 | 1 |
|  | *hsa-miR-371a-5p.3.P1.S.97* | 0 | 0 | 0 | 4 |
|  | *hsa-miR-371a-5p.3.P1.S.98* | 0 | 0 | 0 | 21 |
|  | *hsa-miR-371a-5p.3.P1.S.99* | 0 | 0 | 0 | 1 |
|  | *hsa-miR-371a-5p.3.P1.S.100* | 0 | 0 | 0 | 2 |
|  | *hsa-miR-371a-5p.3.P1.S.101* | 0 | 0 | 0 | 62 |
|  | *hsa-miR-371a-5p.3.P1.S.102* | 0 | 0 | 0 | 7 |
|  | *hsa-miR-371a-5p.3.P1.S.103* | 0 | 0 | 0 | 3 |
|  | *hsa-miR-371a-5p.3.P1.S.104* | 0 | 0 | 0 | 2 |
|  | *hsa-miR-371a-5p.3.P1.S.105* | 0 | 0 | 0 | 2 |
|  | *hsa-miR-371a-5p.3.P1.S.106* | 0 | 0 | 0 | 27 |
|  | *hsa-miR-371a-5p.3.P0.S.30* | 3 | 9 | 37 | 7731 |
|  | *hsa-miR-371a-5p.3.P1.S.107* | 0 | 0 | 0 | 5 |
|  | *hsa-miR-371a-5p.3.P1.S.108* | 0 | 0 | 0 | 4 |
|  | *hsa-miR-371a-5p.3.P1.S.109* | 0 | 0 | 0 | 5 |
|  | *hsa-miR-371a-5p.3.P1.S.110* | 0 | 0 | 0 | 6 |
|  | *hsa-miR-371a-5p.3.P1.S.111* | 0 | 0 | 0 | 1 |
|  | *hsa-miR-371a-5p.3.P1.S.112* | 0 | 0 | 0 | 2 |
|  | *hsa-miR-371a-5p.3.P1.S.113* | 0 | 0 | 0 | 5 |
|  | *hsa-miR-371a-5p.3.P1.S.114* | 0 | 0 | 0 | 5 |
|  | *hsa-miR-371a-5p.3.P1.S.115* | 0 | 0 | 0 | 2 |
|  | *hsa-miR-371a-5p.3.P1.S.116* | 0 | 0 | 0 | 3 |
|  | *hsa-miR-371a-5p.3.P1.S.117* | 0 | 0 | 0 | 4 |
|  | *hsa-miR-371a-5p.3.P1.S.118* | 0 | 0 | 0 | 1 |
|  | *hsa-miR-371a-5p.3.P1.S.119* | 0 | 0 | 0 | 2 |
|  | *hsa-miR-371a-5p.3.P1.S.120* | 0 | 0 | 0 | 2 |
|  | *hsa-miR-371a-5p.3.P1.S.121* | 0 | 0 | 0 | 3 |
|  | *hsa-miR-371a-5p.3.P1.S.122* | 0 | 0 | 0 | 4 |
|  | *hsa-miR-371a-5p.3.P1.S.123* | 0 | 0 | 0 | 1 |
|  | *hsa-miR-371a-5p.3.P1.S.124* | 0 | 0 | 0 | 7 |
|  | *hsa-miR-371a-5p.3.P1.S.125* | 0 | 0 | 0 | 3 |
|  | *hsa-miR-371a-5p.3.P1.S.126* | 0 | 0 | 0 | 5 |
|  | *hsa-miR-371a-5p.3.P1.S.127* | 0 | 0 | 0 | 2 |
|  | *hsa-miR-371a-5p.3.P1.S.128* | 0 | 0 | 0 | 7 |
|  | *hsa-miR-371a-5p.3.P1.S.129* | 0 | 0 | 0 | 6 |
|  | *hsa-miR-371a-5p.3.P0.S.31* | 0 | 0 | 0 | 1 |
|  | *hsa-miR-371a-5p.3.P1.S.130* | 0 | 0 | 0 | 2 |
|  | *hsa-miR-371a-5p.3.P0.S.32* | 0 | 0 | 0 | 352 |
|  | *hsa-miR-371a-5p.3.P1.S.131* | 0 | 0 | 0 | 1 |
|  | *hsa-miR-371a-5p.3.P1.S.132* | 0 | 0 | 0 | 1 |
|  | *hsa-miR-371a-5p.3.P1.S.133* | 0 | 0 | 0 | 1 |
|  | *hsa-miR-371a-5p.3.P1.S.134* | 0 | 0 | 0 | 1 |
|  | *hsa-miR-371a-5p.3.P0.S.33* | 443 | 34 | 411 | 103110 |
|  | *hsa-miR-371a-5p.3.P1.S.135* | 0 | 0 | 0 | 11 |
|  | *hsa-miR-371a-5p.3.P1.S.136* | 0 | 0 | 0 | 34 |
|  | *hsa-miR-371a-5p.3.P1.S.137* | 3 | 0 | 0 | 4 |
|  | *hsa-miR-371a-5p.3.P1.S.138* | 19 | 0 | 0 | 54 |
|  | *hsa-miR-371a-5p.3.P1.S.139* | 3 | 5 | 8 | 96 |
|  | *hsa-miR-371a-5p.3.P1.S.140* | 0 | 0 | 0 | 36 |
|  | *hsa-miR-371a-5p.3.P1.S.141* | 0 | 0 | 0 | 5 |
|  | *hsa-miR-371a-5p.3.P1.S.142* | 0 | 0 | 0 | 43 |
|  | *hsa-miR-371a-5p.3.P1.S.143* | 0 | 0 | 0 | 1 |
|  | *hsa-miR-371a-5p.3.P1.S.144* | 0 | 0 | 0 | 2 |
|  | *hsa-miR-371a-5p.3.P1.S.145* | 0 | 0 | 0 | 23 |
|  | *hsa-miR-371a-5p.3.P1.S.146* | 0 | 0 | 0 | 30 |
|  | *hsa-miR-371a-5p.3.P1.S.147* | 0 | 0 | 0 | 1 |
|  | *hsa-miR-371a-5p.3.P1.S.148* | 0 | 0 | 0 | 27 |
|  | *hsa-miR-371a-5p.3.P1.S.149* | 0 | 0 | 0 | 1 |
|  | *hsa-miR-371a-5p.3.P1.S.150* | 0 | 0 | 0 | 4 |
|  | *hsa-miR-371a-5p.3.P1.S.151* | 0 | 0 | 0 | 2 |
|  | *hsa-miR-371a-5p.3.P1.S.152* | 0 | 0 | 0 | 52 |
|  | *hsa-miR-371a-5p.3.P1.S.153* | 0 | 0 | 0 | 3 |
|  | *hsa-miR-371a-5p.3.P1.S.154* | 0 | 0 | 0 | 19 |
|  | *hsa-miR-371a-5p.3.P1.S.155* | 0 | 0 | 0 | 1 |
|  | *hsa-miR-371a-5p.3.P1.S.156* | 0 | 0 | 0 | 41 |
|  | *hsa-miR-371a-5p.3.P1.S.157* | 0 | 0 | 0 | 1 |
|  | *hsa-miR-371a-5p.3.P1.S.158* | 0 | 0 | 0 | 43 |
|  | *hsa-miR-371a-5p.3.P1.S.159* | 0 | 0 | 0 | 3 |
|  | *hsa-miR-371a-5p.3.P1.S.160* | 0 | 0 | 0 | 4 |
|  | *hsa-miR-371a-5p.3.P1.S.161* | 0 | 0 | 0 | 31 |
|  | *hsa-miR-371a-5p.3.P1.S.162* | 0 | 0 | 0 | 2 |
|  | *hsa-miR-371a-5p.3.P1.S.163* | 0 | 0 | 0 | 31 |
|  | *hsa-miR-371a-5p.3.P1.S.164* | 0 | 0 | 0 | 1 |
|  | *hsa-miR-371a-5p.3.P1.S.165* | 0 | 0 | 0 | 2 |
|  | *hsa-miR-371a-5p.3.P1.S.166* | 0 | 0 | 0 | 25 |
|  | *hsa-miR-371a-5p.3.P1.S.167* | 0 | 0 | 0 | 27 |
|  | *hsa-miR-371a-5p.3.P1.S.168* | 0 | 0 | 0 | 3 |
|  | *hsa-miR-371a-5p.3.P1.S.169* | 0 | 0 | 0 | 6 |
|  | *hsa-miR-371a-5p.3.P1.S.170* | 0 | 0 | 0 | 2 |
|  | *hsa-miR-371a-5p.3.P1.S.171* | 0 | 0 | 0 | 24 |
|  | *hsa-miR-371a-5p.3.P1.S.172* | 0 | 0 | 0 | 12 |
|  | *hsa-miR-371a-5p.3.P1.S.173* | 0 | 0 | 0 | 102 |
|  | *hsa-miR-371a-5p.3.P1.S.174* | 0 | 0 | 0 | 2 |
|  | *hsa-miR-371a-5p.3.P1.S.175* | 0 | 0 | 0 | 1 |
|  | *hsa-miR-371a-5p.3.P1.S.176* | 0 | 0 | 0 | 47 |
|  | *hsa-miR-371a-5p.3.P1.S.177* | 0 | 0 | 0 | 1 |
|  | *hsa-miR-371a-5p.3.P1.S.178* | 0 | 0 | 0 | 2 |
|  | *hsa-miR-371a-5p.3.P1.S.179* | 0 | 0 | 0 | 19 |
|  | *hsa-miR-371a-5p.3.P1.S.180* | 0 | 0 | 5 | 49 |
|  | *hsa-miR-371a-5p.3.P1.S.181* | 0 | 0 | 0 | 2 |
|  | *hsa-miR-371a-5p.3.P1.S.182* | 0 | 0 | 0 | 19 |
|  | *hsa-miR-371a-5p.53.P0.S.0* | 0 | 0 | 0 | 2 |
|  | *hsa-miR-371a-5p.53.P0.S.1* | 0 | 0 | 0 | 9 |
|  | *hsa-miR-371a-5p.53.P0.S.2* | 0 | 0 | 0 | 2 |
|  | *hsa-miR-371a-5p.53.P0.S.3* | 0 | 0 | 0 | 1 |
|  | *hsa-miR-371a-5p.53.P0.S.4* | 0 | 0 | 0 | 2 |
|  | *hsa-miR-371a-5p.5.P0.S.0* | 0 | 0 | 0 | 4 |
|  | *hsa-miR-371a-5p.53.P0.S.5* | 0 | 0 | 0 | 3 |
|  | *hsa-miR-371a-5p.53.P0.S.6* | 0 | 0 | 0 | 1 |
|  | *hsa-miR-371a-5p.53.P0.S.7* | 0 | 0 | 0 | 1 |
|  | *hsa-miR-371a-5p.53.P0.S.8* | 0 | 0 | 0 | 1 |
|  | *hsa-miR-371a-5p.53.P0.S.9* | 0 | 0 | 0 | 1 |
|  | *hsa-miR-371a-5p.53.P0.S.10* | 0 | 0 | 0 | 2 |
|  | *hsa-miR-371a-5p.5.P0.S.1* | 0 | 0 | 0 | 2 |
|  | *hsa-miR-371a-5p.53.P0.S.11* | 0 | 0 | 0 | 1 |
|  | *hsa-miR-371a-5p.53.P0.S.12* | 0 | 0 | 0 | 1 |
|  | *hsa-miR-371a-5p.53.P0.S.13* | 0 | 0 | 0 | 16 |
|  | *hsa-miR-371a-5p.53.P0.S.14* | 0 | 0 | 0 | 16 |
|  | *hsa-miR-371a-5p.5.P0.S.2* | 0 | 0 | 0 | 1 |
|  | *hsa-miR-371a-5p.53.P0.S.15* | 0 | 0 | 0 | 20 |
|  | *hsa-miR-371a-5p.53.P0.S.16* | 0 | 0 | 0 | 14 |
|  | *hsa-miR-371a-5p.53.P0.S.17* | 0 | 0 | 0 | 1 |
|  | *hsa-miR-371a-5p.53.P0.S.18* | 0 | 0 | 0 | 4 |
|  | *hsa-miR-371a-5p.53.P0.S.19* | 0 | 0 | 0 | 2 |
|  | *hsa-miR-371a-5p.53.P0.S.20* | 0 | 0 | 0 | 85 |
|  | *hsa-miR-371a-5p.53.P0.S.21* | 0 | 0 | 0 | 4 |
|  | *hsa-miR-371a-5p.53.P0.S.22* | 0 | 0 | 0 | 11 |
|  | *hsa-miR-371a-5p.53.P0.S.23* | 0 | 0 | 0 | 243 |
|  | *hsa-miR-371a-5p.53.P0.S.24* | 0 | 0 | 0 | 43 |
|  | *hsa-miR-371a-5p.53.P0.S.25* | 0 | 0 | 0 | 71 |
|  | *hsa-miR-371a-5p.53.P0.S.26* | 0 | 0 | 0 | 5 |
|  | *hsa-miR-371a-5p.53.P0.S.27* | 0 | 0 | 0 | 14 |
|  | *hsa-miR-371a-5p.53.P0.S.28* | 0 | 0 | 0 | 3 |
|  | *hsa-miR-371a-5p.53.P0.S.29* | 0 | 0 | 0 | 379 |
|  | *hsa-miR-371a-5p.53.P0.S.30* | 0 | 0 | 0 | 21 |
|  | *hsa-miR-371a-5p.53.P0.S.31* | 0 | 0 | 0 | 31 |
|  | *hsa-miR-371a-5p.53.P0.S.32* | 0 | 0 | 0 | 1180 |
|  | *hsa-miR-371a-5p.53.P1.S.0* | 0 | 0 | 0 | 4 |
|  | *hsa-miR-371a-5p.53.P1.S.1* | 0 | 0 | 0 | 1 |
|  | *hsa-miR-371a-5p.53.P1.S.2* | 0 | 0 | 0 | 2 |
|  | *hsa-miR-371a-5p.53.P1.S.3* | 0 | 0 | 0 | 1 |
|  | *hsa-miR-371a-5p.53.P1.S.4* | 0 | 0 | 0 | 6 |
|  | *hsa-miR-371a-5p.53.P1.S.5* | 0 | 0 | 0 | 1 |
|  | *hsa-miR-371a-5p.53.P1.S.6* | 0 | 0 | 0 | 6 |
|  | *hsa-miR-371a-5p.53.P1.S.7* | 0 | 0 | 0 | 1 |
|  | *hsa-miR-371a-5p.53.P1.S.8* | 0 | 0 | 0 | 5 |
|  | *hsa-miR-371a-5p.53.P1.S.9* | 0 | 0 | 0 | 4 |
|  | *hsa-miR-371a-5p.53.P1.S.10* | 0 | 0 | 0 | 2 |
|  | *hsa-miR-371a-5p.53.P1.S.11* | 0 | 0 | 0 | 1 |
|  | *hsa-miR-371a-5p.53.P1.S.12* | 0 | 0 | 0 | 1 |
|  | *hsa-miR-371a-5p.53.P0.S.33* | 3 | 0 | 14 | 1016 |
|  | *hsa-miR-371a-5p.53.P1.S.13* | 0 | 0 | 0 | 1 |
|  | *hsa-miR-371a-5p.53.P1.S.14* | 0 | 0 | 0 | 1 |
|  | *hsa-miR-371a-5p.53.P1.S.15* | 0 | 0 | 0 | 1 |
|  | *hsa-miR-371a-5p.53.P1.S.16* | 0 | 0 | 0 | 2 |
|  | *hsa-miR-371a-5p.53.P1.S.17* | 0 | 0 | 0 | 4 |
|  | *hsa-miR-371a-5p.53.P1.S.18* | 0 | 0 | 0 | 1 |
|  | *hsa-miR-371a-5p.53.P1.S.19* | 0 | 0 | 0 | 1 |
|  | *hsa-miR-371a-5p.53.P1.S.20* | 0 | 0 | 0 | 1 |
|  | *hsa-miR-371a-5p.53.P1.S.21* | 0 | 0 | 0 | 1 |
|  | *hsa-miR-371a-5p.53.P1.S.22* | 0 | 0 | 0 | 1 |
|  | *hsa-miR-371a-5p.53.P1.S.23* | 0 | 0 | 0 | 1 |
|  | *hsa-miR-371a-5p.53.P0.S.34* | 0 | 0 | 10 | 77 |
|  | *hsa-miR-371a-5p.53.P0.S.35* | 39 | 7 | 30 | 5000 |
|  | *hsa-miR-371a-5p.53.P1.S.24* | 0 | 0 | 0 | 1 |
|  | *hsa-miR-371a-5p.53.P1.S.25* | 0 | 0 | 0 | 1 |
|  | *hsa-miR-371a-5p.53.P1.S.26* | 0 | 0 | 0 | 2 |
|  | *hsa-miR-371a-5p.53.P1.S.27* | 0 | 0 | 0 | 4 |
|  | *hsa-miR-371a-5p.53.P1.S.28* | 0 | 0 | 0 | 4 |
|  | *hsa-miR-371a-5p.53.P1.S.29* | 0 | 0 | 0 | 4 |
|  | *hsa-miR-371a-5p.53.P1.S.30* | 0 | 0 | 0 | 1 |
|  | *hsa-miR-371a-5p.53.P1.S.31* | 0 | 0 | 0 | 1 |
|  | *hsa-miR-371a-5p.53.P1.S.32* | 0 | 0 | 0 | 2 |
|  | *hsa-miR-371a-5p.53.P1.S.33* | 0 | 0 | 0 | 1 |
|  | *hsa-miR-371a-5p.53.P1.S.34* | 0 | 0 | 0 | 1 |
|  | *hsa-miR-371a-5p.53.P1.S.35* | 0 | 0 | 0 | 2 |
|  | *hsa-miR-371a-5p.53.P1.S.36* | 0 | 0 | 0 | 1 |
|  | *hsa-miR-371a-5p.53.P1.S.37* | 0 | 0 | 0 | 6 |
|  | *hsa-miR-371a-5p.53.P1.S.38* | 0 | 0 | 0 | 1 |
|  | *hsa-miR-371a-5p.53.P1.S.39* | 0 | 0 | 0 | 2 |
|  | *hsa-miR-371a-5p.53.P1.S.40* | 0 | 0 | 0 | 1 |
|  | *hsa-miR-371a-5p.53.P1.S.41* | 0 | 0 | 0 | 1 |
|  | *hsa-miR-371a-5p.53.P1.S.42* | 0 | 0 | 0 | 1 |
|  | *hsa-miR-371a-5p.53.P1.S.43* | 0 | 0 | 0 | 2 |
|  | *hsa-miR-371a-5p.53.P1.S.44* | 0 | 0 | 0 | 4 |
|  | *hsa-miR-371a-5p.53.P1.S.45* | 0 | 0 | 0 | 2 |
|  | *hsa-miR-371a-5p.53.P1.S.46* | 0 | 0 | 0 | 2 |
|  | *hsa-miR-371a-5p.53.P1.S.47* | 0 | 0 | 0 | 5 |
|  | *hsa-miR-371a-5p.53.P1.S.48* | 0 | 0 | 0 | 1 |
|  | *hsa-miR-371a-5p.53.P0.S.36* | 0 | 0 | 0 | 4 |
|  | *hsa-miR-371a-5p.53.P0.S.37* | 0 | 0 | 0 | 5 |
|  | *hsa-miR-371a-5p.53.P0.S.38* | 0 | 0 | 0 | 2 |
|  | *hsa-miR-371a-5p.53.P0.S.39* | 0 | 0 | 0 | 5 |
|  | *hsa-miR-371a-5p.53.P0.S.40* | 0 | 0 | 0 | 1 |
|  | *hsa-miR-371a-5p.53.P0.S.41* | 0 | 0 | 0 | 2 |
|  | *hsa-miR-371a-5p.53.P0.S.42* | 0 | 0 | 0 | 35 |
|  | *hsa-miR-371a-5p.53.P1.S.49* | 0 | 0 | 0 | 1 |
|  | *hsa-miR-371a-5p.53.P0.S.43* | 0 | 0 | 0 | 12 |
|  | *hsa-miR-371a-5p.53.P0.S.44* | 0 | 0 | 0 | 3 |
|  | *hsa-miR-371a-5p.53.P1.S.50* | 0 | 0 | 0 | 1 |
|  | *hsa-miR-371a-5p.53.P0.S.45* | 0 | 0 | 0 | 31 |
|  | *hsa-miR-371a-5p.5.P0.S.3* | 0 | 0 | 0 | 14 |
|  | *hsa-miR-371a-5p.5.P1.S.0* | 0 | 0 | 0 | 2 |
|  | *hsa-miR-371a-5p.53.P0.S.46* | 0 | 0 | 0 | 2 |
|  | *hsa-miR-371a-5p.53.P0.S.47* | 0 | 0 | 0 | 6 |
|  | *hsa-miR-371a-5p.53.P0.S.48* | 0 | 0 | 0 | 12 |
|  | *hsa-miR-371a-5p.53.P0.S.49* | 0 | 0 | 0 | 1 |
|  | *hsa-miR-371a-5p.53.P0.S.50* | 0 | 0 | 0 | 49 |
|  | *hsa-miR-371a-5p.53.P0.S.51* | 0 | 0 | 0 | 4 |
|  | *hsa-miR-371a-5p.53.P1.S.51* | 0 | 0 | 0 | 2 |
|  | *hsa-miR-371a-5p.53.P0.S.52* | 0 | 0 | 0 | 52 |
|  | *hsa-miR-371a-5p.53.P1.S.52* | 0 | 0 | 0 | 1 |
|  | *hsa-miR-371a-5p.53.P0.S.53* | 0 | 0 | 0 | 4 |
|  | *hsa-miR-371a-5p.53.P0.S.54* | 0 | 0 | 0 | 16 |
|  | *hsa-miR-371a-5p.53.P0.S.55* | 0 | 0 | 0 | 2 |
|  | *hsa-miR-371a-5p.53.P0.S.56* | 0 | 0 | 0 | 4 |
|  | *hsa-miR-371a-5p.53.P0.S.57* | 0 | 0 | 0 | 24 |
|  | *hsa-miR-371a-5p.53.P1.S.53* | 0 | 0 | 0 | 2 |
|  | *hsa-miR-371a-5p.53.P0.S.58* | 0 | 0 | 0 | 13 |
|  | *hsa-miR-371a-5p.53.P0.S.59* | 0 | 0 | 0 | 1 |
|  | *hsa-miR-371a-5p.53.P0.S.60* | 0 | 0 | 0 | 3 |
|  | *hsa-miR-371a-5p.5.P0.S.4* | 0 | 0 | 0 | 220 |
|  | *hsa-miR-371a-5p.5.P1.S.1* | 0 | 0 | 0 | 1 |
|  | *hsa-miR-371a-5p.5.P1.S.2* | 0 | 0 | 0 | 1 |
|  | *hsa-miR-371a-5p.5.P1.S.3* | 0 | 0 | 0 | 1 |
|  | *hsa-miR-371a-5p.5.P1.S.4* | 0 | 0 | 0 | 2 |
|  | *hsa-miR-371a-5p.5.P1.S.5* | 0 | 0 | 0 | 1 |
|  | *hsa-miR-371a-5p.5.P1.S.6* | 0 | 0 | 0 | 7 |
|  | *hsa-miR-371a-5p.53.P0.S.61* | 0 | 0 | 0 | 3 |
|  | *hsa-miR-371a-5p.53.P0.S.62* | 0 | 0 | 0 | 2 |
|  | *hsa-miR-371a-5p.53.P0.S.63* | 0 | 0 | 0 | 8 |
|  | *hsa-miR-371a-5p.53.P1.S.54* | 0 | 0 | 0 | 1 |
|  | *hsa-miR-371a-5p.53.P0.S.64* | 0 | 0 | 0 | 2 |
|  | *hsa-miR-371a-5p.5.P0.S.5* | 0 | 0 | 0 | 1 |
|  | *hsa-miR-371a-5p.0.P1.S.0* | 0 | 0 | 0 | 2 |
|  | *hsa-miR-371a-5p.0.P1.S.1* | 0 | 0 | 0 | 13 |
|  | *hsa-miR-371a-5p.0.P1.S.2* | 0 | 0 | 0 | 35 |
|  | *hsa-miR-371a-5p.0.P1.S.3* | 0 | 0 | 0 | 31 |
|  | *hsa-miR-371a-5p.0.P1.S.4* | 0 | 0 | 0 | 62 |
|  | *hsa-miR-371a-5p.0.P1.S.5* | 0 | 0 | 0 | 1 |
|  | *hsa-miR-371a-5p.0.P1.S.6* | 0 | 0 | 0 | 13 |
|  | *hsa-miR-371a-5p.0.P1.S.7* | 0 | 0 | 0 | 6 |
|  | *hsa-miR-371a-5p.0.P1.S.8* | 0 | 0 | 0 | 2 |
|  | *hsa-miR-371a-5p.0.P1.S.9* | 0 | 0 | 0 | 3 |
|  | *hsa-miR-371a-5p.0.P1.S.10* | 0 | 0 | 0 | 13 |
|  | *hsa-miR-371a-5p.0.P1.S.11* | 0 | 0 | 0 | 2 |
|  | *hsa-miR-371a-5p.0.P1.S.12* | 0 | 0 | 0 | 16 |
|  | *hsa-miR-371a-5p.0.P1.S.13* | 0 | 0 | 0 | 1 |
|  | *hsa-miR-371a-5p.0.P1.S.14* | 0 | 0 | 0 | 18 |
|  | *hsa-miR-371a-5p.0.P1.S.15* | 0 | 0 | 0 | 1 |
|  | *hsa-miR-371a-5p.0.P1.S.16* | 0 | 0 | 0 | 2 |
|  | *hsa-miR-371a-5p.0.P1.S.17* | 0 | 0 | 0 | 20 |
|  | *hsa-miR-371a-5p.0.P1.S.18* | 0 | 0 | 0 | 9 |
|  | *hsa-miR-371a-5p.0.P1.S.19* | 0 | 0 | 0 | 15 |
|  | *hsa-miR-371a-5p.0.P1.S.20* | 0 | 0 | 0 | 1 |
|  | *hsa-miR-371a-5p.0.P1.S.21* | 0 | 0 | 0 | 4 |
|  | *hsa-miR-371a-5p.0.P1.S.22* | 0 | 0 | 0 | 15 |
|  | *hsa-miR-371a-5p.0.P1.S.23* | 0 | 0 | 0 | 7 |
|  | *hsa-miR-371a-5p.0.P1.S.24* | 0 | 0 | 0 | 2 |
|  | *hsa-miR-371a-5p.0.P1.S.25* | 0 | 0 | 0 | 4 |
|  | *hsa-miR-371a-5p.0.P1.S.26* | 0 | 0 | 0 | 11 |
|  | *hsa-miR-371a-5p.0.P1.S.27* | 0 | 0 | 0 | 6 |
|  | *hsa-miR-371a-5p.0.P1.S.28* | 0 | 0 | 0 | 22 |
|  | *hsa-miR-371a-5p.0.P1.S.29* | 0 | 0 | 0 | 1 |
|  | *hsa-miR-371a-5p.0.P1.S.30* | 0 | 0 | 0 | 454 |
|  | *hsa-miR-371a-5p.0.P1.S.31* | 0 | 0 | 0 | 32 |
|  | *hsa-miR-371a-5p.0.P1.S.32* | 0 | 0 | 0 | 339 |
|  | *hsa-miR-371a-5p.0.P1.S.33* | 0 | 0 | 0 | 825 |
|  | *hsa-miR-371a-5p.0.P1.S.34* | 0 | 0 | 0 | 50 |
|  | *hsa-miR-371a-5p.0.P1.S.35* | 0 | 0 | 0 | 92 |
|  | *hsa-miR-371a-5p.0.P1.S.36* | 0 | 0 | 0 | 1 |
|  | *hsa-miR-371a-5p.0.P1.S.37* | 19 | 0 | 0 | 14 |
|  | *hsa-miR-371a-5p.0.P1.S.38* | 0 | 0 | 0 | 1 |
|  | *hsa-miR-371a-5p.0.P1.S.39* | 0 | 0 | 0 | 2 |
|  | *hsa-miR-371a-5p.0.P1.S.40* | 0 | 0 | 0 | 9 |
|  | *hsa-miR-371a-5p.0.P1.S.41* | 0 | 0 | 0 | 19 |
|  | *hsa-miR-371a-5p.0.P1.S.42* | 0 | 0 | 0 | 2 |
|  | *hsa-miR-371a-5p.0.P1.S.43* | 0 | 0 | 0 | 4 |
