# Supplementary material for: RNA-seq reveals distinctive RNA profiles of small extracellular vesicles from different human liver cancer cell lines
Source: Oncotarget. 2017 Aug 24;8(47):82920–39. doi: 10.18632/oncotarget.20503 (PMC5669939; doi:10.18632/oncotarget.20503)
Supplement: Supplementary file 6 [file oncotarget-08-82920-s006.docx]

**Table S5. snoRNAs**

| **Ensembl Gene ID** | **Gene Symbol** | **HuH7-EVs_1** | **HuH7-EVs_2** | **Hep3B-EVs_1** | **Hep3b-EVs_2** | **HepG2-EVs_1** | **HepG2-EVs_2** | **HuH6-EVs_1** | **HuH6-EVs_2** |
| --- | --- | --- | --- | --- | --- | --- | --- | --- | --- |
| ENSG00000200087 | SNORA73B | 57767 | 66835 | 29338 | 25915 | 34407 | 48810 | 58926 | 70039 |
| ENSG00000209582 | SNORA48 | 36314 | 40995 | 38959 | 33930 | 34848 | 35553 | 21967 | 16676 |
| ENSG00000274266 | SNORA73A | 37477 | 37109 | 24448 | 14961 | 20732 | 23200 | 33821 | 23346 |
| ENSG00000200983 | SNORA3A | 31145 | 37109 | 36593 | 13358 | 19850 | 19584 | 8368 | 10561 |
| ENSG00000200320 | SNORA63 | 37736 | 29532 | 50315 | 53433 | 31319 | 26514 | 25453 | 21679 |
| ENSG00000276788 | SNORD26 | 31533 | 25063 | 17508 | 23778 | 24261 | 21995 | 33124 | 33908 |
| ENSG00000277194 | SNORD22 | 33859 | 17680 | 20978 | 36468 | 26026 | 24706 | 28591 | 35575 |
| ENSG00000221500 | SNORD100 | 20031 | 22149 | 15457 | 24980 | 35730 | 29527 | 18480 | 20011 |
| ENSG00000273544 | SNORA44 | 14345 | 19817 | 18454 | 9885 | 27349 | 32841 | 20572 | 11673 |
| ENSG00000221420 | SNORA81 | 23003 | 13017 | 18454 | 44750 | 7499 | 8738 | 10112 | 9450 |
| ENSG00000201302 | SNORA65 | 13311 | 17486 | 12618 | 5610 | 13233 | 15366 | 10809 | 11673 |
| ENSG00000201675 | SNORD32A | 12536 | 16320 | 8517 | 12290 | 13674 | 15065 | 5579 | 6115 |
| ENSG00000199477 | SNORA31 | 9434 | 18652 | 7729 | 4141 | 15439 | 10244 | 5927 | 3335 |
| ENSG00000199631 | SNORD33 | 17446 | 12046 | 7571 | 10954 | 16762 | 12353 | 10112 | 12229 |
| ENSG00000274582 | SNORA16A | 11502 | 15349 | 22871 | 7080 | 13674 | 18680 | 28243 | 16676 |
| ENSG00000280498 | SNORA16A | 11502 | 15349 | 22871 | 7080 | 13674 | 18680 | 28243 | 16676 |
| ENSG00000199293 | SNORA21 | 10468 | 15737 | 24606 | 11755 | 25584 | 28623 | 12901 | 8894 |
| ENSG00000207166 | SNORA68 | 9822 | 13989 | 16719 | 9084 | 8822 | 10847 | 7322 | 6115 |
| ENSG00000212588 | SNORA26 | 13828 | 10297 | 7098 | 8282 | 3088 | 4218 | 3487 | 556 |
| ENSG00000209480 | SNORD83B | 11502 | 9909 | 3785 | 9217 | 5734 | 5122 | 5927 | 11117 |
| ENSG00000206799 | SNORA32 | 8659 | 11657 | 8833 | 7481 | 7940 | 6026 | 5927 | 5003 |
| ENSG00000207523 | SNORA66 | 9822 | 10492 | 12303 | 9351 | 11469 | 12353 | 5927 | 6670 |
| ENSG00000200354 | SNORA71D | 8917 | 10492 | 3785 | 3874 | 17644 | 13257 | 4184 | 2779 |
| ENSG00000235408 | SNORA71B | 10209 | 8743 | 11356 | 11087 | 18527 | 16873 | 4533 | 2779 |
| ENSG00000207392 | SNORA20 | 9434 | 8743 | 9779 | 11087 | 4852 | 3917 | 3138 | 1668 |
| ENSG00000251898 | SCARNA11 | 4652 | 10880 | 23186 | 8416 | 9704 | 6327 | 2441 | 2223 |
| ENSG00000200084 | SNORD68 | 9305 | 6800 | 3943 | 2137 | 5293 | 6629 | 4881 | 6115 |
| ENSG00000276161 | SNORA17B | 8659 | 6606 | 4890 | 3206 | 5293 | 5423 | 9066 | 5559 |
| ENSG00000280496 | SNORA17B | 8659 | 6606 | 4890 | 3206 | 5293 | 5423 | 9066 | 5559 |
| ENSG00000207165 | SNORA70 | 7754 | 7189 | 5363 | 3473 | 7058 | 7532 | 5927 | 6670 |
| ENSG00000207181 | SNORA14B | 7495 | 6994 | 8360 | 8416 | 5293 | 5423 | 11855 | 7782 |
| ENSG00000201457 | SNORA55 | 7754 | 6412 | 7413 | 6278 | 7940 | 7532 | 9066 | 5559 |
| ENSG00000202363 | SNORA62 | 7495 | 6606 | 2681 | 5343 | 4411 | 2109 | 4533 | 6115 |
| ENSG00000201998 | SNORA23 | 5557 | 7577 | 8991 | 8015 | 3529 | 5725 | 4184 | 7226 |
| ENSG00000225091 | SNORA71A | 5686 | 7383 | 5678 | 2939 | 11028 | 12654 | 5230 | 5003 |
| ENSG00000202031 | SNORD38A | 8659 | 5051 | 3785 | 2672 | 7499 | 8738 | 14644 | 15564 |
| ENSG00000274998 | SNORA17A | 6462 | 6606 | 2681 | 2271 | 6176 | 3917 | 8020 | 10561 |
| ENSG00000281808 | SNORA17 | 6462 | 6606 | 2681 | 2271 | 6176 | 3917 | 8020 | 10561 |
| ENSG00000207008 | SNORA54 | 6074 | 6800 | 8202 | 8149 | 10587 | 5725 | 6276 | 1668 |
| ENSG00000200534 | SNORA33 | 6074 | 6023 | 5836 | 14293 | 2206 | 3314 | 4184 | 6115 |
| ENSG00000206760 | SNORA6 | 6203 | 5829 | 3785 | 3340 | 1764 | 2712 | 5579 | 3335 |
| ENSG00000202503 | SNORD34 | 5945 | 6023 | 3628 | 5878 | 5293 | 5122 | 4184 | 7782 |
| ENSG00000209482 | SNORD83A | 5040 | 6412 | 2524 | 4542 | 2647 | 1808 | 6625 | 4447 |
| ENSG00000239002 | SCARNA10 | 5686 | 5634 | 7098 | 7481 | 3088 | 5423 | 2092 | 4447 |
| ENSG00000264549 | SNORD95 | 6979 | 4469 | 5047 | 6546 | 9263 | 2712 | 14644 | 15564 |
| ENSG00000277846 | SNORD30 | 5299 | 5440 | 6309 | 9484 | 4411 | 1808 | 5927 | 7782 |
| ENSG00000222489 | SNORA79 | 3748 | 6606 | 10883 | 8416 | 7499 | 5725 | 3138 | 2223 |
| ENSG00000238622 | SNORD97 | 4394 | 5829 | 2839 | 2404 | 2206 | 1506 | 1046 | 4447 |
| ENSG00000207304 | SNORA8 | 5945 | 4469 | 5363 | 6679 | 7499 | 3314 | 2789 | 6670 |
| ENSG00000278274 | SNORA61 | 6332 | 4080 | 7256 | 6145 | 9704 | 10545 | 13947 | 18899 |
| ENSG00000275996 | SNORD27 | 6203 | 3691 | 5521 | 7748 | 3088 | 5423 | 9414 | 13341 |
| ENSG00000212607 | SNORA3B | 5686 | 4080 | 4259 | 6546 | 3088 | 4218 | 4533 | 12785 |
| ENSG00000263776 | SNORA4 | 4652 | 4857 | 6467 | 7080 | 10146 | 2712 | 2789 | 5559 |
| ENSG00000253051 | SNORA31 | 2585 | 6412 | 1420 | 1469 | 4411 | 4821 | 1743 | 556 |
| ENSG00000207475 | SNORA80E | 4265 | 4663 | 14038 | 18568 | 3970 | 3616 | 4533 | 7226 |
| ENSG00000251733 | SCARNA8 | 5428 | 3691 | 20505 | 6145 | 7940 | 14161 | 4184 | 3335 |
| ENSG00000212443 | SNORA53 | 3360 | 5051 | 3312 | 4809 | 1323 | 1506 | 3487 | 2779 |
| ENSG00000275994 | SNORA24 | 4523 | 4080 | 4101 | 4675 | 4411 | 4821 | 1046 | 4447 |
| ENSG00000200394 | SNORA38B | 3489 | 4857 | 9306 | 3340 | 3088 | 2410 | 2789 | 2223 |
| ENSG00000252010 | SCARNA5 | 3489 | 4857 | 6625 | 5210 | 14116 | 8738 | 3487 | 2779 |
| ENSG00000207112 | SNORA25 | 4523 | 3886 | 3155 | 4275 | 7058 | 11148 | 7671 | 9450 |
| ENSG00000199753 | SNORD104 | 3231 | 4857 | 4416 | 6145 | 10587 | 7231 | 12901 | 12785 |
| ENSG00000200959 | SNORA74A | 3360 | 4663 | 5521 | 3874 | 5293 | 3314 | 7322 | 3891 |
| ENSG00000207405 | SNORA64 | 4911 | 3303 | 4416 | 3340 | 2206 | 4821 | 2441 | 3891 |
| ENSG00000238363 | SNORA13 | 4394 | 3691 | 4259 | 4675 | 2647 | 3013 | 4184 | 556 |
| ENSG00000206811 | SNORA10 | 3877 | 4080 | 3312 | 1336 | 882 | 3013 | 349 | 1668 |
| ENSG00000200259 | SNORD35A | 3231 | 4469 | 2208 | 2137 | 882 | 2712 | 3487 | 3335 |
| ENSG00000275072 | SNORD50B | 7237 | 1360 | 631 | 2404 | 4411 | 2712 | 3138 | 3335 |
| ENSG00000206838 | SNORA5A | 4394 | 3497 | 14826 | 12022 | 4852 | 3314 | 4184 | 3335 |
| ENSG00000277184 | SNORA9 | 3489 | 4080 | 2208 | 2939 | 441 | 0 | 2789 | 3891 |
| ENSG00000209042 | SNORD12C | 4135 | 3497 | 4890 | 6145 | 7940 | 7834 | 8717 | 7782 |
| ENSG00000263764 | SNORD43 | 4135 | 3497 | 2366 | 3072 | 1323 | 2712 | 3835 | 9450 |
| ENSG00000252481 | SCARNA13 | 2455 | 4663 | 15300 | 7881 | 7058 | 8738 | 2441 | 6115 |
| ENSG00000207406 | SNORA41 | 3619 | 3497 | 4732 | 6946 | 4411 | 4821 | 2092 | 1112 |
| ENSG00000277887 | SNORA50C | 2972 | 3886 | 2366 | 2404 | 5293 | 7532 | 3487 | 1668 |
| ENSG00000281311 | SNORA50 | 2972 | 3886 | 2366 | 2404 | 5293 | 7532 | 3487 | 1668 |
| ENSG00000206630 | SNORD60 | 5945 | 1554 | 631 | 1870 | 1764 | 2712 | 1395 | 3335 |
| ENSG00000281010 | snoR1 | 5945 | 1554 | 631 | 1870 | 1764 | 2712 | 1395 | 3335 |
| ENSG00000275143 | SCARNA16 | 3360 | 3497 | 9306 | 5210 | 4852 | 10244 | 4533 | 4447 |
| ENSG00000280466 | SCARNA4 | 2843 | 3886 | 5994 | 6412 | 4852 | 4519 | 2441 | 2223 |
| ENSG00000281394 | SCARNA4 | 2843 | 3886 | 5994 | 6412 | 4852 | 4519 | 2441 | 2223 |
| ENSG00000200623 | SNORD18A | 3619 | 3109 | 2208 | 2805 | 1323 | 1506 | 1046 | 1112 |
| ENSG00000206597 | SNORA57 | 3748 | 2914 | 6467 | 5076 | 5293 | 3314 | 6276 | 7782 |
| ENSG00000254341 | SNORD87 | 2714 | 3691 | 1735 | 2404 | 2647 | 904 | 8717 | 13897 |
| ENSG00000212452 | SNORD69 | 2455 | 3886 | 3312 | 3340 | 441 | 1506 | 4881 | 6115 |
| ENSG00000212135 | SNORD67 | 3619 | 2914 | 3470 | 8416 | 2647 | 3314 | 5579 | 1668 |
| ENSG00000239039 | SNORD13 | 3360 | 3109 | 1262 | 2672 | 3088 | 6026 | 4881 | 5559 |
| ENSG00000208797 | SNORD73A | 4265 | 2331 | 2208 | 2672 | 441 | 3314 | 4533 | 3891 |
| ENSG00000251791 | SCARNA6 | 4265 | 2331 | 2208 | 2939 | 8381 | 9641 | 2789 | 1668 |
| ENSG00000212158 | SNORD66 | 3489 | 2914 | 2839 | 5744 | 1764 | 2109 | 2092 | 6115 |
| ENSG00000207421 | SNORD38B | 4135 | 2331 | 1735 | 2137 | 3088 | 2712 | 6974 | 8338 |
| ENSG00000281859 | SNORD38B | 4135 | 2331 | 1735 | 2137 | 3088 | 2712 | 6974 | 8338 |
| ENSG00000201129 | SNORA58 | 3619 | 2720 | 7098 | 5610 | 2647 | 1205 | 3835 | 2223 |
| ENSG00000277985 | SNORA67 | 3102 | 3109 | 1577 | 4542 | 3088 | 603 | 3487 | 3891 |
| ENSG00000278249 | SCARNA2 | 3748 | 2526 | 3155 | 2004 | 7499 | 5122 | 6276 | 10006 |
| ENSG00000202252 | SNORD14C | 2972 | 3109 | 2050 | 3473 | 6617 | 6327 | 10112 | 9450 |
| ENSG00000221241 | SNORD88A | 3360 | 2720 | 946 | 4141 | 3970 | 5122 | 4533 | 3335 |
| ENSG00000275043 | SNORD25 | 2843 | 3109 | 1262 | 4675 | 3088 | 1506 | 1395 | 3335 |
| ENSG00000207067 | SNORA72 | 2585 | 3303 | 3628 | 3874 | 2206 | 1808 | 2789 | 1112 |
| ENSG00000238649 | SNORD42A | 3360 | 2526 | 1577 | 2004 | 3970 | 1808 | 3835 | 2779 |
| ENSG00000265236 | SNORD84 | 2585 | 3109 | 3628 | 4275 | 2206 | 3917 | 1395 | 1668 |
| ENSG00000220988 | SNORD88C | 2714 | 2914 | 1577 | 5076 | 4852 | 904 | 1046 | 556 |
| ENSG00000221116 | SNORD110 | 2455 | 3109 | 2050 | 5477 | 5293 | 2109 | 5230 | 1112 |
| ENSG00000206680 | SNORD21 | 3102 | 2526 | 789 | 935 | 3529 | 904 | 1046 | 2779 |
| ENSG00000238835 | SCARNA18 | 2714 | 2720 | 4101 | 3072 | 3529 | 2712 | 2092 | 1112 |
| ENSG00000249020 | SNORA58 | 2455 | 2914 | 3470 | 2939 | 3088 | 2712 | 1395 | 3335 |
| ENSG00000201512 | SNORA71C | 2843 | 2526 | 2208 | 935 | 2206 | 7231 | 2441 | 1112 |
| ENSG00000264346 | SNORA77 | 2197 | 2914 | 4574 | 3072 | 4411 | 1808 | 4533 | 1668 |
| ENSG00000249784 | SCARNA22 | 1680 | 3303 | 5047 | 3072 | 4852 | 6629 | 1395 | 556 |
| ENSG00000199785 | SNORA52 | 2843 | 2331 | 3785 | 1870 | 3529 | 6629 | 1743 | 2779 |
| ENSG00000207280 | SNORD20 | 2068 | 2914 | 1577 | 2538 | 5734 | 4519 | 1046 | 3891 |
| ENSG00000200530 | SNORD35B | 2455 | 2526 | 946 | 3206 | 1323 | 603 | 0 | 1112 |
| ENSG00000206885 | SNORA75 | 2455 | 2526 | 2208 | 3607 | 1764 | 2410 | 697 | 1112 |
| ENSG00000206611 | SNORD24 | 1422 | 3303 | 946 | 668 | 0 | 301 | 1395 | 2223 |
| ENSG00000201772 | SNORA5C | 1422 | 3109 | 2839 | 2805 | 3088 | 2712 | 1046 | 2223 |
| ENSG00000212232 | SNORD17 | 2843 | 1943 | 1893 | 3473 | 8381 | 7834 | 11506 | 12229 |
| ENSG00000221491 | SNORA2C | 2068 | 2526 | 6151 | 4542 | 1764 | 2109 | 1046 | 2779 |
| ENSG00000206622 | SNORA69 | 1809 | 2720 | 1420 | 1336 | 441 | 603 | 1046 | 1112 |
| ENSG00000239183 | SNORA84 | 2843 | 1749 | 1893 | 2672 | 3529 | 904 | 2092 | 1668 |
| ENSG00000238795 | SCARNA12 | 775 | 3303 | 1893 | 1737 | 3970 | 3616 | 4184 | 556 |
| ENSG00000207233 | SNORA37 | 1551 | 2526 | 3785 | 1870 | 3088 | 1506 | 349 | 556 |
| ENSG00000207493 | SNORA46 | 2843 | 1360 | 1420 | 4141 | 1323 | 1205 | 1395 | 0 |
| ENSG00000212304 | SNORD12 | 2326 | 1749 | 1893 | 3473 | 5734 | 9039 | 5579 | 5559 |
| ENSG00000277512 | SNORD65 | 1292 | 2331 | 631 | 3473 | 441 | 603 | 8020 | 7226 |
| ENSG00000238531 | SNORD105B | 2455 | 1360 | 473 | 2538 | 2647 | 1506 | 349 | 1668 |
| ENSG00000238917 | SNORD10 | 1422 | 2137 | 631 | 2137 | 2206 | 1506 | 2441 | 1668 |
| ENSG00000206612 | SNORA2A | 2068 | 1554 | 3628 | 2404 | 882 | 3616 | 4184 | 1668 |
| ENSG00000200816 | SNORA38 | 1292 | 2137 | 3943 | 4275 | 3529 | 3917 | 1046 | 0 |
| ENSG00000206633 | SNORA80B | 1292 | 2137 | 1577 | 1069 | 1764 | 603 | 697 | 0 |
| ENSG00000201229 | SNORA63 | 775 | 2526 | 1104 | 801 | 882 | 904 | 349 | 4447 |
| ENSG00000252577 | SCARNA20 | 2714 | 971 | 2208 | 1336 | 2647 | 3013 | 0 | 556 |
| ENSG00000200879 | SNORD14E | 1422 | 1943 | 2366 | 1603 | 3970 | 3314 | 2789 | 1112 |
| ENSG00000265706 | SNORD53_SNORD92 | 1809 | 1554 | 1104 | 2137 | 2206 | 603 | 3835 | 2779 |
| ENSG00000238423 | SNORD42B | 1551 | 1749 | 789 | 1603 | 1323 | 1506 | 1046 | 1112 |
| ENSG00000201643 | SNORA14A | 1938 | 1360 | 946 | 935 | 441 | 1506 | 1395 | 556 |
| ENSG00000207241 | SNORD45A | 2326 | 971 | 473 | 2271 | 1764 | 1808 | 3138 | 1668 |
| ENSG00000206602 | SNORD58A | 1680 | 1360 | 631 | 2004 | 1764 | 603 | 2789 | 1112 |
| ENSG00000212283 | SNORD89 | 1680 | 1360 | 1104 | 1870 | 7058 | 5423 | 2092 | 3335 |
| ENSG00000252712 | SCARNA14 | 1422 | 1554 | 2366 | 3072 | 882 | 2109 | 697 | 556 |
| ENSG00000206834 | SNORA1 | 1809 | 1166 | 1577 | 1336 | 1323 | 1205 | 697 | 1112 |
| ENSG00000264294 | SNORD55 | 1809 | 1166 | 1262 | 1603 | 441 | 2712 | 3487 | 0 |
| ENSG00000202314 | SNORD6 | 1034 | 1749 | 946 | 1469 | 1323 | 1808 | 4881 | 2779 |
| ENSG00000207313 | SNORA2B | 1680 | 1166 | 2681 | 3473 | 1764 | 1205 | 0 | 1112 |
| ENSG00000206979 | SNORD61 | 1163 | 1554 | 631 | 4408 | 882 | 0 | 697 | 2223 |
| ENSG00000207145 | SNORA18 | 1551 | 1166 | 1577 | 1870 | 1764 | 0 | 2789 | 2779 |
| ENSG00000200792 | SNORA80A | 1292 | 1360 | 2524 | 1202 | 3529 | 904 | 1743 | 2779 |
| ENSG00000207051 | SNORA27 | 1680 | 971 | 789 | 2672 | 882 | 2410 | 697 | 1668 |
| ENSG00000208892 | SNORA49 | 1680 | 971 | 473 | 1336 | 0 | 301 | 2092 | 1112 |
| ENSG00000207445 | SNORD15B | 1422 | 1166 | 1262 | 1202 | 1764 | 1808 | 2092 | 6115 |
| ENSG00000221539 | SNORD99 | 1551 | 971 | 631 | 267 | 441 | 0 | 3138 | 3891 |
| ENSG00000239127 | SNORD125 | 1551 | 971 | 315 | 801 | 882 | 603 | 697 | 0 |
| ENSG00000265145 | SNORD53 | 1034 | 1360 | 789 | 1469 | 3529 | 1808 | 5230 | 3335 |
| ENSG00000271982 | SNORD58B | 905 | 1360 | 473 | 1069 | 882 | 301 | 697 | 0 |
| ENSG00000272296 | SNORD96A | 1292 | 971 | 789 | 1603 | 441 | 2109 | 3487 | 3335 |
| ENSG00000199574 | SNORD18C | 1034 | 1166 | 158 | 668 | 0 | 904 | 1395 | 0 |
| ENSG00000212402 | SNORA74B | 1034 | 1166 | 1420 | 1336 | 882 | 1506 | 1395 | 1668 |
| ENSG00000212309 | SNORD70 | 517 | 1554 | 158 | 668 | 0 | 301 | 697 | 556 |
| ENSG00000238942 | SNORD2 | 517 | 1554 | 1893 | 1469 | 882 | 603 | 2092 | 556 |
| ENSG00000200913 | SNORD46 | 1422 | 777 | 315 | 534 | 441 | 904 | 2092 | 1668 |
| ENSG00000272533 | SNORA28 | 1422 | 777 | 2681 | 2805 | 1323 | 2712 | 1395 | 556 |
| ENSG00000277370 | SNORD49A | 1422 | 777 | 473 | 1069 | 882 | 0 | 3835 | 1668 |
| ENSG00000210825 | SNORA40 | 1163 | 971 | 631 | 935 | 441 | 301 | 697 | 1112 |
| ENSG00000201754 | SNORD52 | 646 | 1360 | 1735 | 4141 | 2647 | 603 | 1743 | 2223 |
| ENSG00000206948 | SNORA36A | 646 | 1360 | 946 | 801 | 0 | 301 | 1046 | 0 |
| ENSG00000199363 | SNORA63 | 1034 | 971 | 1104 | 801 | 441 | 301 | 349 | 556 |
| ENSG00000209645 | SNORD105 | 775 | 1166 | 631 | 267 | 0 | 0 | 1046 | 0 |
| ENSG00000199266 | SNORA60 | 258 | 1554 | 2997 | 1737 | 882 | 2109 | 2441 | 0 |
| ENSG00000226572 | SNORD57 | 1163 | 777 | 473 | 534 | 882 | 301 | 2441 | 3335 |
| ENSG00000229686 | SNORD56 | 1163 | 777 | 473 | 1870 | 882 | 1506 | 8717 | 5559 |
| ENSG00000274544 | SNORD28 | 905 | 971 | 158 | 1202 | 441 | 0 | 1046 | 1112 |
| ENSG00000238741 | SCARNA7 | 905 | 971 | 1577 | 1737 | 882 | 3616 | 3487 | 2779 |
| ENSG00000271798 | SNORA51 | 1551 | 389 | 473 | 2137 | 0 | 603 | 2092 | 3335 |
| ENSG00000206941 | SNORD15A | 1292 | 583 | 158 | 668 | 3088 | 2109 | 4184 | 1112 |
| ENSG00000207468 | SNORA19 | 1292 | 583 | 1577 | 801 | 441 | 1506 | 1395 | 1668 |
| ENSG00000238578 | SNORD4A | 1034 | 777 | 473 | 0 | 0 | 1205 | 1743 | 556 |
| ENSG00000272034 | SNORD14A | 517 | 1166 | 158 | 401 | 441 | 301 | 1046 | 0 |
| ENSG00000201823 | SNORD48 | 258 | 1360 | 1262 | 1336 | 1323 | 1205 | 3138 | 1668 |
| ENSG00000207047 | SNORD51 | 258 | 1360 | 946 | 801 | 882 | 0 | 697 | 556 |
| ENSG00000281780 | snoZ196 | 258 | 1360 | 946 | 801 | 882 | 0 | 697 | 556 |
| ENSG00000200418 | SNORA63 | 1422 | 389 | 2208 | 1603 | 441 | 0 | 2789 | 556 |
| ENSG00000238961 | SNORA47 | 905 | 777 | 1420 | 668 | 1323 | 0 | 697 | 1112 |
| ENSG00000199961 | SNORD1B | 646 | 971 | 631 | 534 | 2647 | 603 | 2441 | 556 |
| ENSG00000208772 | SNORD94 | 775 | 777 | 1735 | 2271 | 882 | 1506 | 2092 | 2223 |
| ENSG00000202400 | SNORD82 | 517 | 971 | 315 | 401 | 0 | 603 | 0 | 2223 |
| ENSG00000252139 | SCARNA18 | 517 | 971 | 473 | 801 | 0 | 603 | 349 | 2223 |
| ENSG00000223224 | SNORD71 | 258 | 1166 | 789 | 801 | 0 | 0 | 349 | 0 |
| ENSG00000199673 | SNORD16 | 1422 | 194 | 789 | 801 | 441 | 301 | 349 | 0 |
| ENSG00000212464 | SNORA12 | 1163 | 389 | 2366 | 668 | 1764 | 1506 | 1046 | 556 |
| ENSG00000206952 | SNORA50A | 646 | 777 | 473 | 1069 | 882 | 603 | 349 | 1112 |
| ENSG00000221803 | SNORD23 | 646 | 777 | 631 | 134 | 1323 | 1808 | 1395 | 5003 |
| ENSG00000281910 | SNORA50A | 646 | 777 | 473 | 1069 | 882 | 603 | 349 | 1112 |
| ENSG00000238344 | SNORD126 | 388 | 971 | 0 | 534 | 0 | 301 | 0 | 0 |
| ENSG00000273587 | SNORA78 | 0 | 1166 | 631 | 267 | 441 | 904 | 1046 | 0 |
| ENSG00000252542 | SNORD36C | 646 | 583 | 315 | 267 | 441 | 0 | 349 | 556 |
| ENSG00000206775 | SNORD37 | 388 | 777 | 631 | 534 | 441 | 904 | 0 | 1668 |
| ENSG00000207031 | SNORD59A | 129 | 971 | 473 | 134 | 0 | 301 | 0 | 0 |
| ENSG00000207496 | SNORA7A | 775 | 389 | 473 | 1336 | 0 | 603 | 1046 | 0 |
| ENSG00000206693 | SNORA56 | 517 | 583 | 315 | 134 | 0 | 301 | 0 | 0 |
| ENSG00000202093 | SNORD58C | 517 | 583 | 0 | 267 | 441 | 301 | 349 | 556 |
| ENSG00000221303 | SNORA79 | 646 | 389 | 946 | 801 | 441 | 301 | 349 | 0 |
| ENSG00000264994 | SNORD92 | 646 | 389 | 0 | 1202 | 1323 | 1506 | 2092 | 2223 |
| ENSG00000252835 | SCARNA21 | 646 | 389 | 1577 | 1069 | 882 | 1506 | 349 | 0 |
| ENSG00000222370 | SNORA36B | 388 | 583 | 946 | 1603 | 0 | 301 | 349 | 0 |
| ENSG00000206989 | SNORD63 | 129 | 777 | 315 | 267 | 0 | 0 | 697 | 1668 |
| ENSG00000207217 | SNORA42 | 129 | 777 | 1577 | 401 | 1323 | 2109 | 0 | 1668 |
| ENSG00000222365 | SNORD12B | 517 | 389 | 946 | 1202 | 3088 | 2712 | 2789 | 2779 |
| ENSG00000199436 | SNORD9 | 905 | 0 | 315 | 1737 | 1764 | 301 | 0 | 556 |
| ENSG00000252947 | SCARNA1 | 646 | 194 | 946 | 2004 | 882 | 904 | 1743 | 1668 |
| ENSG00000200463 | SNORD118 | 388 | 389 | 1104 | 1737 | 441 | 1808 | 2092 | 1668 |
| ENSG00000212163 | SNORD91A | 388 | 389 | 315 | 935 | 0 | 301 | 0 | 1668 |
| ENSG00000199744 | SNORD36A | 388 | 389 | 0 | 134 | 441 | 0 | 697 | 1112 |
| ENSG00000201487 | SNORD45B | 129 | 583 | 158 | 1069 | 0 | 301 | 697 | 1668 |
| ENSG00000209702 | SNORD41 | 129 | 583 | 473 | 668 | 0 | 0 | 697 | 0 |
| ENSG00000252213 | SNORA74 | 129 | 583 | 473 | 935 | 441 | 301 | 1743 | 556 |
| ENSG00000207168 | SNORA15 | 775 | 0 | 315 | 401 | 0 | 603 | 697 | 556 |
| ENSG00000277108 | SNORD49B | 775 | 0 | 158 | 0 | 882 | 603 | 2092 | 3335 |
| ENSG00000200785 | SNORD8 | 517 | 194 | 158 | 267 | 441 | 1808 | 349 | 556 |
| ENSG00000206620 | SNORD45C | 517 | 194 | 158 | 401 | 882 | 1205 | 697 | 556 |
| ENSG00000239195 | SNORD5 | 258 | 389 | 315 | 534 | 441 | 301 | 697 | 4447 |
| ENSG00000252906 | SCARNA3 | 258 | 389 | 3470 | 3340 | 882 | 1205 | 1395 | 2779 |
| ENSG00000271852 | SNORD11B | 646 | 0 | 158 | 1069 | 882 | 904 | 0 | 2779 |
| ENSG00000201785 | SNORD117 | 258 | 194 | 631 | 801 | 0 | 603 | 0 | 1112 |
| ENSG00000206754 | SNORD101 | 258 | 194 | 158 | 534 | 0 | 301 | 697 | 1112 |
| ENSG00000238650 | SNORD54 | 258 | 194 | 631 | 401 | 0 | 0 | 697 | 2779 |
| ENSG00000212498 | SNORD86 | 0 | 389 | 158 | 0 | 882 | 0 | 1743 | 2223 |
| ENSG00000221716 | SNORA11 | 0 | 389 | 631 | 401 | 441 | 0 | 0 | 1112 |
| ENSG00000251992 | SCARNA17 | 388 | 0 | 315 | 0 | 0 | 301 | 1395 | 0 |
| ENSG00000253007 | SNORA76 | 258 | 0 | 315 | 134 | 882 | 603 | 0 | 556 |
| ENSG00000281295 | SNORA50 | 258 | 0 | 315 | 134 | 882 | 603 | 0 | 556 |
| ENSG00000206903 | SNORA24 | 0 | 194 | 158 | 0 | 0 | 1506 | 0 | 0 |
| ENSG00000201403 | SNORD14B | 0 | 194 | 0 | 267 | 0 | 301 | 697 | 1112 |
| ENSG00000207093 | SNORD116-8 | 129 | 0 | 2366 | 3874 | 0 | 0 | 1046 | 0 |
| ENSG00000199666 | U3 | 0 | 0 | 473 | 534 | 441 | 603 | 0 | 556 |
| ENSG00000207118 | SNORD14D | 0 | 0 | 473 | 534 | 2647 | 301 | 0 | 0 |
| ENSG00000207245 | SNORD116-29 | 0 | 0 | 631 | 1870 | 441 | 0 | 0 | 0 |
| ENSG00000207375 | SNORD116-23 | 0 | 0 | 158 | 1202 | 0 | 301 | 0 | 556 |
| ENSG00000275529 | SNORD116-4 | 0 | 0 | 631 | 1737 | 0 | 0 | 349 | 0 |
| ENSG00000276314 | SNORD107 | 0 | 0 | 473 | 935 | 0 | 0 | 0 | 556 |
| ENSG00000276610 | SNORD64 | 0 | 0 | 473 | 534 | 0 | 0 | 0 | 0 |
