# Supplementary material for: RNA-seq reveals distinctive RNA profiles of small extracellular vesicles from different human liver cancer cell lines
Source: Oncotarget. 2017 Aug 24;8(47):82920–39. doi: 10.18632/oncotarget.20503 (PMC5669939; doi:10.18632/oncotarget.20503)
Supplement: Supplementary file 7 [file oncotarget-08-82920-s007.docx]

**Table S6. snoRNA**

| **Ensembl Gene ID** | **Gene symbol** | **HuH7-EVs_1** | **HuH7-EVs_2** | **Hep3B-EVs_1** | **Hep3B-EVs_2** | **HepG2-EVs_1** | **HepG2-EVs_2** | **HuH6-EVs_1** | **HuH6-EVs_2** |
| --- | --- | --- | --- | --- | --- | --- | --- | --- | --- |
| **ENSG00000238622** | SNORD97 | 110982 | 76286 | 32568 | 10522 | 50481 | 73820 | 14179 | 24432 |
| **ENSG00000200087** | SNORA73B | 53850 | 38270 | 22052 | 30448 | 37685 | 40245 | 21387 | 27665 |
| **ENSG00000277194** | SNORD22 | 45620 | 32779 | 41358 | 58028 | 45133 | 21674 | 15715 | 19245 |
| **ENSG00000200879** | SNORD14E | 36683 | 32779 | 20294 | 21402 | 35012 | 30890 | 21505 | 9096 |
| **ENSG00000200394** | SNORA38B | 17167 | 51533 | 39631 | 2720 | 10695 | 13338 | 945 | 1504 |
| **ENSG00000206680** | SNORD21 | 46604 | 13179 | 9838 | 37322 | 25145 | 24082 | 35153 | 20899 |
| **ENSG00000209480** | SNORD83B | 17142 | 37763 | 31335 | 15329 | 7066 | 5974 | 12821 | 16990 |
| **ENSG00000209482** | SNORD83A | 15678 | 25682 | 8204 | 8013 | 8912 | 6298 | 11875 | 9096 |
| **ENSG00000212135** | SNORD67 | 26508 | 12672 | 39477 | 38819 | 17824 | 19682 | 9453 | 2481 |
| **ENSG00000199961** | SNORD1B | 28983 | 8195 | 6508 | 21993 | 14259 | 14079 | 4963 | 3308 |
| **ENSG00000207445** | SNORD15B | 23428 | 9546 | 11997 | 29520 | 28773 | 31214 | 23041 | 13381 |
| **ENSG00000206602** | SNORD58A | 17773 | 13601 | 6693 | 14570 | 17761 | 18895 | 12880 | 7442 |
| **ENSG00000238917** | SNORD10 | 24085 | 7181 | 6662 | 17501 | 6875 | 8336 | 8094 | 1654 |
| **ENSG00000281311** | SNORA50 | 9543 | 21543 | 9993 | 5503 | 16869 | 20284 | 1950 | 752 |
| **ENSG00000277887** | SNORA76C | 9543 | 21543 | 9993 | 5503 | 16869 | 20284 | 1950 | 752 |
| **ENSG00000212283** | SNORD89 | 18480 | 12165 | 10054 | 23490 | 50735 | 43301 | 8980 | 8420 |
| **ENSG00000199631** | SNORD33 | 11386 | 18163 | 12830 | 9320 | 13368 | 13291 | 5022 | 5939 |
| **ENSG00000274266** | SNORA73A | 16814 | 12588 | 8420 | 7148 | 14005 | 9772 | 25168 | 23681 |
| **ENSG00000239002** | SCARNA10 | 16435 | 12588 | 22175 | 7275 | 2419 | 3890 | 4845 | 8946 |
| **ENSG00000201754** | SNORD52 | 20374 | 8364 | 17148 | 25662 | 11395 | 14959 | 5199 | 4435 |
| **ENSG00000202400** | SNORD82 | 19389 | 9124 | 7618 | 13115 | 18652 | 19034 | 5376 | 4886 |
| **ENSG00000200983** | SNORA45A | 8786 | 18670 | 14958 | 4449 | 9294 | 11670 | 4431 | 2556 |
| **ENSG00000200084** | SNORD68 | 8281 | 17403 | 6199 | 4470 | 8339 | 7039 | 2009 | 3458 |
| **ENSG00000278249** | SCARNA2 | 14214 | 10983 | 6137 | 8624 | 17888 | 16255 | 6617 | 1804 |
| **ENSG00000275996** | SNORD27 | 14946 | 8955 | 9653 | 9172 | 5220 | 6484 | 12348 | 7969 |
| **ENSG00000231587** | SNORD62B | 14087 | 8870 | 24087 | 10901 | 9676 | 11763 | 43779 | 23305 |
| **ENSG00000275143** | SCARNA16 | 7018 | 14024 | 32075 | 11450 | 14259 | 12087 | 4963 | 8119 |
| **ENSG00000199753** | SNORD104 | 10780 | 9631 | 14804 | 8329 | 12604 | 10327 | 13234 | 12329 |
| **ENSG00000207280** | SNORD20 | 15400 | 4224 | 4904 | 15329 | 31320 | 23526 | 4372 | 12404 |
| **ENSG00000201675** | SNORD32A | 9164 | 8786 | 6878 | 10817 | 10631 | 8336 | 5376 | 4511 |
| **ENSG00000264294** | SNORD55 | 4898 | 9715 | 3763 | 2298 | 2801 | 3334 | 4195 | 6390 |
| **ENSG00000221803** | SNORD23 | 7145 | 7096 | 5736 | 3310 | 4010 | 4122 | 532 | 526 |
| **ENSG00000212304** | SNORD12 | 9063 | 5069 | 4318 | 12567 | 18524 | 17737 | 16306 | 6991 |
| **ENSG00000276788** | SNORD26 | 8558 | 5322 | 6908 | 6052 | 8466 | 7039 | 29599 | 29770 |
| **ENSG00000199673** | SNORD16 | 7877 | 5491 | 5644 | 10901 | 3438 | 4724 | 5081 | 902 |
| **ENSG00000208772** | SNORD94 | 3888 | 9039 | 6569 | 8540 | 10376 | 11022 | 4136 | 5563 |
| **ENSG00000263934** | SNORD3A | 5731 | 6758 | 6230 | 6832 | 22217 | 17367 | 15538 | 31424 |
| **ENSG00000209582** | SNORA48 | 4393 | 7772 | 6970 | 3395 | 2865 | 2732 | 4963 | 1654 |
| **ENSG00000264940** | SNORD3C | 4898 | 6758 | 5366 | 6747 | 21007 | 17089 | 12525 | 27891 |
| **ENSG00000200623** | SNORD18A | 5706 | 5745 | 3208 | 6895 | 5793 | 5048 | 2718 | 1579 |
| **ENSG00000207241** | SNORD45A | 7321 | 3717 | 3084 | 7696 | 5538 | 5511 | 17901 | 7593 |
| **ENSG00000201403** | SNORD14B | 5933 | 5069 | 3084 | 2425 | 1082 | 1991 | 1241 | 2180 |
| **ENSG00000221500** | SNORD100 | 4292 | 6674 | 11103 | 7886 | 2610 | 2408 | 18788 | 11502 |
| **ENSG00000263764** | SNORD43 | 6968 | 3126 | 4935 | 6769 | 1337 | 2269 | 5022 | 2706 |
| **ENSG00000277947** | SNORD3D | 4393 | 5660 | 4750 | 4976 | 20180 | 17228 | 12584 | 25335 |
| **ENSG00000281000** | SNORD3D | 4393 | 5660 | 4750 | 4976 | 20180 | 17228 | 12584 | 25335 |
| **ENSG00000200026** | U8 | 3736 | 6167 | 1450 | 928 | 2164 | 4075 | 1654 | 6315 |
| **ENSG00000272034** | SNORD14A | 4292 | 5407 | 2683 | 1181 | 1528 | 1852 | 3427 | 2481 |
| **ENSG00000202314** | SNORD6 | 4191 | 5491 | 5274 | 5946 | 4265 | 8429 | 8389 | 11051 |
| **ENSG00000265185** | SNORD3B-1 | 4166 | 5322 | 3238 | 4407 | 16042 | 12458 | 12052 | 22327 |
| **ENSG00000226572** | SNORD57 | 4443 | 4984 | 5274 | 4660 | 5666 | 2269 | 17901 | 11126 |
| **ENSG00000212158** | SNORD66 | 4923 | 4309 | 4965 | 3332 | 5475 | 7132 | 6144 | 6916 |
| **ENSG00000277864** | SCARNA15 | 6438 | 2788 | 2251 | 2994 | 24381 | 25240 | 20501 | 67960 |
| **ENSG00000221420** | SNORA81 | 4115 | 5069 | 8296 | 14866 | 4392 | 5187 | 3486 | 7067 |
| **ENSG00000275043** | SNORD25 | 3787 | 4900 | 6076 | 3901 | 2419 | 2454 | 10221 | 8645 |
| **ENSG00000200354** | SNORA71D | 1818 | 5998 | 1357 | 843 | 2801 | 1343 | 591 | 1955 |
| **ENSG00000206811** | SNORA10 | 3459 | 4140 | 1172 | 1075 | 1591 | 1204 | 945 | 677 |
| **ENSG00000208797** | SNORD73A | 3105 | 4224 | 2375 | 4365 | 3056 | 3103 | 11225 | 10299 |
| **ENSG00000202503** | SNORD34 | 4191 | 3126 | 2961 | 6473 | 4392 | 4400 | 4254 | 2857 |
| **ENSG00000200320** | SNORA63 | 2727 | 4562 | 7309 | 4660 | 3438 | 3149 | 5140 | 6841 |
| **ENSG00000264346** | SNORA77 | 3913 | 2365 | 2344 | 1286 | 509 | 1575 | 1063 | 1052 |
| **ENSG00000200463** | SNORD118 | 2348 | 3886 | 1912 | 3479 | 2546 | 3334 | 3249 | 6991 |
| **ENSG00000235408** | SNORA71B | 2651 | 3548 | 4534 | 5461 | 4583 | 3427 | 2659 | 1654 |
| **ENSG00000199436** | SNORD9 | 3812 | 2365 | 2344 | 3690 | 2483 | 2316 | 1063 | 752 |
| **ENSG00000252010** | SCARNA5 | 4065 | 1943 | 3732 | 10121 | 3247 | 2686 | 7680 | 4360 |
| **ENSG00000220988** | SNORD88C | 3585 | 2365 | 1881 | 3774 | 2483 | 1899 | 2068 | 677 |
| **ENSG00000212232** | SNORD17 | 2701 | 3210 | 4904 | 3479 | 4138 | 3612 | 15066 | 6841 |
| **ENSG00000238942** | SNORD2 | 3711 | 2197 | 13046 | 14844 | 1464 | 1482 | 27000 | 15411 |
| **ENSG00000212588** | SNORA26 | 2499 | 3041 | 1974 | 4618 | 637 | 463 | 886 | 752 |
| **ENSG00000206620** | SNORD45C | 1616 | 3633 | 1450 | 2024 | 2865 | 2130 | 4608 | 3308 |
| **ENSG00000238795** | SCARNA12 | 2222 | 2957 | 1758 | 780 | 1082 | 973 | 709 | 601 |
| **ENSG00000206611** | SNORD24 | 1969 | 3126 | 1388 | 1202 | 637 | 880 | 8153 | 5488 |
| **ENSG00000202252** | SNORD14C | 2247 | 2534 | 2622 | 3353 | 3119 | 2964 | 8980 | 12555 |
| **ENSG00000281147** | SNORD50A | 2222 | 2112 | 1326 | 1982 | 637 | 834 | 1359 | 2105 |
| **ENSG00000200706** | SNORD45 | 1792 | 2450 | 1295 | 1392 | 1591 | 1852 | 8094 | 3383 |
| **ENSG00000212447** | SNORD90 | 2853 | 1267 | 2498 | 9193 | 2164 | 1389 | 709 | 1504 |
| **ENSG00000201457** | SNORA55 | 1666 | 2281 | 2375 | 2910 | 3183 | 3242 | 1123 | 1353 |
| **ENSG00000200534** | SNORA33 | 2373 | 1521 | 2622 | 3985 | 1719 | 1806 | 1713 | 3909 |
| **ENSG00000273544** | SNORA44 | 1262 | 2450 | 1789 | 675 | 1973 | 1389 | 1713 | 1504 |
| **ENSG00000280498** | SNORA16A | 1490 | 2112 | 2776 | 485 | 955 | 880 | 2718 | 2556 |
| **ENSG00000274582** | SNORA16A | 1490 | 2112 | 2776 | 485 | 955 | 880 | 2718 | 2556 |
| **ENSG00000222370** | SNORA36B | 959 | 2619 | 1542 | 3943 | 2610 | 2223 | 3840 | 11953 |
| **ENSG00000206799** | SNORA32 | 2070 | 1436 | 1450 | 1392 | 1337 | 1667 | 236 | 1353 |
| **ENSG00000221539** | SNORD99 | 2045 | 1436 | 987 | 2256 | 1209 | 1482 | 13293 | 8119 |
| **ENSG00000207008** | SNORA54 | 1515 | 1943 | 2837 | 1054 | 1655 | 1019 | 650 | 752 |
| **ENSG00000275072** | SNORD50B | 1893 | 1352 | 524 | 611 | 2737 | 3520 | 5317 | 7292 |
| **ENSG00000212452** | SNORD69 | 1464 | 1774 | 1357 | 1792 | 1146 | 1760 | 8567 | 12404 |
| **ENSG00000277512** | SNORD65 | 1792 | 1436 | 1203 | 1181 | 446 | 880 | 4845 | 3684 |
| **ENSG00000278274** | SNORA61 | 1086 | 2112 | 1234 | 464 | 3883 | 2269 | 2600 | 3533 |
| **ENSG00000221241** | SNORD88A | 2070 | 1098 | 1295 | 2193 | 2992 | 2454 | 591 | 601 |
| **ENSG00000238423** | SNORD42B | 1010 | 2112 | 894 | 1476 | 509 | 1065 | 1950 | 2481 |
| **ENSG00000202031** | SNORD38A | 682 | 2365 | 1110 | 401 | 1082 | 1297 | 2954 | 2556 |
| **ENSG00000251898** | SCARNA11 | 884 | 2112 | 4441 | 464 | 891 | 1482 | 473 | 752 |
| **ENSG00000206630** | SNORD60 | 1136 | 1690 | 679 | 886 | 1146 | 1204 | 1772 | 1804 |
| **ENSG00000281010** | snoR1 | 1136 | 1690 | 679 | 886 | 1146 | 1204 | 1772 | 1804 |
| **ENSG00000278261** | SNORD1A | 1969 | 845 | 308 | 1118 | 1082 | 880 | 768 | 677 |
| **ENSG00000206941** | SNORD15A | 1591 | 1183 | 1604 | 1919 | 2292 | 1436 | 6322 | 3608 |
| **ENSG00000239127** | SNORD125 | 1414 | 1352 | 1018 | 1054 | 1019 | 926 | 1182 | 1579 |
| **ENSG00000265145** | SNORD53 | 1641 | 1098 | 524 | 759 | 1846 | 1714 | 3604 | 3157 |
| **ENSG00000222365** | SNORD12B | 1717 | 1014 | 1727 | 1560 | 1973 | 1945 | 2777 | 4285 |
| **ENSG00000212607** | SNORA45B | 1439 | 1183 | 3146 | 2256 | 3056 | 2686 | 2186 | 3233 |
| **ENSG00000277985** | SNORA67 | 909 | 1690 | 1264 | 886 | 573 | 695 | 886 | 376 |
| **ENSG00000276314** | SNORD107 | 303 | 2281 | 11812 | 2826 | 2483 | 3983 | 886 | 2180 |
| **ENSG00000280496** | SNORA43 | 1060 | 1521 | 802 | 611 | 764 | 1065 | 1418 | 902 |
| **ENSG00000276161** | SNORA43 | 1060 | 1521 | 802 | 611 | 764 | 1065 | 1418 | 902 |
| **ENSG00000275146** | snoU2_19 | 2146 | 422 | 679 | 780 | 955 | 370 | 295 | 601 |
| **ENSG00000207421** | SNORD38B | 833 | 1690 | 1018 | 590 | 1209 | 834 | 9866 | 9322 |
| **ENSG00000281859** | SNORD38B | 833 | 1690 | 1018 | 590 | 1209 | 834 | 9866 | 9322 |
| **ENSG00000271982** | SNORD58B | 1212 | 1267 | 1141 | 1286 | 1591 | 1158 | 1772 | 1504 |
| **ENSG00000252139** | SCARNA18 | 1212 | 1267 | 864 | 1096 | 637 | 741 | 1536 | 1052 |
| **ENSG00000199477** | SNORA31 | 606 | 1859 | 339 | 232 | 891 | 926 | 473 | 677 |
| **ENSG00000207088** | SNORA7B | 808 | 1605 | 1018 | 949 | 573 | 556 | 768 | 226 |
| **ENSG00000199293** | SNORA21 | 1060 | 1352 | 2807 | 1371 | 3310 | 3520 | 5317 | 2932 |
| **ENSG00000201302** | SNORA65 | 1414 | 929 | 2282 | 675 | 3183 | 2408 | 3190 | 2706 |
| **ENSG00000207405** | SNORA64 | 732 | 1605 | 524 | 232 | 573 | 278 | 118 | 301 |
| **ENSG00000277846** | SNORD30 | 959 | 1352 | 1789 | 2783 | 637 | 880 | 4254 | 2631 |
| **ENSG00000238649** | SNORD42A | 858 | 1436 | 524 | 822 | 509 | 695 | 4017 | 4511 |
| **ENSG00000239183** | SNORA84 | 732 | 1521 | 956 | 1075 | 955 | 1343 | 945 | 677 |
| **ENSG00000201348** | RNU105B | 631 | 1521 | 709 | 422 | 1082 | 1065 | 473 | 2706 |
| **ENSG00000212443** | SNORA53 | 1212 | 929 | 1511 | 2130 | 1273 | 1204 | 2422 | 1052 |
| **ENSG00000206592** | SNORA18 | 25 | 2112 | 278 | 42 | 0 | 370 | 118 | 0 |
| **ENSG00000223224** | SNORD71 | 530 | 1605 | 1141 | 1476 | 891 | 602 | 827 | 1278 |
| **ENSG00000207233** | SNORA37 | 1161 | 929 | 1696 | 590 | 509 | 417 | 532 | 301 |
| **ENSG00000264549** | SNORD95 | 1212 | 845 | 1326 | 1265 | 1019 | 973 | 7740 | 5638 |
| **ENSG00000239043** | SNORD127 | 1288 | 760 | 802 | 2319 | 1019 | 370 | 473 | 451 |
| **ENSG00000201823** | SNORD48 | 581 | 1436 | 2190 | 1497 | 828 | 880 | 2186 | 1729 |
| **ENSG00000265236** | SNORD84 | 1060 | 929 | 2436 | 2277 | 1782 | 1436 | 4549 | 3308 |
| **ENSG00000201998** | SNORA23 | 884 | 1098 | 1665 | 1539 | 1591 | 1760 | 945 | 1353 |
| **ENSG00000200259** | SNORD35A | 1111 | 845 | 1018 | 1687 | 700 | 741 | 1713 | 1052 |
| **ENSG00000249784** | SCARNA22 | 808 | 1098 | 3454 | 1413 | 1591 | 1852 | 59 | 601 |
| **ENSG00000272296** | SNORD96A | 959 | 929 | 1542 | 843 | 700 | 648 | 3722 | 3007 |
| **ENSG00000201847** | SNORD31 | 1035 | 845 | 709 | 1012 | 255 | 232 | 650 | 376 |
| **ENSG00000206622** | SNORA69 | 1086 | 760 | 370 | 380 | 191 | 556 | 1359 | 827 |
| **ENSG00000251791** | SCARNA6 | 1136 | 591 | 1079 | 1392 | 1719 | 1899 | 3072 | 1353 |
| **ENSG00000225091** | SNORA71A | 707 | 1014 | 493 | 295 | 955 | 602 | 1359 | 1052 |
| **ENSG00000207392** | SNORA20 | 707 | 1014 | 864 | 1265 | 382 | 46 | 354 | 150 |
| **ENSG00000262074** | SNORD3B-2 | 505 | 1183 | 524 | 822 | 2737 | 2593 | 827 | 5262 |
| **ENSG00000221491** | SNORA34 | 757 | 929 | 1450 | 1118 | 509 | 880 | 532 | 677 |
| **ENSG00000207166** | SNORA68 | 328 | 1352 | 679 | 633 | 318 | 695 | 2068 | 977 |
| **ENSG00000200959** | SNORA74A | 909 | 760 | 1018 | 1075 | 1082 | 880 | 1772 | 827 |
| **ENSG00000221116** | SNORD110 | 530 | 1098 | 401 | 1096 | 891 | 648 | 4963 | 2857 |
| **ENSG00000274998** | SNORA17 | 353 | 1267 | 401 | 232 | 127 | 93 | 1241 | 2406 |
| **ENSG00000281808** | SNORA17 | 353 | 1267 | 401 | 232 | 127 | 93 | 1241 | 2406 |
| **ENSG00000206838** | SNORA5A | 833 | 760 | 2776 | 1877 | 891 | 926 | 354 | 601 |
| **ENSG00000280554** | snoU18 | 808 | 760 | 401 | 780 | 700 | 880 | 2363 | 1579 |
| **ENSG00000202529** | SNORD18B | 808 | 760 | 401 | 780 | 700 | 880 | 2363 | 1579 |
| **ENSG00000252835** | SCARNA21 | 126 | 1436 | 93 | 42 | 255 | 185 | 177 | 0 |
| **ENSG00000277370** | SNORD49A | 1035 | 422 | 771 | 843 | 382 | 741 | 4845 | 2932 |
| **ENSG00000202363** | SNORA62 | 404 | 1014 | 247 | 316 | 764 | 926 | 414 | 451 |
| **ENSG00000200785** | SNORD8 | 656 | 760 | 2683 | 5145 | 1082 | 3195 | 354 | 2105 |
| **ENSG00000201512** | SNORA71C | 379 | 1014 | 463 | 401 | 1019 | 1065 | 354 | 526 |
| **ENSG00000207165** | SNORA70 | 50 | 1267 | 123 | 84 | 318 | 185 | 532 | 0 |
| **ENSG00000207274** | SNORA70 | 278 | 1014 | 278 | 84 | 64 | 139 | 59 | 1879 |
| **ENSG00000274544** | SNORD28 | 353 | 929 | 432 | 527 | 127 | 463 | 5022 | 3533 |
| **ENSG00000272533** | SNORA28 | 606 | 676 | 802 | 696 | 828 | 417 | 591 | 977 |
| **ENSG00000209645** | SNORD105 | 505 | 760 | 62 | 380 | 446 | 232 | 1004 | 150 |
| **ENSG00000221740** | SNORD93 | 1010 | 253 | 1388 | 2488 | 318 | 695 | 0 | 0 |
| **ENSG00000207181** | SNORA14B | 581 | 676 | 987 | 590 | 764 | 324 | 2186 | 1353 |
| **ENSG00000199977** | SNORA73 | 50 | 1183 | 31 | 63 | 127 | 417 | 236 | 150 |
| **ENSG00000207475** | SNORA80E | 303 | 929 | 1943 | 1012 | 318 | 232 | 1241 | 226 |
| **ENSG00000200530** | SNORD35B | 606 | 591 | 740 | 1919 | 637 | 93 | 473 | 1428 |
| **ENSG00000206597** | SNORA57 | 177 | 1014 | 370 | 232 | 255 | 509 | 532 | 0 |
| **ENSG00000206979** | SNORD61 | 833 | 338 | 833 | 1118 | 446 | 741 | 3190 | 1278 |
| **ENSG00000201009** | SNORD46 | 202 | 929 | 93 | 253 | 191 | 139 | 236 | 75 |
| **ENSG00000238531** | SNORD105B | 707 | 422 | 925 | 1118 | 764 | 1204 | 1359 | 677 |
| **ENSG00000206885** | SNORA75 | 353 | 760 | 493 | 295 | 1655 | 787 | 1477 | 1052 |
| **ENSG00000201129** | SNORA58 | 606 | 507 | 1141 | 1581 | 318 | 278 | 0 | 526 |
| **ENSG00000202093** | SNORD58C | 177 | 929 | 586 | 232 | 318 | 463 | 236 | 301 |
| **ENSG00000207406** | SNORA41 | 505 | 591 | 123 | 527 | 446 | 417 | 532 | 902 |
| **ENSG00000249020** | SNORA58 | 404 | 676 | 864 | 611 | 509 | 556 | 118 | 150 |
| **ENSG00000239039** | SNORD13 | 429 | 591 | 154 | 1139 | 446 | 648 | 473 | 1278 |
| **ENSG00000207297** | SNORD7 | 328 | 676 | 833 | 1096 | 382 | 370 | 1063 | 677 |
| **ENSG00000209042** | SNORD12C | 581 | 422 | 1295 | 1560 | 1273 | 509 | 8803 | 4210 |
| **ENSG00000207093** | SNORD116-8 | 303 | 676 | 61621 | 68761 | 6175 | 9679 | 3427 | 4511 |
| **ENSG00000207313** | SNORA2B | 631 | 338 | 586 | 907 | 318 | 139 | 1536 | 376 |
| **ENSG00000238741** | SCARNA7 | 606 | 338 | 1234 | 1371 | 1019 | 787 | 709 | 2330 |
| **ENSG00000251733** | SCARNA8 | 126 | 760 | 1172 | 295 | 255 | 648 | 473 | 827 |
| **ENSG00000238578** | SNORD4A | 379 | 507 | 833 | 928 | 64 | 93 | 650 | 601 |
| **ENSG00000207468** | SNORA19 | 278 | 591 | 278 | 380 | 382 | 46 | 650 | 977 |
| **ENSG00000239195** | SNORD5 | 757 | 84 | 648 | 590 | 255 | 185 | 650 | 1504 |
| **ENSG00000207145** | SNORA18 | 732 | 84 | 432 | 1392 | 446 | 880 | 295 | 376 |
| **ENSG00000207112** | SNORA25 | 429 | 338 | 555 | 485 | 764 | 1528 | 1950 | 1955 |
| **ENSG00000206760** | SNORA6 | 429 | 338 | 740 | 422 | 891 | 509 | 1477 | 902 |
| **ENSG00000252481** | SCARNA13 | 76 | 676 | 1203 | 780 | 1019 | 1111 | 118 | 226 |
| **ENSG00000280466** | SCARNA15 | 328 | 422 | 1573 | 822 | 509 | 463 | 532 | 150 |
| **ENSG00000281394** | SCARNA4 | 328 | 422 | 1573 | 822 | 509 | 463 | 532 | 150 |
| **ENSG00000252906** | SCARNA3 | 50 | 676 | 586 | 696 | 255 | 93 | 118 | 226 |
| **ENSG00000206775** | SNORD37 | 278 | 422 | 925 | 1075 | 191 | 139 | 59 | 226 |
| **ENSG00000206948** | SNORA36A | 353 | 338 | 524 | 211 | 573 | 648 | 945 | 1504 |
| **ENSG00000222489** | SNORA79 | 353 | 338 | 463 | 232 | 1209 | 1158 | 0 | 226 |
| **ENSG00000207062** | SNORA15 | 177 | 507 | 1018 | 759 | 191 | 278 | 59 | 0 |
| **ENSG00000209702** | SNORD41 | 404 | 253 | 586 | 527 | 382 | 463 | 768 | 752 |
| **ENSG00000207217** | SNORA42 | 227 | 422 | 2375 | 527 | 1337 | 1111 | 0 | 226 |
| **ENSG00000254341** | SNORD87 | 631 | 0 | 339 | 274 | 0 | 232 | 2245 | 2556 |
| **ENSG00000207523** | SNORA66 | 429 | 169 | 1912 | 1033 | 1464 | 370 | 473 | 150 |
| **ENSG00000207047** | SNORD51 | 505 | 84 | 679 | 907 | 446 | 370 | 2600 | 1203 |
| **ENSG00000281780** | snoZ196 | 505 | 84 | 679 | 907 | 446 | 324 | 2600 | 1128 |
| **ENSG00000273885** | snoU2-30 | 303 | 253 | 1388 | 633 | 382 | 695 | 236 | 677 |
| **ENSG00000206785** | SNORA15 | 126 | 422 | 864 | 464 | 127 | 278 | 118 | 0 |
| **ENSG00000200816** | SNORA38 | 252 | 253 | 1295 | 590 | 637 | 417 | 0 | 75 |
| **ENSG00000212511** | U3 | 480 | 0 | 370 | 190 | 509 | 741 | 709 | 226 |
| **ENSG00000252542** | SNORD36C | 126 | 338 | 93 | 190 | 127 | 139 | 945 | 1052 |
| **ENSG00000265706** | SNORD53_SNORD92 | 177 | 253 | 586 | 295 | 382 | 1065 | 1359 | 1278 |
| **ENSG00000229686** | SNORD56 | 252 | 169 | 123 | 886 | 255 | 417 | 5081 | 3984 |
| **ENSG00000201785** | SNORD117 | 328 | 84 | 1018 | 696 | 255 | 973 | 2304 | 1128 |
| **ENSG00000207304** | SNORA8 | 50 | 338 | 216 | 148 | 0 | 278 | 768 | 451 |
| **ENSG00000271798** | SNORA51 | 126 | 253 | 278 | 295 | 637 | 1250 | 1477 | 2706 |
| **ENSG00000200831** | SNORD36B | 328 | 0 | 154 | 190 | 0 | 46 | 2481 | 1955 |
| **ENSG00000206989** | SNORD63 | 126 | 169 | 62 | 105 | 382 | 46 | 1300 | 902 |
| **ENSG00000238862** | SNORD19B | 202 | 84 | 154 | 358 | 0 | 0 | 827 | 902 |
| **ENSG00000200418** | SNORA63 | 76 | 169 | 123 | 127 | 64 | 139 | 1536 | 0 |
| **ENSG00000212163** | SNORD91A | 227 | 0 | 216 | 232 | 0 | 185 | 591 | 752 |
| **ENSG00000238317** | SNORD11 | 227 | 0 | 93 | 211 | 0 | 463 | 650 | 451 |
| **ENSG00000264994** | SNORD92 | 126 | 84 | 185 | 590 | 573 | 556 | 1004 | 1278 |
| **ENSG00000207118** | SNORD14D | 126 | 84 | 21250 | 25071 | 7384 | 16302 | 118 | 752 |
| **ENSG00000199574** | SNORD18C | 101 | 84 | 123 | 401 | 255 | 93 | 414 | 601 |
| **ENSG00000274091** | SNORD1C | 177 | 0 | 93 | 316 | 318 | 232 | 945 | 827 |
| **ENSG00000200913** | SNORD46 | 0 | 169 | 247 | 127 | 0 | 0 | 295 | 827 |
| **ENSG00000252213** | SNORA74 | 76 | 84 | 31 | 358 | 127 | 232 | 1123 | 526 |
| **ENSG00000202269** | U8 | 151 | 0 | 123 | 42 | 64 | 324 | 1182 | 601 |
| **ENSG00000207133** | SNORD116-7 | 25 | 84 | 8420 | 4048 | 1528 | 3149 | 650 | 1203 |
| **ENSG00000222345** | SNORD19 | 101 | 0 | 31 | 84 | 0 | 93 | 709 | 376 |
| **ENSG00000238597** | SNORD4B | 101 | 0 | 62 | 127 | 0 | 46 | 945 | 75 |
| **ENSG00000207063** | SNORD116-1 | 0 | 84 | 9838 | 3542 | 1591 | 4075 | 473 | 2706 |
| **ENSG00000239169** | SNORD109B | 0 | 84 | 2375 | 3585 | 255 | 139 | 1300 | 451 |
| **ENSG00000207460** | SNORD116-19 | 0 | 84 | 1357 | 633 | 127 | 417 | 59 | 150 |
| **ENSG00000278715** | SNORD116-20 | 0 | 84 | 987 | 654 | 318 | 556 | 59 | 75 |
| **ENSG00000206621** | SNORD116-14 | 0 | 84 | 987 | 105 | 64 | 93 | 0 | 0 |
| **ENSG00000200496** | U8 | 76 | 0 | 123 | 232 | 64 | 278 | 1300 | 3082 |
| **ENSG00000206754** | SNORD101 | 50 | 0 | 370 | 970 | 64 | 370 | 236 | 827 |
| **ENSG00000238650** | SNORD54 | 25 | 0 | 185 | 105 | 127 | 46 | 1300 | 1128 |
| **ENSG00000206727** | SNORD116-9 | 25 | 0 | 9808 | 4470 | 1464 | 2964 | 473 | 1428 |
| **ENSG00000275529** | SNORD116-4 | 0 | 0 | 11689 | 9868 | 1337 | 2593 | 1654 | 1955 |
| **ENSG00000207191** | SNORD116-5 | 0 | 0 | 8728 | 4070 | 1464 | 3103 | 473 | 1052 |
| **ENSG00000207051** | SNORA27 | 0 | 0 | 62 | 21 | 318 | 139 | 236 | 902 |
| **ENSG00000206656** | SNORD116-17 | 0 | 0 | 1295 | 337 | 64 | 232 | 0 | 601 |
| **ENSG00000207263** | SNORD116-16 | 0 | 0 | 648 | 401 | 0 | 46 | 236 | 0 |
| **ENSG00000275127** | SNORD116-22 | 0 | 0 | 864 | 253 | 191 | 417 | 0 | 226 |
| **ENSG00000207174** | SNORD116-15 | 0 | 0 | 1357 | 527 | 127 | 324 | 0 | 150 |
| **ENSG00000207245** | SNORD116-29 | 0 | 0 | 864 | 443 | 0 | 139 | 59 | 0 |
